# Synthesizing Signaling Pathways from Temporal Phosphoproteomic Data

**DOI:** 10.1016/j.celrep.2018.08.085

**Published:** 2018-09-25

**Authors:** Ali Sinan Kӧksal, Kirsten Beck, Dylan R. Cronin, Aaron McKenna, Nathan D. Camp, Saurabh Srivastava, Matthew E. MacGilvray, Rastislav Bodík, Alejandro Wolf-Yadlin, Ernest Fraenkel, Jasmin Fisher, Anthony Gitter

**Affiliations:** 1 Department of Electrical Engineering and Computer Sciences, University of California, Berkeley, Berkeley, CA, USA; 2 Department of Genome Sciences, University of Washington, Seattle, WA, USA; 3 Department of Biostatistics and Medical Informatics, University of Wisconsin-Madison, Madison, WI, USA; 4 Department of Biological Sciences, Bowling Green State University, Bowling Green, OH, USA; 5 Laboratory of Genetics, University of Wisconsin-Madison, Madison, WI, USA; 6 Paul G. Allen Center for Computer Science and Engineering, University of Washington, Seattle, WA, USA; 7 Department of Biological Engineering, Massachusetts Institute of Technology, Cambridge, MA, USA; 8 Microsoft Research, Cambridge, UK; 9 Department of Biochemistry, University of Cambridge, Cambridge, UK; 10 Morgridge Institute for Research, Madison, WI, USA; 11 Present address: Seattle Genetics, Seattle, WA, USA; 12 Lead Contact

## Abstract

We present a method for automatically discovering signaling pathways from time-resolved phosphoproteomic data. The Temporal Pathway Synthesizer (TPS) algorithm uses constraint-solving techniques first developed in the context of formal verification to explore paths in an interaction network. It systematically eliminates all candidate structures for a signaling pathway where a protein is activated or inactivated before its upstream regulators. The algorithm can model more than one hundred thousand dynamic phosphosites and can discover pathway members that are not differentially phosphorylated. By analyzing temporal data, TPS defines signaling cascades without needing to experimentally perturb individual proteins. It recovers known pathways and proposes pathway connections when applied to the human epidermal growth factor and yeast osmotic stress responses. Independent kinase mutant studies validate predicted substrates in the TPS osmotic stress pathway.

## In Brief

Kӧksal et al. present a computational technique, the Temporal Pathway Synthesizer (TPS), that combines time series global phosphoproteomic data and protein-protein interaction networks to reconstruct the vast signaling pathways that control post-translational modifications.

## Graphical Abstract

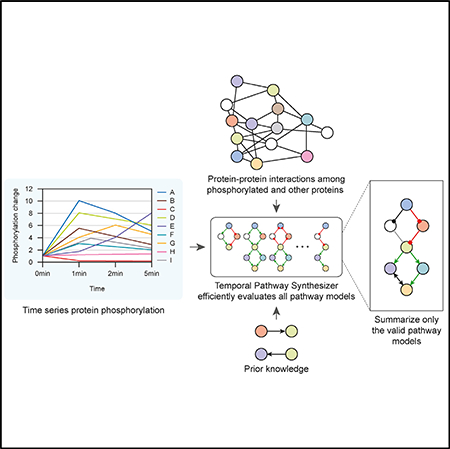

## INTRODUCTION

High-throughput proteomic assays illuminate the amazing breadth and complexity of the signal transduction pathways that cells employ to respond to extracellular cues. These technologies can quantify protein abundance or post-translational modifications (PTMs). Mass spectrometry, in particular, offers a broad view of PTMs, including phosphorylation, ubiquitination, acetylation, and methylation (Choudhary and Mann, 2010), and is not restricted to a predefined list of proteins. Here, we show how to discover new facets of signaling cascades from complex proteomic data by integrating observed PTMs with existing knowledge of protein interactions.

Many gaps persist in our understanding of phosphorylation signaling cascades. For example, our mass spectrometry experiments show that nearly all proteins that are significantly (de)phosphorylated when the epidermal growth factor receptor (EGFR) is stimulated are absent from EGFR pathway maps. The low overlap is consistent with previous temporal phosphoproteomic studies of mammalian signaling (Cao et al., 2012; D’Souza et al., 2014; Humphrey et al., 2015). Discordance between mass spectrometry studies and pathway databases can be caused by extensive crosstalk among pathways (Bauer-Mehren et al., 2009), context-specific interactions (Hill et al., 2017), cell- and tissue-specific protein abundance (Kim et al., 2014), and signaling pathway rewiring (Pawson and Warner, 2007).

Network inference algorithms can explain the phosphorylation events that lie outside of canonical pathways and complement curated pathway maps. Specialized algorithms model time series data, which inform the ordering of phosphorylation changes and support causal instead of correlative modeling (Bar-Joseph et al., 2012). Temporal protein signaling information can be used to reconstruct more accurate and complete networks than a single static snapshot of the phosphoproteome.

A complementary challenge to interpreting off-pathway phosphorylation is that the cellular stimulus response includes mechanisms that are not captured in phosphoproteomic datasets. There is an interplay between phosphorylation changes and other integral parts of signaling cascades. Phosphorylation can affect protein stability, subcellular localization, and recognition of interaction partners (Newman et al., 2014). Phosphoproteomic studies measure only one type of PTM, and not all phosphorylated proteins are detected by mass spectrometry. Additional information is required to infer comprehensive signaling cascades that include non-differentially phosphorylated proteins. Protein-protein interaction (PPI) networks serve this purpose by identifying interactions that connect observed phosphorylation events.

**Figure 1.**
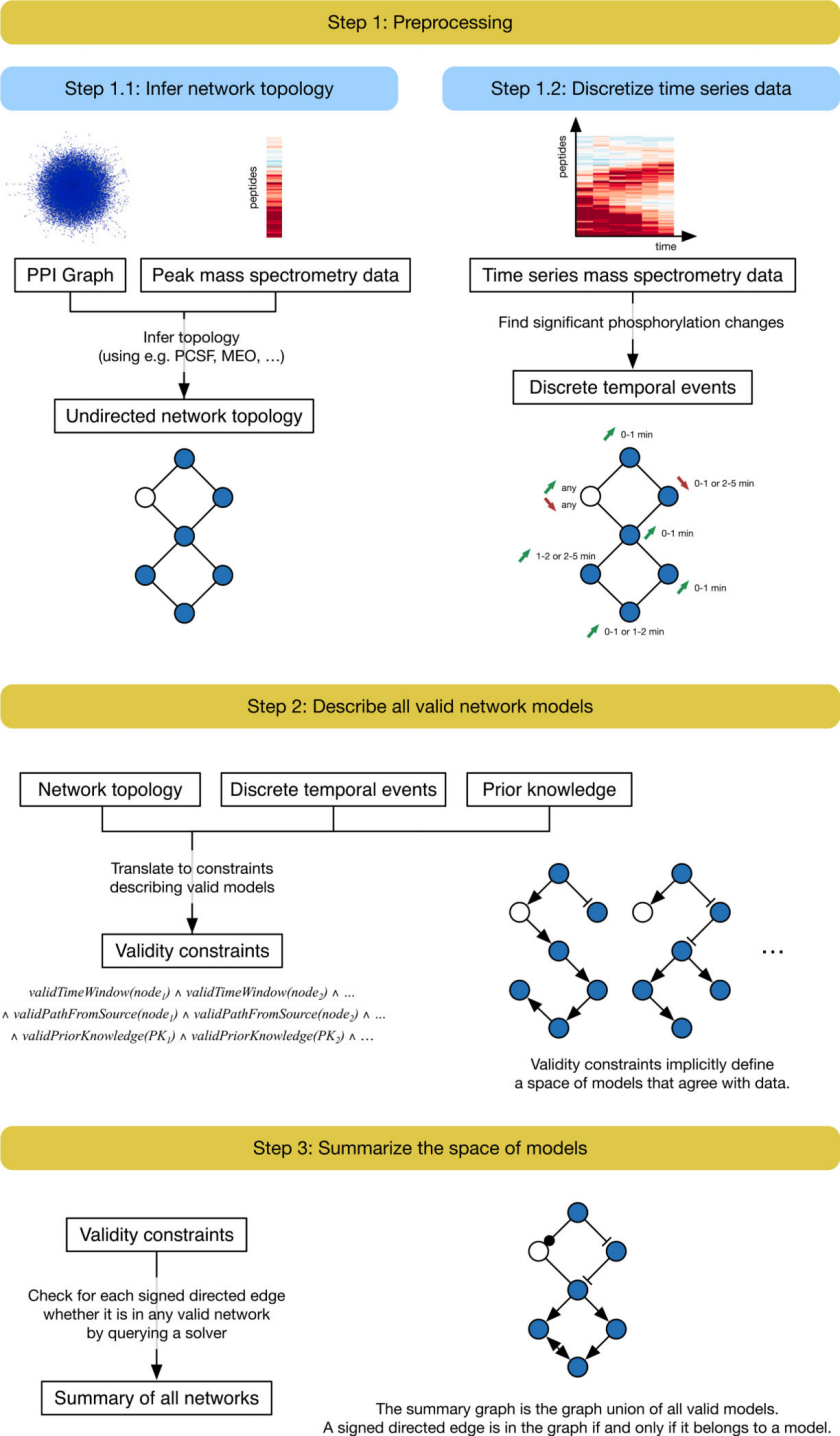
TPS Workflow First, the PPI graph is combined with the phosphorylation data to obtain a condition-specific network (step 1.1). This step does not model the temporal information and instead uses the phosphorylation peak, the highest magnitude fold change. Separately, the time series data are converted into discrete timed signaling events (step 1.2). TPS then defines a space of models that agree with the data by transforming the timed events, undirected network topology, and prior knowledge (kinase-substrate interaction directions in this study) into a set of constraints (step 2). It summarizes the solution space by computing the union of all signed, directed graph models that satisfy the given constraints (step 3). The final pathway model predicts how a subset of generic physical protein interactions coordinates to respond to a specific stimulus in a particular cellular context.

We present the Temporal Pathway Synthesizer (TPS) (Figure 1), a method to assemble temporal phosphoproteomic data into signaling pathways that extend beyond existing canonical maps. “Synthesizer” refers to applying computational program synthesis techniques (Manna and Waldinger, 1980) to produce pathway models from experimental data (Fisher et al., 2014), not synthetic biology (Benner and Sismour, 2005). TPS overcomes both of the aforementioned challenges in interpreting phosphoproteomic data: modeling signaling events that are not captured by pathway databases and including non-phosphorylated proteins in the predicted pathway structures.

TPS first transforms a PPI graph into a condition-specific network by using mass spectrometry data to filter out irrelevant interactions. Next, TPS finds the orientation and sign of edges in the condition-specific interaction graph based on the order of the phosphorylation events. Phosphorylation timing is modeled separately for each observed phosphorylation site on a protein. TPS systematically explores all signed, directed graphs that may explain how signaling messages propagate from the stimulated source protein. Finally, TPS summarizes the valid graphs into a single aggregate network that explicitly tracks confident and ambiguous predictions. Our temporal pathway visualizer tool interactively visualizes the summary network alongside the temporal phosphoproteomic data (Kӧksal et al., 2018).

We study the dynamic signaling responses to human EGF stimulation and yeast osmotic stress. TPS recovers networks that explain how stimulus-responsive proteins are activated or inhibited via chains of physical interactions stemming from the upstream receptors. The highest-confidence TPS predictions are well supported by prior knowledge and consistent with kinase perturbations. These insights into well characterized human and yeast pathways exemplify how TPS can produce condition-specific pathway maps.

## RESULTS

### Quantitative Time Series Phosphoproteomics of EGF Response Captures Widespread Signaling Activity

**Figure 2.**
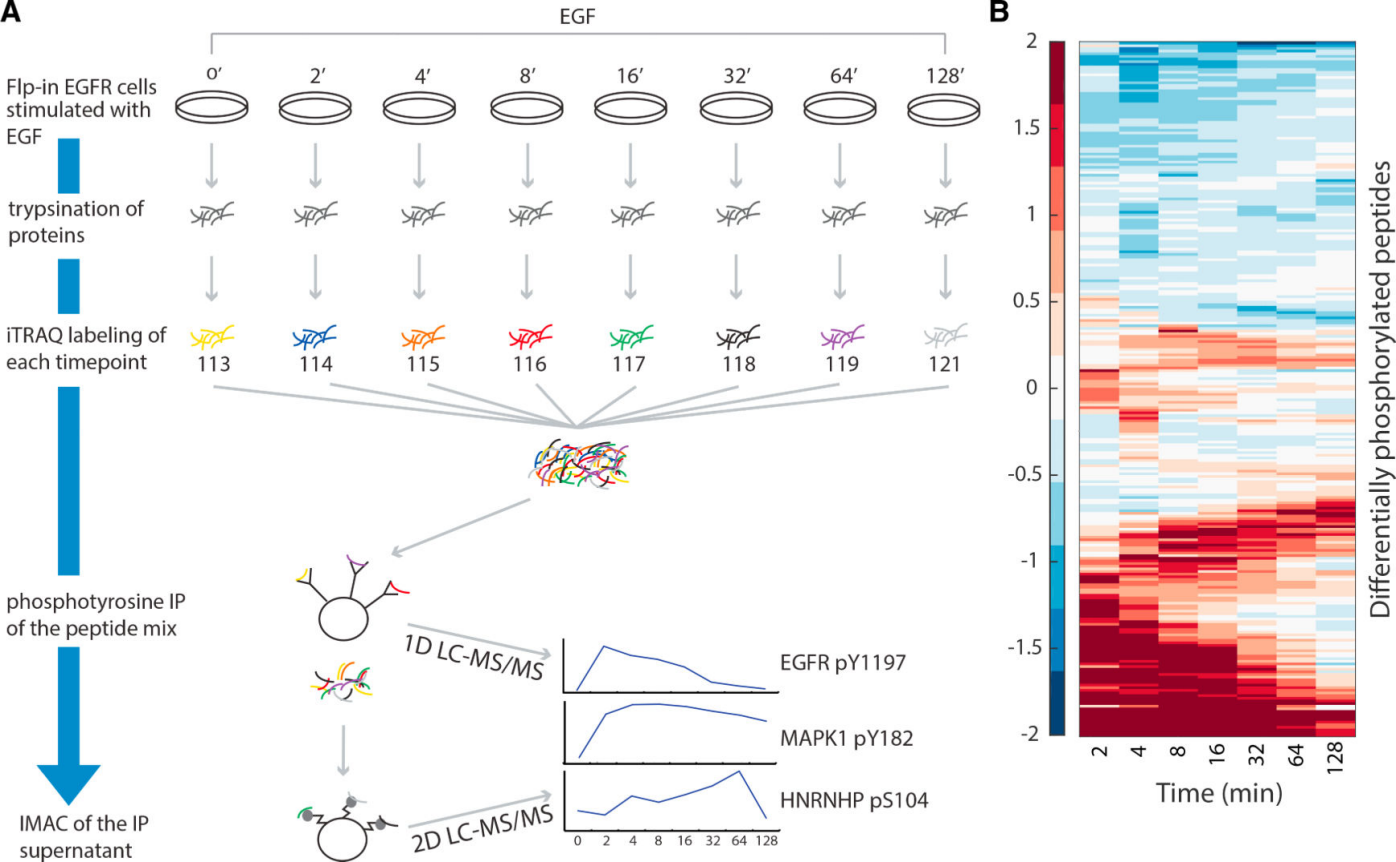
Overview of the EGF Response Proteomics Analysis (A) Cells are stimulated with EGF for 0, 2, 4, 8, 16, 32, 64, or 128 min and then lysed. Cellular protein content is denatured and digested. Peptides are labeled with iTRAQ and mixed. Tyrosine phosphorylated peptides are enriched by immunoprecipitation, and the flowthrough is passed over immobilized metal affinity chromatography to enrich for phosphorylation events on serine and threonine. The phosphotyrosine-rich fraction is analyzed by 1D-LC-MS/MS. The more complex phospho-serine/threonine-rich fraction is analyzed by 2D-LC-MS/MS. Resulting spectra are identified and quantified using Comet. (B) The 263 peptides with significant temporal changes in phosphorylation exhibit distinct types of temporal behaviors (log_2_ fold change with respect to prestimulation intensity). One group of peptides is activated immediately upon stimulation, whereas others display delayed waves of phosphorylation as signals propagate. See also Figures S1 and S2 and Data S1 and S2.

To quantify global EGFR-mediated cellular signaling changes in HEK293 EGFR Flp-In (EGFR Flp-In) cells (Gordus et al., 2009) with phosphoproteomics, we used in-line two-dimensional high-performance liquid chromatography separation (2D-HPLC) coupled to tandem mass spectrometry (MS/MS) (Ficarro et al., 2011; Wolf-Yadlin et al., 2006). We stimulated the cells with EGF for 0, 2, 4, 8, 16, 32, 64, or 128 min and collected three biological replicates with two technical replicates each (Figure 2). We identified 1,068 phosphorylation sites that were detected in all biological replicates (5,442 unique sites detected in at least one replicate), which were then used for TPS network modeling (Data S1 and S2). Phosphorylation intensities were well correlated across the three biological replicates (Kӧksal et al., 2018).

### Reference Pathway Databases Fail to Explain Phosphorylation Changes

We assessed how much of the observed phosphorylation could be explained by existing pathway databases. To obtain a comprehensive view of EGFR-mediated signaling, we collected eight EGFR-related reference pathways (Croft et al., 2014; Gough, 2002; Kandasamy et al., 2010; Kanehisa et al., 2012; Layek et al., 2011; Nishimura, 2001; Schaefer et al., 2009; Supplemental Experimental Procedures). Despite the diversity of the pathway diagrams, they all fail to capture the vast majority of significant phosphorylation events triggered by EGF simulation in our system (Figures S1 and S2). Among the 203 significantly differentially phosphorylated proteins, typically 5% or fewer are present in a reference pathway. 85% of phosphorylated proteins are missing from all of the EGFR-related pathway maps (Figure S1B). Additionally, most of the proteins in the EGFR pathway maps are not differentially phosphorylated (Figure S1A), reflecting a combination of relevant proteins that do not undergo this particular type of PTM, phosphorylation events missed by the mass spectrometry, and interactions that are relevant in some contexts, but not in EGFR Flp-In cells. The low overlaps agree with phosphoproteomic studies of other mammalian signaling pathways. Less than 10% of insulin-regulated proteins were members of a curated insulin pathway (Humphrey et al., 2015). In a study of T cell receptor signaling, only 21% of phosphorylated proteins were known to be involved in the pathway (Cao et al., 2012). Phosphosites regulated by transforming growth factor β (TGF-β) stimulation were not enriched for the TGF- β pathway (D’Souza et al., 2014).

Crosstalk does not explain the low coverage. Most phosphorylated proteins (63%) are not present in the EGFR pathways or any BioCarta, Reactome, or PID pathway (Figure S1B), demonstrating the need for a context-specific representation of EGFR signaling pathway.

### Reconstructing the EGFR Pathway with TPS Explains Temporal Phosphorylation Changes

We applied TPS to model the dynamic signaling response to EGFR stimulation in EGFR Flp-In HEK293 cells. Our workflow consists of three major steps: (1) preprocessing the protein-protein interaction network and temporal phosphorylation data; (2) transforming temporal information, subnetwork structure, and prior knowledge into logical constraints; and (3) summarizing all valid signaling pathway models to discover interactions with unambiguous directions and/or signs (Figure 1).

We first discretized the time series phosphoproteomic data, using Tukey’s honest significant difference (HSD) test (Yandell, 1997) to determine whether a peptide exhibits a significant increase, significant decrease, or no change in phosphorylation at each post-stimulation time point. 263 peptides, corresponding to 203 proteins, significantly change at one or more time points (Kӧksal et al., 2018). Second, we used the prize-collecting Steiner forest (PCSF) (Tuncbag et al., 2013) network algorithm to link the phosphorylated proteins to EGF, the source of stimulation, weighting proteins based on their HSD test significance. PCSF identifies a PPI subnetwork of 316 nodes and 422 edges (Data S3). This subnetwork comprises the interactions through which signaling messages are most likely to propagate. Third, TPS combined the discretized temporal activities of the 263 significantly changing peptides, the PCSF network, and prior knowledge (the orientation of kinase-substrate interactions) to generate a summary of all feasible pathway models (Data S3). Each type of input was translated into logical constraints, which were used to rule out pathway models that are not supported by the data.

**Figure 3.**
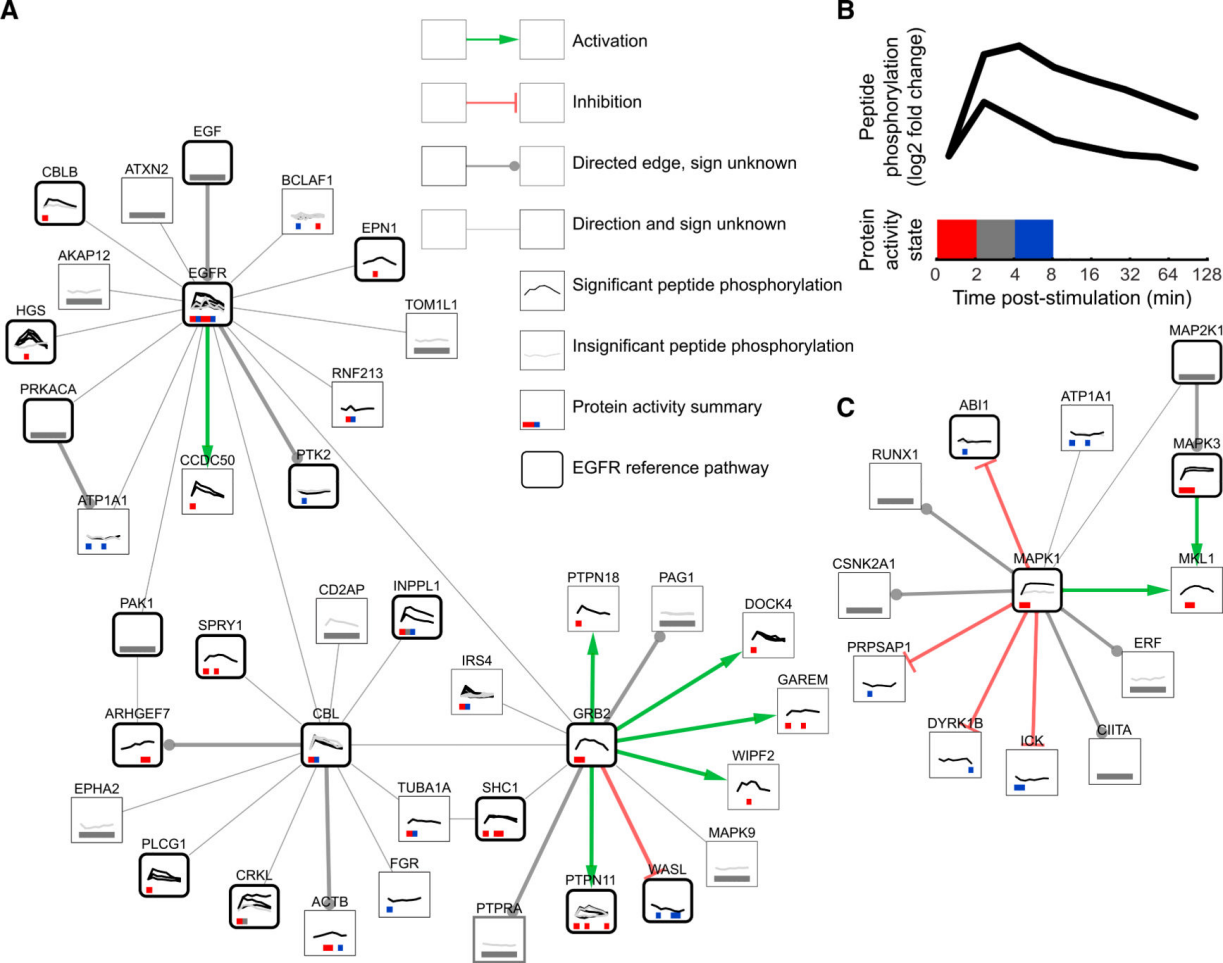
TPS EGF Response Pathway Model Zoomed regions of the full TPS pathway model visualized with Cytoscape (Shannon et al., 2003).(A) The EGFR subnetwork (EGFR, GRB2, CBL, and all their direct neighbors) depicts the proteins that first react to EGF stimulation. A substantial portion (18 of 38 proteins) is known to be associated with EGFR signaling. Green and red edges depict activation and inhibition, respectively. Gray edges that terminate in a circle indicate that the interaction is used in the same direction in all possible pathway models, but the sign is ambiguous. Thin, undirected edges are used in different directions in different valid pathway models. Thick, rounded borders show which proteins are present in one or more reference EGFR pathways. Node annotations are detailed in (B). (B) Line graphs on each protein node show the temporal peptide phosphorylation changes relative to the pre-stimulation level on a log_2_ scale. Multiple lines indicate multiple observed phosphopeptides for that protein, where black lines denote statistically significant phosphorylation changes and gray lines indicate insignificant changes. Proteins without line graphs are connective Steiner nodes inferred by PCSF. Colored boxes summarize the TPS inferred activity state across peptides at each time point. Red indicates activation, blue inhibition, gray ambiguity, and white inactivity. (C) The subnetwork surrounding MAPK1 and MAPK3. TPS correctly determines that MAP2K1 is the kinase that controls both MAPK1 and MAPK3, even though it is not observed in the mass spectrometry data. See also Figures S3 and S4, Table S1, and Data S3 and S4.

In contrast to the reference EGFR pathway diagrams, which capture at most 11% of the differentially phosphorylated proteins, the predicted network from TPS (Figures 3 and S3; Data S3) contains 83% of the responding proteins in its 311 nodes. Each of these proteins is linked to the EGF stimulation with high-confidence protein interactions and has timing that is consistent with the temporal phosphorylation changes of all other proteins in the pathway. These interactions are depicted as directed, signed edges in a graph, where the sign reflects that the proteins have the same (activation) or opposite (inhibition) activity changes. Of the 413 edges in the network, 202 (49%) have a consistent direction in *all of the valid pathway models*, a strong assertion about the confidence in these edge directions. Thirty-eight of the directed edges have a consistent sign as well. The PPI connections, phosphorylation timing, and prior knowledge of kinase-substrate interaction direction all play distinct, important roles in reducing the number of valid pathway models (Kӧksal et al., 2018). The timing of protein activation and inactivation in the TPS pathway reveals a rapid spread of signaling post-stimulation (Kӧksal et al., 2018).

### Prior Evidence Supports EGFR Pathway Predictions

Although nearly all differentially phosphorylated proteins lie outside traditional EGFR pathway representations, 29 (11%) of the 273 phosphorylated proteins and 5 (13%) of the 38 unphosphorylated connective proteins in the TPS network are recognized as EGFR pathway members (Kӧksal et al., 2018). We find strong evidence for many of the predicted directions as well (Kӧksal et al., 2018). In total, 82 of 202 interaction directions are supported by our semi-automated evaluations using EGFR reference pathways, the PhosphoSitePlus input data (Hornbeck et al., 2015), and natural language processing software (Chen and Sharp, 2004; Hoffmann and Valencia, 2004; Poon et al., 2014; Data S3 and S4; Supplemental Experimental Procedures). The vast majority of the remaining directions can neither be confirmed nor refuted (Data S3). Our additional analyses (Kӧksal et al., 2018; Data S3) show that TPS also recovers high-quality pathway models when applied to existing EGF response datasets with lower temporal resolution (Olsen et al., 2006).

### TPS Network Models Can Guide Follow-Up Experiments

The TPS network can be used to prioritize proteins and interactions for additional experimental testing. To illustrate this process, we focused on edges for which the direction or sign were predicted confidently and one of the two proteins is a member of an EGFR reference pathway (Kӧksal et al., 2018). For each interaction, we inhibited the predicted upstream protein and measured the effect on the predicted target’s phosphorylation using western blotting. From our list of ten candidate interactions (Table S1), we selected the three edges for which the antibodies reliably produced clean and quantifiable bands at the right molecular weight: MAPK1-ATP1A1; ABL2 → CRK; and AKT1 → ZYX (zyxin) (Figures 3C and S4). These proteins are already known to physically interact. The novelty of the TPS predictions is the interactions’ relevance to the EGF response. The inhibitors used to inhibit the upstream proteins were SCH772984 for MAPK1, dasatinib for ABL2, and MK-2206 for AKT1. After serum starvation, the cells were treated with an inhibitor for one hour and then stimulated with EGF. We collected data at two time points (denoted short and long; see Figure S4) based on the timing of the phosphorylation events in our mass spectrometry data. Lysates were then assayed by western blot to quantify the level of phosphorylation of the downstream protein.

Dasatinib decreased phosphorylation of CRK (isoform Crk-II) pY221, consistent with the TPS pathway edge (Figure S4). Inhibiting AKT1 increased phosphorylation of Zyxin. In both cases, the predicted interaction direction is supported. MAPK1 inhibition increased ATP1A1 pY10 phosphorylation. The TPS model predicted an inhibitory interaction between these proteins, but the direction was ambiguous. Our data agree with the predicted edge sign and suggest that MAPK1 is upstream of ATP1A1 (Kӧksal et al., 2018). Truly validating the predicted edges would require more direct manipulation of the relevant kinases because Dasatinib is a multi-target inhibitor (Lindauer and Hochhaus, 2014); SCH772984 inhibits both MAPK1 and MAPK3 (Morris et al., 2013); and MK-2206 inhibits AKT1, AKT2, and AKT3 (Yan, 2009). However, these inhibitor experiments demonstrate how TPS can generate testable predictions from global phosphoproteomic data.

### TPS Makes Network Predictions Not Captured by Alternative Approaches

We compared TPS to two existing methods that combine PPI networks and time series data and a third that uses only the phosphorylation data (Supplemental Experimental Procedures). The dynamic Bayesian network (DBN) (Hill et al., 2012) infers posterior peptide-peptide interaction probabilities from time series data and network priors. TimeXNet (Patil et al., 2013) formulates pathway prediction as a network flow problem. FunChisq (Zhang and Song, 2013) uses an adapted chi-square test to detect directed relationships between phosphorylated proteins. Comparing the four predicted EGF response pathway models demonstrates the impact of the diverse algorithmic strategies. Almost all of the protein-protein edges are unique to a single method, and no edges are predicted by all four methods (Kӧksal et al., 2018). Despite greater overlap among the predicted nodes, the four pathways are divergent.

Because most of the differentially phosphorylated proteins are not members of any reference pathway, these pathways cannot be used to assess the overall quality of the predictions. The TimeXNet pathway, the largest of the three predicted networks, generally captures the most reference pathway interactions when ignoring edge direction and sign (Data S4). However, a closer examination that accounts for the predicted interaction direction shows that TPS typically makes the fewest errors, even when controlling for the size of the predicted pathways (Data S4).

### Yeast Osmotic Stress Response Model Recapitulates Known Pathway Structure and Nominates Candidate Rck2 and Cdc28 Substrates

Although they are still not fully characterized, stress-response signaling cascades in the yeast *Saccharomyces cerevisiae* are better understood than their human counterparts and are not subject to cell-type-specific effects. Thus, we applied TPS to model the yeast osmotic stress response to assess its ability to recapitulate this frequently studied pathway and reveal additional interactions. The hyperosmotic stress response is primarily controlled by the high osmolarity glycerol (HOG) pathway. Kanshin et al. (2015) profiled the rapid response to NaCl, an osmotic stressor, measuring phosphorylation changes for 60 s post-stimulation at uniform 5-s intervals. They identified 1,596 phosphorylated proteins, including 1,401 dynamic phosphopeptides on 784 proteins based on their fold changes in the salt stress time series with respect to a control (Table S2). We used these data to construct a TPS pathway model of the early osmotic stress response (Data S3).

**Figure 4.**
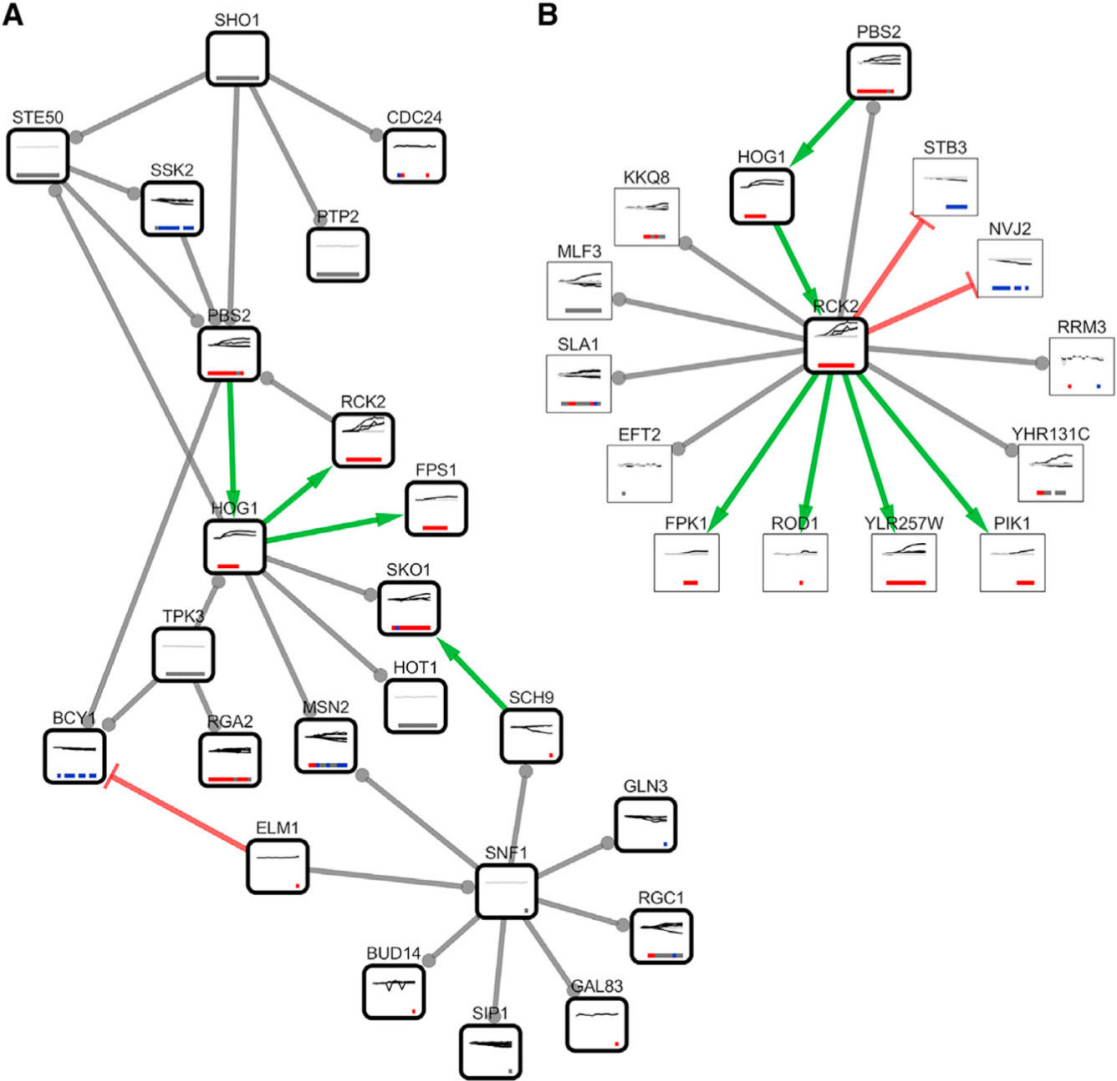
TPS Osmotic Stress Response Pathway Model (A) The portion of the TPS yeast osmotic stress response pathway model for which both proteins are in the osmotic stress reference pathway. TPS correctly recovers the core pathway structure from the Sho1 osmosensor to the primary kinases and transcription factors by ordering proteins based on the phosphorylation timing. Twelve of these pathway interactions are supported by the KEGG high-osmolarity pathway or other literature (Data S4). Node and edge visualizations are as in Figure 3. Note that three interactions (Ste50 → Pbs2, Ste50 → Ssk2, and Rck2 → Pbs2), derived from references (Chasman et al., 2014; Sharifpoor et al., 2011), are not found in other curated versions of the yeast interaction network. (B) A zoomed view of the TPS pathway depicting Rck2 and the proteins it is predicted to interact with. All four proteins predicted to be activated by Rck2—Fpk1, Pik1, Rod1, and YLR257W—displayed decreased phosphorylation in the *RCK2* mutant strain (Romanov et al., 2017), as did predicted targets Mlf3, Sla1, and YHR131C. See also Figure S5 and Data S3 and S4.

The TPS osmotic stress pathway contains 216 proteins and 287 interactions (Figure S5). Thirty-six of these proteins (17%) have been previously annotated as osmotic stress pathway proteins (Kawakami et al., 2016). Focusing on the subset of interactions that connect known HOG pathway members reveals that many of the edges connecting them are correct as well (Figure 4A). TPS recovers the core part of the Kyoto Encyclopedia of Genes and Genomes (KEGG) high-osmolarity pathway, including the interactions Sho1 → Ste50, Sho1 → Cdc24, Sho1 → Pbs2, Ssk2 → Pbs2, and Pbs2 → Hog1 (Data S4). In addition, it correctly places Hog1 as the direct regulator of Rck2 (Romanov et al., 2017) and the transcription factors Hot1, Msn2, and Sko1 (Capaldi et al., 2008). TPS identifies Sch9 as an additional regulator of Sko1 (Pascual-Ahuir and Proft, 2007). Following hyperosmotic shock, Hog1 is recruited to Fps1 (Lee et al., 2013), consistent with the TPS prediction. The predicted feedback from Hog1 to Ste50 is also well supported in osmotic stress (Hao et al., 2008). Many predicted interactions that deviate from the canonical HOG pathway model can be attributed to the input phosphorylation data and background network, not the TPS algorithm (Kӧksal et al., 2018).

After confirming the TPS osmotic stress model agrees well with existing models, we investigated novel candidate pathway members. The TPS model captured the cascade Hog1 → Rck2 → Eft2 (Romanov et al., 2017; Teige et al., 2001) and predicted additional Rck2 targets (Figure 4B). To test these predictions, we compared them to a recent phosphoproteomic study of an *RCK2* mutant subjected to osmotic stress (Romanov et al., 2017). All four proteins that TPS predicts are activated by Rck2 have defective phosphorylation on at least one phosphosite in *rck2*Δ five minutes after osmotic insult (Romanov et al., 2017). Thus, Rck2 likely directly phosphorylates Fpk1, Pik1, Rod1, and YLR257W upon osmotic stress, as TPS predicts. In addition to the four activated substrates, TPS predicts that Rck2 directly regulates seven additional proteins with an ambiguous sign. Three of these seven predicted targets—Mlf3, Sla1, and YHR131C—have a phosphosite that is dependent on Rck2 during osmotic stress (Romanov et al., 2017), supporting the TPS predictions. The three protein-protein edge signs are ambiguous because some phosphosites on the proteins exhibit a significant increase in phosphorylation and others decrease. Similarly, we verified that 67 out of 91 (74%) predicted Cdc28 targets have at least one phosphosite with defective phosphorylation following Cdc28 inhibition (Holt et al., 2009; Kanshin et al., 2017; Kӧksal et al., 2018).

The high-quality TPS osmotic stress pathway demonstrates the algorithm is broadly useful beyond our own EGF stimulation study. It not only recovers many major elements of the classic HOG pathway representation but also prioritizes condition-specific kinase targets that are supported by independent perturbations.

## DISCUSSION

**Figure 5.**
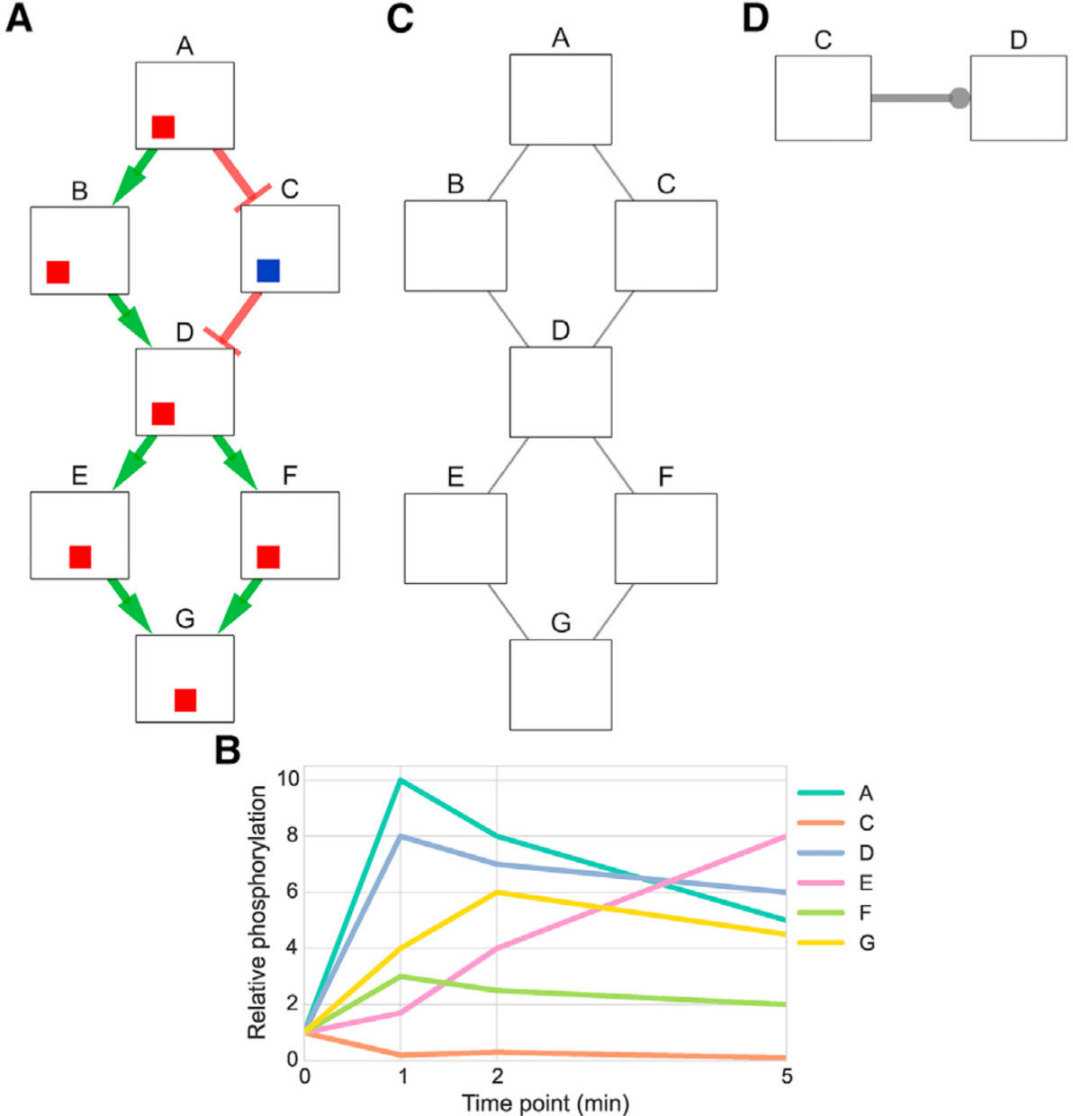
Artificial Example Illustrating the Inputs to TPS (A) The hypothetical signaling pathway that responds to stimulation of node A. The colored boxes on each node show the time at which the protein is activated or inhibited and begins influencing its downstream neighbors, with the leftmost position indicating the earliest time point. Red boxes are increases in activity, blue boxes are decreases, and white boxes are inactive time points, as in Figure 3B. The left position indicates the activity at 0 to 1 min, the center position at 1 to 2 min, and the right position at 2 to 5 min. (B) The first input to TPS is time series phosphorylation data of the response to stimulating node A. (C) The second input is an undirected graph of high-confidence interactions that can recover hidden components that do not appear in the temporal data, such as node B. (D) The last input, which is optional, is prior knowledge of the pathway interactions expressed as (unsigned) directed edges. We represent unsigned edges with a circular arrowhead.

**Figure 6.**
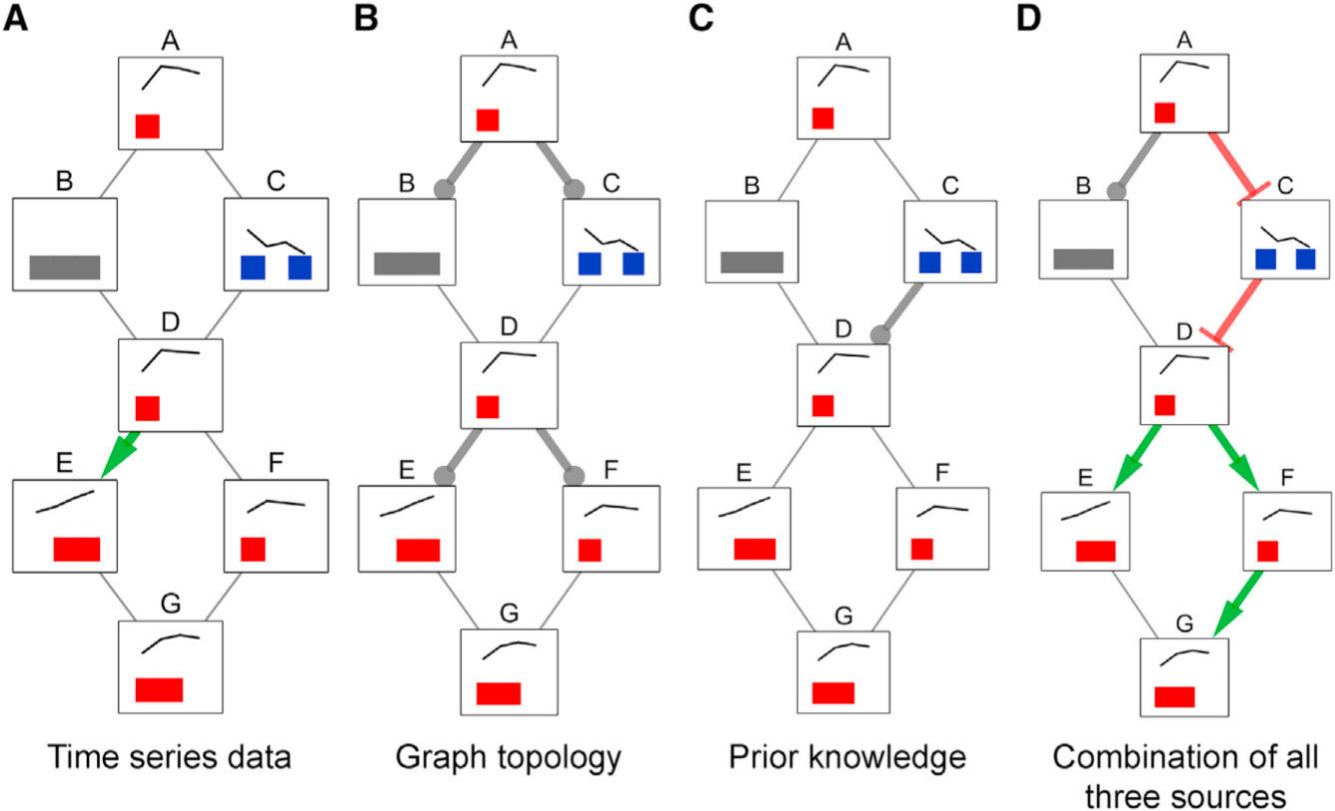
TPS Models for Individual versus Combined Data Sources Summary graphs obtained by aggregating (via graph union) all possible signed, directed tree models for different constraints obtained from time series data (A), graph topology (B), prior knowledge (in this example, kinase-substrate interaction directions) (C), and all three types of input at the same time (D). If an edge has a unique sign and direction in a summary graph (colored green and red for activations and inhibitions, respectively), this means there are no valid models that assign a different orientation or sign to that edge. Edges that can have any combination of sign and direction in different models are gray without an arrowhead. See also Figure S7.

The pathway structure illuminated by the phosphorylated proteins in our EGFR Flp-In cells differs considerably from the simple representations in pathway databases. Interpreting signaling data requires reconstructing models specific to the cells, stimuli, and environment being studied. TPS combines condition-specific information—time series phosphoproteomic data and the source of stimulation—with generic PPI networks and optional prior knowledge (Figure 5) to produce custom pathway representations. The predicted EGFR signaling network highlights alternative connections to classic EGFR pathway kinases and extends the pathway with interactions that are supported by prior knowledge in other contexts or kinase inhibition. Combining different constraints on pathway structure from PPI network topology and temporal information is computationally challenging, and we identify predictions that can be obtained only through joint reasoning with all available data (Figure 6).

### Contrasting TPS with Related Computational Approaches

TPS integrates information from PPI networks, phosphosite-specific time series phosphoproteomic data, and prior knowledge by introducing a powerful constraint-based approach. Existing classes of signaling pathway inference algorithms do not offer the same functionality as TPS. Methods that identify dependencies in phosphorylation levels (Hill et al., 2012; Zhang and Song, 2013) omit pathway members without observed phosphorylation changes. TPS does not require perturbations to reconstruct pathways (Ciaccio et al., 2015; Molinelli et al., 2013; Terfve et al., 2015). Participants in the HPN-DREAM network inference challenge (Hill et al., 2016) inferred signaling networks from time series data for tens of phosphoproteins, but the top methods either did not scale to our dataset (PropheticGranger; Carlin et al., 2017) or did not perform well (FunChisq; Zhang and Song, 2013). Other algorithms that integrate temporal information with PPI networks (Budak et al., 2015; Gitter and Bar-Joseph, 2013; Jain et al., 2016; Norman and Cicek, 2018; Patil et al., 2013) do not evaluate and summarize all pathway models that are supported by the network and phosphorylation timing constraints. This summarization strategy is what enables TPS to scale to solution spaces (Figure S6) that are substantially larger than those typically considered by declarative computational approaches (Chasman et al., 2014; Dunn et al., 2014; Guziolowski et al., 2013; Kӧksal et al., 2013; Moignard et al., 2015; Sharan and Karp, 2013). The Supplemental Experimental Procedures contain additional related software beyond these representative examples.

### Future Directions in Pathway Synthesis

TPS offers a powerful framework for combining multiple types of declarative constraints to generate condition-specific signaling pathways. The constraint-based approach could be extended to include additional types of data, such as perturbation data that link kinase inhibition or deletion to phosphorylation changes. Both temporal (Kanshin et al., 2015) and kinase perturbation (MacGilvray et al., 2018; Romanov et al., 2017) phosphoproteomic data are available for the yeast osmotic stress response. Modeling multiple related conditions (e.g., different ligand stimuli and inhibitor perturbations) could allow TPS to learn not only the signs of interactions but also the logic employed when multiple incoming signals influence a protein. TPS could also accommodate user-defined assumptions or heuristics about pathway properties, such as restrictions on pathway length. Such complex constraints cannot be readily included in approaches like DBN or TimeXNet.

For scalability, TPS requires hard logical constraints instead of probabilistic constraints (Hinton et al., 2006; Katoen et al., 2005). Discrete logic models for noisy biological data require modeling assumptions in order to balance model ambiguity and expressiveness. These tradeoffs and assumptions provide additional opportunities to modify and generalize the TPS model, for instance, a potential TPS extension to infer feedback in networks that is described in the Supplemental Experimental Procedures.

As proteomic technologies continue to improve in terms of depth of coverage (Sharma et al., 2014) and temporal resolution (Humphrey et al., 2015; Kanshin et al., 2015; Reddy et al., 2016), the need to systematically interpret these data will likewise grow. TPS enables reasoning with temporal phosphorylation changes and physical protein interactions to define what drives the vast protein modifications that are not represented by existing knowledge in pathway databases.

## EXPERIMENTAL PROCEDURES

### Temporal Pathway Synthesizer Algorithm Overview

TPS receives three types of input (Figure 1): a time series mass spectrometry phosphoproteomic analysis of a stimulus response; an undirected PPI subnetwork; and optional prior knowledge about interaction directions.

The undirected graph is obtained through a static analysis in which the significantly changing proteins are overlaid on a PPI network. A network algorithm recovers connections among the affected proteins, removing interactions that do not form critical connections between these proteins and nominating hidden proteins that do, even if they are not themselves phosphorylated. We recommend PCSF (Tuncbag et al., 2013) to select the PPI subnetwork but also successfully applied other methods (Gitter et al., 2011; Patil et al., 2013; Yeger-Lotem et al., 2009).

TPS transforms the input data into logical constraints that determine which pathway models can explain the observed phosphoproteomic data. Topological constraints stem from the filtered PPI network and require that phosphorylated proteins are connected to the source of stimulation, such as EGF, by a cascade of signaling events. These signaling events propagate along the edges of the filtered PPI network. Temporal constraints ensure that the order of the signaling events is consistent with the timing of the phosphorylation changes. If protein B is downstream of protein A on the pathway, B cannot be activated or inhibited before A. Prior knowledge constraints guarantee that if the direction or sign of an interaction is known in advance, the pathway may not contain the edge with the opposite direction or sign. Typically, many possible pathways meet all constraints, so TPS summarizes the entire collection of valid pathways and identifies interactions that are used with the same direction or sign across all models. A symbolic solver reasons with these logical constraints and produces the pathway summary without explicitly enumerating all possible pathway models.

To illustrate this process, consider a hypothetical signaling pathway that contains a receptor node A and six other downstream proteins that respond when A is stimulated (Figure 5). The first input is time series mass spectrometry data measuring the response to stimulating the receptor (node A), which quantifies phosphorylation activity for six proteins. Node B is absent from the phosphorylation data because it is post-translationally modified, but not phosphorylated, by A. The second input is an undirected protein-protein interaction graph. These are detected independently of the stimulation condition but filtered based on their presumed relevance to the responding proteins with an algorithm such as PCSF. By combining phosphorylation data with the PPI subnetwork, this topology can recover “hidden” components of the pathway that are not phosphorylated (node B). Finally, TPS accepts prior knowledge of directed kinase-substrate or phosphatase-substrate interactions, such as the edge C → D. Each of these inputs can be used individually to restrict the space of plausible pathway models. Reasoning about them jointly produces more unambiguous predictions than considering each resource separately.

To formulate temporal constraints, we transform the time series data into a set of discrete signaling events (activation or inhibition) for each node, taking an event-based view of the signaling process (Table 1). We determine time points for each node that correspond to statistically significant phosphorylation changes. These discrete events are then used to rule out network models that contain signed, directed paths that violate the temporal ordering of these events no matter which event is chosen for each node. For example, there can be no edge from E to D in any model because D is activated strictly earlier than E regardless of whether E is activated at 1 to 2 min or 2 to 5 min. Because the time series data measure the response to a specific stimulus, we also devise topological constraints that ensure all signaling activity originates from this source. In our example, this asserts that all edges in a solution network must be on a directed path that starts at node A. Finally, our third input, the set of directed interactions, requires that no model violates this prior knowledge by including an edge from D to C.

Figure 6 shows the pathway models that can be learned using each type of constraint alone and in combination. When we enforce only temporal constraints, which corresponds to reasoning locally with phosphorylation data for pairs of nodes to see whether one signaling event strictly precedes another, we obtain a single precise (signed and directed) prediction from D to E (Figure 6A). The topological constraints by themselves are sufficient to orient edges from the source A and from node D because D forms a bottleneck (Figure 6B). The prior knowledge constrains the direction of the edge from C to D, but its sign remains unknown (Figure 6C). Jointly enforcing all of these constraints has a nontrivial impact on the solution space (Figure 6D). For instance, we can infer that F must activate G. If the edge direction was reversed, F would be downstream of E, but the data show that activation of F precedes activation of E. The final model that includes all available data closely resembles the true pathway structure (Figure 5A). The edges incident to node B are ambiguous, and the interaction between E and G cannot be uniquely oriented, but all other interactions are recovered.

The summary for the combination of all constraints produces precise predictions that cannot be obtained by intersecting the summaries for the individual types of constraints. For instance, TPS infers that the relationship between F and G must be an activation from F to G because the sole way G can reach F in a tree rooted at A is through E, but F’s activation precedes E’s. This inference cannot be made by combining the models in panels A, B, and C. The simple example also highlights the differences in how the TPS constraint-based approach improves upon related methods based on correlation or the time point of maximum phosphorylation change (Kӧksal et al., 2018). See also Figure S7.

### TPS Pathway Synthesis

TPS takes the undirected network from PCSF and transforms it into a collection of signed, directed graphs that explain dynamic signaling events.

#### Discretization of Time Series Data

To find pathway models that agree with the phosphorylation dynamics, TPS first performs a discretization step that determines time intervals in which each protein may be differentially phosphorylated. The discrete set of activation and inhibition state changes is then used to rule out networks that violate the observed temporal behavior.

The transformation consists of finding time points for each profile where phosphorylation significantly differs from either the baseline (pre-stimulation) or the previous time point. In the baseline comparison, this time point is accepted only if it is not preceded by an earlier, larger change with respect to the baseline. If there is a hypothetical phosphorylation level at which the protein is activated and acts upon its downstream targets, a signaling event occurs only at the first time this threshold value is reached. This criterion does not apply when comparing to the phosphorylation level at the previous time point. TPS supports missing values in the time series data. The time points for which a phosphopeptide is missing data are assumed to be insignificant in the discretized data.

In our EGF study, we use Tukey’s HSD test to find significant differential phosphorylation. If comparing a time point to the baseline or the previous measurement produces a p value below a user-defined threshold, the time point is marked as a possible activation or inhibition event depending on whether the phosphorylation level increased or decreased relative to the earlier time point to which it was compared.

#### Modeling Assumptions

We assume at most one signaling event happens for every node across time points. Our logical solver can explore all possible activation and inhibition events for every node, but the data are often too ambiguous to allow multiple events per node given a single type of stimulation. In the absence of perturbation experiments that test the pathway behavior under different initial conditions, it is impossible to distinguish between different Boolean logic functions governing the behavior of each node and whether a node responds to one or multiple regulators. We therefore formalize pathway models as signed, directed trees, which provide a sufficient basis for explaining the dynamic system behavior under these assumptions.

### Translating Input into Constraints

TPS transforms each input into a set of constraints that declaratively specify valid signed, directed tree models that agree with the data (Supplemental Experimental Procedures). These constraints are expressed as Boolean formulas with linear integer arithmetic, ranging over symbolic variables that represent choices on edge signs and orientations as well as how the temporal data are interpreted. The constraints can then be solved by a satisfiability modulo theories (SMT) solver to find a network model that satisfies all constraints along with dynamic timing annotations for each interaction in the network.

Using constraints, we restrict the possible orientation and sign assignments to signed, directed tree networks rooted at the source node (e.g., EGF). Furthermore, constraints express how every tree model must agree with the time series data by establishing a correspondence between the order of nodes on tree paths and their temporal order of activity according to the time series data. Finally, we declaratively rule out models that contradict the prior knowledge of kinase-substrate interaction directions. These constraints define a very large space of candidate networks that agree with the data.

### Pathway Summaries

TPS can reason with large state spaces by summarizing all valid pathways instead of explicitly enumerating them. A summary network is the graph union of all signed, directed tree networks that satisfy the stated constraints (Figure 6). Timing annotations are summarized by computing the set of possible annotations for each node over all solutions. In the graph union, some edges have a unique direction and sign combination, which signifies that this was the only observed signed, directed edge between two given nodes across the solution space. However, this does not guarantee that the edge between the interacting proteins must be present in all valid pathway models. Ambiguous directions or signs in the summary means that there are valid models with different direction or sign assignments.

We compute the summary graph by performing a linear number of SMT solver queries in terms of the size of the input graph. Each queries whether at least one signed, directed model contains a specific signed, directed edge. Because individual queries are computationally cheap, we can summarize the entire solution space without enumerating all models, which is typically intractable. The summary graph over-approximates the solution space. It is not possible to recover the exact set of valid models from the summary, only a superset of the models (Figure S7). This tradeoff must be made in order to analyze such a large state space.

### Using Solvers for Synthesis

TPS uses the Z3 theorem prover (De Moura and Bjørner, 2008) via the ScalaZ3 interface (Kӧksal et al., 2011) to solve the constraints it generates. It also provides a custom data flow solver specifically for computing pathway summaries. The custom solver and the symbolic solver produce identical pathway summaries. However, the custom solver is much more scalable because it is specifically designed to address our synthesis task and can handle networks containing more than a hundred thousand edges and phosphosites (Figure S6; Kӧksal et al., 2018).

### Cell Culture and Mass Spectrometry

We stimulated EGFR Flp-In cells (Gordus et al., 2009) with 23.6 nM EGF (Peprotech) for 0, 2, 4, 8, 16, 32, 64, or 128 min. Cells were lysed and proteins were extracted, denatured, alkylated, and trypsin digested. Following digestion, the tryptic peptides were either lyophilized, stored for future use, or directly processed for mass spectrometry analysis. To quantify dynamic changes in protein phosphorylation, all peptides were isobarically labeled (Ross et al., 2004), enriched using phosphotyrosine-specific antibodies and/ or immobilized metal affinity chromatography (IMAC) (Ficarro et al., 2002), and analyzed on a Thermo Fisher Velos Orbitrap mass spectrometer (Ficarro et al., 2011; Wolf-Yadlin et al., 2006) in data-dependent acquisition mode. We determined peptide sequences using Comet (Eng et al., 2013; Data S5) and quantified the iTRAQ signals with Libra (Deutsch et al., 2010). Across three biological replicates, we quantified 5,442 unique peptides in at least one replicate and 1,068 peptides in all replicates and used Tukey’s honest significant difference for statistical testing (Data S1). See the Supplemental Experimental Procedures for details and data processing. Also see our p value sensitivity analysis (Kӧksal et al., 2018).

### Quantitative Western Blotting

We used 25 nM Dasatinib (no. S1021), 400 nM SCH772984 (no. S7101), and 800 nM MK-2206 (no. S1078; all Selleckchem) for kinase inhibition and antibodies pY221-CRK (no. 3491; Crk-II isoform), pY10-ATP1A1 (no. 3060), and pS142/143-Zyxin (no. 8467; all Cell Signaling Technology) for western blotting (Supplemental Experimental Procedures). We normalized loading with β-actin (no. 3700) and imaged blots with an Odyssey Infrared Imaging System (Li-COR Biosciences).

## PCSF

We used the Omics Integrator PCSF implementation (Tuncbag et al., 2016) with msgsteiner (Bailly-Bechet et al., 2011) to recover the most relevant PPIs connecting the phosphorylated proteins. The Supplemental Experimental Procedures describe how we selected parameters, ran PCSF multiple times to identify parallel connections between proteins, generated prizes from the phosphoproteomic data, and created a weighted interaction network from iRefIndex (Razick et al., 2008) and PhosphoSitePlus (Hornbeck et al., 2015).

## DATA AND SOFTWARE AVAILABILITY

The accession number for the raw mass spectrometry proteomics data reported in this paper is PRIDE: PXD006697.

The processed data are in Data S1. TPS (https://github.com/koksal/tps) and our visualization tool for TPS output (https://github.com/koksal/tpv) are available as MIT-licensed open source software. An archival copy of TPS version 2.2, including instructions for running the software, example data, and scripts for linking PCSF and TPS, is available at https://doi.org/10.5281/zenodo.1215177.

## Supplementary Material

1

2

3

4

5

6

7

## Figures and Tables

**Table 1.** Signaling Timing in the Artificial Example

Node		Plausible Temporal Signaling Events
A		activated 0 to 1 min
B		activated or inhibited at any time
C		inhibited 0 to 1 min or 2 to 5 min
D		activated 0 to 1 min
E		activated 1 to 2 min or 2 to 5 min
F		activated 0 to 1 min
G		activated 0 to 1 min or 1 to 2 min

Plausible signaling events inferred for each node through a statistical analysis of the time series phosphorylation data. Although B is modified in the 0 to 1 min interval, this is not observed in the phosphoproteomic input data.

## Highlights

- TPS predicts signaling pathways that explain large-scale phosphorylation changes
- TPS models dynamics of individual phosphosites in a protein network context
- Existing algorithms do not recover the temporal relationships detected by TPS
