# Supplementary material for: Synthesizing Signaling Pathways from Temporal Phosphoproteomic Data
Source: Cell Rep. Author manuscript; Available in PMC 2018 Dec 16. (PMC6295338; doi:10.1016/j.celrep.2018.08.085)
Supplement: 1 [file NIHMS1508952-supplement-1.pdf]

Cell Reports, Volume *24*

Supplemental Information

Synthesizing Signaling Pathways from Temporal Phosphoproteomic Data

Ali Sinan Köksal, Kirsten Beck, Dylan R. Cronin, Aaron McKenna, Nathan D. Camp, Saurabh Srivastava, Matthew E. MacGilvray, Rastislav Bodík, Alejandro Wolf-Yadlin, Ernest Fraenkel, Jasmin Fisher, and Anthony Gitter

Supplemental Figures

A)

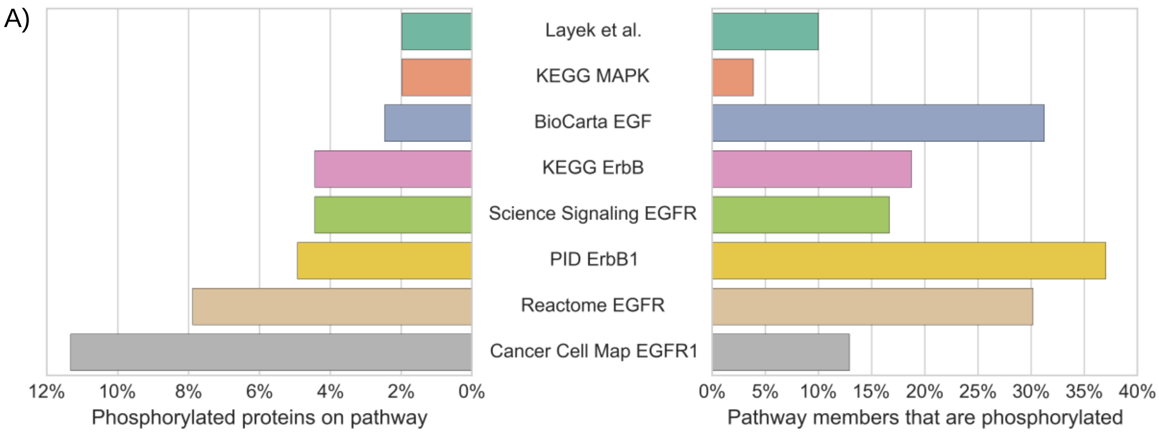

B)

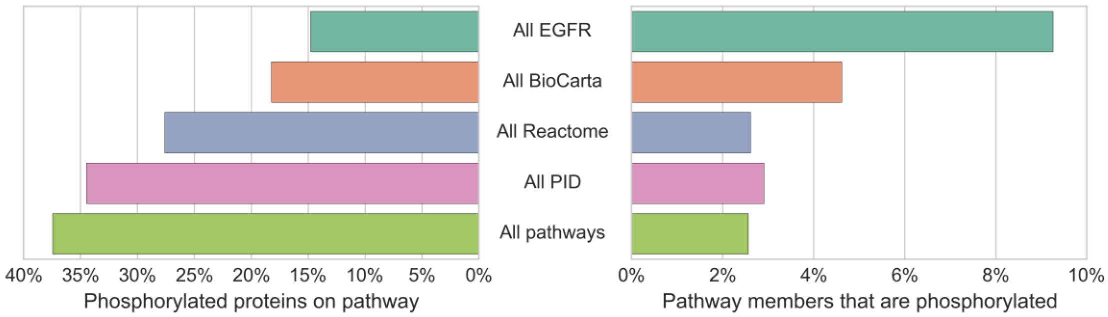

Figure S1. Related to Figure 2. A) Over 95% of the significantly differentially phosphorylated proteins in response to EGF stimulation are not included in six of the eight reference pathways. Conversely, the majority of proteins in the reference pathways are not significantly differentially phosphorylated. The Cancer Cell Map pathway achieves the best phosphorylation coverage, but it is still only 11%. B) Most significantly differentially phosphorylated proteins do not appear in any pathway diagram, even when extending the analysis to non-EGFR-specific pathways. All EGFR is the union of the EGFR-related pathways in panel A. All BioCarta, Reactome, and PID reflect the union of all pathways in the respective database. All pathways is the union of all of the above pathways.

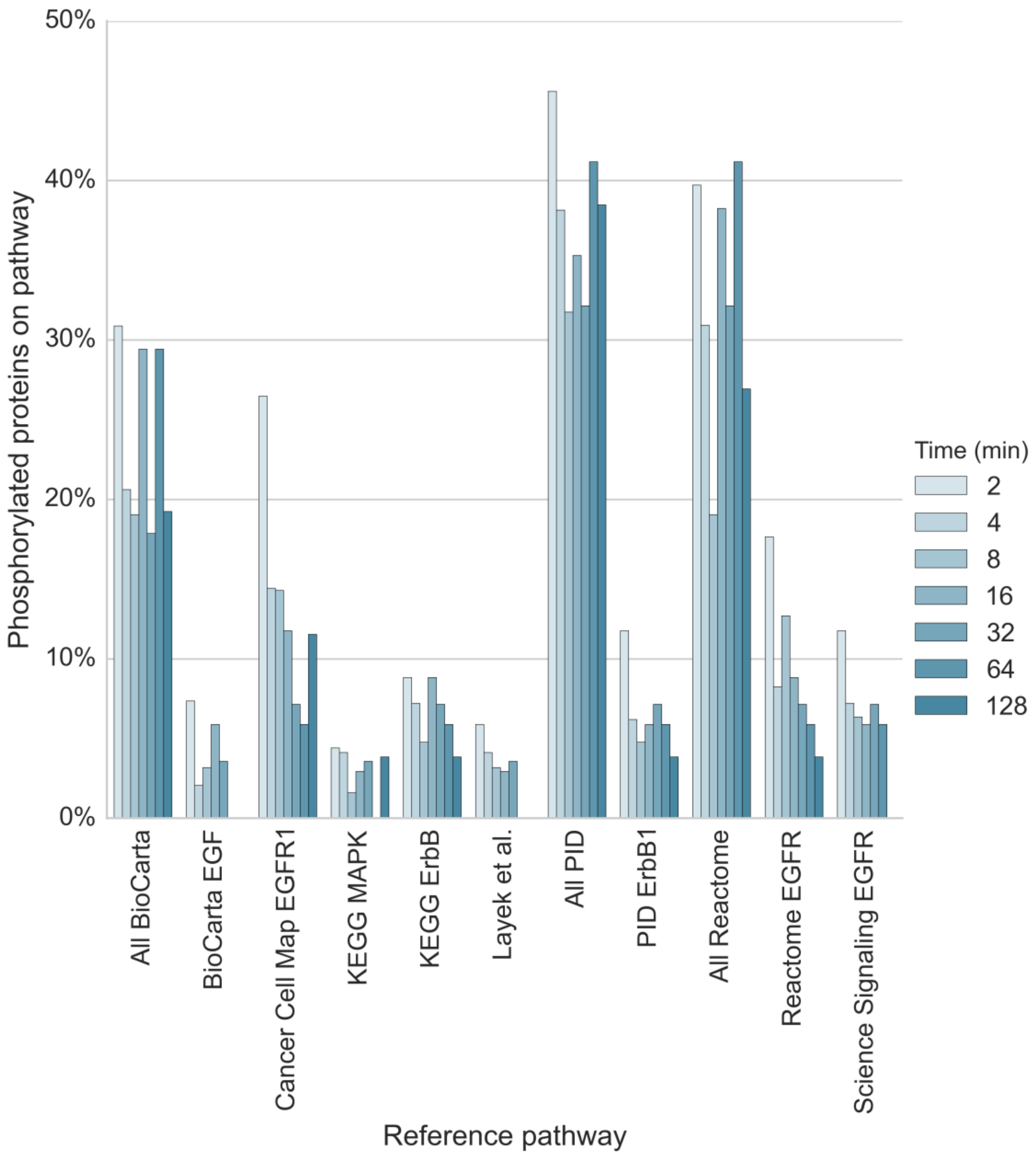

Figure S2. Related to Figure 2. The percentage of proteins that are significantly differentially phosphorylated and overlap with the reference pathways varies by time point. The percentage is with respect to the number of proteins that are significantly differentially phosphorylated at the given time point: 68 (2 min), 97, 63, 34, 28, 17, and 26 proteins (128 min).

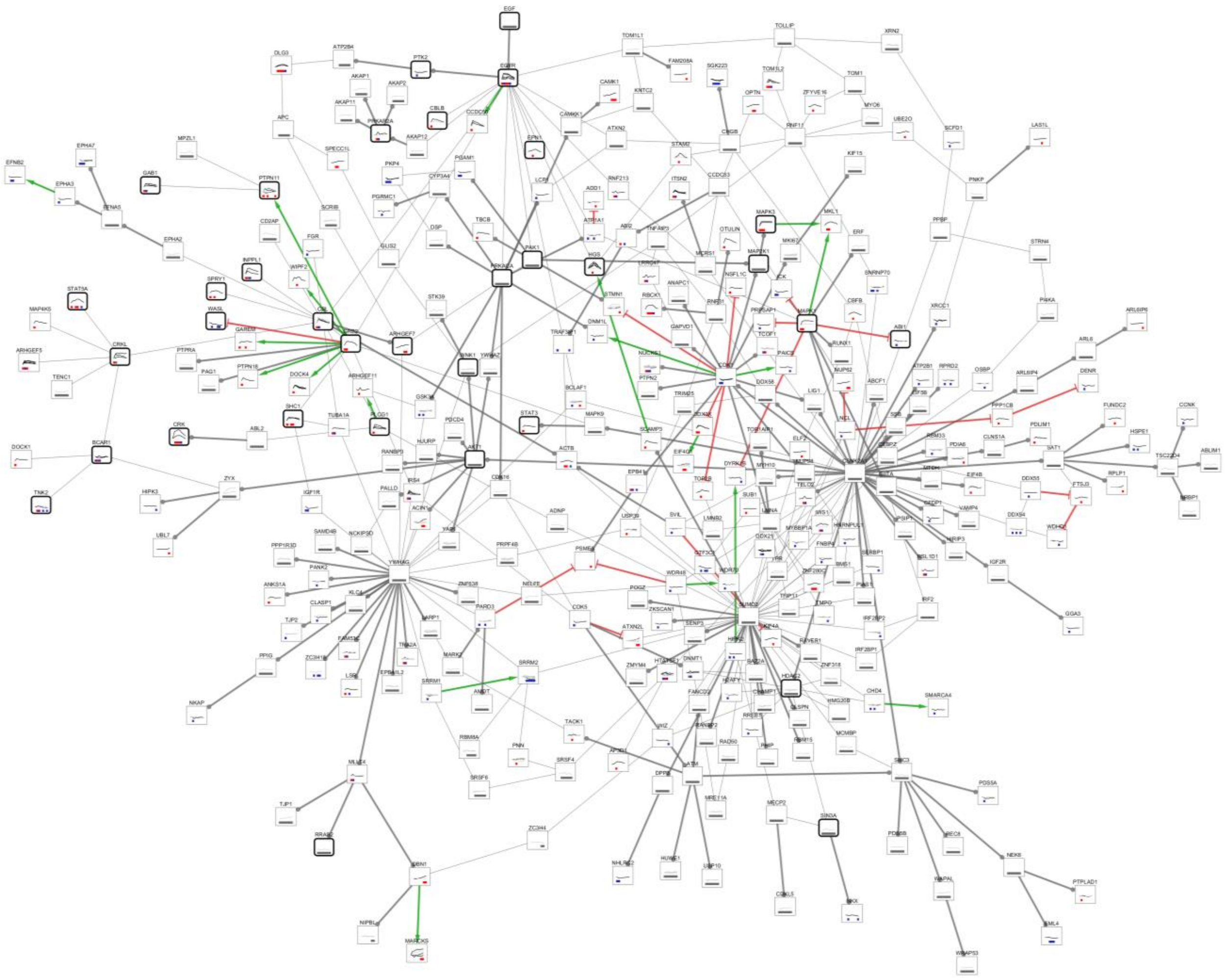

Figure S3. Related to Figure 3. The full TPS EGF response pathway summary (Supplemental File 3). Node and edge visualizations are as in Figure 3.

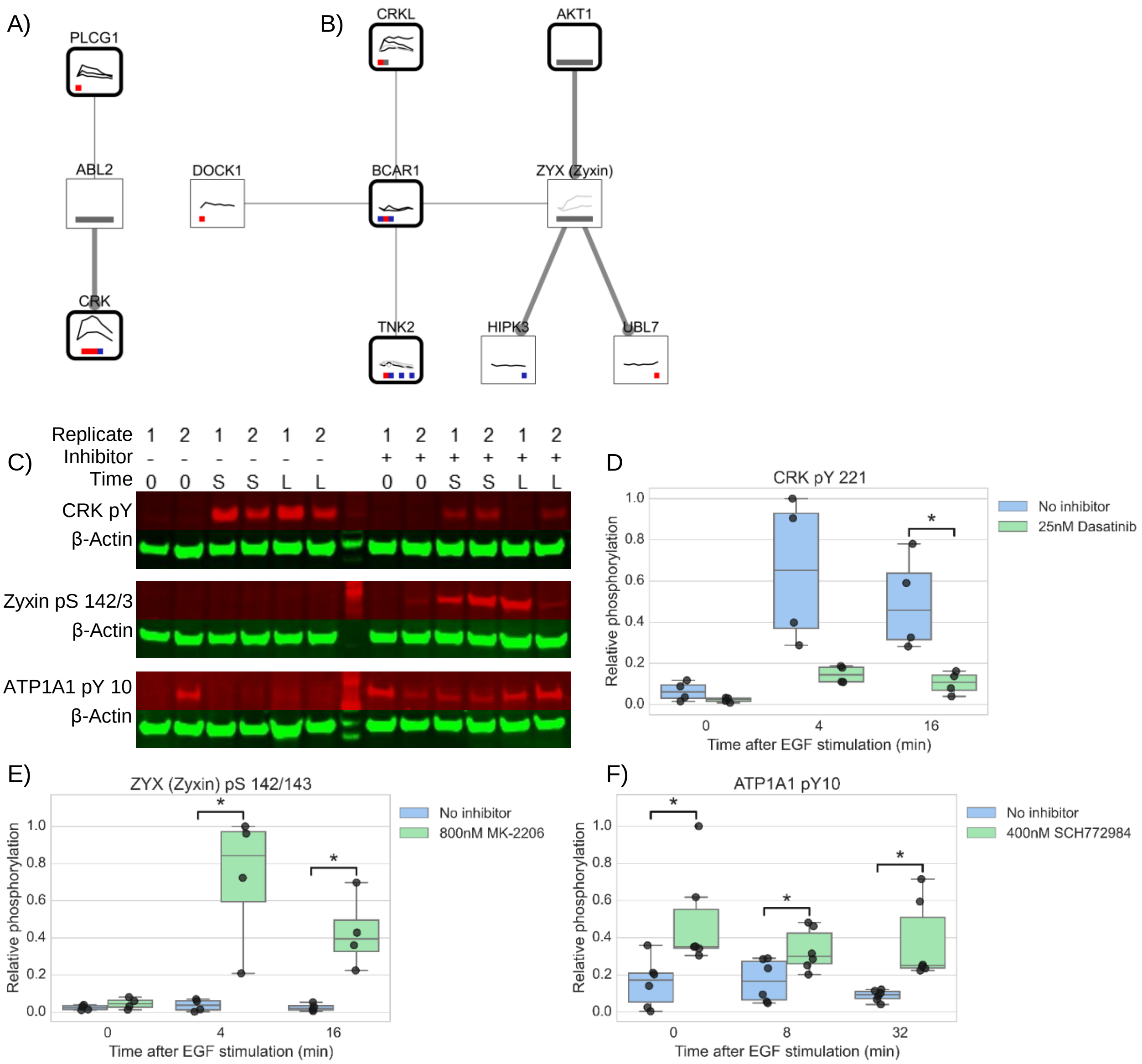

Figure S4. Related to Figure 3. Inhibiting predicted pathway edges. A) The predicted pathway context of the ABL2 to CRK interaction. B) The pathway context of the AKT1 to Zyxin interaction, which includes BCAR1, Zyxin, and all of their neighbors. The pathway context of the MAPK1 and ATP1A1 interaction is shown in Figure 3C. C) Western blots for CRK pY 221, Zyxin pS 142/143 and ATP1A1 pY 10 in the presence and absence of small molecule inhibitors targeting their parent node (Dasatinib/ABL2, MK-2206/AKT1, and SCH772984/MAPK1, respectively). The red channel displays detection of the specific phosphorylation sites. The green channel displays detection of β-Actin (a loading control used for normalization of each specific phospho signal). Two biological replicates are shown in each Western blot. Time "0" indicates no EGF stimulation; "S" is short EGF stimulation (four or eight min), and "L" is long EGF stimulation (sixteen or thirty-two min). Absence or presence of inhibitor is shown by "-" and "+", respectively. D) Quantification of CRK pY 221 phosphorylation (four replicates). Phosphorylation levels are relative to the maximum phosphorylation across all conditions and replicates. An asterisk denotes $p < 0.05$ (two-sided, unpaired, unequal variances t-test). Whiskers show 1.5 times the interquartile range. E) Quantification of Zyxin pS 142/143 phosphorylation (four replicates). F) Quantification of ATP1A1 pY 10 phosphorylation (six replicates).

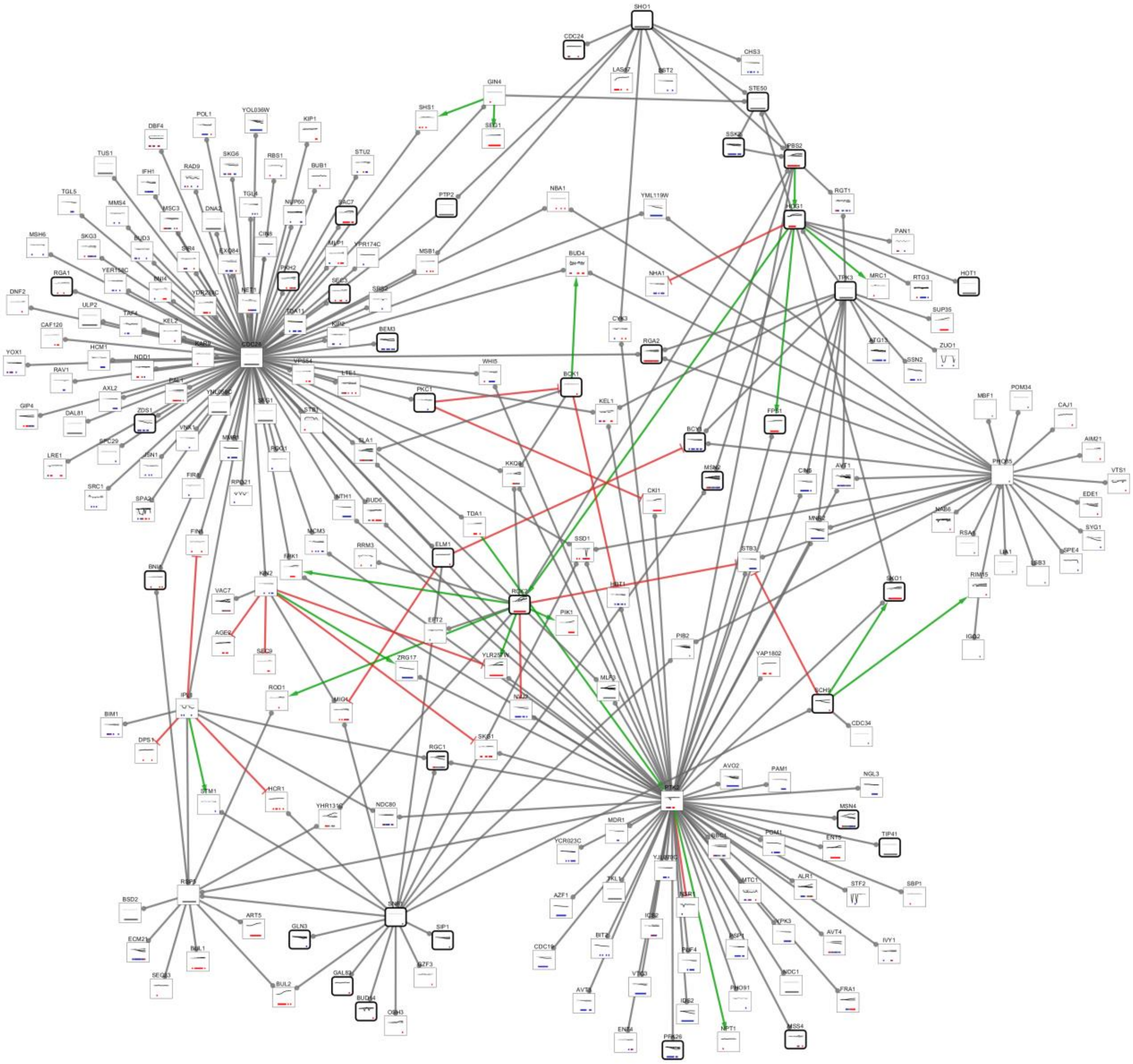

Figure S5. Related to Figure 4. The full TPS pathway summary for the yeast osmotic stress response (Supplemental File 3). Node and edge visualizations are as in Figure 3.

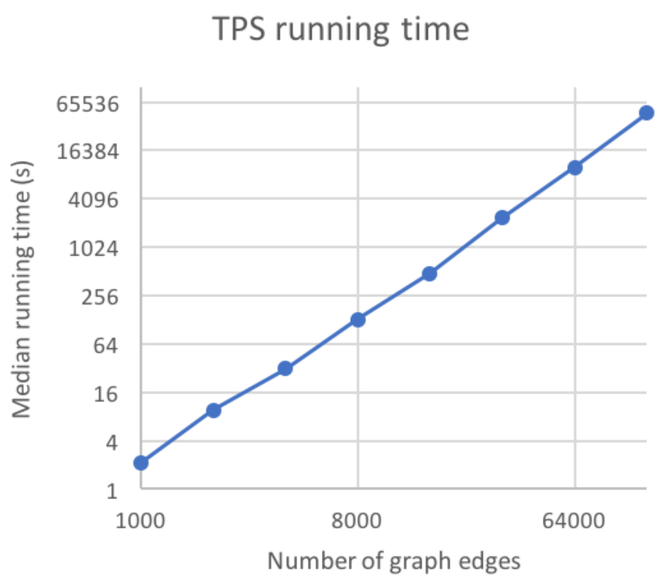

Figure S6. Related to Experimental Procedures. The median running time of TPS over three replicate runs against the number of input graph edges in log-log scale.

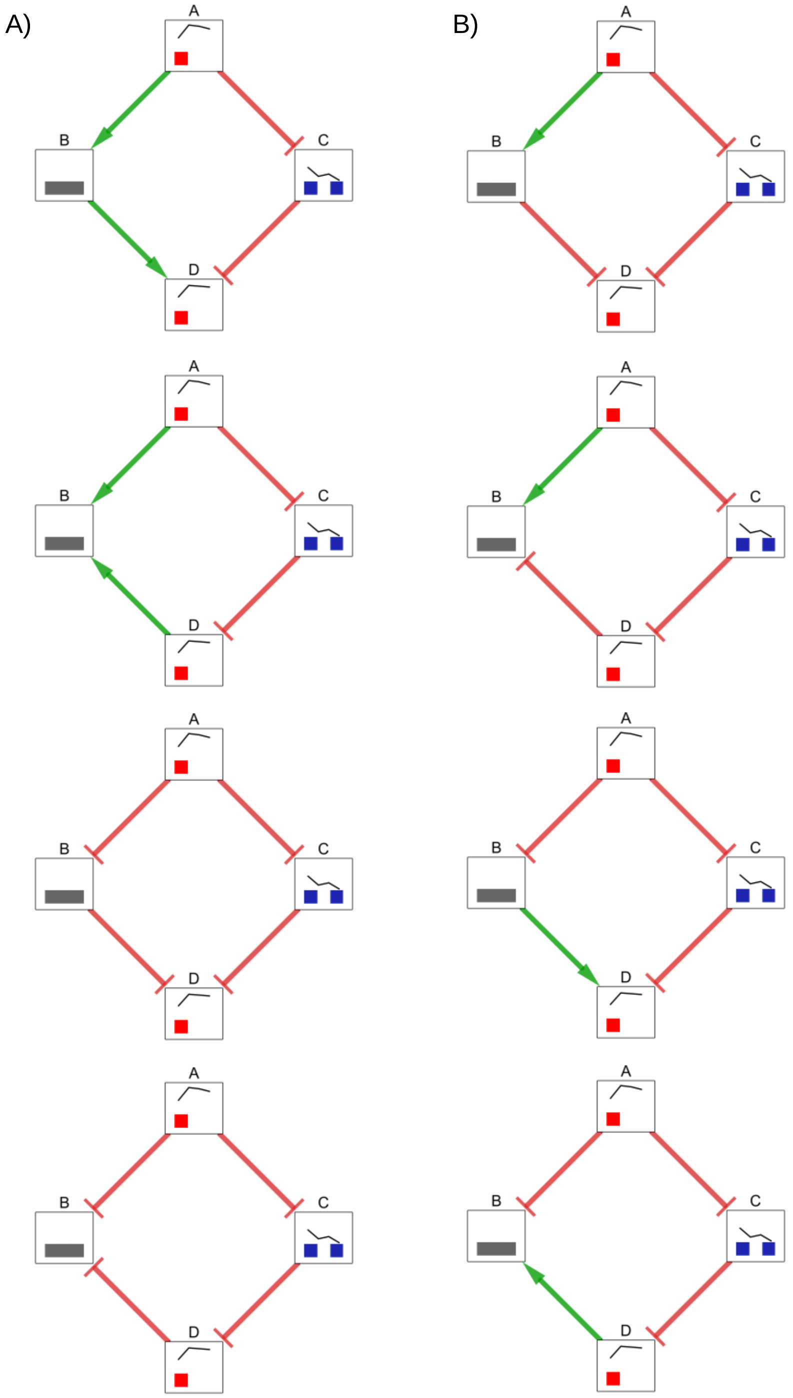

Figure S7. Related to Figure 6. An illustration of how the pathway summary graph (Figure 6D) is a generalization of the individual pathway models and can also include invalid models. Not all combinations of direction and sign assignments to the ambiguous edges yield pathways that satisfy all constraints. Here we depict only edges among nodes A, B, C, and D. For simplicity we ignore the models in which one of these edges are absent, even though these models are also included in the summarization. In some cases, B has no valid temporal activity, which we denote with the ambiguous temporal annotation (gray). A) Pathway models in which the signs of edges A-B and B-D are consistent with the constraint that A and D are both activated. B) Pathway models in which these edges have opposing signs and are included in the summary even though they violate a constraint.

Supplemental Tables

Table S1. Related to Figure 3. All predicted interactions tested experimentally. Outcomes are presented in Figure S4 and detailed in (Köksal et al., 2018). MAPK1->MKL1 was tested with two inhibitors. DRP1 is a synonym for DNM1L.

**Inhibit**	**Detect**	**Compound**	**Concentration**	**Antibody**	**Results**
ABL2	CRK	Dasatinib	25 nM	#3491 Phospho-CrkII (Tyr221)	Figure S4
AKT1	RANBP3	MK-2206	800 nM	#9380 Phospho-RanBP3 (Ser58)	No change detected
AKT1	YAP1	MK-2206	800 nM	#13008 Phospho-YAP (Ser127)	No band detected
AKT1	ZYX	MK-2206	800 nM	#8467 Phospho-Zyxin (Ser142/143)	Figure S4
CDK1	MAP2K1	RO-3306	250 nM	#9154 Phospho-MEK1/2 (Ser217/221)	No change detected
EGFR	CCDC50	Erlotinib	200 nM	sc-79367 YMER Antibody (I-12)	No band detected
MAPK1	ATP1A1	SCH772984	400 nM	#3060 Phospho-Na,K-ATPase α1 (Tyr10)	Figure S4
MAPK1	MKL1	AZD6244	140 nM	Gift from Dr. Ron Prywes MKL1 (Ser454)	No band detected
MAPK1	MKL1	SCH772984	400 nM	Gift from Dr. Ron Prywes MKL1 (Ser454)	No band detected
PRKACA	DNM1L	H-89	480 nM	#4867 Phospho-DRP1 (Ser637)	No band detected
PRKACA	GSK3A	H-89	480 nM	#8452 Phospho-GSK-3α (Ser21)	No band detected

Table S2. Related to Experimental Procedures. The time point-specific log_2 fold changes used to determine significance in the yeast osmotic stress phosphoproteomic dataset.

**Time (s)**	**Threshold for significant dephosphorylation**	**Threshold for significant phosphorylation**
5	-0.4006441	0.3706107
10	-0.3740002	0.3337672
15	-0.3561254	0.3403914
20	-0.3685697	0.3871419
25	-0.4068662	0.3568199
30	-0.4895241	0.4388251
35	-0.5204797	0.4964112
40	-0.4190634	0.4029404
45	-0.3548886	0.3397075
50	-0.4688706	0.4645637
55	-0.4468361	0.3969267
60	-0.4861253	0.4287856

Supplemental Experimental Procedures

Cell culture, stimulation, and generation of peptides

Flp-In 293 cells expressing EGFR were described previously (Gordus et al., 2009) and have been used in our previous studies of receptor tyrosine kinase signaling (Wagner et al., 2013). These isogenic cells do not express EGFR heterodimerization receptor partners, and receptor quantities are uniform across cells (~100,000 EGFR/cell). We selected EGFR Flp-In cells for this study because they are easy to manipulate and provide full control of the input signal. We know the number of receptors per cell and thus the ligand concentration necessary to achieve different levels of saturation. Most importantly, because EGFR Flp-In cells are homogeneous with respect to EGFR expression, this system ensures high reproducibility between replicates and minimizes effects of heterogeneous receptor expression between different samples and time points. Although their signaling response may differ from *in vivo* responses in human tissues, this system ensures the phosphorylation changes are EGFR-specific.

Cells were grown using standard cell culture procedures in Dulbecco's modified Eagle's medium supplemented with 10% (v/v) fetal bovine serum, 2 mM glutamine, 100 U/ml penicillin, 100 µg/ml streptomycin, and 150 µg/ml hygromycin B. For the activation of the EGF receptor, the cells were grown in plates to approximately 70% confluency, then washed once with phosphate-buffered saline (PBS) and incubated for 16 hours in serum-free medium. Subsequently cells were stimulated with 23.6 nM EGF (Peprotech) for 0, 2, 4, 8, 16, 32, 64, or 128 minutes. Untreated plates were used for the 0 min time point. After EGF stimulation, cells were lysed on ice with 3 ml of 8 M urea supplemented with 1 mM Na_3VO_4. A 10 µl aliquot was taken from each sample to perform the micro bicinchoninic acid protein concentration assay (Pierce) according to the manufacturer's protocol. Cell lysates were reduced with 10 mM DTT for 1 hr at 56 °C, alkylated with 55 mM iodoacetamide for 45 min at room temperature, and diluted to 12 ml with 100 mM ammonium acetate; pH 8.9. 40 µg trypsin (Promega) was added to each sample (~200:1 substrate to trypsin ratio) and the lysates were digested overnight at room temperature. The whole cell digest solutions were acidified to pH 3 with acetic acid (HOAc) and loaded onto C18 Sep-Pak Plus C18 Cartridges (Waters). The peptides were desalted (10 mL 0.1% trifluoroacetic acid (TFA)) and eluted with 10 mL of a solution comprised of 40% acetonitrile (MeCN) with 0.1% TFA. Each sample was divided into ten aliquots and lyophilized overnight to dryness for storage at –80°C.

The peptides were then labeled using 8-plex iTRAQ reagents (Ab Sciex) according to the manufacturer's instructions. 200 µg of lyophilized peptides were resuspended in 30 µl of dissolution buffer, and the corresponding iTRAQ reagent dissolved in 70 µl isopropanol was added. The mixtures were incubated at room temperature for 1 hr and concentrated to ~30 µl. Samples labeled with eight different isotopic iTRAQ reagents were combined and dried to completion. The sample was then rehydrated in 500 µl (0.1% HOAc) and desalted using a Sep-Pack Vac C18 column (Waters). The peptides were eluted with 80% acetonitrile, 0.1% HOAc. The eluate was evaporated to 100 µl in the SpeedVac and lyophilized.

Phosphopeptide enrichment, mass spectrometry, and identification

Peptides containing phosphotyrosines were enriched using immunoprecipitation (IP). 12 µg of each of the antibodies P-Tyr-1000 (Cell Signaling Technologies), 4G10 (Millipore), and PT-66 (Sigma-Aldrich) were bound to 20 µl of packed protein G Plus agarose beads (Calbiochem) in IP buffer (100 mM Tris-HCl pH 7.4, 0.3% NP-40). The lyophilized peptides were rehydrated with IP buffer, and the pH of the solution was adjusted to 7.4 using 100 mM Tris-HCL ph 8.5. The peptide sample was added to the beads and incubated for 4 hours. The supernatant was then removed and saved for the next step. The beads were washed extensively using IP buffer, 100 mM Tris-HCl and H_2O. The bound peptides were eluted using 50 µl 15% acetonitrile/0.1% TFA.

Serine and threonine (and remaining tyrosine) phosphorylated peptides were enriched from the IP supernatant using immobilized metal affinity chromatography. Briefly, protein concentration was adjusted to 1 mg/ml protein using wash buffer (80% MeCN/0.1%TFA). 100 µl of Ni-depleted Ni-NTA Superflow beads (Qiagen) were activated with 100 mM FeCl3. The supernatant was loaded onto the beads and incubated for 1hr. After washing the beads three times with wash buffer, the bound peptides were eluted twice with 1.4% ammonium hydroxide. The eluates were combined and evaporated in a SpeedVac to 5-10 µl. The sample was then reconstituted to a total volume of 20 µl in 20 mM ammonium formate pH 9.8.

All mass spectrometry experiments were performed on a Thermo Fisher Velos Orbitrap mass spectrometer (Ficarro et al., 2011; Wolf-Yadlin et al., 2006; Zhang et al., 2005) equipped with a nanospray ionsource coupled to a

nanoACQUITY Ultra Performance LC system (Waters) equipped with two binary pumps. Samples were separated using either 1D (IP eluate) or 2D (IMAC eluate) chromatography. For the 1D separation, the sample was loaded onto a 5 cm self-packed (Reliasil, 5 µm C18, Orochem) pre-column (inner diameter 150 µm) connected to a 20-cm self-packed (ReproSil, 3 µm C18, Dr. Maisch) analytical capillary column (inner diameter 50 µm) with an integrated electrospray tip (~1 µm orifice). Peptides were separated using a 115-minute gradient with solvents A (H_2O/formic acid (FA), 99.9:1 (v/v)) and B (MeCN/FA, 99.9:1 (v/v)) as follows: 1 min at 2% B, 84 min from 98 to 40% B, 5 min at 40% B, 20 min at 20% B, and 14 min at 2% B. For the 2D reverse phase chromatography, the sample was first loaded onto a 5 cm self-packed Xbridge column (Waters, inner diameter 150 µm) and eluted with a 7-step gradient of 1, 3, 6, 9, 13, 25, and 44% B with solvents A (H_2O/20 mM ammonium formate pH 9.8) and B (MeCN/20 mM ammonium formate, pH 9.8). The eluted sample was directly loaded onto a 5 cm self-packed precolumn (Reliasil, 5 µm C18, Orochem), which was connected to a 20 cm self-packed analytical column (ReproSil, 3 µm C18, Dr. Maisch). The peptides were eluted with the same gradient as described above in the second dimension.

Eluted peptides were directly analyzed using the Velos-Orbitrap mass spectrometer operated in data-dependent acquisition (DDA) mode to automatically switch between MS and MS/MS acquisitions. The Top 10 method was used, in which full-scan MS (from m/z 350–2000) was acquired in the Orbitrap analyzer at 120,000 resolution, followed by high-energy, collision-induced dissociation (HCD) MS/MS analysis (from m/z 100–1700) of the top 10 most intense precursor ions with a charge state >2. The HCD MS/MS scans were acquired using the Orbitrap analyzer at 15,000 resolution at a normalized collision energy of 45%, with the ion selection threshold set to 10,000 counts. Precursor ion isolation width of 2 m/z was used for the MS/MS scans, and the maximum allowed ion accumulation times were set to 500 ms for full (MS) scans and 250 ms for HCD (MS/MS). The standard mass spectrometer tune method settings were as follows: Spray Voltage, 2.2 kV; no sheath and auxiliary gas flow; heated capillary temperature, 325 C; Automatic Gain Control (AGC) enabled. All samples were analyzed by LC-MS/MS in biological triplicates.

MS/MS data files were searched against the human protein database using Comet (Eng et al., 2013). Variable (phosphorylation of serine, threonine, or tyrosine, 79.966331 Da, methionine oxidation, 15.9949 Da) and static (carbamidomethylation of cysteine, 57.02 Da and the iTRAQ modification of 304.205360 Da to peptide N-terminus and lysine side-chains) modifications were used for the search (Supplemental File 5). We applied a 1% false discovery rate threshold at the peptide level. Quantitation of the iTRAQ signals was performed using Libra (Deutsch et al., 2010). We deposited the raw mass spectrometry data to the ProteomeXchange Consortium via the PRIDE (Vizcaíno et al., 2016) partner repository.

Mass spectrometry data analysis and temporal phosphorylation significance

We obtained two technical replicates for each of the three biological replicates, quantifying IP and IMAC eluate separately (Supplemental File 1). To decrease noise, we applied an iTRAQ channel intensity cutoff of 5,000 for IP and 10,000 for IMAC (arbitrary units), removing peptides that did not exceed the threshold in at least one channel. We median centered the data within each channel by dividing the signal by the channel median. For each biological replicate, we concatenated the technical replicates and the IP and IMAC intensities. We removed non-phosphopeptides and aggregated the signal for phosphopeptides with the same sequence (regardless of post-translational modifications) by summing the normalized values (Supplemental File 1). Across the three biological replicates, we quantified 5,442 unique peptides in at least one replicate and 1,068 peptides in all replicates. We focused on these 1,068 peptides for the computational modeling because the repeated observations indicate more reliable quantification and let us assess the significance of phosphorylation changes (Supplemental File 1).

After filtering peptides missing data in one or more biological replicates, we median centered the data again and performed Tukey's Honest Significant Difference (HSD) test (Yandell, 1997) for each peptide (Supplemental File 1). Tukey's HSD test reports the significance in the difference of the mean peptide intensities for all pairs of time points, correcting for the multiple comparisons within each peptide's time course. It is conceptually similar to performing t-tests for all pairs of time points, but it conducts all tests simultaneously and adjusts for multiple testing appropriately. Although we have temporal data, we selected Tukey's HSD test instead of a temporal statistical test like EDGE (Leek et al., 2006) because TPS requires a test statistic for individual time points, not the entire time course.

Tukey's HSD test is already quite conservative (Yandell, 1997). However, it does not correct for the multiple comparisons from testing multiple peptides. As a precaution, we computed q-values (Storey and Tibshirani, 2003)

from the distribution of p-values for all pairs of time points and all peptides. Our p-value threshold $p < 0.01$ is equivalent to a q-value threshold $q < 0.188$, which we consider to be an acceptable false discovery rate for this application. In addition, the predicted network is reasonably robust to the choice of p-value threshold (Köksal et al., 2018).

Our temporal analysis was based on two types of significant changes in phosphorylation: changes relative to the baseline (0 minute) time point and changes relative to the previous time point. For each time point, we took the smaller of these two p-values (comparison to baseline or previous) from Tukey's HSD test as the significance of that time point and produced an aggregate p-value for the peptide as the minimum such p-value over all time points. The aggregate p-value serves as a proxy of whether the peptide significantly changes in response to stimulation at any time point, and 263 peptides responded significantly ($p < 0.01$). In addition, we calculated a summary phosphorylation profile for each peptide by taking the median value at each time point. In order to visualize the $\log_2$ fold changes (as in Figure 2B), we used the only non-zero replicate for the three peptides where the median value was zero. This substitution was only for visualization; TPS does not operate on fold changes. We mapped peptides to UniProt identifiers, using the best-matching protein identifier for each peptide.

We cannot recover a kinetic model of kinase (or phosphatase) activity from the mass spectrometry data, but our temporal constraints assume that there is an unknown, hidden phosphorylation level at which a kinase (phosphatase) begins to substantially (de)phosphorylate its target. This phosphorylation level may vary by protein, so we do not set a threshold on the phosphorylation fold change. Furthermore, the protein may continue to increase (or decrease) in phosphorylation past this critical level even after it has begun to affect the substrate, so we do not assume that the time of peak phosphorylation is the only relevant time.

Quantitative Western blotting
The following kinase inhibitors were used at the following concentrations: 25 nM Dasatinib (#S1021), 400 nM SCH772984 (#S7101), and 800 nM MK-2206 (#S1078, all Selleckchem). The Flp-In 293 EGFR cells were serum-starved for 16 hours in growth medium without FBS. Then, if indicated, the kinase inhibitors were added and incubated with the cells for 60 min. Only DMSO was added to control cells. After stimulation with 23.6 nM EGF for the indicated times, the cells were lysed using RIPA buffer (25mM Tris-HCl pH 7.6, 150mM NaCl, 1% NP-40, 1% sodium deoxycholate, 0.1% SDS) with 1 mM sodium orthovanadate. Cells without EGF stimulation were used as controls (0 min time point). After incubation on ice for 15 minutes, the lysates were centrifuged at 21000 g for 10 min, and the protein concentration of the supernatants was determined. Protein amounts were adjusted to 30–50 ug protein/well, and protein phosphorylation was assessed by SDS separation and Western blotting. The membranes were probed with the following antibodies at a dilution of 1:1000: pY221-CRK (#3491, Crk-II isoform), pY10-ATP1A1 (#3060), and pS142/143-Zyxin (#8467, all Cell Signaling Technologies). β-actin (#3700) was used to normalize loading across the gel. Fluorescently labeled secondary antibodies were added according to the manufacturer's instructions at 1:5000 (Goat anti rabbit IRDye 680 and Goat anti mouse IRDye 800, Li-COR Biosciences). Blots were imaged using an Odyssey Infrared Imaging System (Li-COR Biosciences). Quantification of the phosphorylated proteins was performed with the Odyssey analysis software.

Translating the input into constraints
A symbolic signed, directed graph that assigns a sign and direction to each edge in (a subgraph of) the undirected input graph can be represented by maintaining four Boolean variables per edge, one for each sign-orientation combination. The truth value of a variable denotes whether there is an edge with the corresponding sign and orientation in the solution network. To find a tree network model rooted at the stimulated source node, we need to constrain these truth values. First, we assert that at most one of these four variables can be true. The case where all four variables are false corresponds to the undirected edge being excluded from the solution network. Then, we assert that there are no cycles in the solution graph. To implement the acyclicity constraint, we maintain one integer-valued variable per node and assert that the integer values along all directed paths must monotonically increase. Finally, we assert that if a non-source node has an outgoing edge, it must have an incoming edge as well. This prevents modeling spurious phosphorylation changes that are not caused by the source's stimulation. These constraints together guarantee that we obtain a tree network model in which all edges are on a directed path originating at the source node.

Example: The edge *(A, B)* in the undirected graph shown in Figure 5C, like all other edges in the network, is translated into four Boolean variables, *activation-A-B, inhibition-A-B, activation-B-A, inhibition-B-A*. We ensure at

most one variable is true by asserting that if one of the variables is true, the rest must be false. For instance, we assert:

activation-A-B ==> !inhibition-A-B && !activation-B-A && !inhibition-B-A

which means that if *activation-A-B* is true, the remaining variables must all be false. Both A and B have an associated integer variable, *index-A* and *index-B*, and we state that if there is an edge from A to B, B must be assigned a greater value than A:

activation-A-B || inhibition-A-B ==> index-A < index-B

A similar constraint is asserted for the opposite direction. Finally, we create a constraint that requires B to have an incoming edge if it has an outgoing edge, based on its neighbors in the undirected graph:

activation-B-A || inhibition-B-A || activation-B-D || inhibition-B-D

==>

activation-A-B || inhibition-A-B || activation-D-B || inhibition-D-B

These constraints together guarantee that a valid solution must be a tree network rooted at A. ■

While the above constraints will ensure that solutions satisfy topological properties, they don't constrain models with respect to the temporal data. Using the temporal events computed from the time series data, TPS requires that the sequence of nodes in each signed, directed path of a tree model must be supported by a corresponding temporally ordered sequence of phosphorylation events. In the example from Figure 5, there can be no models that include an edge from E to D, because it is impossible for E to precede D in a directed path due to all possible activations of E being later than the possible activation of D (Table 1). The same example shows that the temporal ordering along paths can also have an effect beyond pairwise interactions. The sequence of nodes E, G, F cannot appear on a directed path, even though both pairwise interactions are locally consistent, because E can only be activated strictly after F. Concretely, this constraint is enforced by keeping an integer-valued variable for each node, which corresponds to the choice of activation time for that node. The same is done for representing inhibitions, and we assert that at most one of the two events can occur. We restrict the values that the activation variable can take to the time points computed in the discretization step. Finally, we state that if there is an edge from A to B in the signed, directed tree, there must be corresponding choices of time points for A and B that support the interaction. An activation edge from A to B must be supported by the activation (respectively, inhibition) of A, succeeded by the activation (respectively, inhibition) of B; similarly, an inhibition from A to B requires finding an activation (respectively, inhibition) of A, succeeded by the inhibition (respectively, activation) of B.

Example: Consider the nodes D and E in Figure 5. We constrain activation choices for D to be 0 (no activation) or 1 (the first time interval):

activation-D == 0 || activation-D == 1

Similarly, E is either not activated or is active in intervals 2 or 3:

activation-E == 0 || activation-E == 2 || activation-E == 3

Finally, we assert that if there is an activation from D to E, both nodes must be activated or inhibited, in that order:

activation-D-E ==>

activation-D != 0 && activation-E != 0 && activation-D <= activation-E

We only show constraints for the activation of E by D through their successive activation events, the other cases are similar. ■

The last type of constraint that TPS enforces follows simply from the prior knowledge information. For all known kinase-substrate interactions (given as directed, unsigned edges), no pathway model can include an edge directed in the opposite orientation. This is implemented by ruling out certain values for the edge variables if data is available for a given edge. TPS currently represents kinase-substrate interactions as unsigned but could be trivially extended to treat kinase-substrate interactions as positive edges and phosphatase-substrate interactions as negative edges.

Example: In the example from Figure 5D, we are given the kinase-substrate interaction C-D. As a result, we rule out the opposite direction:

!activation-D-C && !inhibition-D-C

■

Extending constraints to model feedback loops

Although TPS does not model feedback loops, it is possible to extend our declarative constraint solving approach to infer network models with feedback. We sketch below one way to allow each node to change its binary activity level up to N times, thereby allowing a node's early activity to influence its late activity through cycles in the network model. The high level idea consists of using multiple copies of the constraint system we describe above, each copy corresponding to a different "layer" in the network. Conceptually, each layer describes temporal activity that comes after all activity in the previous layer. While each layer corresponds to a tree network (using our existing constraint system), their composition represents cyclic graphs that can model feedback.

For the layered system of constraints, we need to formulate constraints that relate each successive layer to its predecessor. Specifically, having an outgoing edge from a node in a given layer requires the same node to have an incoming edge in the same layer or the preceding layer. Furthermore, because each layer corresponds to activity that follows the previous layer, the activation interval of a node in one layer must not come before its chosen activation interval in the preceding layer. Together, these requirements ensure that all activity seen across layers originates from the source node in the first layer, and the order of node activations across layers is correct.

Example: We revisit the constraints generated for the edge A-B in the example undirected graph shown in Figure 5C. If we want to model at most two activity intervals per node, we will have two copies of the Boolean variables expressing the sign and orientation of the edge, and we will ensure that at most one Boolean variable is true per layer by asserting the constraint below. We refer to the Boolean variable representing an activation from A to B in layer *i* as $activation_i$*-A-B*.

$activation_1$*-A-B ==> !*$inhibition_1$*-A-B && !*$activation_1$*-B-A && !*$inhibition_1$*-B-A*

&&

$activation_2$*-A-B ==> !*$inhibition_2$*-A-B && !*$activation_2$*-B-A && !*$inhibition_2$*-B-A*

The modified connectivity constraint for node B will now require B to have an incoming edge in the current layer or the previous layer based on its neighbors in the undirected graph. For instance, we will assert the following for layer 2:

$activation_2$*-B-A* || $inhibition_2$*-B-A* || $activation_2$*-B-D* || $inhibition_2$*-B-D*

==>

$activation_2$*-A-B* || $inhibition_2$*-A-B* || $activation_2$*-D-B* || $inhibition_2$*-D-B*

||

$activation_1$*-A-B* || $inhibition_1$*-A-B* || $activation_1$*-D-B* || $inhibition_1$*-D-B*

■

It is possible to further extend this modeling approach to require successive activations of the same node to correspond to distinct time intervals. This can be achieved by asserting that if a node has an incoming edge in layer *i* and an outgoing edge in a preceding layer *j*, then the activation interval picked for layer *i* must come strictly after the interval picked for layer *j*.

Example: For our running example, we will assert the following constraint for node B. We denote the variable corresponding to the activation interval chosen for B in layer *i* as $activation_i$-B.

$$activation_2\text{-}A\text{-}B \;||\; inhibition_2\text{-}A\text{-}B \;||\; activation_2\text{-}D\text{-}B \;||\; inhibition_2\text{-}D\text{-}B$$

$$\&\&$$

$$activation_1\text{-}B\text{-}A \;||\; inhibition_1\text{-}B\text{-}A \;||\; activation_1\text{-}B\text{-}D \;||\; inhibition_1\text{-}B\text{-}D$$

$$==>$$

$$activation_1\text{-}B < activation_2\text{-}B$$

■

This approach to model feedback loops using constraints is similar to the way dynamic Bayesian networks create multiple copies of each variable by "unrolling" the network over each time point. Whereas DBNs have to make N copies of the network for N time points, we only need one copy per node activation. Using the above constraint formulation, a solution without feedback loops will still use only one copy of each variable. If we allow two activations per node, we will only need two copies of each variable, irrespective of how many time points there are in the time course dataset.

Pathway summarization

The space of all valid pathway models with timing annotations defined by the constraints we specified is typically very large, and enumerating all models is not computationally feasible. Given an undirected network G with V nodes and E edges, along with T time points, there are 5^E ways of assigning a sign and orientation to edges of G and $(T*2 + 1)^V$ ways of assigning timing annotations to its nodes. Even for a network with 200 edges, the number of possible sign and orientation assignments is $6*10^{139}$. Pathway summarization supports these large state spaces because is it intractable to enumerate all valid pathway models.

The main text describes the edge query procedure used to generate pathway summaries. The example summary in Figure 6 and the summary expansion in Figure S7 illustrate how this summary can contain a superset of the valid pathway models. There exists no valid model that contains an activation from A to B and an inhibition from B to D. The existence of the first edge dictates that B is activated, which implies an inhibition from B to D would decrease D's activity, contrary to what is observed in D's temporal activity profile. However, the knowledge that the edges A-B and B-D must have the same sign is lost through the summarization process.

For visualization and analysis purposes, pathway summaries are depicted as interactions among proteins even though the temporal consistency constraints operate at the level of individual peptides when peptide-level data are available. The protein-level summaries collapse the expanded PPI network, which can introduce ambiguities if there are interactions that are unambiguous at the peptide-level that conflict in terms of direction or sign at the protein-level. TPS is able to detect and report this loss of precision when transitioning to the protein-level network.

A final summarization observation relates to the distinguishability problem between trees and directed acyclic graphs (DAGs) that we discussed in the context of our modeling assumptions. We note that summarizing the space of all tree models as a union graph leads to the same result as summarizing the space of DAGs satisfying the same properties. This stems from the fact that for each DAG model, there exists a set of tree models whose union is the DAG. As a result, the union of all tree models corresponds to the union of all DAGs.

Tradeoffs between ambiguity, expressiveness, and correctness

The modeling assumptions made when interpreting and translating biological data into logical constraints have complex effects on the degree of ambiguity, expressiveness, and accuracy of the resulting pathway summary. Even with temporal information, many pathway structures can explain the ordered signaling events. This motivates the

reduction of ambiguity with hard logical constraints, where each constraint is fully trusted, instead of with probabilistic constraints (Hinton et al., 2006; Katoen et al., 2005), where a constraint can potentially be violated.

In the PPI network, we allow paths only through chains of experimentally detected PPI. In settings where the PPI network is less complete, we could include edges among highly correlated phosphorylated proteins or predicted interactions based on protein sequence, protein structure, pathway connectivity, or literature mining (Lees et al., 2011; Mosca et al., 2013). The pre-processing step that filters the PPI network operates on a weighted network. These additional edges could be assigned lower weights so that PCSF includes them in the TPS input network only if they are critical for connecting significantly phosphorylated proteins. This would reduce the impact of missing interactions on TPS pathways at the cost of potentially increasing ambiguity because there would be more possible paths through which signal can flow.

Likewise, we observe that some proteins, such as RAS and RAF family members, are not included in the TPS pathway because our mass spectrometry data do not detect their phosphorylation. To increase robustness to potential false negatives in the mass spectrometry, the input PPI network could be modified to include edges from relevant reference pathways with high weights (similar to (Patil et al., 2013)) so that PCSF prefers to include these interactions instead of other high-confidence connections in the PPI network. The weight of these prior knowledge edges would control the tradeoff between condition-specific *de novo* pathway discovery and conformance with prior knowledge.

Unlike single-cell mass cytometry data, where the peak activity times of a small number of phosphoproteins can be resolved precisely (Krishnaswamy et al., 2014), phosphorylation timing in cell population-level mass spectrometry data is inherently ambiguous. Therefore, instead of rigidly determining a protein's time of activity by selecting the time point at which the greatest phosphorylation change is observed, TPS takes a more general approach. It allows a protein to be activated or inhibited whenever the phosphorylation significantly differs from the level before stimulation or at the immediately preceding time point as long as it is the *first time* at which that phosphorylation level has been observed. We focus on the initial pulse of signaling activity following stimulation, sampling more early time points in our EGF response study because we are more confident that these changes in phosphorylation intensity are due to PTMs instead of changes in protein abundance. Feedback loops cannot be detected when learning a single activation or inhibition time per peptide, a modeling decision we made in our three case studies. However, the TPS framework makes it possible to allow multiple activity changes per peptide in future applications, as we outlined above. Statistical tests of the temporal phosphorylation profiles could determine the number of significant activity changes for each peptide. Then, TPS could search for pathway structures with feedback loops that explain the multiple activation or inhibition events per peptide.

Lastly, we recognize that different phosphopeptides on the same protein can have different phosphorylation changes over time, and we allow each peptide to have its own activation times instead of forcing a single time per protein. This decision can lead to ambiguous edge direction predictions at the protein-level even when the directions are consistent at the peptide level. For example, DOCK1 interacts only with BCAR1 (Figure S4B), yet the direction and sign of the interaction are ambiguous. The uncertainty arises because BCAR1 is phosphorylated on both Y249 and Y387. TPS correctly concludes that the sign cannot be determined because one site could activate DOCK1 and then feed back and affect the other BCAR1 site.

Prize-collecting Steiner forest

PCSF recovers the sparse subnetwork $F = (V_F, E_F)$ from a dense PPI network that links proteins of interest by solving

$$\operatorname*{argmin}_F \sum_{v \notin V_F} (\beta \cdot p(v) - \mu \cdot d(v)) + \sum_{e \in E_F} c(e) + \omega \cdot \kappa$$

where p is a positive score (prize) that reflects the relevance of a vertex (protein) v, d is the degree (number of neighbors) of v, c is a positive cost for including an edge (interaction) e in the subnetwork, and κ is the number of disconnected trees in the subnetwork. Parameters β, μ, and ω control the size and structure of the solution subnetwork. We swept over many PCSF parameter combinations and identified the parameters that produced reasonably large subnetworks containing many phosphorylated proteins. Including a greater fraction of phosphorylated proteins in the PCSF subnetwork, as opposed to the connective Steiner nodes without observed phosphorylation changes, provides more temporal information for the TPS constraints. An advantage of PCSF is

that it nominates pathway members that are not detected by the mass spectrometry but form critical pathway connections to phosphorylated proteins, like ABL2 and AKT1 in our EGF response study (Figure S4).

In our EGF response analysis, we computed the node prize for each protein using the minimum peptide p-value over all peptides that map to the protein. We computed prizes as $-\log_{10} pvalue$, yielding 701 protein prizes. The proteins were not filtered at a particular p-value threshold. There are no replicates in the Olsen et al. EGF response dataset so we used phosphorylation fold changes to define prizes instead of p-values. For each peptide, we computed the $\log_2$ phosphorylation fold changes at each time point with respect to both the previous time point and the initial 0 minute time point. We extracted the maximum absolute value $\log_2$ fold change for each peptide as the peptide prize. We then derived protein prizes by taking the maximum peptide prize over all peptides that map to a protein.

The human PPI network we used for both EGF analyses was obtained from two sources: 159,095 undirected interactions from iRefIndex (version 13.0) (Razick et al., 2008) and 4,080 directed kinase-substrate interactions from PhosphoSitePlus (downloaded October 16, 2013) (Hornbeck et al., 2015). The iRefIndex database provides interaction confidence scores that are inspired by the Molecular INTeraction database (MINT) (Ceol et al., 2010; Villaveces et al., 2015), which account for the number of publications supporting the interaction, the type of interaction, and the experimental detection methods. Similarly, we scored the directed interactions based on the number of interactions reported for each kinase-substrate pair (across all substrate sites) and whether an interaction was detected *in vitro*, *in vivo*, or both. When merging the iRefIndex and PhosphoSitePlus interactions, if both databases reported an interaction between a pair of proteins we retained the more specific, directed kinase-substrate interaction and its weight, discarding the undirected iRefIndex interaction. The final network contains 15,677 proteins, 157,984 undirected interactions, and 3,917 directed interactions (reciprocal pairs of directed edges with the same score are represented as an undirected edge). It contains 653 of the 701 proteins with mass spectrometry-based prizes from our EGF response data. PCSF respects the direction of the kinase-substrate interactions so that they can only be used in the specified direction.

We derived PCSF protein prizes for the yeast osmotic stress study from the time-point specific salt/control $\log_2$ fold changes. For each protein, we computed the maximum absolute value $\log_2$ fold change considering all phosphopeptides mapping to that protein and all time points. This produced 1,596 protein scores from the 4,337 phosphopeptides. Because a complete control time series was available, we also used the temporal salt versus control $\log_2$ fold changes to obtain the temporal significance scores for TPS. Kanshin et al. used the non-phosphorylated peptides that were not filtered during phosphopeptide enrichment to define a null distribution of $\log_2$ fold changes at each time point (Kanshin et al., 2015). From these null distributions, they derived time point-specific $\log_2$ fold change thresholds that correspond to a p-value < 0.05 after adjusting for multiple hypothesis testing (Table S2). We called a phosphopeptide significant at any time point where its $\log_2$ fold change was more extreme than the time point-specific threshold, which produced 1,401 phosphopeptides and 784 proteins with significant dynamic responses. We did not consider the 0 second fold changes. All analyses considered only the singly phosphorylated (1P) peptides.

Chasman et al. compiled the yeast interaction network we used for the osmotic stress model from many sources (Chasman et al., 2014), with the vast majority of kinase-substrate interactions coming from Ptacek et al. (Ptacek et al., 2005). We removed edges where the interaction evidence was listed as "inferred" or the edge type was "cxcx", "cxorf", or "metapath". To emphasize post-translational modifications, we retained 7,583 directed interactions from this network, which included 4,875 phosphorylation-specific interactions with the edge type "kinasesubstrate", "literaturephos", or "phosphatasesubstrate". Not all source nodes were connected to the rest of the network with these directed edges so we also included 32 undirected PPI between the sources and other proteins. Because the network was originally unweighted, we assigned a default weight of 0.5 to all 7,615 edges.

The PCSF parameters β, μ, and ω influence the structure of the optimal PPI subnetwork (Tuncbag et al., 2016). As β grows, the incentive to include (de)phosphorylated proteins increases, outweighing the expense of adding additional edges to connect them. The PCSF solution includes novel proteins, termed Steiner nodes, that are not phosphorylated but are useful for connecting the proteins with positive prizes. To control for hub proteins in the Steiner forests, which can produce non-condition-specific solutions due to their high-connectivity and ability to link many phosphorylated proteins, μ penalizes the inclusion of high-degree nodes. Large μ results in fewer hubs in the forest.

In order to select the parameters β and μ for our EGF response data, we ran PCSF with all combinations of β from 0.05 to 1.0 (step size of 0.05) and μ from 0 to 0.01 (step size of 0.001), producing 220 Steiner forests. Because the source of stimulation is known and specific, we rooted the subnetwork at EGF. This forces $\kappa = 1$, reducing the problem to the prize-collecting Steiner tree problem and removing the influence of ω. We set msgsteiner's parameters $D = 10$ and $g = 0.001$ (msgsteiner version 1.1) (Bailly-Bechet et al., 2011). After removing the 34 small or empty solutions with two or fewer Steiner nodes, we selected $\beta = 0.55$ and $\mu = 0.008$, the solution that maximized the fraction of proteins in the Steiner forest that had prizes (were phosphorylated or dephosphorylated in response to EGF). In order to recover alternative connections among the (de)phosphorylated proteins, we generated a collection of 100 PCSF networks. For each PCSF run, we used the previously selected β and μ and added random noise to all interaction costs by setting msgsteiner's $r = 0.01$. The union of these 100 networks was used as input to TPS.

The Olsen et al. EGF response PCSF analysis used the same source node, parameter sweeping strategy, and msgsteiner parameters (msgsteiner version 1.3). For this dataset, we discarded 47 solutions with two or fewer Steiner nodes, including empty networks, and chose $\beta = 0.45$ and $\mu = 0.005$ for generating the final collection of 100 PCSF networks. These parameters produced a network in which the fraction of proteins with prizes, 0.88, was slightly less than the maximum over all solutions, 0.90. However, we preferred this network because it was closer in size to the PCSF network obtained using our EGF response data, facilitating their direct comparison.

For the yeast osmotic stress study, we selected the five sources from the KEGG yeast high osmolarity pathway: Hkr1, Msb2, Opy2, Sho1, and Sln1. We ran PCSF with all combinations of β from 0.25 to 10.0 (step size of 0.25), μ from 0 to 0.1 (step size of 0.005), ω from 0.5 to 10 (step size of 0.5), generating 16,800 Steiner forests. We discarded 14,562 solutions with two or fewer Steiner nodes, including empty networks. Multiple Steiner forests had the same fraction of proteins with prizes, all of which had the same β and μ parameters. From this set of forests, we selected $\beta = 1.75$, $\mu = 0.095$, and $\omega = 4.5$ to generate a collection of 1000 Steiner forests using the same msgsteiner parameters as the EGF analyses (msgsteiner version 1.3).

For all three phosphoproteomic datasets, the temporal phosphorylation data included measurements of individual peptides. TPS expands the subnetwork from PCSF by replacing each protein node by a collection of peptide nodes. These peptide nodes are all peptides that are significantly phosphorylated in the mass spectrometry data and map to the protein. The protein-protein edges are transformed to peptide-peptide edges by connecting all peptide nodes that replace protein A to all peptides nodes that replace protein B if there was a PPI between A and B.

Phosphoproteomic and network data randomization

We performed three types of data randomization to evaluate the impact on the TPS predictions for our EGF response data. First, we permuted the peptide to protein assignments. Next, we permuted time points within each time course except for the baseline (0 min) time point. Finally, we permuted the PPI network with BiRewire (Iorio et al., 2016). BiRewire supports undirected or directed graphs but not partially directed graphs, so we randomized the directed edges and undirected edges separately and then merged them. For each type of permutation, we ran PCSF and TPS on 100 different versions of the randomized data. Because the phosphorylation timing does not affect the protein prizes, we did not rerun PCSF when permuting the time points. Instead, we reused the PCSF subnetwork from the original data. The PCSF and TPS parameters for the permuted datasets were the same as for the original data. We collected aggregate metrics that compare the permuted data results to the TPS network obtained using the original dataset (Köksal et al., 2018).

In addition, we performed bootstrap analyses by randomly removing 10, 25, or 50% of the significant phosphopeptides. For each fraction of held out data, we generated protein prizes and ran PCSF and TPS 100 times using the original parameters. We again computed aggregate metrics that compare the permuted data results to the TPS network obtained using the original dataset (Köksal et al., 2018).

Evaluation against pathway databases

In order to compare our predictions against a set of manually curated pathway databases, we developed a framework that collects signed, directed protein-protein interactions from the BioPAX L3 and SIF formats. Our framework allows us to perform a systematic comparison against reference pathways. We extracted pathway interactions from multiple references represented using BioPAX L3. The comparison against SIF networks is straightforward because SIF files are simply lists of pairwise protein-protein interactions.

Extraction from pathway maps is performed in two steps. First, we extract all proteins, complexes, and paths reported between these entities. Then, we query the extracted paths for reachability between pairs of proteins, following rules that allow us to handle a multitude of formalisms used by different references to represent pathways within the BioPAX L3 format.

Not all reference pathway maps use the same types of identifiers. We therefore establish a name correspondence between the protein interaction network and all pathway maps by using protein-gene name mappings and gene synonyms. In order to find entities corresponding to a protein of interest, we query pathway maps with the UniProt protein name and identifier, as well as the corresponding gene name and Entrez gene synonyms retrieved from NCBI at ftp://ftp.ncbi.nih.gov/gene/DATA/GENE_INFO/Mammalia/Homo_sapiens.gene_info.gz. This can introduce ambiguity in the analysis because some pathway databases use non-standard gene symbols or a single symbol to represent multiple genes in the same family. For example, ABL2 is a member of KEGG's ErbB signaling pathway, but we did not annotate it as a known EGFR pathway member in Figure S4 due to these gene naming differences. Rather than manually adjust the gene name matching, we use a fully automated process in order to make unbiased claims about pathway converge in Figures S1 and S2.

Extracting proteins, complexes, and paths between them from BioPAX L3

In the first step, we compute all paths from a protein or complex to another protein or complex without going through an intermediate protein, complex or small molecule. We explicitly handle different representations of interactions including catalysis, control, template reaction regulation, and complex assembly.

A *catalysis* has a controller entity and a controlled conversion. It may optionally have a catalysis direction, which indicates whether the conversion is catalyzed left-to-right or right-to-left. We say that when an entity E is a controller of a conversion C, then the entities at one or both sides of the conversion are reachable from E (depending on the catalysis direction information).

A more general interaction type than catalysis, a *control* has a controller entity and a controlled interaction and does not specify a direction for the controlled interaction. We say that when entity E controls interaction I, both the left and right participants of I can be reached from E.

A *template reaction regulation* expresses the regulation of a template reaction by a physical entity. Template reactions only have a product, which we say is reachable by the entity controlling the regulation.

A *complex assembly* has left and right participants. If P is a left participant, then a right participant complex is reachable from P. We do not use this type of path currently, with a protein start node and complex end node. We instead just look for common complexes that contain two given proteins.

Additionally, we use the reported *molecular interactions* to infer complex assemblies. We say that entities E_1 and E_2 appear in a complex when they are participants in the same molecular interaction.

Computing protein interactions from extracted paths

Now that we have information about which complexes and which protein/complex paths exist in a resource, we can query the extracted paths for reachability from one protein to another.

Interactions between proteins may be expressed either between the entities directly or indirectly through successive steps of complex formation. We want to use the former when it is available because it usually provides information on interaction directions unambiguously. Considering paths that have complexes at their endpoints, or even simply analyzing which proteins appear in the same complex is essential for mining some interactions, but only considering the more specific, direct interaction information results in a better analysis. For this reason, we perform a staged reachability query with three different types of queries.

In the first stage, we look for paths that have A and B at each end. If such a path can be found for at least one direction, we stop the process and report the direction(s). If an interaction cannot be found in the first stage between A and B, we advance to a second stage of querying, in which we search for complex-to-protein interactions from, for instance, C to B, where A is a member of the complex C. Note that A might be transitively contained in C through another complex contained by C. We further refine this interaction by enforcing that A must have joined complex C no earlier than other non-complex components. If we can find complex assemblies that produce C, we

check whether A is a direct participant of the complex assembly (as opposed to having earlier formed a complex that is a participant in the complex C). If no complex assemblies can be found, we check whether A is a direct participant of the complex C (as opposed to being a participant in a complex that is itself a participant of C).

If neither of the above stages result in inferring a direction, we perform a third and final query, looking for a common complex that contains both A and B. If there is such a complex, we infer an interaction in both directions.

Pathway resources
We applied our data extraction procedure to eight human pathway references, which are shown with their full names and identifiers when available:

- NCI-Nature Pathway Interaction Database "EGF receptor (ErbB1) signaling pathway" (erbb1_receptor_proximal_pathway): http://pid.nci.nih.gov/download.shtml
- Reactome "Signaling by EGFR" (R-HSA-177929): http://www.reactome.org/ReactomeGWT/entrypoint.html
- BioCarta "EGF signaling pathway" (h_egfPathway): http://pid.nci.nih.gov/download.shtml
- Cancer Cell Map "EGFR1": http://www.pathwaycommons.org/pc/dbSnapshot.do?snapshot_id=8
- KEGG "MAPK signaling pathway" (hsa04010) and "ErbB signaling pathway" (hsa04012): http://ipavs.cidms.org/downloads
- Layek et al.: Manually reconstructed from Figure 4 of (Layek et al., 2011)
- *Science Signaling* Database of Cell Signaling "Epidermal Growth Factor Receptor Pathway" (CMP_14987): Originally manually reconstructed from the image at http://stke.sciencemag.org/cgi/cm/stkecm;CMP_14987, which was archived in June 2015 and is no longer available. The XML version is available from http://stke.sciencemag.org/about/help/cm.

This collection of reference pathways includes: EGFR pathway maps from six popular databases (Croft et al., 2014; Gough, 2002; Kandasamy et al., 2010; Kanehisa et al., 2012; Nishimura, 2001; Schaefer et al., 2009); a Boolean circuit representation of growth factor signaling (Layek et al., 2011); and the related but more general mitogen-activated protein kinase (MAPK) pathway from KEGG. These resources reflect the diverse goals and biases of different pathway curators. BioCarta focuses on the most essential signaling events, containing only 16 proteins. Conversely, Cancer Cell Map, which is part of the NetPath resource (Kandasamy et al., 2010), seeks broader coverage. Its EGFR map contains 178 proteins, approaching the 202 proteins cataloged in a thorough EGFR review (Oda et al., 2005).

For the yeast evaluation, we downloaded the KEGG pathway "MAPK signaling pathway" (sce04011) from https://www.genome.jp/kegg-bin/show_pathway?map=sce04011. We used capDSD (Cao et al., 2014) to parse the XML file and Cytoscape (Shannon et al., 2003) to retain only the edges in the "High osmolarity" connected component of the KEGG pathway. Finally, we exported the Cytoscape network into a SIF format for the evaluation. The SIF file was a multigraph, containing more than one edge for some protein pairs. We retained the most specific direction and sign available for each protein pair.

In addition, we considered an upper and lower bound when calculating the directed precision (Supplemental File 4) because not all KEGG pathway edges have reported directions. Both cases consider edges that are in the KEGG pathway (directed or undirected) and in the TPS network with a specific predicted direction as the positive predictions. For the upper bound on directed precision, the true positives are the edges for which the predicted direction does not conflict with the reported direction in KEGG. For the lower bound, the true positives are the edges for which KEGG reports a unique direction that agrees with the TPS prediction.

Natural language processing evaluation
We used three NLP tools – iHOP (Hoffmann and Valencia, 2004), Chilibot (Chen and Sharp, 2004), and Literome (Poon et al., 2014) – to perform unbiased literature searches for previous evidence of direct interaction for the 54 fringe pathway predictions. Fringe predictions are interactions that are at the periphery of the canonical pathway, meaning one or both of the interacting proteins are members of a reference EGFR pathway map but there is no interaction reported. Each NLP tool takes gene symbols as input so we mapped the UniProt identifiers in our predicted pathway edges to gene symbols. For each pair of genes, the NLP tools report the sentence from a PubMed abstract that is believed to describe an interaction among the corresponding proteins and either the full abstract or

the PubMed Unique Identifier (PMID), which can be used to retrieve the full abstract. All three tools perform entity normalization to automatically reconcile synonyms that refer to the same gene or protein. We did not intend to evaluate the quality of the NLP itself so we manually read the entire abstract to select the sentence that most specifically confirms or denies the predicted interaction, which is not always the sentence identified by the NLP tool. Literome searches for direct interactions and indirect interactions that are mediated by an intermediate third protein, and we only considered the direct interactions. For Chilibot, we considered interactive sentences but not parallel sentences. We classified the degree of support for each interaction using the categories in Supplemental File 3 and summarized the evidence by taking the most specific sentence across the three tools. When evaluating interaction sign, we assessed the interaction's impact on the target protein's phosphorylation, not its function. Because some interactions may have conflicting roles in different contexts and abstracts and each NLP tool has unique strengths, we kept the sentence that best matched our prediction when there were multiple possibilities or conflicts.

Osmotic stress evaluation

Predicted, direct Rck2 targets in the TPS network were compared against a recent phosphoproteomics study on an *RCK2* mutant strain that identified peptides with defective phosphorylation 5 minutes after treatment with 0.5M NaCl (Romanov et al., 2017). We defined defective phosphosites as those that exhibited a wild type osmotic stress response and at least a 1.5-fold defect in *rck2*Δ compared to the wild type. Phosphosites with a wild type osmotic stress response included those with at least a 2-fold increase in phosphorylation in the wild type strain after NaCl treatment in the Romanov et al. study and those that were not quantified in that condition by Romanov et al. but responded significantly to salt stress in the Kanshin et al. study (Kanshin et al., 2015). Cdc28 targets were merged from two *cdc28-as* studies (Holt et al., 2009; Kanshin et al., 2017) using the substrate identification techniques they proposed. Holt et al. required phosphopeptides to match the Cdc28 consensus sequence and a 50% decrease in phosphorylation. Kanshin et al. trained a support vector machine to identify direct Cdc28 substrates. For both Rck2 and Cdc28, we only considered defective phosphosites that were quantified by Kanshin et al. (Kanshin et al., 2015) because those phosphorylated proteins were provided as input to TPS.

Running the dynamic Bayesian network, TimeXNet, and FunChisq

We downloaded the dynamic Bayesian network software (Hill et al., 2012) from http://mukherjeelab.nki.nl/DBN.html. We constructed a network prior that indicates, for each pair of peptides in the time series data, whether there exists an edge corresponding to the pair in the protein-protein interaction network we prepared for PCSF. We ran the method with a maximum in-degree of 2 nodes, which is the largest value that the DBN could handle for this dataset. The method chose an optimal prior strength of 1.5 for inference. Edges were generally assigned low probability values across the network with a maximum edge probability of 0.10967. To obtain a final network of size comparable to the TPS output, we filtered the DBN predictions by a minimum probability value of 0.025. We treated opposite directed predictions for the same pair of proteins as one undirected edge in our comparative evaluations.

We downloaded the TimeXNet software (Patil and Nakai, 2014; Patil et al., 2013) from http://timexnet.hgc.jp/ and ran it with the same weighted protein-protein interaction network we prepared for PCSF. To assign proteins to temporal groups, we selected the time point at which the most significant phosphorylation change occurs, considering all peptides that correspond to a protein. The initial temporal group contained proteins with the most significant change at 2 or 4 minutes. The intermediate group contained the 8, 16, and 32 minute proteins. The 64 and 128 minute proteins composed the late group. As in PCSF, we computed node scores as $-\log_{10} pvalue$. Following the parameter selection guidelines in (Patil et al., 2013), we ran TimeXNet using all combinations of γ_1 and γ_2 from 0 to 5 (step size of 0.5). There were no subnetworks with less than 1% unreliable edges when defining unreliable edges as edges with weight less than 0.5. We instead defined unreliable edges as those with edge weight less than 0.3 and selected $\gamma_1 = 4.5$ and $\gamma_2 = 0$ as the optimal parameters. Among the 11 subnetworks with less than 5% unreliable edges, these parameters maximized the number of source nodes and minimized the fraction of unreliable edges. We did not filter the subnetwork by node or edge flow and treated all edges as directed edges based on the flow direction.

We downloaded FunChisq (Zhang and Song, 2013) and the associated data discretization tool Ckmeans.1d.dp as R packages. We wrote a wrapper in Scala that uses the two packages to provide an end-to-end inference pipeline that accepts time series data and produces FunChisq scores (a statistic and a p-value) for every interaction. The wrapper is available publicly at https://github.com/koksal/funchisq-wrapper. We discarded time series profiles that were

discretized into only one level by Ckmeans.1d.dp. We mapped the peptide-level output of FunChisq to the protein level, which produced duplicate protein-level edges when multiple peptides mapped to the same protein. We aggregated these duplicate edges by taking the minimum p-value observed for each edge. We filtered the edges by keeping those with p-value ≤ 0.01 and limited the network to 1000 edges by descending FunChisq statistic to be comparable in size to the other algorithms. As for the dynamic Bayesian networks, we treated opposite directed predictions for the same pair of proteins as one undirected edge in our comparative evaluations.

Contrasting TPS with related computational approaches

Approaches for building networks from gene expression data alone (reviewed in (De Smet and Marchal, 2010)) can be applied to phosphoproteomic data as well. Extensions of these methods for temporal data introduce time lags and search for dependencies between genes' expression levels over time (Zoppoli et al., 2010). Methods based on Granger causality (Masnadi-Shirazi et al., 2014) identify proteins whose phosphorylation predicts behavior at later time points and provide one type of causal model. However, as we showed in our comparison with the dynamic Bayesian network (Hill et al., 2012) and FunChisq (Zhang and Song, 2013), these and other methods that rely on the phosphorylation data alone (Henriques et al., 2017) miss critical signaling pathway interactions because not all pathway members have observed phosphorylation changes.

Algorithms based on gene and protein perturbations provide an alternative approach toward causal models. Transcriptional regulatory networks have been inferred from expression changes induced by gene knockouts and knockdowns (Anchang et al., 2009; Markowetz et al., 2007; Wang et al., 2014; Yeang et al., 2004). Likewise, signaling networks have been reconstructed by stimulating a pathway and perturbing signaling nodes with kinase inhibitors or RNA interference. Protein activities are observed with antibody-based assays, and pathways are recovered *de novo* (Ciaccio et al., 2015; Fröhlich et al., 2009; Kiani and Kaderali, 2014; Molinelli et al., 2013) or by adapting prior pathway knowledge (Morris et al., 2011). The PHONEMeS method is unique for its ability to handle large-scale phosphoproteomic perturbation data (Terfve et al., 2015).

The HPN-DREAM network inference challenge (Hill et al., 2016) spawned several new approaches for analyzing time series phosphorylation data. Participants predicted signaling pathways from *in silico* time series data and temporal reverse phase protein array data for approximately 45 phosphoproteins in four breast cancer cell lines under various stimuli and inhibitor treatments. In contrast, TPS focuses on the unique demands in modeling and scaling to global phosphoproteomic data with over one hundred thousand phosphosites that are not encountered when modeling only tens of proteins. PropheticGranger (Carlin et al., 2017), the top performer in the HPN-DREAM experimental task, demonstrated the importance of prior knowledge in network inference and modified the standard Granger causality approach to assess dependencies between observed proteins. However, PropheticGranger did not scale when we applied it to our EGF response data with a complete PPI network as the prior. FunChisq (Zhang and Song, 2013), the top performer in the HPN-DREAM *in silico* task, was able to run on our data but did not perform well (Supplemental File 4).

In our EGFR study, the TPS PPI subnetwork input is provided by PCSF, but other network algorithms can also connect phosphorylated proteins using PPI. A related algorithm interpolates between globally optimal (Steiner tree) and locally optimal (shortest path) connections to different proteins (Yosef et al., 2009), and this method has been applied to link functional signaling proteins derived from phosphoproteomics data (Rudolph et al., 2016). Many other approaches connect source and target proteins in a PPI network to identify pathways. ResponseNet (Yeger-Lotem et al., 2009) does so with a maximum flow formulation; SHORTEST (Silverbush and Sharan, 2014) and PathLinker (Ritz et al., 2016) use shortest paths; Maximum Edge Orientation (MEO) (Gitter et al., 2011) orients the undirected edges to produce short, directed paths. Integer programs can express complex optimization preferences with multi-stage objective functions when predicting source-target connections (Chasman et al., 2014; MacGilvray et al., 2018). The predicted networks from any of these methods can be used as input for temporal analysis with TPS.

Among methods that integrate dynamic data and PPI networks, TPS is unique in its ability to assess and summarize all possible pathway structures that are consistent with the input network and the temporal constraints. TPS also considers all possible temporal activations for each peptide instead of mapping proteins to temporal bins in advance like TimeXNet (Patil and Nakai, 2014; Patil et al., 2013). Similarly, Budak et al. use time point-specific PCSF networks to map proteins to times (Budak et al., 2015), TimePath assigns genes to transcriptional phases based on gene expression timing (Jain et al., 2016), Khodaverdian et al. explore theoretical properties of temporal Steiner trees (Khodaverdian et al., 2016), and ST-Steiner allows early subnetworks to influence the structure of subsequent

subnetworks (Norman and Cicek, 2018). The Signaling and Dynamic Regulatory Events Miner (SDREM) models temporal gene expression to infer the timing of transcription factor activity, but the pathway discovery phase does not use any temporal information (Gitter and Bar-Joseph, 2013; Gitter et al., 2013). Vinayagam et al. used temporal phosphorylation to evaluate their predicted PPI directions but did not consider dynamics when making the predictions (Vinayagam et al., 2011). Time series data and interaction networks have also been combined for inferring protein complex dynamics (Park and Bader, 2012), pathway enrichment (Jo et al., 2016), and related problems reviewed in Przytycka et al. (Przytycka et al., 2010).

The key difference between our work and other declarative computational approaches is that TPS operates on networks that are several orders of magnitude larger and summarizes very large solution spaces defined by sparser and less precise experimental data. Model checking and symbolic reasoning have been used to verify properties of manually constructed biological models (Fisher and Piterman, 2014), complete partially specified pathways using perturbation data (Köksal et al., 2013), and synthesize gene regulatory networks directly from data (Dunn et al., 2014; Moignard et al., 2015) (reviewed in (Fisher et al., 2014)). In addition, other types of declarative approaches, such as integer programming (Budak et al., 2015; Chasman et al., 2014; Jain et al., 2016; Ourfali et al., 2007; Sharan and Karp, 2013; Silverbush and Sharan, 2014) and answer set programming (Guziolowski et al., 2013), have been applied to biological pathway analysis. The TPS model summarization strategy, which makes it applicable to comprehensive signaling networks containing more than a hundred thousand edges and phosphosites, sets it apart from these related methods (Figure S6).

Random data generation for scalability analysis
In order to evaluate the ability of TPS to scale to large datasets, we randomly generated graphs, time course phosphorylation profiles, and protein-peptide maps of increasing size, up to 128,000 edges. We tuned the random data generation to approximate the following characteristics of our main case study: median node degree, median number of phosphosites per node, and median number of time points that exhibit a statistically significant change from the baseline. Both the number of phosphosites per node and the number of significant changes per time course were sampled from Poisson distributions.

Visualization
Network figures were created with Cytoscape version 3.2.0 (Shannon et al., 2003). Most other images were generated using the matplotlib (Hunter, 2007), matplotlib-venn, py-upset, and Seaborn Python libraries. The py-upset package (https://github.com/ImSoErgodic/py-upset) is a Python port of the UpSet technique (Lex et al., 2014).

Supplemental References

Anchang, B., Sadeh, M.J., Jacob, J., Tresch, A., Vlad, M.O., Oefner, P.J., and Spang, R. (2009). Modeling the Temporal Interplay of Molecular Signaling and Gene Expression by Using Dynamic Nested Effects Models. Proc. Natl. Acad. Sci. *106*, 6447–6452.

Cao, M., Pietras, C.M., Feng, X., Doroschak, K.J., Schaffner, T., Park, J., Zhang, H., Cowen, L.J., and Hescott, B.J. (2014). New directions for diffusion-based network prediction of protein function: incorporating pathways with confidence. Bioinformatics *30*, i219–i227.

Ceol, A., Chatr-aryamontri, A., Licata, L., Peluso, D., Briganti, L., Perfetto, L., Castagnoli, L., and Cesareni, G. (2010). MINT, the molecular interaction database: 2009 update. Nucleic Acids Res. *38*, D532–D539.

De Smet, R., and Marchal, K. (2010). Advantages and limitations of current network inference methods. Nat. Rev. Microbiol. *8*, 717–729.

Fisher, J., and Piterman, N. (2014). Model Checking in Biology. In A Systems Theoretic Approach to Systems and Synthetic Biology I: Models and System Characterizations, V.V. Kulkarni, G.-B. Stan, and K. Raman, eds. (Springer Netherlands), pp. 255–279.

Fröhlich, H., Sahin, Ö., Arlt, D., Bender, C., and Beißbarth, T. (2009). Deterministic Effects Propagation Networks for reconstructing protein signaling networks from multiple interventions. BMC Bioinformatics *10*, 322.

Gitter, A., Carmi, M., Barkai, N., and Bar-Joseph, Z. (2013). Linking the signaling cascades and dynamic regulatory networks controlling stress responses. Genome Res. *23*, 365–376.

Henriques, D., Villaverde, A.F., Rocha, M., Saez-Rodriguez, J., and Banga, J.R. (2017). Data-driven reverse engineering of signaling pathways using ensembles of dynamic models. PLOS Comput. Biol. *13*, e1005379.

Hunter, J.D. (2007). Matplotlib: A 2D Graphics Environment. Comput. Sci. Eng. *9*, 90–95.

Iorio, F., Bernardo-Faura, M., Gobbi, A., Cokelaer, T., Jurman, G., and Saez-Rodriguez, J. (2016). Efficient randomization of biological networks while preserving functional characterization of individual nodes. BMC Bioinformatics *17*, 542.

Jo, K., Jung, I., Moon, J.H., and Kim, S. (2016). Influence maximization in time bounded network identifies transcription factors regulating perturbed pathways. Bioinformatics *32*, i128–i136.

Khodaverdian, A., Weitz, B., Wu, J., and Yosef, N. (2016). Steiner Network Problems on Temporal Graphs. arXiv:1609.04918 [cs].

Kiani, N.A., and Kaderali, L. (2014). Dynamic probabilistic threshold networks to infer signaling pathways from time-course perturbation data. BMC Bioinformatics *15*, 250.

Krishnaswamy, S., Spitzer, M.H., Mingueneau, M., Bendall, S.C., Litvin, O., Stone, E., Pe'er, D., and Nolan, G.P. (2014). Conditional density-based analysis of T cell signaling in single-cell data. Science *346*, 1250689.

Leek, J.T., Monsen, E., Dabney, A.R., and Storey, J.D. (2006). EDGE: extraction and analysis of differential gene expression. Bioinformatics *22*, 507–508.

Lees, J.G., Heriche, J.K., Morilla, I., Ranea, J.A., and Orengo, C.A. (2011). Systematic computational prediction of protein interaction networks. Phys. Biol. *8*, 35008.

Lex, A., Gehlenborg, N., Strobelt, H., Vuillemot, R., and Pfister, H. (2014). UpSet: Visualization of Intersecting Sets. IEEE Trans. Vis. Comput. Graph. *20*, 1983–1992.

Markowetz, F., Kostka, D., Troyanskaya, O.G., and Spang, R. (2007). Nested Effects Models for High-Dimensional Phenotyping Screens. Bioinformatics *23*, i305–i312.

Masnadi-Shirazi, M., Maurya, M.R., and Subramaniam, S. (2014). Time-Varying Causal Inference From Phosphoproteomic Measurements in Macrophage Cells. IEEE Trans. Biomed. Circuits Syst. *8*, 74–86.

Morris, M.K., Saez-Rodriguez, J., Clarke, D.C., Sorger, P.K., and Lauffenburger, D.A. (2011). Training Signaling Pathway Maps to Biochemical Data with Constrained Fuzzy Logic: Quantitative Analysis of Liver Cell Responses to Inflammatory Stimuli. PLoS Comput. Biol. *7*, e1001099.

Mosca, R., Pons, T., Céol, A., Valencia, A., and Aloy, P. (2013). Towards a detailed atlas of protein–protein interactions. Curr. Opin. Struct. Biol. *23*, 929–940.

Oda, K., Matsuoka, Y., Funahashi, A., and Kitano, H. (2005). A comprehensive pathway map of epidermal growth factor receptor signaling. Mol. Syst. Biol. *1*, 2005.0010.

Ourfali, O., Shlomi, T., Ideker, T., Ruppin, E., and Sharan, R. (2007). SPINE: a framework for signaling-regulatory pathway inference from cause-effect experiments. Bioinformatics *23*, i359–i366.

Park, Y., and Bader, J.S. (2012). How networks change with time. Bioinformatics *28*, i40–i48.

Patil, A., and Nakai, K. (2014). TimeXNet: Identifying active gene sub-networks using time-course gene expression profiles. BMC Syst. Biol. *8*, S2.

Przytycka, T.M., Singh, M., and Slonim, D.K. (2010). Toward the dynamic interactome: it's about time. Brief. Bioinform. *11*, 15–29.

Ptacek, J., Devgan, G., Michaud, G., Zhu, H., Zhu, X., Fasolo, J., Guo, H., Jona, G., Breitkreutz, A., Sopko, R., et al. (2005). Global analysis of protein phosphorylation in yeast. Nature *438*, 679–684.

Ritz, A., Poirel, C.L., Tegge, A.N., Sharp, N., Simmons, K., Powell, A., Kale, S.D., and Murali, T. (2016). Pathways on demand: automated reconstruction of human signaling networks. Npj Syst. Biol. Appl. *2*, 16002.

Rudolph, J.D., Graauw, M. de, Water, B. van de, Geiger, T., and Sharan, R. (2016). Elucidation of Signaling Pathways from Large-Scale Phosphoproteomic Data Using Protein Interaction Networks. Cell Syst. *3*, 585–593.e3.

Silverbush, D., and Sharan, R. (2014). Network orientation via shortest paths. Bioinformatics *30*, 1449–1455.

Storey, J.D., and Tibshirani, R. (2003). Statistical significance for genomewide studies. Proc. Natl. Acad. Sci. *100*, 9440–9445.

Villaveces, J.M., Jiménez, R.C., Porras, P., del-Toro, N., Duesbury, M., Dumousseau, M., Orchard, S., Choi, H., Ping, P., Zong, N.C., et al. (2015). Merging and scoring molecular interactions utilising existing community standards: tools, use-cases and a case study. Database *2015*, bau131.

Vinayagam, A., Stelzl, U., Foulle, R., Plassmann, S., Zenkner, M., Timm, J., Assmus, H.E., Andrade-Navarro, M.A., and Wanker, E.E. (2011). A Directed Protein Interaction Network for Investigating Intracellular Signal Transduction. Sci. Signal. *4*, rs8.

Vizcaíno, J.A., Csordas, A., del-Toro, N., Dianes, J.A., Griss, J., Lavidas, I., Mayer, G., Perez-Riverol, Y., Reisinger, F., Ternent, T., et al. (2016). 2016 update of the PRIDE database and its related tools. Nucleic Acids Res. *44*, D447–D456.

Wagner, J.P., Wolf-Yadlin, A., Sevecka, M., Grenier, J.K., Root, D.E., Lauffenburger, D.A., and MacBeath, G. (2013). Receptor Tyrosine Kinases Fall into Distinct Classes Based on Their Inferred Signaling Networks. Sci. Signal. *6*, ra58-ra58.

Wang, X., Yuan, K., Hellmayr, C., Liu, W., and Markowetz, F. (2014). Reconstructing evolving signalling networks by hidden Markov nested effects models. Ann. Appl. Stat. *8*, 448–480.

Yeang, C.-H., Ideker, T., and Jaakkola, T. (2004). Physical Network Models. J. Comput. Biol. *11*, 243–262.

Yosef, N., Ungar, L., Zalckvar, E., Kimchi, A., Kupiec, M., Ruppin, E., and Sharan, R. (2009). Toward accurate reconstruction of functional protein networks. Mol. Syst. Biol. *5*.

Zhang, Y., Wolf-Yadlin, A., Ross, P.L., Pappin, D.J., Rush, J., Lauffenburger, D.A., and White, F.M. (2005). Time-resolved Mass Spectrometry of Tyrosine Phosphorylation Sites in the Epidermal Growth Factor Receptor Signaling Network Reveals Dynamic Modules. Mol. Cell. Proteomics *4*, 1240–1250.

Zoppoli, P., Morganella, S., and Ceccarelli, M. (2010). TimeDelay-ARACNE: Reverse engineering of gene networks from time-course data by an information theoretic approach. BMC Bioinformatics *11*, 154.
