# Supplementary material for: Synthesizing Signaling Pathways from Temporal Phosphoproteomic Data
Source: Cell Rep. Author manuscript; Available in PMC 2018 Dec 16. (PMC6295338; doi:10.1016/j.celrep.2018.08.085)
Supplement: 5 [file NIHMS1508952-supplement-5.zip › directed_evaluation.html]

Evaluate TPS pathway directed edges


# Analysis of TPS directed predictions

## Statistics

Number of predictions with unambiguous direction:
202
(48.91%)
  

## Color legend

- Exact prediction match
- Approximate prediction match
- Imprecise prediction
- Partial conflict
- Total conflict

## Edges

### EGF\_HUMAN → EGFR\_HUMAN / EGF\_HUMAN ⊣ EGFR\_HUMAN

#### 1. Resources that have the edge

- science-signaling-egfr
  [+]

  Resource paths:
  - EGF {EGF\_HUMAN} (Protein) → EGFR {EGFR\_HUMAN} (Protein) [ProteinProteinInteraction (activation)]
- kegg-erbb
  [+]

  Resource paths:
  - rnC8\_EGF,\_HOMG4,\_URG { HOMG4, EGF,HOMG4,URG, URG, EGF, HOMG4, URG, URG, EGF, HOMG4} (Protein) → rnC45\_EGFR,\_ERBB,\_ERBB1,\_HER1,\_PIG61,\_mENA { PIG61, PIG61, HER1, ERBB1, ERBB1, mENA, HER1, ERBB, EGFR,ERBB,ERBB1,HER1,PIG61,mENA, ERBB, mENA, EGFR, ERBB, ERBB1, HER1, PIG61, mENA, EGFR} (Protein) [Catalysis (activation)]
  - EGF\_HUMAN and HOMG4 are synonyms.
  - EGFR\_HUMAN and PIG61 are synonyms.
- reactome-egfr
  [+]

  Resource has common complex(es):
  - Complex3679 {EGF:Phospho-EGFR (Y1045) dimer:CBL:Phospho-Sprouty ubiquitinated, EGF:p-6Y-EGFR:CBL:Ub-p-Y53/55-SPRY1/2} (Complex)
  - Complex3700 {EGF:Phospho-EGFR dimer:CBL:Phospho-Sprouty, EGF:p-6Y-EGFR:CBL:p-Y53/55-SPRY1/2} (Complex)
  - Complex1025 {EGF:Phospho-EGFR} (Complex)
  - Complex3673 {EGF:Phospho-EGFR (-Y992) dimer} (Complex)
  - Complex3698 {EGF:Phospho-EGFR (Y1045) dimer:Phospho-CBL:GRB2:CIN85:Endophilin:Epsin:Eps15R:Eps15 complex:Clathrin, EGF:p-6Y-EGFR:p-Y371-CBL:GRB2:CIN85:Endophilin:Epsin:Eps15R:Eps15:Clathrin} (Complex)
  - Complex3699 {EGF:Phospho-EGFR (Y1045) dimer:Phospho-CBL:CIN85:Endophilin:Epsin:Eps15R:Eps15 complex, EGF:p-6Y-EGFR:p-Y371-CBL:CIN85:Endophilin:Epsin:Eps15R:Eps15} (Complex)
  - Complex3666 {GAB1:GRB2-EGF-Phospho-EGFR dimer, EGF:p-6Y-EGFR:GRB2:GAB1} (Complex)
  - Complex3685 {EGF:Phospho-EGFR (Y1045) dimer Ubiquitinated:Phospho-CBL:GRB2, EGF:Ub-p-6Y-EGFR:p-Y371-CBL:GRB2} (Complex)
  - Complex3656 {EGF:EGFR} (Complex)
  - Complex3703 {EGF:Phospho-EGFR (Y1045) dimer:Phospho-CBL:CIN85-Sprouty:Endophilin:Epsin:Eps15R:Eps15 complex, EGF:p-6Y-EGFR:p-Y371-CBL:CIN85:SPRY1/2:Endophilin:Epsin:Eps15R:Eps15} (Complex)
  - Complex1023 {EGF:Phospho-EGFR-GRB2:GAB1:PI3Kreg:PI3Kcat, EGF:p-6Y-EGFR:GRB2:GAB1:PIK3} (Complex)
  - Complex3679 {EGF:Phospho-EGFR (Y1045) dimer:CBL:Phospho-Sprouty ubiquitinated, EGF:p-6Y-EGFR:CBL:Ub-p-Y53/55-SPRY1/2} (Complex)
  - Complex3679 {EGF:Phospho-EGFR (Y1045) dimer:CBL:Phospho-Sprouty ubiquitinated, EGF:p-6Y-EGFR:CBL:Ub-p-Y53/55-SPRY1/2} (Complex)
  - Complex3703 {EGF:Phospho-EGFR (Y1045) dimer:Phospho-CBL:CIN85-Sprouty:Endophilin:Epsin:Eps15R:Eps15 complex, EGF:p-6Y-EGFR:p-Y371-CBL:CIN85:SPRY1/2:Endophilin:Epsin:Eps15R:Eps15} (Complex)
  - Complex3698 {EGF:Phospho-EGFR (Y1045) dimer:Phospho-CBL:GRB2:CIN85:Endophilin:Epsin:Eps15R:Eps15 complex:Clathrin, EGF:p-6Y-EGFR:p-Y371-CBL:GRB2:CIN85:Endophilin:Epsin:Eps15R:Eps15:Clathrin} (Complex)
  - Complex3679 {EGF:Phospho-EGFR (Y1045) dimer:CBL:Phospho-Sprouty ubiquitinated, EGF:p-6Y-EGFR:CBL:Ub-p-Y53/55-SPRY1/2} (Complex)
  - Complex3659 {EGF:p-6Y-EGFR:p-Y472,771,783,1254-PLCG1, Activated EGFR:Phospho-PLC-gamma1} (Complex)
  - Complex3693 {EGF:Phospho-EGFR (Y1045) dimer:CBL:Cool/Pix:CDC42-GTP, EGF:p-6Y-EGFR:CBL:Beta-Pix:CDC42:GTP} (Complex)
  - Complex3679 {EGF:Phospho-EGFR (Y1045) dimer:CBL:Phospho-Sprouty ubiquitinated, EGF:p-6Y-EGFR:CBL:Ub-p-Y53/55-SPRY1/2} (Complex)
  - Complex3691 {EGF:Phospho-EGFR (Y1045) dimer:CBL:CIN85, EGF:p-6Y-EGFR:CBL:CIN85} (Complex)
  - Complex3657 {EGF:EGFR dimer} (Complex)
  - Complex3700 {EGF:Phospho-EGFR dimer:CBL:Phospho-Sprouty, EGF:p-6Y-EGFR:CBL:p-Y53/55-SPRY1/2} (Complex)
  - Complex3678 {EGF:Phospho-EGFR (Y1045) dimer:Phospho-CBL, EGF:p-6Y-EGFR:p-Y371-CBL} (Complex)
  - Complex3674 {EGF:Phospho-EGFR (- Y992)} (Complex)
  - Complex3681 {EGF:Phospho-EGFR (Y1045) dimer Ubiquitinated:Phospho-CBL, EGF:Ub-p-6Y-EGFR:p-Y371-CBL} (Complex)
  - Complex3681 {EGF:Phospho-EGFR (Y1045) dimer Ubiquitinated:Phospho-CBL, EGF:Ub-p-6Y-EGFR:p-Y371-CBL} (Complex)
  - Complex3701 {EGF:Phospho-EGFR (Y1045) dimer:Phospho-CBL:CIN85 ubiquitinated:Endophilin:Epsin:Eps15R:Eps15 complex, EGF:p-6Y-EGFR:p-Y371-CBL:Ub-CIN85:Endophilin:Epsin:Eps15R:Eps15} (Complex)
  - Complex3658 {Activated EGFR:PLC-gamma1, EGF:p-6Y-EGFR:PLCG1} (Complex)
  - Complex3698 {EGF:Phospho-EGFR (Y1045) dimer:Phospho-CBL:GRB2:CIN85:Endophilin:Epsin:Eps15R:Eps15 complex:Clathrin, EGF:p-6Y-EGFR:p-Y371-CBL:GRB2:CIN85:Endophilin:Epsin:Eps15R:Eps15:Clathrin} (Complex)
  - Complex3662 {EGF:p-6Y-EGFR:p-Y349,350-SHC1, EGF:Phospho-EGFR:Phospho-SHC} (Complex)
  - Complex1024 {EGF:Phospho-EGFR (Y992, Y1068, Y1086, Y1148, Y1173) dimer, EGF:p-6Y-EGFR} (Complex)
  - Complex3684 {EGF:Phospho-EGFR (Y1045) dimer:Phospho-CBL:GRB2, EGF:p-6Y-EGFR:p-Y371-CBL:GRB2} (Complex)
  - Complex3685 {EGF:Phospho-EGFR (Y1045) dimer Ubiquitinated:Phospho-CBL:GRB2, EGF:Ub-p-6Y-EGFR:p-Y371-CBL:GRB2} (Complex)
  - Complex3685 {EGF:Phospho-EGFR (Y1045) dimer Ubiquitinated:Phospho-CBL:GRB2, EGF:Ub-p-6Y-EGFR:p-Y371-CBL:GRB2} (Complex)
  - Complex3677 {EGF:Phospho-EGFR (Y1045) dimer:CBL, EGF:p-6Y-EGFR:CBL} (Complex)
  - Complex3681 {EGF:Phospho-EGFR (Y1045) dimer Ubiquitinated:Phospho-CBL, EGF:Ub-p-6Y-EGFR:p-Y371-CBL} (Complex)
  - Complex3661 {EGF:Phospho-EGFR-SHC, EGF:p-6Y-EGFR:SHC1} (Complex)
  - Complex3670 {SHP2-GRB2:Phospho GAB1(dephos)-EGF-Phospho-EGFR dimer, EGF:p-6Y-EGFR:GRB2:p-Y627,659-GAB1:SHP2} (Complex)
  - Complex3679 {EGF:Phospho-EGFR (Y1045) dimer:CBL:Phospho-Sprouty ubiquitinated, EGF:p-6Y-EGFR:CBL:Ub-p-Y53/55-SPRY1/2} (Complex)
  - Complex3660 {GRB2:SOS-EGF-Phospho-EGFR dimer, EGF:p-6Y-EGFR:GRB2:SOS1} (Complex)
  - Complex3696 {EGF:Phospho-EGFR (Y1045) dimer:Phospho-CBL:GRB2:CIN85:Endophilin, EGF:p-6Y-EGFR:p-Y371-CBL:GRB2:CIN85:Endophilin} (Complex)
  - Complex3672 {SHP2:GRB2:Phospho GAB1-EGF-Phospho-EGFR (-Y992) dimer, EGF:p-5Y-EGFR:GRB2:p-5Y-GAB1:SHP2} (Complex)
  - Complex3663 {GRB2:SOS-Phospho-SHC:EGF:Phospho-EGFR dimer, EGF:p-6Y-EGFR:p-Y349,350-SHC1:GRB2:SOS1} (Complex)
  - Complex3667 {GRB2:Phospho GAB1-EGF-Phospho-EGFR dimer, EGF:p-6Y-EGFR:GRB2:p-5Y-GAB1} (Complex)
  - Complex3669 {SHP2-GRB2:Phospho GAB1-EGF-Phospho-EGFR dimer, EGF:p-6Y-EGFR:GRB2:p-5Y-GAB1:SHP2} (Complex)
  - Complex3683 {EGF:Phospho-EGFR (Y1045) dimer:CBL:GRB2, EGF:p-6Y-EGFR:CBL:GRB2} (Complex)
  - Complex3664 {EGF:Phospho-EGFR-GRB2:GAB1:PI3Kreg, EGF:p-6Y-EGFR:GRB2:GAB1:PIK3R1} (Complex)
  - EGF\_HUMAN and UniProt:P01133 EGF are synonyms.
  - EGFR\_HUMAN and UniProt:P00533 EGFR are synonyms.
- pid-erbb1
  [+]

  Resource has common complex(es):
  - pid\_47681 {EGFR/EGFR/EGF/EGF/JAK1-JAK2} (Complex)
  - pid\_48005 {EGFR/EGFR/EGF/EGF} (Complex)
  - pid\_47909 {EGFR/EGFR/EGF/EGF} (Complex)
  - pid\_47873 {EGFR/EGFR/EGF/EGF/PLCgamma1} (Complex)
  - pid\_47747 {EGFR/EGFR/EGF/EGF/GNAI1/GNAI3/GAB1} (Complex)
  - pid\_40369 {EGFR/EGFR/EGF/EGF/GRB2/GAB1} (Complex)
  - pid\_47843 {EGFR/EGFR/EGF/EGF/GRB2/PAK1} (Complex)
  - pid\_26325 {EGFR/EGFR/EGF/EGF} (Complex)
  - pid\_47490 {EGFR/EGFR/EGF/EGF/p52 SHC/GRB2/SOS1} (Complex)
  - pid\_47530 {EGFR/EGFR/EGF/EGF/GRB2} (Complex)
  - pid\_47659 {EGFR/EGFR/EGF/EGF/p46 SHC} (Complex)
  - pid\_47593 {EGFR/EGFR/EGF/EGF/NCK1-2/PAK1} (Complex)
  - pid\_47975 {EGFR/EGFR/EGF/EGF/p66 SHC} (Complex)
  - pid\_47989 {EGFR/EGFR/EGF/EGF} (Complex)
  - pid\_47681 {EGFR/EGFR/EGF/EGF/JAK1-JAK2} (Complex)
  - pid\_47648 {EGFR/EGFR/EGF/EGF/p46 SHC/GRB2/SOS1} (Complex)
  - pid\_47471 {EGFR/EGFR/EGF/EGF/GNAI1/GNAI3/GAB1} (Complex)
  - pid\_48019 {EGFR/EGFR/EGF/EGF/GRB2/GAB1/RasGAP} (Complex)
  - pid\_47567 {EGFR/EGFR/EGF/EGF} (Complex)
  - pid\_47882 {EGFR/EGFR/EGF/EGF/GNAI1/GNAI3/GAB1} (Complex)
  - pid\_48074 {EGFR/EGFR/EGF/EGF/p52 SHC} (Complex)
  - pid\_40050 {EGFR/EGFR/EGF/EGF} (Complex)
  - pid\_47609 {EGFR/EGFR/EGF/EGF/GRB2/GAB1} (Complex)
  - pid\_47593 {EGFR/EGFR/EGF/EGF/NCK1-2/PAK1} (Complex)
  - pid\_47506 {EGFR/EGFR/EGF/EGF} (Complex)
  - pid\_47951 {EGFR/EGFR/EGF/EGF/p46 SHC} (Complex)
  - pid\_47633 {EGFR/EGFR/EGF/EGF/GNAI1/GNAI3/GAB1/RasGAP} (Complex)
  - pid\_48039 {EGFR/EGFR/EGF/EGF/GNAI1/GNAI3/GAB1/RasGAP} (Complex)
  - pid\_47561 {EGFR/EGFR/EGF/EGF/GRB2/SOS1} (Complex)
  - pid\_47826 {EGFR/EGFR/EGF/EGF/p52 SHC} (Complex)
  - pid\_47699 {EGFR/EGFR/EGF/EGF/GRB2/N-WASP} (Complex)
  - pid\_47798 {EGFR/EGFR/EGF/EGF/FAK} (Complex)
  - pid\_47712 {EGFR/EGFR/EGF/EGF/p52 SHC} (Complex)
  - pid\_47626 {EGFR/EGFR/EGF/EGF/GNAI1/GNAI3/GAB1} (Complex)
  - pid\_47932 {EGFR/EGFR/EGF/EGF/p46 SHC} (Complex)
  - pid\_47787 {EGFR/EGFR/EGF/EGF/RasGAP} (Complex)
  - EGF\_HUMAN and EGF are synonyms.
  - EGFR\_HUMAN and EGFR are synonyms.
- layek
  [+]

  Resource paths:
  - EGF {EGF\_HUMAN} (Protein) → EGFR {EGFR\_HUMAN} (Protein) [ProteinProteinInteraction (activation)]
- kegg-mapk
  [+]

  Resource paths:
  - rnC4\_EGF,\_HOMG4,\_URG { HOMG4, EGF,HOMG4,URG, URG, EGF, HOMG4, URG, URG, EGF, HOMG4} (Protein) → rnC22\_EGFR,\_ERBB,\_ERBB1,\_HER1,\_PIG61,\_mENA { PIG61, PIG61, HER1, ERBB1, ERBB1, mENA, HER1, ERBB, EGFR,ERBB,ERBB1,HER1,PIG61,mENA, ERBB, mENA, EGFR, ERBB, ERBB1, HER1, PIG61, mENA, EGFR} (Protein) [Catalysis (activation)]
  - EGF\_HUMAN and HOMG4 are synonyms.
  - EGFR\_HUMAN and PIG61 are synonyms.
- biocarta-egf
  [+]

  Resource has common complex(es):
  - pid\_7077 {EGF/EGF-R} (Complex)
  - EGF\_HUMAN and EGF are synonyms.
  - EGFR\_HUMAN and EGFR are synonyms.
- cancer-cell-map-egfr1
  [+]

  Resource has common complex(es):
  - physicalInteraction2841 {Epidermal growth factor/ErbB1/Epidermal growth factor receptor/Beta-Urogastrone/HER1/URG/EGF/Urogastrone/EGFR} (Complex)
  - physicalInteraction3012 {Epidermal growth factor/ErbB1/Epidermal growth factor receptor/Beta-Urogastrone/HER1/URG/EGF/Urogastrone/EGFR} (Complex)
  - EGF\_HUMAN and URG are synonyms.
  - EGFR\_HUMAN and HER1 are synonyms.

#### 2. Resources that contain endpoints (but not an edge)


#### 3. Resources that match only one endpoint (fringe)

- phosphositeplus-kinase-substrate
  [+]

  No paths found.
  - EGF\_HUMAN was not found.

#### 4. Resources that don't match any endpoint


### MP2K1\_HUMAN → MK03\_HUMAN / MP2K1\_HUMAN ⊣ MK03\_HUMAN

#### 1. Resources that have the edge

- phosphositeplus-kinase-substrate
  [+]

  Resource paths:
  - Q02750 {MP2K1\_HUMAN} (Protein) → P27361 {MK03\_HUMAN} (Protein) [ProteinProteinInteraction (unknown sign)]
  - P27361 {MK03\_HUMAN} (Protein) → Q02750 {MP2K1\_HUMAN} (Protein) [ProteinProteinInteraction (unknown sign)]
- cancer-cell-map-egfr1
  [+]

  Resource paths:
  - sequenceParticipant886 {MAPK/ERK kinase, MAP kinase kinase 1, MEK1, MAP2K1, MKK1, MAPKK1, Dual specificity mitogen-activated protein kinase kinase 1, Mitogen activated protein kinase kinase 1, ERK activator kinase 1, PRKMK1} (Protein) → sequenceParticipant315 {Insulin stimulated MAP2 kinase, Microtubule associated protein 2 kinase, MAPK1, PRKM3, P44MAPK, MAP kinase1, P44ERK1, ERK1, Protein kinase, mitogen activated, 3, MAPK3, ERT2, Mitogen activated protein kinase3, Extracellular signal regulated kinase1} (Protein) [Catalysis (unknown sign)]
  - MP2K1\_HUMAN and MEK1 are synonyms.
  - MK03\_HUMAN and PRKM3 are synonyms.
- reactome-egfr
  [+]

  Resource has common complex(es):
  - Complex1304 {phospho-ERK-1:MEK1} (Complex)
  - Complex1303 {MEK1:ERK-1} (Complex)
  - MP2K1\_HUMAN and MEK1 are synonyms.
  - MK03\_HUMAN and PRKM3 are synonyms.
- science-signaling-egfr
  [+]

  Resource paths:
  - MAPKK {MP2K1\_HUMAN} (Protein) → MAPK (ERK) {MK03\_HUMAN} (Protein) [ProteinProteinInteraction (activation)]
- layek
  [+]

  Resource paths:
  - MEK1 {MP2K1\_HUMAN} (Protein) → ERK1 {MK03\_HUMAN} (Protein) [ProteinProteinInteraction (activation)]

#### 2. Resources that contain endpoints (but not an edge)


#### 3. Resources that match only one endpoint (fringe)

- kegg-mapk
  [+]

  No paths found.
  - MK03\_HUMAN was not found.
- kegg-erbb
  [+]

  No paths found.
  - MK03\_HUMAN was not found.

#### 4. Resources that don't match any endpoint

- pid-erbb1
  [+]

  No paths found.
  - MP2K1\_HUMAN was not found.
  - MK03\_HUMAN was not found.
- biocarta-egf
  [+]

  No paths found.
  - MP2K1\_HUMAN was not found.
  - MK03\_HUMAN was not found.

### GRB2\_HUMAN → PTN11\_HUMAN

#### 1. Resources that have the edge

- cancer-cell-map-egfr1
  [+]

  Resource has common complex(es):
  - physicalInteraction3002 {SHP2/SHPTP2/PTP-1D/SHPTP3/Growth factor receptor bound protein 2/Protein tyrosine phosphatase, non-receptor type 11/abundant SRC homology/ASH/GRB2/PTPN11} (Complex)
  - PTN11\_HUMAN and SHP2 are synonyms.
  - GRB2\_HUMAN and ASH are synonyms.
- pid-erbb1
  [+]

  Resource paths:
  - pid\_40369 {EGFR/EGFR/EGF/EGF/GRB2/GAB1} (Complex) → pid\_47514 {SHP2} (Protein) [Catalysis (activation)]
  - PTN11\_HUMAN and SHP2 are synonyms.
  - GRB2\_HUMAN and Grb2 are synonyms.
- reactome-egfr
  [+]

  Resource has common complex(es):
  - Complex3670 {SHP2-GRB2:Phospho GAB1(dephos)-EGF-Phospho-EGFR dimer, EGF:p-6Y-EGFR:GRB2:p-Y627,659-GAB1:SHP2} (Complex)
  - Complex3672 {SHP2:GRB2:Phospho GAB1-EGF-Phospho-EGFR (-Y992) dimer, EGF:p-5Y-EGFR:GRB2:p-5Y-GAB1:SHP2} (Complex)
  - Complex3669 {SHP2-GRB2:Phospho GAB1-EGF-Phospho-EGFR dimer, EGF:p-6Y-EGFR:GRB2:p-5Y-GAB1:SHP2} (Complex)
  - PTN11\_HUMAN and UniProt:Q06124 PTPN11 are synonyms.
  - GRB2\_HUMAN and ASH are synonyms.

#### 2. Resources that contain endpoints (but not an edge)

- phosphositeplus-kinase-substrate
  [+]

  No paths found.

#### 3. Resources that match only one endpoint (fringe)

- layek
  [+]

  No paths found.
  - PTN11\_HUMAN was not found.
- biocarta-egf
  [+]

  No paths found.
  - PTN11\_HUMAN was not found.
- kegg-erbb
  [+]

  No paths found.
  - PTN11\_HUMAN was not found.
- kegg-mapk
  [+]

  No paths found.
  - PTN11\_HUMAN was not found.
- science-signaling-egfr
  [+]

  No paths found.
  - PTN11\_HUMAN was not found.

#### 4. Resources that don't match any endpoint


### EGFR\_HUMAN ⊣ FAK1\_HUMAN / EGFR\_HUMAN → FAK1\_HUMAN

#### 1. Resources that have the edge

- phosphositeplus-kinase-substrate
  [+]

  Resource paths:
  - P00533 {EGFR\_HUMAN} (Protein) → Q05397 {FAK1\_HUMAN} (Protein) [ProteinProteinInteraction (unknown sign)]
- pid-erbb1
  [+]

  Resource has common complex(es):
  - pid\_47798 {EGFR/EGFR/EGF/EGF/FAK} (Complex)
  - EGFR\_HUMAN and EGFR are synonyms.
  - FAK1\_HUMAN and FAK are synonyms.

#### 2. Resources that contain endpoints (but not an edge)

- kegg-erbb
  [+]

  No paths found.

#### 3. Resources that match only one endpoint (fringe)

- kegg-mapk
  [+]

  No paths found.
  - FAK1\_HUMAN was not found.
- layek
  [+]

  No paths found.
  - FAK1\_HUMAN was not found.
- biocarta-egf
  [+]

  No paths found.
  - FAK1\_HUMAN was not found.
- cancer-cell-map-egfr1
  [+]

  No paths found.
  - FAK1\_HUMAN was not found.
- science-signaling-egfr
  [+]

  No paths found.
  - FAK1\_HUMAN was not found.
- reactome-egfr
  [+]

  No paths found.
  - FAK1\_HUMAN was not found.

#### 4. Resources that don't match any endpoint


### PAK1\_HUMAN → MP2K1\_HUMAN / PAK1\_HUMAN ⊣ MP2K1\_HUMAN

#### 1. Resources that have the edge

- phosphositeplus-kinase-substrate
  [+]

  Resource paths:
  - Q13153 {PAK1\_HUMAN} (Protein) → Q02750 {MP2K1\_HUMAN} (Protein) [ProteinProteinInteraction (unknown sign)]
- cancer-cell-map-egfr1
  [+]

  Resource paths:
  - sequenceParticipant1012 {Alpha-PAK, Serine/threonine protein kinase PAK1, PAK1, P65-PAK, PAKalpha, p21/Cdc42/Rac1 activated kinase1, p21 activated kinase1} (Protein) → sequenceParticipant312 {MAPK/ERK kinase, MAP kinase kinase 1, MEK1, MAP2K1, MKK1, MAPKK1, Dual specificity mitogen-activated protein kinase kinase 1, Mitogen activated protein kinase kinase 1, ERK activator kinase 1, PRKMK1} (Protein) [Catalysis (unknown sign)]
  - PAK1\_HUMAN and PAK1 are synonyms.
  - MP2K1\_HUMAN and MEK1 are synonyms.

#### 2. Resources that contain endpoints (but not an edge)


#### 3. Resources that match only one endpoint (fringe)

- kegg-mapk
  [+]

  No paths found.
  - PAK1\_HUMAN was not found.
- reactome-egfr
  [+]

  No paths found.
  - PAK1\_HUMAN was not found.
- kegg-erbb
  [+]

  No paths found.
  - PAK1\_HUMAN was not found.
- science-signaling-egfr
  [+]

  No paths found.
  - PAK1\_HUMAN was not found.
- pid-erbb1
  [+]

  No paths found.
  - MP2K1\_HUMAN was not found.
- layek
  [+]

  No paths found.
  - PAK1\_HUMAN was not found.

#### 4. Resources that don't match any endpoint

- biocarta-egf
  [+]

  No paths found.
  - PAK1\_HUMAN was not found.
  - MP2K1\_HUMAN was not found.

### GRB2\_HUMAN ⊣ WASL\_HUMAN

#### 1. Resources that have the edge

- pid-erbb1
  [+]

  Resource has common complex(es):
  - pid\_47942 {GRB2/N-WASP} (Complex)
  - pid\_47699 {EGFR/EGFR/EGF/EGF/GRB2/N-WASP} (Complex)
  - GRB2\_HUMAN and Grb2 are synonyms.
  - WASL\_HUMAN and N-WASP are synonyms.

#### 2. Resources that contain endpoints (but not an edge)

- science-signaling-egfr
  [+]

  No paths found.

#### 3. Resources that match only one endpoint (fringe)

- biocarta-egf
  [+]

  No paths found.
  - WASL\_HUMAN was not found.
- phosphositeplus-kinase-substrate
  [+]

  No paths found.
  - WASL\_HUMAN was not found.
- kegg-erbb
  [+]

  No paths found.
  - WASL\_HUMAN was not found.
- reactome-egfr
  [+]

  No paths found.
  - WASL\_HUMAN was not found.
- cancer-cell-map-egfr1
  [+]

  No paths found.
  - WASL\_HUMAN was not found.
- kegg-mapk
  [+]

  No paths found.
  - WASL\_HUMAN was not found.
- layek
  [+]

  No paths found.
  - WASL\_HUMAN was not found.

#### 4. Resources that don't match any endpoint


### MK01\_HUMAN ⊣ ABI1\_HUMAN

#### 1. Resources that have the edge

- phosphositeplus-kinase-substrate
  [+]

  Resource paths:
  - P28482 {MK01\_HUMAN} (Protein) → Q8IZP0 {ABI1\_HUMAN} (Protein) [ProteinProteinInteraction (unknown sign)]

#### 2. Resources that contain endpoints (but not an edge)

- cancer-cell-map-egfr1
  [+]

  No paths found.

#### 3. Resources that match only one endpoint (fringe)

- kegg-mapk
  [+]

  No paths found.
  - ABI1\_HUMAN was not found.
- reactome-egfr
  [+]

  No paths found.
  - ABI1\_HUMAN was not found.
- science-signaling-egfr
  [+]

  No paths found.
  - ABI1\_HUMAN was not found.
- kegg-erbb
  [+]

  No paths found.
  - ABI1\_HUMAN was not found.
- layek
  [+]

  No paths found.
  - ABI1\_HUMAN was not found.
- pid-erbb1
  [+]

  No paths found.
  - ABI1\_HUMAN was not found.

#### 4. Resources that don't match any endpoint

- biocarta-egf
  [+]

  No paths found.
  - MK01\_HUMAN was not found.
  - ABI1\_HUMAN was not found.

### AKT1\_HUMAN → WNK1\_HUMAN / AKT1\_HUMAN ⊣ WNK1\_HUMAN

#### 1. Resources that have the edge

- phosphositeplus-kinase-substrate
  [+]

  Resource paths:
  - P31749 {AKT1\_HUMAN} (Protein) → Q9H4A3 {WNK1\_HUMAN} (Protein) [ProteinProteinInteraction (unknown sign)]

#### 2. Resources that contain endpoints (but not an edge)

- cancer-cell-map-egfr1
  [+]

  No paths found.

#### 3. Resources that match only one endpoint (fringe)

- layek
  [+]

  No paths found.
  - WNK1\_HUMAN was not found.
- reactome-egfr
  [+]

  No paths found.
  - WNK1\_HUMAN was not found.
- science-signaling-egfr
  [+]

  No paths found.
  - WNK1\_HUMAN was not found.

#### 4. Resources that don't match any endpoint

- kegg-mapk
  [+]

  No paths found.
  - AKT1\_HUMAN was not found.
  - WNK1\_HUMAN was not found.
- kegg-erbb
  [+]

  No paths found.
  - AKT1\_HUMAN was not found.
  - WNK1\_HUMAN was not found.
- pid-erbb1
  [+]

  No paths found.
  - AKT1\_HUMAN was not found.
  - WNK1\_HUMAN was not found.
- biocarta-egf
  [+]

  No paths found.
  - AKT1\_HUMAN was not found.
  - WNK1\_HUMAN was not found.

### EGFR\_HUMAN → CCD50\_HUMAN

#### 1. Resources that have the edge

- phosphositeplus-kinase-substrate
  [+]

  Resource paths:
  - P00533 {EGFR\_HUMAN} (Protein) → Q8IVM0 {CCD50\_HUMAN} (Protein) [ProteinProteinInteraction (unknown sign)]

#### 2. Resources that contain endpoints (but not an edge)


#### 3. Resources that match only one endpoint (fringe)

- cancer-cell-map-egfr1
  [+]

  No paths found.
  - CCD50\_HUMAN was not found.
- kegg-mapk
  [+]

  No paths found.
  - CCD50\_HUMAN was not found.
- reactome-egfr
  [+]

  No paths found.
  - CCD50\_HUMAN was not found.
- kegg-erbb
  [+]

  No paths found.
  - CCD50\_HUMAN was not found.
- layek
  [+]

  No paths found.
  - CCD50\_HUMAN was not found.
- pid-erbb1
  [+]

  No paths found.
  - CCD50\_HUMAN was not found.
- biocarta-egf
  [+]

  No paths found.
  - CCD50\_HUMAN was not found.
- science-signaling-egfr
  [+]

  No paths found.
  - CCD50\_HUMAN was not found.

#### 4. Resources that don't match any endpoint


### MK01\_HUMAN → RUNX1\_HUMAN / MK01\_HUMAN ⊣ RUNX1\_HUMAN

#### 1. Resources that have the edge

- phosphositeplus-kinase-substrate
  [+]

  Resource paths:
  - P28482 {MK01\_HUMAN} (Protein) → Q01196 {RUNX1\_HUMAN} (Protein) [ProteinProteinInteraction (unknown sign)]

#### 2. Resources that contain endpoints (but not an edge)


#### 3. Resources that match only one endpoint (fringe)

- reactome-egfr
  [+]

  No paths found.
  - RUNX1\_HUMAN was not found.
- pid-erbb1
  [+]

  No paths found.
  - RUNX1\_HUMAN was not found.
- science-signaling-egfr
  [+]

  No paths found.
  - RUNX1\_HUMAN was not found.
- layek
  [+]

  No paths found.
  - RUNX1\_HUMAN was not found.
- kegg-mapk
  [+]

  No paths found.
  - RUNX1\_HUMAN was not found.
- kegg-erbb
  [+]

  No paths found.
  - RUNX1\_HUMAN was not found.
- cancer-cell-map-egfr1
  [+]

  No paths found.
  - RUNX1\_HUMAN was not found.

#### 4. Resources that don't match any endpoint

- biocarta-egf
  [+]

  No paths found.
  - MK01\_HUMAN was not found.
  - RUNX1\_HUMAN was not found.

### MK01\_HUMAN → ERF\_HUMAN / MK01\_HUMAN ⊣ ERF\_HUMAN

#### 1. Resources that have the edge

- phosphositeplus-kinase-substrate
  [+]

  Resource paths:
  - P28482 {MK01\_HUMAN} (Protein) → P50548 {ERF\_HUMAN} (Protein) [ProteinProteinInteraction (unknown sign)]

#### 2. Resources that contain endpoints (but not an edge)


#### 3. Resources that match only one endpoint (fringe)

- layek
  [+]

  No paths found.
  - ERF\_HUMAN was not found.
- kegg-mapk
  [+]

  No paths found.
  - ERF\_HUMAN was not found.
- pid-erbb1
  [+]

  No paths found.
  - ERF\_HUMAN was not found.
- science-signaling-egfr
  [+]

  No paths found.
  - ERF\_HUMAN was not found.
- kegg-erbb
  [+]

  No paths found.
  - ERF\_HUMAN was not found.
- reactome-egfr
  [+]

  No paths found.
  - ERF\_HUMAN was not found.
- cancer-cell-map-egfr1
  [+]

  No paths found.
  - ERF\_HUMAN was not found.

#### 4. Resources that don't match any endpoint

- biocarta-egf
  [+]

  No paths found.
  - MK01\_HUMAN was not found.
  - ERF\_HUMAN was not found.

### MK01\_HUMAN → CSK21\_HUMAN / MK01\_HUMAN ⊣ CSK21\_HUMAN

#### 1. Resources that have the edge

- phosphositeplus-kinase-substrate
  [+]

  Resource paths:
  - P28482 {MK01\_HUMAN} (Protein) → P68400 {CSK21\_HUMAN} (Protein) [ProteinProteinInteraction (unknown sign)]

#### 2. Resources that contain endpoints (but not an edge)


#### 3. Resources that match only one endpoint (fringe)

- cancer-cell-map-egfr1
  [+]

  No paths found.
  - CSK21\_HUMAN was not found.
- kegg-mapk
  [+]

  No paths found.
  - CSK21\_HUMAN was not found.
- kegg-erbb
  [+]

  No paths found.
  - CSK21\_HUMAN was not found.
- layek
  [+]

  No paths found.
  - CSK21\_HUMAN was not found.
- science-signaling-egfr
  [+]

  No paths found.
  - CSK21\_HUMAN was not found.
- pid-erbb1
  [+]

  No paths found.
  - CSK21\_HUMAN was not found.
- reactome-egfr
  [+]

  No paths found.
  - CSK21\_HUMAN was not found.

#### 4. Resources that don't match any endpoint

- biocarta-egf
  [+]

  No paths found.
  - MK01\_HUMAN was not found.
  - CSK21\_HUMAN was not found.

### MK01\_HUMAN → C2TA\_HUMAN / MK01\_HUMAN ⊣ C2TA\_HUMAN

#### 1. Resources that have the edge

- phosphositeplus-kinase-substrate
  [+]

  Resource paths:
  - P28482 {MK01\_HUMAN} (Protein) → P33076 {C2TA\_HUMAN} (Protein) [ProteinProteinInteraction (unknown sign)]

#### 2. Resources that contain endpoints (but not an edge)


#### 3. Resources that match only one endpoint (fringe)

- reactome-egfr
  [+]

  No paths found.
  - C2TA\_HUMAN was not found.
- kegg-mapk
  [+]

  No paths found.
  - C2TA\_HUMAN was not found.
- pid-erbb1
  [+]

  No paths found.
  - C2TA\_HUMAN was not found.
- layek
  [+]

  No paths found.
  - C2TA\_HUMAN was not found.
- science-signaling-egfr
  [+]

  No paths found.
  - C2TA\_HUMAN was not found.
- cancer-cell-map-egfr1
  [+]

  No paths found.
  - C2TA\_HUMAN was not found.
- kegg-erbb
  [+]

  No paths found.
  - C2TA\_HUMAN was not found.

#### 4. Resources that don't match any endpoint

- biocarta-egf
  [+]

  No paths found.
  - MK01\_HUMAN was not found.
  - C2TA\_HUMAN was not found.

### MK01\_HUMAN → MKL1\_HUMAN

#### 1. Resources that have the edge

- phosphositeplus-kinase-substrate
  [+]

  Resource paths:
  - P28482 {MK01\_HUMAN} (Protein) → Q969V6 {MKL1\_HUMAN} (Protein) [ProteinProteinInteraction (unknown sign)]

#### 2. Resources that contain endpoints (but not an edge)


#### 3. Resources that match only one endpoint (fringe)

- kegg-mapk
  [+]

  No paths found.
  - MKL1\_HUMAN was not found.
- science-signaling-egfr
  [+]

  No paths found.
  - MKL1\_HUMAN was not found.
- reactome-egfr
  [+]

  No paths found.
  - MKL1\_HUMAN was not found.
- layek
  [+]

  No paths found.
  - MKL1\_HUMAN was not found.
- pid-erbb1
  [+]

  No paths found.
  - MKL1\_HUMAN was not found.
- cancer-cell-map-egfr1
  [+]

  No paths found.
  - MKL1\_HUMAN was not found.
- kegg-erbb
  [+]

  No paths found.
  - MKL1\_HUMAN was not found.

#### 4. Resources that don't match any endpoint

- biocarta-egf
  [+]

  No paths found.
  - MK01\_HUMAN was not found.
  - MKL1\_HUMAN was not found.

### CDK1\_HUMAN → MP2K1\_HUMAN / CDK1\_HUMAN ⊣ MP2K1\_HUMAN

#### 1. Resources that have the edge

- phosphositeplus-kinase-substrate
  [+]

  Resource paths:
  - P06493 {CDK1\_HUMAN} (Protein) → Q02750 {MP2K1\_HUMAN} (Protein) [ProteinProteinInteraction (unknown sign)]

#### 2. Resources that contain endpoints (but not an edge)


#### 3. Resources that match only one endpoint (fringe)

- cancer-cell-map-egfr1
  [+]

  No paths found.
  - CDK1\_HUMAN was not found.
- kegg-mapk
  [+]

  No paths found.
  - CDK1\_HUMAN was not found.
- layek
  [+]

  No paths found.
  - CDK1\_HUMAN was not found.
- kegg-erbb
  [+]

  No paths found.
  - CDK1\_HUMAN was not found.
- reactome-egfr
  [+]

  No paths found.
  - CDK1\_HUMAN was not found.
- science-signaling-egfr
  [+]

  No paths found.
  - CDK1\_HUMAN was not found.

#### 4. Resources that don't match any endpoint

- pid-erbb1
  [+]

  No paths found.
  - CDK1\_HUMAN was not found.
  - MP2K1\_HUMAN was not found.
- biocarta-egf
  [+]

  No paths found.
  - CDK1\_HUMAN was not found.
  - MP2K1\_HUMAN was not found.

### AKT1\_HUMAN → 1433Z\_HUMAN / AKT1\_HUMAN ⊣ 1433Z\_HUMAN

#### 1. Resources that have the edge

- phosphositeplus-kinase-substrate
  [+]

  Resource paths:
  - P31749 {AKT1\_HUMAN} (Protein) → P63104 {1433Z\_HUMAN} (Protein) [ProteinProteinInteraction (unknown sign)]

#### 2. Resources that contain endpoints (but not an edge)


#### 3. Resources that match only one endpoint (fringe)

- cancer-cell-map-egfr1
  [+]

  No paths found.
  - 1433Z\_HUMAN was not found.
- layek
  [+]

  No paths found.
  - 1433Z\_HUMAN was not found.
- reactome-egfr
  [+]

  No paths found.
  - 1433Z\_HUMAN was not found.
- science-signaling-egfr
  [+]

  No paths found.
  - 1433Z\_HUMAN was not found.

#### 4. Resources that don't match any endpoint

- kegg-mapk
  [+]

  No paths found.
  - AKT1\_HUMAN was not found.
  - 1433Z\_HUMAN was not found.
- kegg-erbb
  [+]

  No paths found.
  - AKT1\_HUMAN was not found.
  - 1433Z\_HUMAN was not found.
- pid-erbb1
  [+]

  No paths found.
  - AKT1\_HUMAN was not found.
  - 1433Z\_HUMAN was not found.
- biocarta-egf
  [+]

  No paths found.
  - AKT1\_HUMAN was not found.
  - 1433Z\_HUMAN was not found.

### AKT1\_HUMAN → PDCD4\_HUMAN / AKT1\_HUMAN ⊣ PDCD4\_HUMAN

#### 1. Resources that have the edge

- phosphositeplus-kinase-substrate
  [+]

  Resource paths:
  - P31749 {AKT1\_HUMAN} (Protein) → Q53EL6 {PDCD4\_HUMAN} (Protein) [ProteinProteinInteraction (unknown sign)]

#### 2. Resources that contain endpoints (but not an edge)


#### 3. Resources that match only one endpoint (fringe)

- cancer-cell-map-egfr1
  [+]

  No paths found.
  - PDCD4\_HUMAN was not found.
- layek
  [+]

  No paths found.
  - PDCD4\_HUMAN was not found.
- reactome-egfr
  [+]

  No paths found.
  - PDCD4\_HUMAN was not found.
- science-signaling-egfr
  [+]

  No paths found.
  - PDCD4\_HUMAN was not found.

#### 4. Resources that don't match any endpoint

- kegg-mapk
  [+]

  No paths found.
  - AKT1\_HUMAN was not found.
  - PDCD4\_HUMAN was not found.
- kegg-erbb
  [+]

  No paths found.
  - AKT1\_HUMAN was not found.
  - PDCD4\_HUMAN was not found.
- pid-erbb1
  [+]

  No paths found.
  - AKT1\_HUMAN was not found.
  - PDCD4\_HUMAN was not found.
- biocarta-egf
  [+]

  No paths found.
  - AKT1\_HUMAN was not found.
  - PDCD4\_HUMAN was not found.

### AKT1\_HUMAN → RANB3\_HUMAN / AKT1\_HUMAN ⊣ RANB3\_HUMAN

#### 1. Resources that have the edge

- phosphositeplus-kinase-substrate
  [+]

  Resource paths:
  - P31749 {AKT1\_HUMAN} (Protein) → Q9H6Z4 {RANB3\_HUMAN} (Protein) [ProteinProteinInteraction (unknown sign)]

#### 2. Resources that contain endpoints (but not an edge)


#### 3. Resources that match only one endpoint (fringe)

- cancer-cell-map-egfr1
  [+]

  No paths found.
  - RANB3\_HUMAN was not found.
- layek
  [+]

  No paths found.
  - RANB3\_HUMAN was not found.
- reactome-egfr
  [+]

  No paths found.
  - RANB3\_HUMAN was not found.
- science-signaling-egfr
  [+]

  No paths found.
  - RANB3\_HUMAN was not found.

#### 4. Resources that don't match any endpoint

- kegg-mapk
  [+]

  No paths found.
  - AKT1\_HUMAN was not found.
  - RANB3\_HUMAN was not found.
- kegg-erbb
  [+]

  No paths found.
  - AKT1\_HUMAN was not found.
  - RANB3\_HUMAN was not found.
- pid-erbb1
  [+]

  No paths found.
  - AKT1\_HUMAN was not found.
  - RANB3\_HUMAN was not found.
- biocarta-egf
  [+]

  No paths found.
  - AKT1\_HUMAN was not found.
  - RANB3\_HUMAN was not found.

### MK03\_HUMAN → MKL1\_HUMAN

#### 1. Resources that have the edge

- phosphositeplus-kinase-substrate
  [+]

  Resource paths:
  - P27361 {MK03\_HUMAN} (Protein) → Q969V6 {MKL1\_HUMAN} (Protein) [ProteinProteinInteraction (unknown sign)]

#### 2. Resources that contain endpoints (but not an edge)


#### 3. Resources that match only one endpoint (fringe)

- cancer-cell-map-egfr1
  [+]

  No paths found.
  - MKL1\_HUMAN was not found.
- layek
  [+]

  No paths found.
  - MKL1\_HUMAN was not found.
- reactome-egfr
  [+]

  No paths found.
  - MKL1\_HUMAN was not found.
- science-signaling-egfr
  [+]

  No paths found.
  - MKL1\_HUMAN was not found.

#### 4. Resources that don't match any endpoint

- kegg-mapk
  [+]

  No paths found.
  - MK03\_HUMAN was not found.
  - MKL1\_HUMAN was not found.
- kegg-erbb
  [+]

  No paths found.
  - MK03\_HUMAN was not found.
  - MKL1\_HUMAN was not found.
- pid-erbb1
  [+]

  No paths found.
  - MK03\_HUMAN was not found.
  - MKL1\_HUMAN was not found.
- biocarta-egf
  [+]

  No paths found.
  - MK03\_HUMAN was not found.
  - MKL1\_HUMAN was not found.

### AKT1\_HUMAN → ZYX\_HUMAN / AKT1\_HUMAN ⊣ ZYX\_HUMAN

#### 1. Resources that have the edge

- phosphositeplus-kinase-substrate
  [+]

  Resource paths:
  - P31749 {AKT1\_HUMAN} (Protein) → Q15942 {ZYX\_HUMAN} (Protein) [ProteinProteinInteraction (unknown sign)]

#### 2. Resources that contain endpoints (but not an edge)


#### 3. Resources that match only one endpoint (fringe)

- cancer-cell-map-egfr1
  [+]

  No paths found.
  - ZYX\_HUMAN was not found.
- layek
  [+]

  No paths found.
  - ZYX\_HUMAN was not found.
- reactome-egfr
  [+]

  No paths found.
  - ZYX\_HUMAN was not found.
- science-signaling-egfr
  [+]

  No paths found.
  - ZYX\_HUMAN was not found.

#### 4. Resources that don't match any endpoint

- kegg-mapk
  [+]

  No paths found.
  - AKT1\_HUMAN was not found.
  - ZYX\_HUMAN was not found.
- kegg-erbb
  [+]

  No paths found.
  - AKT1\_HUMAN was not found.
  - ZYX\_HUMAN was not found.
- pid-erbb1
  [+]

  No paths found.
  - AKT1\_HUMAN was not found.
  - ZYX\_HUMAN was not found.
- biocarta-egf
  [+]

  No paths found.
  - AKT1\_HUMAN was not found.
  - ZYX\_HUMAN was not found.

### AKT1\_HUMAN → HJURP\_HUMAN / AKT1\_HUMAN ⊣ HJURP\_HUMAN

#### 1. Resources that have the edge

- phosphositeplus-kinase-substrate
  [+]

  Resource paths:
  - P31749 {AKT1\_HUMAN} (Protein) → Q8NCD3 {HJURP\_HUMAN} (Protein) [ProteinProteinInteraction (unknown sign)]

#### 2. Resources that contain endpoints (but not an edge)


#### 3. Resources that match only one endpoint (fringe)

- cancer-cell-map-egfr1
  [+]

  No paths found.
  - HJURP\_HUMAN was not found.
- layek
  [+]

  No paths found.
  - HJURP\_HUMAN was not found.
- reactome-egfr
  [+]

  No paths found.
  - HJURP\_HUMAN was not found.
- science-signaling-egfr
  [+]

  No paths found.
  - HJURP\_HUMAN was not found.

#### 4. Resources that don't match any endpoint

- kegg-mapk
  [+]

  No paths found.
  - AKT1\_HUMAN was not found.
  - HJURP\_HUMAN was not found.
- kegg-erbb
  [+]

  No paths found.
  - AKT1\_HUMAN was not found.
  - HJURP\_HUMAN was not found.
- pid-erbb1
  [+]

  No paths found.
  - AKT1\_HUMAN was not found.
  - HJURP\_HUMAN was not found.
- biocarta-egf
  [+]

  No paths found.
  - AKT1\_HUMAN was not found.
  - HJURP\_HUMAN was not found.

### AKT1\_HUMAN → PALLD\_HUMAN / AKT1\_HUMAN ⊣ PALLD\_HUMAN

#### 1. Resources that have the edge

- phosphositeplus-kinase-substrate
  [+]

  Resource paths:
  - P31749 {AKT1\_HUMAN} (Protein) → Q8WX93 {PALLD\_HUMAN} (Protein) [ProteinProteinInteraction (unknown sign)]

#### 2. Resources that contain endpoints (but not an edge)


#### 3. Resources that match only one endpoint (fringe)

- cancer-cell-map-egfr1
  [+]

  No paths found.
  - PALLD\_HUMAN was not found.
- layek
  [+]

  No paths found.
  - PALLD\_HUMAN was not found.
- reactome-egfr
  [+]

  No paths found.
  - PALLD\_HUMAN was not found.
- science-signaling-egfr
  [+]

  No paths found.
  - PALLD\_HUMAN was not found.

#### 4. Resources that don't match any endpoint

- kegg-mapk
  [+]

  No paths found.
  - AKT1\_HUMAN was not found.
  - PALLD\_HUMAN was not found.
- kegg-erbb
  [+]

  No paths found.
  - AKT1\_HUMAN was not found.
  - PALLD\_HUMAN was not found.
- pid-erbb1
  [+]

  No paths found.
  - AKT1\_HUMAN was not found.
  - PALLD\_HUMAN was not found.
- biocarta-egf
  [+]

  No paths found.
  - AKT1\_HUMAN was not found.
  - PALLD\_HUMAN was not found.

### AKT1\_HUMAN → YAP1\_HUMAN / AKT1\_HUMAN ⊣ YAP1\_HUMAN

#### 1. Resources that have the edge

- phosphositeplus-kinase-substrate
  [+]

  Resource paths:
  - P31749 {AKT1\_HUMAN} (Protein) → P46937 {YAP1\_HUMAN} (Protein) [ProteinProteinInteraction (unknown sign)]

#### 2. Resources that contain endpoints (but not an edge)


#### 3. Resources that match only one endpoint (fringe)

- cancer-cell-map-egfr1
  [+]

  No paths found.
  - YAP1\_HUMAN was not found.
- layek
  [+]

  No paths found.
  - YAP1\_HUMAN was not found.
- reactome-egfr
  [+]

  No paths found.
  - YAP1\_HUMAN was not found.
- science-signaling-egfr
  [+]

  No paths found.
  - YAP1\_HUMAN was not found.

#### 4. Resources that don't match any endpoint

- kegg-mapk
  [+]

  No paths found.
  - AKT1\_HUMAN was not found.
  - YAP1\_HUMAN was not found.
- kegg-erbb
  [+]

  No paths found.
  - AKT1\_HUMAN was not found.
  - YAP1\_HUMAN was not found.
- pid-erbb1
  [+]

  No paths found.
  - AKT1\_HUMAN was not found.
  - YAP1\_HUMAN was not found.
- biocarta-egf
  [+]

  No paths found.
  - AKT1\_HUMAN was not found.
  - YAP1\_HUMAN was not found.

### CSK21\_HUMAN → AKT1\_HUMAN / CSK21\_HUMAN ⊣ AKT1\_HUMAN

#### 1. Resources that have the edge

- phosphositeplus-kinase-substrate
  [+]

  Resource paths:
  - P68400 {CSK21\_HUMAN} (Protein) → P31749 {AKT1\_HUMAN} (Protein) [ProteinProteinInteraction (unknown sign)]

#### 2. Resources that contain endpoints (but not an edge)


#### 3. Resources that match only one endpoint (fringe)

- cancer-cell-map-egfr1
  [+]

  No paths found.
  - CSK21\_HUMAN was not found.
- layek
  [+]

  No paths found.
  - CSK21\_HUMAN was not found.
- reactome-egfr
  [+]

  No paths found.
  - CSK21\_HUMAN was not found.
- science-signaling-egfr
  [+]

  No paths found.
  - CSK21\_HUMAN was not found.

#### 4. Resources that don't match any endpoint

- kegg-mapk
  [+]

  No paths found.
  - CSK21\_HUMAN was not found.
  - AKT1\_HUMAN was not found.
- kegg-erbb
  [+]

  No paths found.
  - CSK21\_HUMAN was not found.
  - AKT1\_HUMAN was not found.
- pid-erbb1
  [+]

  No paths found.
  - CSK21\_HUMAN was not found.
  - AKT1\_HUMAN was not found.
- biocarta-egf
  [+]

  No paths found.
  - CSK21\_HUMAN was not found.
  - AKT1\_HUMAN was not found.

### AKT1\_HUMAN → ACINU\_HUMAN / AKT1\_HUMAN ⊣ ACINU\_HUMAN

#### 1. Resources that have the edge

- phosphositeplus-kinase-substrate
  [+]

  Resource paths:
  - P31749 {AKT1\_HUMAN} (Protein) → Q9UKV3 {ACINU\_HUMAN} (Protein) [ProteinProteinInteraction (unknown sign)]

#### 2. Resources that contain endpoints (but not an edge)


#### 3. Resources that match only one endpoint (fringe)

- cancer-cell-map-egfr1
  [+]

  No paths found.
  - ACINU\_HUMAN was not found.
- layek
  [+]

  No paths found.
  - ACINU\_HUMAN was not found.
- reactome-egfr
  [+]

  No paths found.
  - ACINU\_HUMAN was not found.
- science-signaling-egfr
  [+]

  No paths found.
  - ACINU\_HUMAN was not found.

#### 4. Resources that don't match any endpoint

- kegg-mapk
  [+]

  No paths found.
  - AKT1\_HUMAN was not found.
  - ACINU\_HUMAN was not found.
- kegg-erbb
  [+]

  No paths found.
  - AKT1\_HUMAN was not found.
  - ACINU\_HUMAN was not found.
- pid-erbb1
  [+]

  No paths found.
  - AKT1\_HUMAN was not found.
  - ACINU\_HUMAN was not found.
- biocarta-egf
  [+]

  No paths found.
  - AKT1\_HUMAN was not found.
  - ACINU\_HUMAN was not found.

### KAPCA\_HUMAN → DESP\_HUMAN / KAPCA\_HUMAN ⊣ DESP\_HUMAN

#### 1. Resources that have the edge

- phosphositeplus-kinase-substrate
  [+]

  Resource paths:
  - P17612 {KAPCA\_HUMAN} (Protein) → P15924 {DESP\_HUMAN} (Protein) [ProteinProteinInteraction (unknown sign)]

#### 2. Resources that contain endpoints (but not an edge)


#### 3. Resources that match only one endpoint (fringe)

- cancer-cell-map-egfr1
  [+]

  No paths found.
  - DESP\_HUMAN was not found.
- kegg-mapk
  [+]

  No paths found.
  - DESP\_HUMAN was not found.
- reactome-egfr
  [+]

  No paths found.
  - DESP\_HUMAN was not found.

#### 4. Resources that don't match any endpoint

- kegg-erbb
  [+]

  No paths found.
  - KAPCA\_HUMAN was not found.
  - DESP\_HUMAN was not found.
- biocarta-egf
  [+]

  No paths found.
  - KAPCA\_HUMAN was not found.
  - DESP\_HUMAN was not found.
- layek
  [+]

  No paths found.
  - KAPCA\_HUMAN was not found.
  - DESP\_HUMAN was not found.
- pid-erbb1
  [+]

  No paths found.
  - KAPCA\_HUMAN was not found.
  - DESP\_HUMAN was not found.
- science-signaling-egfr
  [+]

  No paths found.
  - KAPCA\_HUMAN was not found.
  - DESP\_HUMAN was not found.

### KAPCA\_HUMAN → GSK3A\_HUMAN / KAPCA\_HUMAN ⊣ GSK3A\_HUMAN

#### 1. Resources that have the edge

- phosphositeplus-kinase-substrate
  [+]

  Resource paths:
  - P17612 {KAPCA\_HUMAN} (Protein) → P49840 {GSK3A\_HUMAN} (Protein) [ProteinProteinInteraction (unknown sign)]

#### 2. Resources that contain endpoints (but not an edge)


#### 3. Resources that match only one endpoint (fringe)

- cancer-cell-map-egfr1
  [+]

  No paths found.
  - GSK3A\_HUMAN was not found.
- kegg-mapk
  [+]

  No paths found.
  - GSK3A\_HUMAN was not found.
- reactome-egfr
  [+]

  No paths found.
  - GSK3A\_HUMAN was not found.

#### 4. Resources that don't match any endpoint

- science-signaling-egfr
  [+]

  No paths found.
  - KAPCA\_HUMAN was not found.
  - GSK3A\_HUMAN was not found.
- pid-erbb1
  [+]

  No paths found.
  - KAPCA\_HUMAN was not found.
  - GSK3A\_HUMAN was not found.
- biocarta-egf
  [+]

  No paths found.
  - KAPCA\_HUMAN was not found.
  - GSK3A\_HUMAN was not found.
- layek
  [+]

  No paths found.
  - KAPCA\_HUMAN was not found.
  - GSK3A\_HUMAN was not found.
- kegg-erbb
  [+]

  No paths found.
  - KAPCA\_HUMAN was not found.
  - GSK3A\_HUMAN was not found.

### CBL\_HUMAN → ARHG7\_HUMAN / CBL\_HUMAN ⊣ ARHG7\_HUMAN

#### 1. Resources that have the edge

- reactome-egfr
  [+]

  Resource has common complex(es):
  - Complex3693 {EGF:Phospho-EGFR (Y1045) dimer:CBL:Cool/Pix:CDC42-GTP, EGF:p-6Y-EGFR:CBL:Beta-Pix:CDC42:GTP} (Complex)
  - Complex3690 {CBL:Cool/Pix:CDC42:GTP, CBL:Beta-Pix:CDC42:GTP} (Complex)
  - Complex3689 {CBL:Cool-Pix, CBL:Beta-Pix} (Complex)
  - ARHG7\_HUMAN and COOL1 are synonyms.
  - CBL\_HUMAN and CBL2 are synonyms.

#### 2. Resources that contain endpoints (but not an edge)


#### 3. Resources that match only one endpoint (fringe)

- phosphositeplus-kinase-substrate
  [+]

  No paths found.
  - ARHG7\_HUMAN was not found.
- cancer-cell-map-egfr1
  [+]

  No paths found.
  - ARHG7\_HUMAN was not found.
- science-signaling-egfr
  [+]

  No paths found.
  - ARHG7\_HUMAN was not found.

#### 4. Resources that don't match any endpoint

- kegg-mapk
  [+]

  No paths found.
  - ARHG7\_HUMAN was not found.
  - CBL\_HUMAN was not found.
- biocarta-egf
  [+]

  No paths found.
  - ARHG7\_HUMAN was not found.
  - CBL\_HUMAN was not found.
- pid-erbb1
  [+]

  No paths found.
  - ARHG7\_HUMAN was not found.
  - CBL\_HUMAN was not found.
- layek
  [+]

  No paths found.
  - ARHG7\_HUMAN was not found.
  - CBL\_HUMAN was not found.
- kegg-erbb
  [+]

  No paths found.
  - ARHG7\_HUMAN was not found.
  - CBL\_HUMAN was not found.

### KAPCA\_HUMAN → AT1A1\_HUMAN / KAPCA\_HUMAN ⊣ AT1A1\_HUMAN

#### 1. Resources that have the edge

- phosphositeplus-kinase-substrate
  [+]

  Resource paths:
  - P17612 {KAPCA\_HUMAN} (Protein) → P05023 {AT1A1\_HUMAN} (Protein) [ProteinProteinInteraction (unknown sign)]

#### 2. Resources that contain endpoints (but not an edge)


#### 3. Resources that match only one endpoint (fringe)

- cancer-cell-map-egfr1
  [+]

  No paths found.
  - AT1A1\_HUMAN was not found.
- kegg-mapk
  [+]

  No paths found.
  - AT1A1\_HUMAN was not found.
- reactome-egfr
  [+]

  No paths found.
  - AT1A1\_HUMAN was not found.

#### 4. Resources that don't match any endpoint

- science-signaling-egfr
  [+]

  No paths found.
  - KAPCA\_HUMAN was not found.
  - AT1A1\_HUMAN was not found.
- layek
  [+]

  No paths found.
  - KAPCA\_HUMAN was not found.
  - AT1A1\_HUMAN was not found.
- biocarta-egf
  [+]

  No paths found.
  - KAPCA\_HUMAN was not found.
  - AT1A1\_HUMAN was not found.
- kegg-erbb
  [+]

  No paths found.
  - KAPCA\_HUMAN was not found.
  - AT1A1\_HUMAN was not found.
- pid-erbb1
  [+]

  No paths found.
  - KAPCA\_HUMAN was not found.
  - AT1A1\_HUMAN was not found.

### KAPCA\_HUMAN → 1433Z\_HUMAN / KAPCA\_HUMAN ⊣ 1433Z\_HUMAN

#### 1. Resources that have the edge

- phosphositeplus-kinase-substrate
  [+]

  Resource paths:
  - P17612 {KAPCA\_HUMAN} (Protein) → P63104 {1433Z\_HUMAN} (Protein) [ProteinProteinInteraction (unknown sign)]

#### 2. Resources that contain endpoints (but not an edge)


#### 3. Resources that match only one endpoint (fringe)

- cancer-cell-map-egfr1
  [+]

  No paths found.
  - 1433Z\_HUMAN was not found.
- kegg-mapk
  [+]

  No paths found.
  - 1433Z\_HUMAN was not found.
- reactome-egfr
  [+]

  No paths found.
  - 1433Z\_HUMAN was not found.

#### 4. Resources that don't match any endpoint

- biocarta-egf
  [+]

  No paths found.
  - KAPCA\_HUMAN was not found.
  - 1433Z\_HUMAN was not found.
- pid-erbb1
  [+]

  No paths found.
  - KAPCA\_HUMAN was not found.
  - 1433Z\_HUMAN was not found.
- kegg-erbb
  [+]

  No paths found.
  - KAPCA\_HUMAN was not found.
  - 1433Z\_HUMAN was not found.
- science-signaling-egfr
  [+]

  No paths found.
  - KAPCA\_HUMAN was not found.
  - 1433Z\_HUMAN was not found.
- layek
  [+]

  No paths found.
  - KAPCA\_HUMAN was not found.
  - 1433Z\_HUMAN was not found.

### KAPCA\_HUMAN → KKCC1\_HUMAN / KAPCA\_HUMAN ⊣ KKCC1\_HUMAN

#### 1. Resources that have the edge

- phosphositeplus-kinase-substrate
  [+]

  Resource paths:
  - P17612 {KAPCA\_HUMAN} (Protein) → Q8N5S9 {KKCC1\_HUMAN} (Protein) [ProteinProteinInteraction (unknown sign)]

#### 2. Resources that contain endpoints (but not an edge)


#### 3. Resources that match only one endpoint (fringe)

- cancer-cell-map-egfr1
  [+]

  No paths found.
  - KKCC1\_HUMAN was not found.
- kegg-mapk
  [+]

  No paths found.
  - KKCC1\_HUMAN was not found.
- reactome-egfr
  [+]

  No paths found.
  - KKCC1\_HUMAN was not found.

#### 4. Resources that don't match any endpoint

- biocarta-egf
  [+]

  No paths found.
  - KAPCA\_HUMAN was not found.
  - KKCC1\_HUMAN was not found.
- science-signaling-egfr
  [+]

  No paths found.
  - KAPCA\_HUMAN was not found.
  - KKCC1\_HUMAN was not found.
- pid-erbb1
  [+]

  No paths found.
  - KAPCA\_HUMAN was not found.
  - KKCC1\_HUMAN was not found.
- layek
  [+]

  No paths found.
  - KAPCA\_HUMAN was not found.
  - KKCC1\_HUMAN was not found.
- kegg-erbb
  [+]

  No paths found.
  - KAPCA\_HUMAN was not found.
  - KKCC1\_HUMAN was not found.

### KAPCA\_HUMAN → CP3A4\_HUMAN / KAPCA\_HUMAN ⊣ CP3A4\_HUMAN

#### 1. Resources that have the edge

- phosphositeplus-kinase-substrate
  [+]

  Resource paths:
  - P17612 {KAPCA\_HUMAN} (Protein) → P08684 {CP3A4\_HUMAN} (Protein) [ProteinProteinInteraction (unknown sign)]

#### 2. Resources that contain endpoints (but not an edge)


#### 3. Resources that match only one endpoint (fringe)

- cancer-cell-map-egfr1
  [+]

  No paths found.
  - CP3A4\_HUMAN was not found.
- kegg-mapk
  [+]

  No paths found.
  - CP3A4\_HUMAN was not found.
- reactome-egfr
  [+]

  No paths found.
  - CP3A4\_HUMAN was not found.

#### 4. Resources that don't match any endpoint

- pid-erbb1
  [+]

  No paths found.
  - KAPCA\_HUMAN was not found.
  - CP3A4\_HUMAN was not found.
- biocarta-egf
  [+]

  No paths found.
  - KAPCA\_HUMAN was not found.
  - CP3A4\_HUMAN was not found.
- layek
  [+]

  No paths found.
  - KAPCA\_HUMAN was not found.
  - CP3A4\_HUMAN was not found.
- science-signaling-egfr
  [+]

  No paths found.
  - KAPCA\_HUMAN was not found.
  - CP3A4\_HUMAN was not found.
- kegg-erbb
  [+]

  No paths found.
  - KAPCA\_HUMAN was not found.
  - CP3A4\_HUMAN was not found.

### KAPCA\_HUMAN → PLSL\_HUMAN / KAPCA\_HUMAN ⊣ PLSL\_HUMAN

#### 1. Resources that have the edge

- phosphositeplus-kinase-substrate
  [+]

  Resource paths:
  - P17612 {KAPCA\_HUMAN} (Protein) → P13796 {PLSL\_HUMAN} (Protein) [ProteinProteinInteraction (unknown sign)]

#### 2. Resources that contain endpoints (but not an edge)


#### 3. Resources that match only one endpoint (fringe)

- cancer-cell-map-egfr1
  [+]

  No paths found.
  - PLSL\_HUMAN was not found.
- kegg-mapk
  [+]

  No paths found.
  - PLSL\_HUMAN was not found.
- reactome-egfr
  [+]

  No paths found.
  - PLSL\_HUMAN was not found.

#### 4. Resources that don't match any endpoint

- layek
  [+]

  No paths found.
  - KAPCA\_HUMAN was not found.
  - PLSL\_HUMAN was not found.
- science-signaling-egfr
  [+]

  No paths found.
  - KAPCA\_HUMAN was not found.
  - PLSL\_HUMAN was not found.
- biocarta-egf
  [+]

  No paths found.
  - KAPCA\_HUMAN was not found.
  - PLSL\_HUMAN was not found.
- kegg-erbb
  [+]

  No paths found.
  - KAPCA\_HUMAN was not found.
  - PLSL\_HUMAN was not found.
- pid-erbb1
  [+]

  No paths found.
  - KAPCA\_HUMAN was not found.
  - PLSL\_HUMAN was not found.

### KAPCA\_HUMAN → CDK16\_HUMAN / KAPCA\_HUMAN ⊣ CDK16\_HUMAN

#### 1. Resources that have the edge

- phosphositeplus-kinase-substrate
  [+]

  Resource paths:
  - P17612 {KAPCA\_HUMAN} (Protein) → Q00536 {CDK16\_HUMAN} (Protein) [ProteinProteinInteraction (unknown sign)]

#### 2. Resources that contain endpoints (but not an edge)


#### 3. Resources that match only one endpoint (fringe)

- cancer-cell-map-egfr1
  [+]

  No paths found.
  - CDK16\_HUMAN was not found.
- kegg-mapk
  [+]

  No paths found.
  - CDK16\_HUMAN was not found.
- reactome-egfr
  [+]

  No paths found.
  - CDK16\_HUMAN was not found.

#### 4. Resources that don't match any endpoint

- biocarta-egf
  [+]

  No paths found.
  - KAPCA\_HUMAN was not found.
  - CDK16\_HUMAN was not found.
- pid-erbb1
  [+]

  No paths found.
  - KAPCA\_HUMAN was not found.
  - CDK16\_HUMAN was not found.
- science-signaling-egfr
  [+]

  No paths found.
  - KAPCA\_HUMAN was not found.
  - CDK16\_HUMAN was not found.
- layek
  [+]

  No paths found.
  - KAPCA\_HUMAN was not found.
  - CDK16\_HUMAN was not found.
- kegg-erbb
  [+]

  No paths found.
  - KAPCA\_HUMAN was not found.
  - CDK16\_HUMAN was not found.

### KAPCA\_HUMAN → DNM1L\_HUMAN / KAPCA\_HUMAN ⊣ DNM1L\_HUMAN

#### 1. Resources that have the edge

- phosphositeplus-kinase-substrate
  [+]

  Resource paths:
  - P17612 {KAPCA\_HUMAN} (Protein) → O00429 {DNM1L\_HUMAN} (Protein) [ProteinProteinInteraction (unknown sign)]

#### 2. Resources that contain endpoints (but not an edge)


#### 3. Resources that match only one endpoint (fringe)

- cancer-cell-map-egfr1
  [+]

  No paths found.
  - DNM1L\_HUMAN was not found.
- kegg-mapk
  [+]

  No paths found.
  - DNM1L\_HUMAN was not found.
- reactome-egfr
  [+]

  No paths found.
  - DNM1L\_HUMAN was not found.

#### 4. Resources that don't match any endpoint

- pid-erbb1
  [+]

  No paths found.
  - KAPCA\_HUMAN was not found.
  - DNM1L\_HUMAN was not found.
- science-signaling-egfr
  [+]

  No paths found.
  - KAPCA\_HUMAN was not found.
  - DNM1L\_HUMAN was not found.
- biocarta-egf
  [+]

  No paths found.
  - KAPCA\_HUMAN was not found.
  - DNM1L\_HUMAN was not found.
- layek
  [+]

  No paths found.
  - KAPCA\_HUMAN was not found.
  - DNM1L\_HUMAN was not found.
- kegg-erbb
  [+]

  No paths found.
  - KAPCA\_HUMAN was not found.
  - DNM1L\_HUMAN was not found.

### MK09\_HUMAN → STAT3\_HUMAN / MK09\_HUMAN ⊣ STAT3\_HUMAN

#### 1. Resources that have the edge

- phosphositeplus-kinase-substrate
  [+]

  Resource paths:
  - P45984 {MK09\_HUMAN} (Protein) → P40763 {STAT3\_HUMAN} (Protein) [ProteinProteinInteraction (unknown sign)]

#### 2. Resources that contain endpoints (but not an edge)


#### 3. Resources that match only one endpoint (fringe)

- cancer-cell-map-egfr1
  [+]

  No paths found.
  - MK09\_HUMAN was not found.
- pid-erbb1
  [+]

  No paths found.
  - MK09\_HUMAN was not found.
- biocarta-egf
  [+]

  No paths found.
  - MK09\_HUMAN was not found.

#### 4. Resources that don't match any endpoint

- kegg-erbb
  [+]

  No paths found.
  - MK09\_HUMAN was not found.
  - STAT3\_HUMAN was not found.
- reactome-egfr
  [+]

  No paths found.
  - MK09\_HUMAN was not found.
  - STAT3\_HUMAN was not found.
- science-signaling-egfr
  [+]

  No paths found.
  - MK09\_HUMAN was not found.
  - STAT3\_HUMAN was not found.
- kegg-mapk
  [+]

  No paths found.
  - MK09\_HUMAN was not found.
  - STAT3\_HUMAN was not found.
- layek
  [+]

  No paths found.
  - MK09\_HUMAN was not found.
  - STAT3\_HUMAN was not found.

### PAK1\_HUMAN → STMN1\_HUMAN / PAK1\_HUMAN ⊣ STMN1\_HUMAN

#### 1. Resources that have the edge

- phosphositeplus-kinase-substrate
  [+]

  Resource paths:
  - Q13153 {PAK1\_HUMAN} (Protein) → P16949 {STMN1\_HUMAN} (Protein) [ProteinProteinInteraction (unknown sign)]

#### 2. Resources that contain endpoints (but not an edge)


#### 3. Resources that match only one endpoint (fringe)

- cancer-cell-map-egfr1
  [+]

  No paths found.
  - STMN1\_HUMAN was not found.
- pid-erbb1
  [+]

  No paths found.
  - STMN1\_HUMAN was not found.

#### 4. Resources that don't match any endpoint

- layek
  [+]

  No paths found.
  - PAK1\_HUMAN was not found.
  - STMN1\_HUMAN was not found.
- kegg-mapk
  [+]

  No paths found.
  - PAK1\_HUMAN was not found.
  - STMN1\_HUMAN was not found.
- kegg-erbb
  [+]

  No paths found.
  - PAK1\_HUMAN was not found.
  - STMN1\_HUMAN was not found.
- reactome-egfr
  [+]

  No paths found.
  - PAK1\_HUMAN was not found.
  - STMN1\_HUMAN was not found.
- science-signaling-egfr
  [+]

  No paths found.
  - PAK1\_HUMAN was not found.
  - STMN1\_HUMAN was not found.
- biocarta-egf
  [+]

  No paths found.
  - PAK1\_HUMAN was not found.
  - STMN1\_HUMAN was not found.

### PAK1\_HUMAN → TBCB\_HUMAN / PAK1\_HUMAN ⊣ TBCB\_HUMAN

#### 1. Resources that have the edge

- phosphositeplus-kinase-substrate
  [+]

  Resource paths:
  - Q13153 {PAK1\_HUMAN} (Protein) → Q99426 {TBCB\_HUMAN} (Protein) [ProteinProteinInteraction (unknown sign)]

#### 2. Resources that contain endpoints (but not an edge)


#### 3. Resources that match only one endpoint (fringe)

- cancer-cell-map-egfr1
  [+]

  No paths found.
  - TBCB\_HUMAN was not found.
- pid-erbb1
  [+]

  No paths found.
  - TBCB\_HUMAN was not found.

#### 4. Resources that don't match any endpoint

- science-signaling-egfr
  [+]

  No paths found.
  - PAK1\_HUMAN was not found.
  - TBCB\_HUMAN was not found.
- reactome-egfr
  [+]

  No paths found.
  - PAK1\_HUMAN was not found.
  - TBCB\_HUMAN was not found.
- kegg-mapk
  [+]

  No paths found.
  - PAK1\_HUMAN was not found.
  - TBCB\_HUMAN was not found.
- biocarta-egf
  [+]

  No paths found.
  - PAK1\_HUMAN was not found.
  - TBCB\_HUMAN was not found.
- kegg-erbb
  [+]

  No paths found.
  - PAK1\_HUMAN was not found.
  - TBCB\_HUMAN was not found.
- layek
  [+]

  No paths found.
  - PAK1\_HUMAN was not found.
  - TBCB\_HUMAN was not found.

### FAK1\_HUMAN → AT2B4\_HUMAN / FAK1\_HUMAN ⊣ AT2B4\_HUMAN

#### 1. Resources that have the edge

- phosphositeplus-kinase-substrate
  [+]

  Resource paths:
  - Q05397 {FAK1\_HUMAN} (Protein) → P23634 {AT2B4\_HUMAN} (Protein) [ProteinProteinInteraction (unknown sign)]

#### 2. Resources that contain endpoints (but not an edge)


#### 3. Resources that match only one endpoint (fringe)

- kegg-erbb
  [+]

  No paths found.
  - AT2B4\_HUMAN was not found.
- pid-erbb1
  [+]

  No paths found.
  - AT2B4\_HUMAN was not found.

#### 4. Resources that don't match any endpoint

- reactome-egfr
  [+]

  No paths found.
  - FAK1\_HUMAN was not found.
  - AT2B4\_HUMAN was not found.
- science-signaling-egfr
  [+]

  No paths found.
  - FAK1\_HUMAN was not found.
  - AT2B4\_HUMAN was not found.
- layek
  [+]

  No paths found.
  - FAK1\_HUMAN was not found.
  - AT2B4\_HUMAN was not found.
- cancer-cell-map-egfr1
  [+]

  No paths found.
  - FAK1\_HUMAN was not found.
  - AT2B4\_HUMAN was not found.
- kegg-mapk
  [+]

  No paths found.
  - FAK1\_HUMAN was not found.
  - AT2B4\_HUMAN was not found.
- biocarta-egf
  [+]

  No paths found.
  - FAK1\_HUMAN was not found.
  - AT2B4\_HUMAN was not found.

### PAK1\_HUMAN → PGAM1\_HUMAN / PAK1\_HUMAN ⊣ PGAM1\_HUMAN

#### 1. Resources that have the edge

- phosphositeplus-kinase-substrate
  [+]

  Resource paths:
  - Q13153 {PAK1\_HUMAN} (Protein) → P18669 {PGAM1\_HUMAN} (Protein) [ProteinProteinInteraction (unknown sign)]

#### 2. Resources that contain endpoints (but not an edge)


#### 3. Resources that match only one endpoint (fringe)

- cancer-cell-map-egfr1
  [+]

  No paths found.
  - PGAM1\_HUMAN was not found.
- pid-erbb1
  [+]

  No paths found.
  - PGAM1\_HUMAN was not found.

#### 4. Resources that don't match any endpoint

- kegg-mapk
  [+]

  No paths found.
  - PAK1\_HUMAN was not found.
  - PGAM1\_HUMAN was not found.
- reactome-egfr
  [+]

  No paths found.
  - PAK1\_HUMAN was not found.
  - PGAM1\_HUMAN was not found.
- layek
  [+]

  No paths found.
  - PAK1\_HUMAN was not found.
  - PGAM1\_HUMAN was not found.
- kegg-erbb
  [+]

  No paths found.
  - PAK1\_HUMAN was not found.
  - PGAM1\_HUMAN was not found.
- biocarta-egf
  [+]

  No paths found.
  - PAK1\_HUMAN was not found.
  - PGAM1\_HUMAN was not found.
- science-signaling-egfr
  [+]

  No paths found.
  - PAK1\_HUMAN was not found.
  - PGAM1\_HUMAN was not found.

### ABL2\_HUMAN → CRK\_HUMAN / ABL2\_HUMAN ⊣ CRK\_HUMAN

#### 1. Resources that have the edge

- phosphositeplus-kinase-substrate
  [+]

  Resource paths:
  - P42684 {ABL2\_HUMAN} (Protein) → P46108 {CRK\_HUMAN} (Protein) [ProteinProteinInteraction (unknown sign)]

#### 2. Resources that contain endpoints (but not an edge)


#### 3. Resources that match only one endpoint (fringe)

- kegg-erbb
  [+]

  No paths found.
  - ABL2\_HUMAN was not found.

#### 4. Resources that don't match any endpoint

- kegg-mapk
  [+]

  No paths found.
  - ABL2\_HUMAN was not found.
  - CRK\_HUMAN was not found.
- science-signaling-egfr
  [+]

  No paths found.
  - ABL2\_HUMAN was not found.
  - CRK\_HUMAN was not found.
- cancer-cell-map-egfr1
  [+]

  No paths found.
  - ABL2\_HUMAN was not found.
  - CRK\_HUMAN was not found.
- layek
  [+]

  No paths found.
  - ABL2\_HUMAN was not found.
  - CRK\_HUMAN was not found.
- pid-erbb1
  [+]

  No paths found.
  - ABL2\_HUMAN was not found.
  - CRK\_HUMAN was not found.
- biocarta-egf
  [+]

  No paths found.
  - ABL2\_HUMAN was not found.
  - CRK\_HUMAN was not found.
- reactome-egfr
  [+]

  No paths found.
  - ABL2\_HUMAN was not found.
  - CRK\_HUMAN was not found.

### CSK21\_HUMAN → HDAC2\_HUMAN / CSK21\_HUMAN ⊣ HDAC2\_HUMAN

#### 1. Resources that have the edge

- phosphositeplus-kinase-substrate
  [+]

  Resource paths:
  - P68400 {CSK21\_HUMAN} (Protein) → Q92769 {HDAC2\_HUMAN} (Protein) [ProteinProteinInteraction (unknown sign)]

#### 2. Resources that contain endpoints (but not an edge)


#### 3. Resources that match only one endpoint (fringe)

- cancer-cell-map-egfr1
  [+]

  No paths found.
  - CSK21\_HUMAN was not found.

#### 4. Resources that don't match any endpoint

- layek
  [+]

  No paths found.
  - CSK21\_HUMAN was not found.
  - HDAC2\_HUMAN was not found.
- kegg-erbb
  [+]

  No paths found.
  - CSK21\_HUMAN was not found.
  - HDAC2\_HUMAN was not found.
- biocarta-egf
  [+]

  No paths found.
  - CSK21\_HUMAN was not found.
  - HDAC2\_HUMAN was not found.
- kegg-mapk
  [+]

  No paths found.
  - CSK21\_HUMAN was not found.
  - HDAC2\_HUMAN was not found.
- science-signaling-egfr
  [+]

  No paths found.
  - CSK21\_HUMAN was not found.
  - HDAC2\_HUMAN was not found.
- pid-erbb1
  [+]

  No paths found.
  - CSK21\_HUMAN was not found.
  - HDAC2\_HUMAN was not found.
- reactome-egfr
  [+]

  No paths found.
  - CSK21\_HUMAN was not found.
  - HDAC2\_HUMAN was not found.

### WNK1\_HUMAN → STK39\_HUMAN / WNK1\_HUMAN ⊣ STK39\_HUMAN

#### 1. Resources that have the edge

- phosphositeplus-kinase-substrate
  [+]

  Resource paths:
  - Q9H4A3 {WNK1\_HUMAN} (Protein) → Q9UEW8 {STK39\_HUMAN} (Protein) [ProteinProteinInteraction (unknown sign)]

#### 2. Resources that contain endpoints (but not an edge)


#### 3. Resources that match only one endpoint (fringe)

- cancer-cell-map-egfr1
  [+]

  No paths found.
  - STK39\_HUMAN was not found.

#### 4. Resources that don't match any endpoint

- kegg-mapk
  [+]

  No paths found.
  - WNK1\_HUMAN was not found.
  - STK39\_HUMAN was not found.
- layek
  [+]

  No paths found.
  - WNK1\_HUMAN was not found.
  - STK39\_HUMAN was not found.
- science-signaling-egfr
  [+]

  No paths found.
  - WNK1\_HUMAN was not found.
  - STK39\_HUMAN was not found.
- reactome-egfr
  [+]

  No paths found.
  - WNK1\_HUMAN was not found.
  - STK39\_HUMAN was not found.
- pid-erbb1
  [+]

  No paths found.
  - WNK1\_HUMAN was not found.
  - STK39\_HUMAN was not found.
- biocarta-egf
  [+]

  No paths found.
  - WNK1\_HUMAN was not found.
  - STK39\_HUMAN was not found.
- kegg-erbb
  [+]

  No paths found.
  - WNK1\_HUMAN was not found.
  - STK39\_HUMAN was not found.

### CDK1\_HUMAN → NUP98\_HUMAN / CDK1\_HUMAN ⊣ NUP98\_HUMAN

#### 1. Resources that have the edge

- phosphositeplus-kinase-substrate
  [+]

  Resource paths:
  - P06493 {CDK1\_HUMAN} (Protein) → P52948 {NUP98\_HUMAN} (Protein) [ProteinProteinInteraction (unknown sign)]

#### 2. Resources that contain endpoints (but not an edge)


#### 3. Resources that match only one endpoint (fringe)


#### 4. Resources that don't match any endpoint

- biocarta-egf
  [+]

  No paths found.
  - CDK1\_HUMAN was not found.
  - NUP98\_HUMAN was not found.
- reactome-egfr
  [+]

  No paths found.
  - CDK1\_HUMAN was not found.
  - NUP98\_HUMAN was not found.
- science-signaling-egfr
  [+]

  No paths found.
  - CDK1\_HUMAN was not found.
  - NUP98\_HUMAN was not found.
- cancer-cell-map-egfr1
  [+]

  No paths found.
  - CDK1\_HUMAN was not found.
  - NUP98\_HUMAN was not found.
- pid-erbb1
  [+]

  No paths found.
  - CDK1\_HUMAN was not found.
  - NUP98\_HUMAN was not found.
- kegg-erbb
  [+]

  No paths found.
  - CDK1\_HUMAN was not found.
  - NUP98\_HUMAN was not found.
- layek
  [+]

  No paths found.
  - CDK1\_HUMAN was not found.
  - NUP98\_HUMAN was not found.
- kegg-mapk
  [+]

  No paths found.
  - CDK1\_HUMAN was not found.
  - NUP98\_HUMAN was not found.

### ATM\_HUMAN → TAOK1\_HUMAN / ATM\_HUMAN ⊣ TAOK1\_HUMAN

#### 1. Resources that have the edge

- phosphositeplus-kinase-substrate
  [+]

  Resource paths:
  - Q13315 {ATM\_HUMAN} (Protein) → Q7L7X3 {TAOK1\_HUMAN} (Protein) [ProteinProteinInteraction (unknown sign)]

#### 2. Resources that contain endpoints (but not an edge)


#### 3. Resources that match only one endpoint (fringe)


#### 4. Resources that don't match any endpoint

- kegg-erbb
  [+]

  No paths found.
  - ATM\_HUMAN was not found.
  - TAOK1\_HUMAN was not found.
- pid-erbb1
  [+]

  No paths found.
  - ATM\_HUMAN was not found.
  - TAOK1\_HUMAN was not found.
- reactome-egfr
  [+]

  No paths found.
  - ATM\_HUMAN was not found.
  - TAOK1\_HUMAN was not found.
- layek
  [+]

  No paths found.
  - ATM\_HUMAN was not found.
  - TAOK1\_HUMAN was not found.
- biocarta-egf
  [+]

  No paths found.
  - ATM\_HUMAN was not found.
  - TAOK1\_HUMAN was not found.
- kegg-mapk
  [+]

  No paths found.
  - ATM\_HUMAN was not found.
  - TAOK1\_HUMAN was not found.
- cancer-cell-map-egfr1
  [+]

  No paths found.
  - ATM\_HUMAN was not found.
  - TAOK1\_HUMAN was not found.
- science-signaling-egfr
  [+]

  No paths found.
  - ATM\_HUMAN was not found.
  - TAOK1\_HUMAN was not found.

### CDK5\_HUMAN → ATM\_HUMAN / CDK5\_HUMAN ⊣ ATM\_HUMAN

#### 1. Resources that have the edge

- phosphositeplus-kinase-substrate
  [+]

  Resource paths:
  - Q00535 {CDK5\_HUMAN} (Protein) → Q13315 {ATM\_HUMAN} (Protein) [ProteinProteinInteraction (unknown sign)]

#### 2. Resources that contain endpoints (but not an edge)


#### 3. Resources that match only one endpoint (fringe)


#### 4. Resources that don't match any endpoint

- pid-erbb1
  [+]

  No paths found.
  - CDK5\_HUMAN was not found.
  - ATM\_HUMAN was not found.
- layek
  [+]

  No paths found.
  - CDK5\_HUMAN was not found.
  - ATM\_HUMAN was not found.
- cancer-cell-map-egfr1
  [+]

  No paths found.
  - CDK5\_HUMAN was not found.
  - ATM\_HUMAN was not found.
- kegg-erbb
  [+]

  No paths found.
  - CDK5\_HUMAN was not found.
  - ATM\_HUMAN was not found.
- reactome-egfr
  [+]

  No paths found.
  - CDK5\_HUMAN was not found.
  - ATM\_HUMAN was not found.
- kegg-mapk
  [+]

  No paths found.
  - CDK5\_HUMAN was not found.
  - ATM\_HUMAN was not found.
- science-signaling-egfr
  [+]

  No paths found.
  - CDK5\_HUMAN was not found.
  - ATM\_HUMAN was not found.
- biocarta-egf
  [+]

  No paths found.
  - CDK5\_HUMAN was not found.
  - ATM\_HUMAN was not found.

### CDK1\_HUMAN ⊣ DDX3X\_HUMAN

#### 1. Resources that have the edge

- phosphositeplus-kinase-substrate
  [+]

  Resource paths:
  - P06493 {CDK1\_HUMAN} (Protein) → O00571 {DDX3X\_HUMAN} (Protein) [ProteinProteinInteraction (unknown sign)]

#### 2. Resources that contain endpoints (but not an edge)


#### 3. Resources that match only one endpoint (fringe)


#### 4. Resources that don't match any endpoint

- pid-erbb1
  [+]

  No paths found.
  - CDK1\_HUMAN was not found.
  - DDX3X\_HUMAN was not found.
- cancer-cell-map-egfr1
  [+]

  No paths found.
  - CDK1\_HUMAN was not found.
  - DDX3X\_HUMAN was not found.
- kegg-mapk
  [+]

  No paths found.
  - CDK1\_HUMAN was not found.
  - DDX3X\_HUMAN was not found.
- kegg-erbb
  [+]

  No paths found.
  - CDK1\_HUMAN was not found.
  - DDX3X\_HUMAN was not found.
- biocarta-egf
  [+]

  No paths found.
  - CDK1\_HUMAN was not found.
  - DDX3X\_HUMAN was not found.
- science-signaling-egfr
  [+]

  No paths found.
  - CDK1\_HUMAN was not found.
  - DDX3X\_HUMAN was not found.
- reactome-egfr
  [+]

  No paths found.
  - CDK1\_HUMAN was not found.
  - DDX3X\_HUMAN was not found.
- layek
  [+]

  No paths found.
  - CDK1\_HUMAN was not found.
  - DDX3X\_HUMAN was not found.

### CDK1\_HUMAN → KI67\_HUMAN / CDK1\_HUMAN ⊣ KI67\_HUMAN

#### 1. Resources that have the edge

- phosphositeplus-kinase-substrate
  [+]

  Resource paths:
  - P06493 {CDK1\_HUMAN} (Protein) → P46013 {KI67\_HUMAN} (Protein) [ProteinProteinInteraction (unknown sign)]

#### 2. Resources that contain endpoints (but not an edge)


#### 3. Resources that match only one endpoint (fringe)


#### 4. Resources that don't match any endpoint

- biocarta-egf
  [+]

  No paths found.
  - CDK1\_HUMAN was not found.
  - KI67\_HUMAN was not found.
- pid-erbb1
  [+]

  No paths found.
  - CDK1\_HUMAN was not found.
  - KI67\_HUMAN was not found.
- layek
  [+]

  No paths found.
  - CDK1\_HUMAN was not found.
  - KI67\_HUMAN was not found.
- kegg-erbb
  [+]

  No paths found.
  - CDK1\_HUMAN was not found.
  - KI67\_HUMAN was not found.
- kegg-mapk
  [+]

  No paths found.
  - CDK1\_HUMAN was not found.
  - KI67\_HUMAN was not found.
- science-signaling-egfr
  [+]

  No paths found.
  - CDK1\_HUMAN was not found.
  - KI67\_HUMAN was not found.
- reactome-egfr
  [+]

  No paths found.
  - CDK1\_HUMAN was not found.
  - KI67\_HUMAN was not found.
- cancer-cell-map-egfr1
  [+]

  No paths found.
  - CDK1\_HUMAN was not found.
  - KI67\_HUMAN was not found.

### CSK21\_HUMAN → LA\_HUMAN / CSK21\_HUMAN ⊣ LA\_HUMAN

#### 1. Resources that have the edge

- phosphositeplus-kinase-substrate
  [+]

  Resource paths:
  - P68400 {CSK21\_HUMAN} (Protein) → P05455 {LA\_HUMAN} (Protein) [ProteinProteinInteraction (unknown sign)]

#### 2. Resources that contain endpoints (but not an edge)


#### 3. Resources that match only one endpoint (fringe)


#### 4. Resources that don't match any endpoint

- reactome-egfr
  [+]

  No paths found.
  - CSK21\_HUMAN was not found.
  - LA\_HUMAN was not found.
- kegg-mapk
  [+]

  No paths found.
  - CSK21\_HUMAN was not found.
  - LA\_HUMAN was not found.
- pid-erbb1
  [+]

  No paths found.
  - CSK21\_HUMAN was not found.
  - LA\_HUMAN was not found.
- cancer-cell-map-egfr1
  [+]

  No paths found.
  - CSK21\_HUMAN was not found.
  - LA\_HUMAN was not found.
- biocarta-egf
  [+]

  No paths found.
  - CSK21\_HUMAN was not found.
  - LA\_HUMAN was not found.
- science-signaling-egfr
  [+]

  No paths found.
  - CSK21\_HUMAN was not found.
  - LA\_HUMAN was not found.
- layek
  [+]

  No paths found.
  - CSK21\_HUMAN was not found.
  - LA\_HUMAN was not found.
- kegg-erbb
  [+]

  No paths found.
  - CSK21\_HUMAN was not found.
  - LA\_HUMAN was not found.

### CDK1\_HUMAN → GAPD1\_HUMAN / CDK1\_HUMAN ⊣ GAPD1\_HUMAN

#### 1. Resources that have the edge

- phosphositeplus-kinase-substrate
  [+]

  Resource paths:
  - P06493 {CDK1\_HUMAN} (Protein) → Q14C86 {GAPD1\_HUMAN} (Protein) [ProteinProteinInteraction (unknown sign)]

#### 2. Resources that contain endpoints (but not an edge)


#### 3. Resources that match only one endpoint (fringe)


#### 4. Resources that don't match any endpoint

- reactome-egfr
  [+]

  No paths found.
  - CDK1\_HUMAN was not found.
  - GAPD1\_HUMAN was not found.
- kegg-mapk
  [+]

  No paths found.
  - CDK1\_HUMAN was not found.
  - GAPD1\_HUMAN was not found.
- cancer-cell-map-egfr1
  [+]

  No paths found.
  - CDK1\_HUMAN was not found.
  - GAPD1\_HUMAN was not found.
- kegg-erbb
  [+]

  No paths found.
  - CDK1\_HUMAN was not found.
  - GAPD1\_HUMAN was not found.
- pid-erbb1
  [+]

  No paths found.
  - CDK1\_HUMAN was not found.
  - GAPD1\_HUMAN was not found.
- layek
  [+]

  No paths found.
  - CDK1\_HUMAN was not found.
  - GAPD1\_HUMAN was not found.
- biocarta-egf
  [+]

  No paths found.
  - CDK1\_HUMAN was not found.
  - GAPD1\_HUMAN was not found.
- science-signaling-egfr
  [+]

  No paths found.
  - CDK1\_HUMAN was not found.
  - GAPD1\_HUMAN was not found.

### CDK1\_HUMAN ⊣ STMN1\_HUMAN

#### 1. Resources that have the edge

- phosphositeplus-kinase-substrate
  [+]

  Resource paths:
  - P06493 {CDK1\_HUMAN} (Protein) → P16949 {STMN1\_HUMAN} (Protein) [ProteinProteinInteraction (unknown sign)]

#### 2. Resources that contain endpoints (but not an edge)


#### 3. Resources that match only one endpoint (fringe)


#### 4. Resources that don't match any endpoint

- biocarta-egf
  [+]

  No paths found.
  - CDK1\_HUMAN was not found.
  - STMN1\_HUMAN was not found.
- pid-erbb1
  [+]

  No paths found.
  - CDK1\_HUMAN was not found.
  - STMN1\_HUMAN was not found.
- layek
  [+]

  No paths found.
  - CDK1\_HUMAN was not found.
  - STMN1\_HUMAN was not found.
- kegg-mapk
  [+]

  No paths found.
  - CDK1\_HUMAN was not found.
  - STMN1\_HUMAN was not found.
- cancer-cell-map-egfr1
  [+]

  No paths found.
  - CDK1\_HUMAN was not found.
  - STMN1\_HUMAN was not found.
- science-signaling-egfr
  [+]

  No paths found.
  - CDK1\_HUMAN was not found.
  - STMN1\_HUMAN was not found.
- kegg-erbb
  [+]

  No paths found.
  - CDK1\_HUMAN was not found.
  - STMN1\_HUMAN was not found.
- reactome-egfr
  [+]

  No paths found.
  - CDK1\_HUMAN was not found.
  - STMN1\_HUMAN was not found.

### CDK1\_HUMAN → APC1\_HUMAN / CDK1\_HUMAN ⊣ APC1\_HUMAN

#### 1. Resources that have the edge

- phosphositeplus-kinase-substrate
  [+]

  Resource paths:
  - P06493 {CDK1\_HUMAN} (Protein) → Q9H1A4 {APC1\_HUMAN} (Protein) [ProteinProteinInteraction (unknown sign)]

#### 2. Resources that contain endpoints (but not an edge)


#### 3. Resources that match only one endpoint (fringe)


#### 4. Resources that don't match any endpoint

- kegg-erbb
  [+]

  No paths found.
  - CDK1\_HUMAN was not found.
  - APC1\_HUMAN was not found.
- kegg-mapk
  [+]

  No paths found.
  - CDK1\_HUMAN was not found.
  - APC1\_HUMAN was not found.
- layek
  [+]

  No paths found.
  - CDK1\_HUMAN was not found.
  - APC1\_HUMAN was not found.
- reactome-egfr
  [+]

  No paths found.
  - CDK1\_HUMAN was not found.
  - APC1\_HUMAN was not found.
- pid-erbb1
  [+]

  No paths found.
  - CDK1\_HUMAN was not found.
  - APC1\_HUMAN was not found.
- biocarta-egf
  [+]

  No paths found.
  - CDK1\_HUMAN was not found.
  - APC1\_HUMAN was not found.
- cancer-cell-map-egfr1
  [+]

  No paths found.
  - CDK1\_HUMAN was not found.
  - APC1\_HUMAN was not found.
- science-signaling-egfr
  [+]

  No paths found.
  - CDK1\_HUMAN was not found.
  - APC1\_HUMAN was not found.

### MARK2\_HUMAN → PARD3\_HUMAN / MARK2\_HUMAN ⊣ PARD3\_HUMAN

#### 1. Resources that have the edge

- phosphositeplus-kinase-substrate
  [+]

  Resource paths:
  - Q7KZI7 {MARK2\_HUMAN} (Protein) → Q8TEW0 {PARD3\_HUMAN} (Protein) [ProteinProteinInteraction (unknown sign)]

#### 2. Resources that contain endpoints (but not an edge)


#### 3. Resources that match only one endpoint (fringe)


#### 4. Resources that don't match any endpoint

- reactome-egfr
  [+]

  No paths found.
  - MARK2\_HUMAN was not found.
  - PARD3\_HUMAN was not found.
- science-signaling-egfr
  [+]

  No paths found.
  - MARK2\_HUMAN was not found.
  - PARD3\_HUMAN was not found.
- cancer-cell-map-egfr1
  [+]

  No paths found.
  - MARK2\_HUMAN was not found.
  - PARD3\_HUMAN was not found.
- biocarta-egf
  [+]

  No paths found.
  - MARK2\_HUMAN was not found.
  - PARD3\_HUMAN was not found.
- kegg-mapk
  [+]

  No paths found.
  - MARK2\_HUMAN was not found.
  - PARD3\_HUMAN was not found.
- pid-erbb1
  [+]

  No paths found.
  - MARK2\_HUMAN was not found.
  - PARD3\_HUMAN was not found.
- kegg-erbb
  [+]

  No paths found.
  - MARK2\_HUMAN was not found.
  - PARD3\_HUMAN was not found.
- layek
  [+]

  No paths found.
  - MARK2\_HUMAN was not found.
  - PARD3\_HUMAN was not found.

### CDK1\_HUMAN → LMNA\_HUMAN / CDK1\_HUMAN ⊣ LMNA\_HUMAN

#### 1. Resources that have the edge

- phosphositeplus-kinase-substrate
  [+]

  Resource paths:
  - P06493 {CDK1\_HUMAN} (Protein) → P02545 {LMNA\_HUMAN} (Protein) [ProteinProteinInteraction (unknown sign)]

#### 2. Resources that contain endpoints (but not an edge)


#### 3. Resources that match only one endpoint (fringe)


#### 4. Resources that don't match any endpoint

- reactome-egfr
  [+]

  No paths found.
  - CDK1\_HUMAN was not found.
  - LMNA\_HUMAN was not found.
- science-signaling-egfr
  [+]

  No paths found.
  - CDK1\_HUMAN was not found.
  - LMNA\_HUMAN was not found.
- biocarta-egf
  [+]

  No paths found.
  - CDK1\_HUMAN was not found.
  - LMNA\_HUMAN was not found.
- cancer-cell-map-egfr1
  [+]

  No paths found.
  - CDK1\_HUMAN was not found.
  - LMNA\_HUMAN was not found.
- layek
  [+]

  No paths found.
  - CDK1\_HUMAN was not found.
  - LMNA\_HUMAN was not found.
- kegg-erbb
  [+]

  No paths found.
  - CDK1\_HUMAN was not found.
  - LMNA\_HUMAN was not found.
- pid-erbb1
  [+]

  No paths found.
  - CDK1\_HUMAN was not found.
  - LMNA\_HUMAN was not found.
- kegg-mapk
  [+]

  No paths found.
  - CDK1\_HUMAN was not found.
  - LMNA\_HUMAN was not found.

### CDK1\_HUMAN → PTN2\_HUMAN / CDK1\_HUMAN ⊣ PTN2\_HUMAN

#### 1. Resources that have the edge

- phosphositeplus-kinase-substrate
  [+]

  Resource paths:
  - P06493 {CDK1\_HUMAN} (Protein) → P17706 {PTN2\_HUMAN} (Protein) [ProteinProteinInteraction (unknown sign)]

#### 2. Resources that contain endpoints (but not an edge)


#### 3. Resources that match only one endpoint (fringe)


#### 4. Resources that don't match any endpoint

- pid-erbb1
  [+]

  No paths found.
  - CDK1\_HUMAN was not found.
  - PTN2\_HUMAN was not found.
- biocarta-egf
  [+]

  No paths found.
  - CDK1\_HUMAN was not found.
  - PTN2\_HUMAN was not found.
- kegg-mapk
  [+]

  No paths found.
  - CDK1\_HUMAN was not found.
  - PTN2\_HUMAN was not found.
- cancer-cell-map-egfr1
  [+]

  No paths found.
  - CDK1\_HUMAN was not found.
  - PTN2\_HUMAN was not found.
- science-signaling-egfr
  [+]

  No paths found.
  - CDK1\_HUMAN was not found.
  - PTN2\_HUMAN was not found.
- reactome-egfr
  [+]

  No paths found.
  - CDK1\_HUMAN was not found.
  - PTN2\_HUMAN was not found.
- kegg-erbb
  [+]

  No paths found.
  - CDK1\_HUMAN was not found.
  - PTN2\_HUMAN was not found.
- layek
  [+]

  No paths found.
  - CDK1\_HUMAN was not found.
  - PTN2\_HUMAN was not found.

### CSK21\_HUMAN → DDX58\_HUMAN / CSK21\_HUMAN ⊣ DDX58\_HUMAN

#### 1. Resources that have the edge

- phosphositeplus-kinase-substrate
  [+]

  Resource paths:
  - P68400 {CSK21\_HUMAN} (Protein) → O95786 {DDX58\_HUMAN} (Protein) [ProteinProteinInteraction (unknown sign)]

#### 2. Resources that contain endpoints (but not an edge)


#### 3. Resources that match only one endpoint (fringe)


#### 4. Resources that don't match any endpoint

- reactome-egfr
  [+]

  No paths found.
  - CSK21\_HUMAN was not found.
  - DDX58\_HUMAN was not found.
- kegg-erbb
  [+]

  No paths found.
  - CSK21\_HUMAN was not found.
  - DDX58\_HUMAN was not found.
- science-signaling-egfr
  [+]

  No paths found.
  - CSK21\_HUMAN was not found.
  - DDX58\_HUMAN was not found.
- cancer-cell-map-egfr1
  [+]

  No paths found.
  - CSK21\_HUMAN was not found.
  - DDX58\_HUMAN was not found.
- kegg-mapk
  [+]

  No paths found.
  - CSK21\_HUMAN was not found.
  - DDX58\_HUMAN was not found.
- pid-erbb1
  [+]

  No paths found.
  - CSK21\_HUMAN was not found.
  - DDX58\_HUMAN was not found.
- biocarta-egf
  [+]

  No paths found.
  - CSK21\_HUMAN was not found.
  - DDX58\_HUMAN was not found.
- layek
  [+]

  No paths found.
  - CSK21\_HUMAN was not found.
  - DDX58\_HUMAN was not found.

### CSK21\_HUMAN → MPRI\_HUMAN / CSK21\_HUMAN ⊣ MPRI\_HUMAN

#### 1. Resources that have the edge

- phosphositeplus-kinase-substrate
  [+]

  Resource paths:
  - P68400 {CSK21\_HUMAN} (Protein) → P11717 {MPRI\_HUMAN} (Protein) [ProteinProteinInteraction (unknown sign)]

#### 2. Resources that contain endpoints (but not an edge)


#### 3. Resources that match only one endpoint (fringe)


#### 4. Resources that don't match any endpoint

- pid-erbb1
  [+]

  No paths found.
  - CSK21\_HUMAN was not found.
  - MPRI\_HUMAN was not found.
- kegg-mapk
  [+]

  No paths found.
  - CSK21\_HUMAN was not found.
  - MPRI\_HUMAN was not found.
- layek
  [+]

  No paths found.
  - CSK21\_HUMAN was not found.
  - MPRI\_HUMAN was not found.
- biocarta-egf
  [+]

  No paths found.
  - CSK21\_HUMAN was not found.
  - MPRI\_HUMAN was not found.
- cancer-cell-map-egfr1
  [+]

  No paths found.
  - CSK21\_HUMAN was not found.
  - MPRI\_HUMAN was not found.
- science-signaling-egfr
  [+]

  No paths found.
  - CSK21\_HUMAN was not found.
  - MPRI\_HUMAN was not found.
- kegg-erbb
  [+]

  No paths found.
  - CSK21\_HUMAN was not found.
  - MPRI\_HUMAN was not found.
- reactome-egfr
  [+]

  No paths found.
  - CSK21\_HUMAN was not found.
  - MPRI\_HUMAN was not found.

### ATM\_HUMAN → SMC3\_HUMAN / ATM\_HUMAN ⊣ SMC3\_HUMAN

#### 1. Resources that have the edge

- phosphositeplus-kinase-substrate
  [+]

  Resource paths:
  - Q13315 {ATM\_HUMAN} (Protein) → Q9UQE7 {SMC3\_HUMAN} (Protein) [ProteinProteinInteraction (unknown sign)]

#### 2. Resources that contain endpoints (but not an edge)


#### 3. Resources that match only one endpoint (fringe)


#### 4. Resources that don't match any endpoint

- biocarta-egf
  [+]

  No paths found.
  - ATM\_HUMAN was not found.
  - SMC3\_HUMAN was not found.
- science-signaling-egfr
  [+]

  No paths found.
  - ATM\_HUMAN was not found.
  - SMC3\_HUMAN was not found.
- layek
  [+]

  No paths found.
  - ATM\_HUMAN was not found.
  - SMC3\_HUMAN was not found.
- kegg-erbb
  [+]

  No paths found.
  - ATM\_HUMAN was not found.
  - SMC3\_HUMAN was not found.
- cancer-cell-map-egfr1
  [+]

  No paths found.
  - ATM\_HUMAN was not found.
  - SMC3\_HUMAN was not found.
- reactome-egfr
  [+]

  No paths found.
  - ATM\_HUMAN was not found.
  - SMC3\_HUMAN was not found.
- pid-erbb1
  [+]

  No paths found.
  - ATM\_HUMAN was not found.
  - SMC3\_HUMAN was not found.
- kegg-mapk
  [+]

  No paths found.
  - ATM\_HUMAN was not found.
  - SMC3\_HUMAN was not found.

### CSK21\_HUMAN → TELO2\_HUMAN / CSK21\_HUMAN ⊣ TELO2\_HUMAN

#### 1. Resources that have the edge

- phosphositeplus-kinase-substrate
  [+]

  Resource paths:
  - P68400 {CSK21\_HUMAN} (Protein) → Q9Y4R8 {TELO2\_HUMAN} (Protein) [ProteinProteinInteraction (unknown sign)]

#### 2. Resources that contain endpoints (but not an edge)


#### 3. Resources that match only one endpoint (fringe)


#### 4. Resources that don't match any endpoint

- cancer-cell-map-egfr1
  [+]

  No paths found.
  - CSK21\_HUMAN was not found.
  - TELO2\_HUMAN was not found.
- pid-erbb1
  [+]

  No paths found.
  - CSK21\_HUMAN was not found.
  - TELO2\_HUMAN was not found.
- layek
  [+]

  No paths found.
  - CSK21\_HUMAN was not found.
  - TELO2\_HUMAN was not found.
- reactome-egfr
  [+]

  No paths found.
  - CSK21\_HUMAN was not found.
  - TELO2\_HUMAN was not found.
- kegg-erbb
  [+]

  No paths found.
  - CSK21\_HUMAN was not found.
  - TELO2\_HUMAN was not found.
- kegg-mapk
  [+]

  No paths found.
  - CSK21\_HUMAN was not found.
  - TELO2\_HUMAN was not found.
- biocarta-egf
  [+]

  No paths found.
  - CSK21\_HUMAN was not found.
  - TELO2\_HUMAN was not found.
- science-signaling-egfr
  [+]

  No paths found.
  - CSK21\_HUMAN was not found.
  - TELO2\_HUMAN was not found.

### CDK1\_HUMAN → DNM1L\_HUMAN

#### 1. Resources that have the edge

- phosphositeplus-kinase-substrate
  [+]

  Resource paths:
  - P06493 {CDK1\_HUMAN} (Protein) → O00429 {DNM1L\_HUMAN} (Protein) [ProteinProteinInteraction (unknown sign)]

#### 2. Resources that contain endpoints (but not an edge)


#### 3. Resources that match only one endpoint (fringe)


#### 4. Resources that don't match any endpoint

- kegg-mapk
  [+]

  No paths found.
  - CDK1\_HUMAN was not found.
  - DNM1L\_HUMAN was not found.
- layek
  [+]

  No paths found.
  - CDK1\_HUMAN was not found.
  - DNM1L\_HUMAN was not found.
- reactome-egfr
  [+]

  No paths found.
  - CDK1\_HUMAN was not found.
  - DNM1L\_HUMAN was not found.
- pid-erbb1
  [+]

  No paths found.
  - CDK1\_HUMAN was not found.
  - DNM1L\_HUMAN was not found.
- biocarta-egf
  [+]

  No paths found.
  - CDK1\_HUMAN was not found.
  - DNM1L\_HUMAN was not found.
- science-signaling-egfr
  [+]

  No paths found.
  - CDK1\_HUMAN was not found.
  - DNM1L\_HUMAN was not found.
- cancer-cell-map-egfr1
  [+]

  No paths found.
  - CDK1\_HUMAN was not found.
  - DNM1L\_HUMAN was not found.
- kegg-erbb
  [+]

  No paths found.
  - CDK1\_HUMAN was not found.
  - DNM1L\_HUMAN was not found.

### CDK1\_HUMAN → PUR6\_HUMAN

#### 1. Resources that have the edge

- phosphositeplus-kinase-substrate
  [+]

  Resource paths:
  - P06493 {CDK1\_HUMAN} (Protein) → P22234 {PUR6\_HUMAN} (Protein) [ProteinProteinInteraction (unknown sign)]

#### 2. Resources that contain endpoints (but not an edge)


#### 3. Resources that match only one endpoint (fringe)


#### 4. Resources that don't match any endpoint

- kegg-erbb
  [+]

  No paths found.
  - CDK1\_HUMAN was not found.
  - PUR6\_HUMAN was not found.
- reactome-egfr
  [+]

  No paths found.
  - CDK1\_HUMAN was not found.
  - PUR6\_HUMAN was not found.
- cancer-cell-map-egfr1
  [+]

  No paths found.
  - CDK1\_HUMAN was not found.
  - PUR6\_HUMAN was not found.
- pid-erbb1
  [+]

  No paths found.
  - CDK1\_HUMAN was not found.
  - PUR6\_HUMAN was not found.
- biocarta-egf
  [+]

  No paths found.
  - CDK1\_HUMAN was not found.
  - PUR6\_HUMAN was not found.
- layek
  [+]

  No paths found.
  - CDK1\_HUMAN was not found.
  - PUR6\_HUMAN was not found.
- kegg-mapk
  [+]

  No paths found.
  - CDK1\_HUMAN was not found.
  - PUR6\_HUMAN was not found.
- science-signaling-egfr
  [+]

  No paths found.
  - CDK1\_HUMAN was not found.
  - PUR6\_HUMAN was not found.

### CSK21\_HUMAN → PIAS1\_HUMAN / CSK21\_HUMAN ⊣ PIAS1\_HUMAN

#### 1. Resources that have the edge

- phosphositeplus-kinase-substrate
  [+]

  Resource paths:
  - P68400 {CSK21\_HUMAN} (Protein) → O75925 {PIAS1\_HUMAN} (Protein) [ProteinProteinInteraction (unknown sign)]

#### 2. Resources that contain endpoints (but not an edge)


#### 3. Resources that match only one endpoint (fringe)


#### 4. Resources that don't match any endpoint

- kegg-erbb
  [+]

  No paths found.
  - CSK21\_HUMAN was not found.
  - PIAS1\_HUMAN was not found.
- cancer-cell-map-egfr1
  [+]

  No paths found.
  - CSK21\_HUMAN was not found.
  - PIAS1\_HUMAN was not found.
- kegg-mapk
  [+]

  No paths found.
  - CSK21\_HUMAN was not found.
  - PIAS1\_HUMAN was not found.
- biocarta-egf
  [+]

  No paths found.
  - CSK21\_HUMAN was not found.
  - PIAS1\_HUMAN was not found.
- layek
  [+]

  No paths found.
  - CSK21\_HUMAN was not found.
  - PIAS1\_HUMAN was not found.
- pid-erbb1
  [+]

  No paths found.
  - CSK21\_HUMAN was not found.
  - PIAS1\_HUMAN was not found.
- reactome-egfr
  [+]

  No paths found.
  - CSK21\_HUMAN was not found.
  - PIAS1\_HUMAN was not found.
- science-signaling-egfr
  [+]

  No paths found.
  - CSK21\_HUMAN was not found.
  - PIAS1\_HUMAN was not found.

### CDK5\_HUMAN → DNMT1\_HUMAN / CDK5\_HUMAN ⊣ DNMT1\_HUMAN

#### 1. Resources that have the edge

- phosphositeplus-kinase-substrate
  [+]

  Resource paths:
  - Q00535 {CDK5\_HUMAN} (Protein) → P26358 {DNMT1\_HUMAN} (Protein) [ProteinProteinInteraction (unknown sign)]

#### 2. Resources that contain endpoints (but not an edge)


#### 3. Resources that match only one endpoint (fringe)


#### 4. Resources that don't match any endpoint

- science-signaling-egfr
  [+]

  No paths found.
  - CDK5\_HUMAN was not found.
  - DNMT1\_HUMAN was not found.
- biocarta-egf
  [+]

  No paths found.
  - CDK5\_HUMAN was not found.
  - DNMT1\_HUMAN was not found.
- reactome-egfr
  [+]

  No paths found.
  - CDK5\_HUMAN was not found.
  - DNMT1\_HUMAN was not found.
- kegg-mapk
  [+]

  No paths found.
  - CDK5\_HUMAN was not found.
  - DNMT1\_HUMAN was not found.
- cancer-cell-map-egfr1
  [+]

  No paths found.
  - CDK5\_HUMAN was not found.
  - DNMT1\_HUMAN was not found.
- layek
  [+]

  No paths found.
  - CDK5\_HUMAN was not found.
  - DNMT1\_HUMAN was not found.
- kegg-erbb
  [+]

  No paths found.
  - CDK5\_HUMAN was not found.
  - DNMT1\_HUMAN was not found.
- pid-erbb1
  [+]

  No paths found.
  - CDK5\_HUMAN was not found.
  - DNMT1\_HUMAN was not found.

### CSK21\_HUMAN → MYH10\_HUMAN / CSK21\_HUMAN ⊣ MYH10\_HUMAN

#### 1. Resources that have the edge

- phosphositeplus-kinase-substrate
  [+]

  Resource paths:
  - P68400 {CSK21\_HUMAN} (Protein) → P35580 {MYH10\_HUMAN} (Protein) [ProteinProteinInteraction (unknown sign)]

#### 2. Resources that contain endpoints (but not an edge)


#### 3. Resources that match only one endpoint (fringe)


#### 4. Resources that don't match any endpoint

- biocarta-egf
  [+]

  No paths found.
  - CSK21\_HUMAN was not found.
  - MYH10\_HUMAN was not found.
- science-signaling-egfr
  [+]

  No paths found.
  - CSK21\_HUMAN was not found.
  - MYH10\_HUMAN was not found.
- cancer-cell-map-egfr1
  [+]

  No paths found.
  - CSK21\_HUMAN was not found.
  - MYH10\_HUMAN was not found.
- pid-erbb1
  [+]

  No paths found.
  - CSK21\_HUMAN was not found.
  - MYH10\_HUMAN was not found.
- layek
  [+]

  No paths found.
  - CSK21\_HUMAN was not found.
  - MYH10\_HUMAN was not found.
- kegg-mapk
  [+]

  No paths found.
  - CSK21\_HUMAN was not found.
  - MYH10\_HUMAN was not found.
- kegg-erbb
  [+]

  No paths found.
  - CSK21\_HUMAN was not found.
  - MYH10\_HUMAN was not found.
- reactome-egfr
  [+]

  No paths found.
  - CSK21\_HUMAN was not found.
  - MYH10\_HUMAN was not found.

### CDK1\_HUMAN → DNLI1\_HUMAN / CDK1\_HUMAN ⊣ DNLI1\_HUMAN

#### 1. Resources that have the edge

- phosphositeplus-kinase-substrate
  [+]

  Resource paths:
  - P06493 {CDK1\_HUMAN} (Protein) → P18858 {DNLI1\_HUMAN} (Protein) [ProteinProteinInteraction (unknown sign)]

#### 2. Resources that contain endpoints (but not an edge)


#### 3. Resources that match only one endpoint (fringe)


#### 4. Resources that don't match any endpoint

- pid-erbb1
  [+]

  No paths found.
  - CDK1\_HUMAN was not found.
  - DNLI1\_HUMAN was not found.
- reactome-egfr
  [+]

  No paths found.
  - CDK1\_HUMAN was not found.
  - DNLI1\_HUMAN was not found.
- layek
  [+]

  No paths found.
  - CDK1\_HUMAN was not found.
  - DNLI1\_HUMAN was not found.
- kegg-erbb
  [+]

  No paths found.
  - CDK1\_HUMAN was not found.
  - DNLI1\_HUMAN was not found.
- biocarta-egf
  [+]

  No paths found.
  - CDK1\_HUMAN was not found.
  - DNLI1\_HUMAN was not found.
- science-signaling-egfr
  [+]

  No paths found.
  - CDK1\_HUMAN was not found.
  - DNLI1\_HUMAN was not found.
- cancer-cell-map-egfr1
  [+]

  No paths found.
  - CDK1\_HUMAN was not found.
  - DNLI1\_HUMAN was not found.
- kegg-mapk
  [+]

  No paths found.
  - CDK1\_HUMAN was not found.
  - DNLI1\_HUMAN was not found.

### KKCC1\_HUMAN → KCC1A\_HUMAN / KKCC1\_HUMAN ⊣ KCC1A\_HUMAN

#### 1. Resources that have the edge

- phosphositeplus-kinase-substrate
  [+]

  Resource paths:
  - Q8N5S9 {KKCC1\_HUMAN} (Protein) → Q14012 {KCC1A\_HUMAN} (Protein) [ProteinProteinInteraction (unknown sign)]

#### 2. Resources that contain endpoints (but not an edge)


#### 3. Resources that match only one endpoint (fringe)


#### 4. Resources that don't match any endpoint

- reactome-egfr
  [+]

  No paths found.
  - KKCC1\_HUMAN was not found.
  - KCC1A\_HUMAN was not found.
- kegg-mapk
  [+]

  No paths found.
  - KKCC1\_HUMAN was not found.
  - KCC1A\_HUMAN was not found.
- layek
  [+]

  No paths found.
  - KKCC1\_HUMAN was not found.
  - KCC1A\_HUMAN was not found.
- cancer-cell-map-egfr1
  [+]

  No paths found.
  - KKCC1\_HUMAN was not found.
  - KCC1A\_HUMAN was not found.
- science-signaling-egfr
  [+]

  No paths found.
  - KKCC1\_HUMAN was not found.
  - KCC1A\_HUMAN was not found.
- biocarta-egf
  [+]

  No paths found.
  - KKCC1\_HUMAN was not found.
  - KCC1A\_HUMAN was not found.
- kegg-erbb
  [+]

  No paths found.
  - KKCC1\_HUMAN was not found.
  - KCC1A\_HUMAN was not found.
- pid-erbb1
  [+]

  No paths found.
  - KKCC1\_HUMAN was not found.
  - KCC1A\_HUMAN was not found.

### CSK21\_HUMAN → SAT1\_HUMAN / CSK21\_HUMAN ⊣ SAT1\_HUMAN

#### 1. Resources that have the edge

- phosphositeplus-kinase-substrate
  [+]

  Resource paths:
  - P68400 {CSK21\_HUMAN} (Protein) → P21673 {SAT1\_HUMAN} (Protein) [ProteinProteinInteraction (unknown sign)]

#### 2. Resources that contain endpoints (but not an edge)


#### 3. Resources that match only one endpoint (fringe)


#### 4. Resources that don't match any endpoint

- kegg-mapk
  [+]

  No paths found.
  - CSK21\_HUMAN was not found.
  - SAT1\_HUMAN was not found.
- cancer-cell-map-egfr1
  [+]

  No paths found.
  - CSK21\_HUMAN was not found.
  - SAT1\_HUMAN was not found.
- reactome-egfr
  [+]

  No paths found.
  - CSK21\_HUMAN was not found.
  - SAT1\_HUMAN was not found.
- layek
  [+]

  No paths found.
  - CSK21\_HUMAN was not found.
  - SAT1\_HUMAN was not found.
- science-signaling-egfr
  [+]

  No paths found.
  - CSK21\_HUMAN was not found.
  - SAT1\_HUMAN was not found.
- kegg-erbb
  [+]

  No paths found.
  - CSK21\_HUMAN was not found.
  - SAT1\_HUMAN was not found.
- biocarta-egf
  [+]

  No paths found.
  - CSK21\_HUMAN was not found.
  - SAT1\_HUMAN was not found.
- pid-erbb1
  [+]

  No paths found.
  - CSK21\_HUMAN was not found.
  - SAT1\_HUMAN was not found.

### CDK1\_HUMAN ⊣ TOP2B\_HUMAN

#### 1. Resources that have the edge

- phosphositeplus-kinase-substrate
  [+]

  Resource paths:
  - P06493 {CDK1\_HUMAN} (Protein) → Q02880 {TOP2B\_HUMAN} (Protein) [ProteinProteinInteraction (unknown sign)]

#### 2. Resources that contain endpoints (but not an edge)


#### 3. Resources that match only one endpoint (fringe)


#### 4. Resources that don't match any endpoint

- cancer-cell-map-egfr1
  [+]

  No paths found.
  - CDK1\_HUMAN was not found.
  - TOP2B\_HUMAN was not found.
- reactome-egfr
  [+]

  No paths found.
  - CDK1\_HUMAN was not found.
  - TOP2B\_HUMAN was not found.
- pid-erbb1
  [+]

  No paths found.
  - CDK1\_HUMAN was not found.
  - TOP2B\_HUMAN was not found.
- science-signaling-egfr
  [+]

  No paths found.
  - CDK1\_HUMAN was not found.
  - TOP2B\_HUMAN was not found.
- biocarta-egf
  [+]

  No paths found.
  - CDK1\_HUMAN was not found.
  - TOP2B\_HUMAN was not found.
- kegg-erbb
  [+]

  No paths found.
  - CDK1\_HUMAN was not found.
  - TOP2B\_HUMAN was not found.
- layek
  [+]

  No paths found.
  - CDK1\_HUMAN was not found.
  - TOP2B\_HUMAN was not found.
- kegg-mapk
  [+]

  No paths found.
  - CDK1\_HUMAN was not found.
  - TOP2B\_HUMAN was not found.

### CSK21\_HUMAN → SMC3\_HUMAN / CSK21\_HUMAN ⊣ SMC3\_HUMAN

#### 1. Resources that have the edge

- phosphositeplus-kinase-substrate
  [+]

  Resource paths:
  - P68400 {CSK21\_HUMAN} (Protein) → Q9UQE7 {SMC3\_HUMAN} (Protein) [ProteinProteinInteraction (unknown sign)]

#### 2. Resources that contain endpoints (but not an edge)


#### 3. Resources that match only one endpoint (fringe)


#### 4. Resources that don't match any endpoint

- biocarta-egf
  [+]

  No paths found.
  - CSK21\_HUMAN was not found.
  - SMC3\_HUMAN was not found.
- reactome-egfr
  [+]

  No paths found.
  - CSK21\_HUMAN was not found.
  - SMC3\_HUMAN was not found.
- kegg-erbb
  [+]

  No paths found.
  - CSK21\_HUMAN was not found.
  - SMC3\_HUMAN was not found.
- science-signaling-egfr
  [+]

  No paths found.
  - CSK21\_HUMAN was not found.
  - SMC3\_HUMAN was not found.
- pid-erbb1
  [+]

  No paths found.
  - CSK21\_HUMAN was not found.
  - SMC3\_HUMAN was not found.
- cancer-cell-map-egfr1
  [+]

  No paths found.
  - CSK21\_HUMAN was not found.
  - SMC3\_HUMAN was not found.
- layek
  [+]

  No paths found.
  - CSK21\_HUMAN was not found.
  - SMC3\_HUMAN was not found.
- kegg-mapk
  [+]

  No paths found.
  - CSK21\_HUMAN was not found.
  - SMC3\_HUMAN was not found.

### ATM\_HUMAN → HUWE1\_HUMAN / ATM\_HUMAN ⊣ HUWE1\_HUMAN

#### 1. Resources that have the edge

- phosphositeplus-kinase-substrate
  [+]

  Resource paths:
  - Q13315 {ATM\_HUMAN} (Protein) → Q7Z6Z7 {HUWE1\_HUMAN} (Protein) [ProteinProteinInteraction (unknown sign)]

#### 2. Resources that contain endpoints (but not an edge)


#### 3. Resources that match only one endpoint (fringe)


#### 4. Resources that don't match any endpoint

- reactome-egfr
  [+]

  No paths found.
  - ATM\_HUMAN was not found.
  - HUWE1\_HUMAN was not found.
- science-signaling-egfr
  [+]

  No paths found.
  - ATM\_HUMAN was not found.
  - HUWE1\_HUMAN was not found.
- pid-erbb1
  [+]

  No paths found.
  - ATM\_HUMAN was not found.
  - HUWE1\_HUMAN was not found.
- layek
  [+]

  No paths found.
  - ATM\_HUMAN was not found.
  - HUWE1\_HUMAN was not found.
- cancer-cell-map-egfr1
  [+]

  No paths found.
  - ATM\_HUMAN was not found.
  - HUWE1\_HUMAN was not found.
- kegg-mapk
  [+]

  No paths found.
  - ATM\_HUMAN was not found.
  - HUWE1\_HUMAN was not found.
- biocarta-egf
  [+]

  No paths found.
  - ATM\_HUMAN was not found.
  - HUWE1\_HUMAN was not found.
- kegg-erbb
  [+]

  No paths found.
  - ATM\_HUMAN was not found.
  - HUWE1\_HUMAN was not found.

### ATM\_HUMAN → UBP10\_HUMAN / ATM\_HUMAN ⊣ UBP10\_HUMAN

#### 1. Resources that have the edge

- phosphositeplus-kinase-substrate
  [+]

  Resource paths:
  - Q13315 {ATM\_HUMAN} (Protein) → Q14694 {UBP10\_HUMAN} (Protein) [ProteinProteinInteraction (unknown sign)]

#### 2. Resources that contain endpoints (but not an edge)


#### 3. Resources that match only one endpoint (fringe)


#### 4. Resources that don't match any endpoint

- reactome-egfr
  [+]

  No paths found.
  - ATM\_HUMAN was not found.
  - UBP10\_HUMAN was not found.
- layek
  [+]

  No paths found.
  - ATM\_HUMAN was not found.
  - UBP10\_HUMAN was not found.
- kegg-erbb
  [+]

  No paths found.
  - ATM\_HUMAN was not found.
  - UBP10\_HUMAN was not found.
- kegg-mapk
  [+]

  No paths found.
  - ATM\_HUMAN was not found.
  - UBP10\_HUMAN was not found.
- biocarta-egf
  [+]

  No paths found.
  - ATM\_HUMAN was not found.
  - UBP10\_HUMAN was not found.
- science-signaling-egfr
  [+]

  No paths found.
  - ATM\_HUMAN was not found.
  - UBP10\_HUMAN was not found.
- cancer-cell-map-egfr1
  [+]

  No paths found.
  - ATM\_HUMAN was not found.
  - UBP10\_HUMAN was not found.
- pid-erbb1
  [+]

  No paths found.
  - ATM\_HUMAN was not found.
  - UBP10\_HUMAN was not found.

### CSK21\_HUMAN → CDK1\_HUMAN / CSK21\_HUMAN ⊣ CDK1\_HUMAN

#### 1. Resources that have the edge

- phosphositeplus-kinase-substrate
  [+]

  Resource paths:
  - P68400 {CSK21\_HUMAN} (Protein) → P06493 {CDK1\_HUMAN} (Protein) [ProteinProteinInteraction (unknown sign)]
  - P06493 {CDK1\_HUMAN} (Protein) → P68400 {CSK21\_HUMAN} (Protein) [ProteinProteinInteraction (unknown sign)]

#### 2. Resources that contain endpoints (but not an edge)


#### 3. Resources that match only one endpoint (fringe)


#### 4. Resources that don't match any endpoint

- kegg-erbb
  [+]

  No paths found.
  - CSK21\_HUMAN was not found.
  - CDK1\_HUMAN was not found.
- science-signaling-egfr
  [+]

  No paths found.
  - CSK21\_HUMAN was not found.
  - CDK1\_HUMAN was not found.
- layek
  [+]

  No paths found.
  - CSK21\_HUMAN was not found.
  - CDK1\_HUMAN was not found.
- pid-erbb1
  [+]

  No paths found.
  - CSK21\_HUMAN was not found.
  - CDK1\_HUMAN was not found.
- biocarta-egf
  [+]

  No paths found.
  - CSK21\_HUMAN was not found.
  - CDK1\_HUMAN was not found.
- reactome-egfr
  [+]

  No paths found.
  - CSK21\_HUMAN was not found.
  - CDK1\_HUMAN was not found.
- cancer-cell-map-egfr1
  [+]

  No paths found.
  - CSK21\_HUMAN was not found.
  - CDK1\_HUMAN was not found.
- kegg-mapk
  [+]

  No paths found.
  - CSK21\_HUMAN was not found.
  - CDK1\_HUMAN was not found.

### CSK21\_HUMAN → XRCC1\_HUMAN / CSK21\_HUMAN ⊣ XRCC1\_HUMAN

#### 1. Resources that have the edge

- phosphositeplus-kinase-substrate
  [+]

  Resource paths:
  - P68400 {CSK21\_HUMAN} (Protein) → P18887 {XRCC1\_HUMAN} (Protein) [ProteinProteinInteraction (unknown sign)]

#### 2. Resources that contain endpoints (but not an edge)


#### 3. Resources that match only one endpoint (fringe)


#### 4. Resources that don't match any endpoint

- cancer-cell-map-egfr1
  [+]

  No paths found.
  - CSK21\_HUMAN was not found.
  - XRCC1\_HUMAN was not found.
- pid-erbb1
  [+]

  No paths found.
  - CSK21\_HUMAN was not found.
  - XRCC1\_HUMAN was not found.
- kegg-mapk
  [+]

  No paths found.
  - CSK21\_HUMAN was not found.
  - XRCC1\_HUMAN was not found.
- biocarta-egf
  [+]

  No paths found.
  - CSK21\_HUMAN was not found.
  - XRCC1\_HUMAN was not found.
- science-signaling-egfr
  [+]

  No paths found.
  - CSK21\_HUMAN was not found.
  - XRCC1\_HUMAN was not found.
- kegg-erbb
  [+]

  No paths found.
  - CSK21\_HUMAN was not found.
  - XRCC1\_HUMAN was not found.
- reactome-egfr
  [+]

  No paths found.
  - CSK21\_HUMAN was not found.
  - XRCC1\_HUMAN was not found.
- layek
  [+]

  No paths found.
  - CSK21\_HUMAN was not found.
  - XRCC1\_HUMAN was not found.

### CSK21\_HUMAN → MK09\_HUMAN / CSK21\_HUMAN ⊣ MK09\_HUMAN

#### 1. Resources that have the edge

- phosphositeplus-kinase-substrate
  [+]

  Resource paths:
  - P68400 {CSK21\_HUMAN} (Protein) → P45984 {MK09\_HUMAN} (Protein) [ProteinProteinInteraction (unknown sign)]

#### 2. Resources that contain endpoints (but not an edge)


#### 3. Resources that match only one endpoint (fringe)


#### 4. Resources that don't match any endpoint

- cancer-cell-map-egfr1
  [+]

  No paths found.
  - CSK21\_HUMAN was not found.
  - MK09\_HUMAN was not found.
- layek
  [+]

  No paths found.
  - CSK21\_HUMAN was not found.
  - MK09\_HUMAN was not found.
- kegg-mapk
  [+]

  No paths found.
  - CSK21\_HUMAN was not found.
  - MK09\_HUMAN was not found.
- biocarta-egf
  [+]

  No paths found.
  - CSK21\_HUMAN was not found.
  - MK09\_HUMAN was not found.
- pid-erbb1
  [+]

  No paths found.
  - CSK21\_HUMAN was not found.
  - MK09\_HUMAN was not found.
- science-signaling-egfr
  [+]

  No paths found.
  - CSK21\_HUMAN was not found.
  - MK09\_HUMAN was not found.
- kegg-erbb
  [+]

  No paths found.
  - CSK21\_HUMAN was not found.
  - MK09\_HUMAN was not found.
- reactome-egfr
  [+]

  No paths found.
  - CSK21\_HUMAN was not found.
  - MK09\_HUMAN was not found.

### ATM\_HUMAN → FACD2\_HUMAN / ATM\_HUMAN ⊣ FACD2\_HUMAN

#### 1. Resources that have the edge

- phosphositeplus-kinase-substrate
  [+]

  Resource paths:
  - Q13315 {ATM\_HUMAN} (Protein) → Q9BXW9 {FACD2\_HUMAN} (Protein) [ProteinProteinInteraction (unknown sign)]

#### 2. Resources that contain endpoints (but not an edge)


#### 3. Resources that match only one endpoint (fringe)


#### 4. Resources that don't match any endpoint

- layek
  [+]

  No paths found.
  - ATM\_HUMAN was not found.
  - FACD2\_HUMAN was not found.
- cancer-cell-map-egfr1
  [+]

  No paths found.
  - ATM\_HUMAN was not found.
  - FACD2\_HUMAN was not found.
- kegg-mapk
  [+]

  No paths found.
  - ATM\_HUMAN was not found.
  - FACD2\_HUMAN was not found.
- kegg-erbb
  [+]

  No paths found.
  - ATM\_HUMAN was not found.
  - FACD2\_HUMAN was not found.
- biocarta-egf
  [+]

  No paths found.
  - ATM\_HUMAN was not found.
  - FACD2\_HUMAN was not found.
- reactome-egfr
  [+]

  No paths found.
  - ATM\_HUMAN was not found.
  - FACD2\_HUMAN was not found.
- pid-erbb1
  [+]

  No paths found.
  - ATM\_HUMAN was not found.
  - FACD2\_HUMAN was not found.
- science-signaling-egfr
  [+]

  No paths found.
  - ATM\_HUMAN was not found.
  - FACD2\_HUMAN was not found.

### CDK1\_HUMAN → NUCKS\_HUMAN / CDK1\_HUMAN ⊣ NUCKS\_HUMAN

#### 1. Resources that have the edge

- phosphositeplus-kinase-substrate
  [+]

  Resource paths:
  - P06493 {CDK1\_HUMAN} (Protein) → Q9H1E3 {NUCKS\_HUMAN} (Protein) [ProteinProteinInteraction (unknown sign)]

#### 2. Resources that contain endpoints (but not an edge)


#### 3. Resources that match only one endpoint (fringe)


#### 4. Resources that don't match any endpoint

- science-signaling-egfr
  [+]

  No paths found.
  - CDK1\_HUMAN was not found.
  - NUCKS\_HUMAN was not found.
- kegg-erbb
  [+]

  No paths found.
  - CDK1\_HUMAN was not found.
  - NUCKS\_HUMAN was not found.
- pid-erbb1
  [+]

  No paths found.
  - CDK1\_HUMAN was not found.
  - NUCKS\_HUMAN was not found.
- biocarta-egf
  [+]

  No paths found.
  - CDK1\_HUMAN was not found.
  - NUCKS\_HUMAN was not found.
- cancer-cell-map-egfr1
  [+]

  No paths found.
  - CDK1\_HUMAN was not found.
  - NUCKS\_HUMAN was not found.
- layek
  [+]

  No paths found.
  - CDK1\_HUMAN was not found.
  - NUCKS\_HUMAN was not found.
- kegg-mapk
  [+]

  No paths found.
  - CDK1\_HUMAN was not found.
  - NUCKS\_HUMAN was not found.
- reactome-egfr
  [+]

  No paths found.
  - CDK1\_HUMAN was not found.
  - NUCKS\_HUMAN was not found.

### CDK1\_HUMAN ⊣ NSF1C\_HUMAN

#### 1. Resources that have the edge

- phosphositeplus-kinase-substrate
  [+]

  Resource paths:
  - P06493 {CDK1\_HUMAN} (Protein) → Q9UNZ2 {NSF1C\_HUMAN} (Protein) [ProteinProteinInteraction (unknown sign)]

#### 2. Resources that contain endpoints (but not an edge)


#### 3. Resources that match only one endpoint (fringe)


#### 4. Resources that don't match any endpoint

- cancer-cell-map-egfr1
  [+]

  No paths found.
  - CDK1\_HUMAN was not found.
  - NSF1C\_HUMAN was not found.
- pid-erbb1
  [+]

  No paths found.
  - CDK1\_HUMAN was not found.
  - NSF1C\_HUMAN was not found.
- reactome-egfr
  [+]

  No paths found.
  - CDK1\_HUMAN was not found.
  - NSF1C\_HUMAN was not found.
- biocarta-egf
  [+]

  No paths found.
  - CDK1\_HUMAN was not found.
  - NSF1C\_HUMAN was not found.
- layek
  [+]

  No paths found.
  - CDK1\_HUMAN was not found.
  - NSF1C\_HUMAN was not found.
- kegg-erbb
  [+]

  No paths found.
  - CDK1\_HUMAN was not found.
  - NSF1C\_HUMAN was not found.
- science-signaling-egfr
  [+]

  No paths found.
  - CDK1\_HUMAN was not found.
  - NSF1C\_HUMAN was not found.
- kegg-mapk
  [+]

  No paths found.
  - CDK1\_HUMAN was not found.
  - NSF1C\_HUMAN was not found.

### CDK1\_HUMAN → TCOF\_HUMAN / CDK1\_HUMAN ⊣ TCOF\_HUMAN

#### 1. Resources that have the edge

- phosphositeplus-kinase-substrate
  [+]

  Resource paths:
  - P06493 {CDK1\_HUMAN} (Protein) → Q13428 {TCOF\_HUMAN} (Protein) [ProteinProteinInteraction (unknown sign)]

#### 2. Resources that contain endpoints (but not an edge)


#### 3. Resources that match only one endpoint (fringe)


#### 4. Resources that don't match any endpoint

- pid-erbb1
  [+]

  No paths found.
  - CDK1\_HUMAN was not found.
  - TCOF\_HUMAN was not found.
- kegg-erbb
  [+]

  No paths found.
  - CDK1\_HUMAN was not found.
  - TCOF\_HUMAN was not found.
- biocarta-egf
  [+]

  No paths found.
  - CDK1\_HUMAN was not found.
  - TCOF\_HUMAN was not found.
- science-signaling-egfr
  [+]

  No paths found.
  - CDK1\_HUMAN was not found.
  - TCOF\_HUMAN was not found.
- layek
  [+]

  No paths found.
  - CDK1\_HUMAN was not found.
  - TCOF\_HUMAN was not found.
- cancer-cell-map-egfr1
  [+]

  No paths found.
  - CDK1\_HUMAN was not found.
  - TCOF\_HUMAN was not found.
- reactome-egfr
  [+]

  No paths found.
  - CDK1\_HUMAN was not found.
  - TCOF\_HUMAN was not found.
- kegg-mapk
  [+]

  No paths found.
  - CDK1\_HUMAN was not found.
  - TCOF\_HUMAN was not found.

### CSK21\_HUMAN → DNLI1\_HUMAN / CSK21\_HUMAN ⊣ DNLI1\_HUMAN

#### 1. Resources that have the edge

- phosphositeplus-kinase-substrate
  [+]

  Resource paths:
  - P68400 {CSK21\_HUMAN} (Protein) → P18858 {DNLI1\_HUMAN} (Protein) [ProteinProteinInteraction (unknown sign)]

#### 2. Resources that contain endpoints (but not an edge)


#### 3. Resources that match only one endpoint (fringe)


#### 4. Resources that don't match any endpoint

- pid-erbb1
  [+]

  No paths found.
  - CSK21\_HUMAN was not found.
  - DNLI1\_HUMAN was not found.
- reactome-egfr
  [+]

  No paths found.
  - CSK21\_HUMAN was not found.
  - DNLI1\_HUMAN was not found.
- kegg-mapk
  [+]

  No paths found.
  - CSK21\_HUMAN was not found.
  - DNLI1\_HUMAN was not found.
- layek
  [+]

  No paths found.
  - CSK21\_HUMAN was not found.
  - DNLI1\_HUMAN was not found.
- science-signaling-egfr
  [+]

  No paths found.
  - CSK21\_HUMAN was not found.
  - DNLI1\_HUMAN was not found.
- cancer-cell-map-egfr1
  [+]

  No paths found.
  - CSK21\_HUMAN was not found.
  - DNLI1\_HUMAN was not found.
- kegg-erbb
  [+]

  No paths found.
  - CSK21\_HUMAN was not found.
  - DNLI1\_HUMAN was not found.
- biocarta-egf
  [+]

  No paths found.
  - CSK21\_HUMAN was not found.
  - DNLI1\_HUMAN was not found.

### CSK21\_HUMAN → ABCF1\_HUMAN / CSK21\_HUMAN ⊣ ABCF1\_HUMAN

#### 1. Resources that have the edge

- phosphositeplus-kinase-substrate
  [+]

  Resource paths:
  - P68400 {CSK21\_HUMAN} (Protein) → Q8NE71 {ABCF1\_HUMAN} (Protein) [ProteinProteinInteraction (unknown sign)]

#### 2. Resources that contain endpoints (but not an edge)


#### 3. Resources that match only one endpoint (fringe)


#### 4. Resources that don't match any endpoint

- layek
  [+]

  No paths found.
  - CSK21\_HUMAN was not found.
  - ABCF1\_HUMAN was not found.
- reactome-egfr
  [+]

  No paths found.
  - CSK21\_HUMAN was not found.
  - ABCF1\_HUMAN was not found.
- kegg-erbb
  [+]

  No paths found.
  - CSK21\_HUMAN was not found.
  - ABCF1\_HUMAN was not found.
- biocarta-egf
  [+]

  No paths found.
  - CSK21\_HUMAN was not found.
  - ABCF1\_HUMAN was not found.
- science-signaling-egfr
  [+]

  No paths found.
  - CSK21\_HUMAN was not found.
  - ABCF1\_HUMAN was not found.
- kegg-mapk
  [+]

  No paths found.
  - CSK21\_HUMAN was not found.
  - ABCF1\_HUMAN was not found.
- cancer-cell-map-egfr1
  [+]

  No paths found.
  - CSK21\_HUMAN was not found.
  - ABCF1\_HUMAN was not found.
- pid-erbb1
  [+]

  No paths found.
  - CSK21\_HUMAN was not found.
  - ABCF1\_HUMAN was not found.

### CDK1\_HUMAN → 41\_HUMAN / CDK1\_HUMAN ⊣ 41\_HUMAN

#### 1. Resources that have the edge

- phosphositeplus-kinase-substrate
  [+]

  Resource paths:
  - P06493 {CDK1\_HUMAN} (Protein) → P11171 {41\_HUMAN} (Protein) [ProteinProteinInteraction (unknown sign)]

#### 2. Resources that contain endpoints (but not an edge)


#### 3. Resources that match only one endpoint (fringe)


#### 4. Resources that don't match any endpoint

- kegg-mapk
  [+]

  No paths found.
  - CDK1\_HUMAN was not found.
  - 41\_HUMAN was not found.
- science-signaling-egfr
  [+]

  No paths found.
  - CDK1\_HUMAN was not found.
  - 41\_HUMAN was not found.
- pid-erbb1
  [+]

  No paths found.
  - CDK1\_HUMAN was not found.
  - 41\_HUMAN was not found.
- kegg-erbb
  [+]

  No paths found.
  - CDK1\_HUMAN was not found.
  - 41\_HUMAN was not found.
- layek
  [+]

  No paths found.
  - CDK1\_HUMAN was not found.
  - 41\_HUMAN was not found.
- biocarta-egf
  [+]

  No paths found.
  - CDK1\_HUMAN was not found.
  - 41\_HUMAN was not found.
- reactome-egfr
  [+]

  No paths found.
  - CDK1\_HUMAN was not found.
  - 41\_HUMAN was not found.
- cancer-cell-map-egfr1
  [+]

  No paths found.
  - CDK1\_HUMAN was not found.
  - 41\_HUMAN was not found.

### CSK21\_HUMAN → PDIA6\_HUMAN / CSK21\_HUMAN ⊣ PDIA6\_HUMAN

#### 1. Resources that have the edge

- phosphositeplus-kinase-substrate
  [+]

  Resource paths:
  - P68400 {CSK21\_HUMAN} (Protein) → Q15084 {PDIA6\_HUMAN} (Protein) [ProteinProteinInteraction (unknown sign)]

#### 2. Resources that contain endpoints (but not an edge)


#### 3. Resources that match only one endpoint (fringe)


#### 4. Resources that don't match any endpoint

- cancer-cell-map-egfr1
  [+]

  No paths found.
  - CSK21\_HUMAN was not found.
  - PDIA6\_HUMAN was not found.
- layek
  [+]

  No paths found.
  - CSK21\_HUMAN was not found.
  - PDIA6\_HUMAN was not found.
- pid-erbb1
  [+]

  No paths found.
  - CSK21\_HUMAN was not found.
  - PDIA6\_HUMAN was not found.
- science-signaling-egfr
  [+]

  No paths found.
  - CSK21\_HUMAN was not found.
  - PDIA6\_HUMAN was not found.
- reactome-egfr
  [+]

  No paths found.
  - CSK21\_HUMAN was not found.
  - PDIA6\_HUMAN was not found.
- kegg-erbb
  [+]

  No paths found.
  - CSK21\_HUMAN was not found.
  - PDIA6\_HUMAN was not found.
- biocarta-egf
  [+]

  No paths found.
  - CSK21\_HUMAN was not found.
  - PDIA6\_HUMAN was not found.
- kegg-mapk
  [+]

  No paths found.
  - CSK21\_HUMAN was not found.
  - PDIA6\_HUMAN was not found.

### GRB2\_HUMAN → PTPRA\_HUMAN / GRB2\_HUMAN ⊣ PTPRA\_HUMAN

#### 1. Resources that have the edge


#### 2. Resources that contain endpoints (but not an edge)

- phosphositeplus-kinase-substrate
  [+]

  No paths found.

#### 3. Resources that match only one endpoint (fringe)

- kegg-erbb
  [+]

  No paths found.
  - PTPRA\_HUMAN was not found.
- kegg-mapk
  [+]

  No paths found.
  - PTPRA\_HUMAN was not found.
- science-signaling-egfr
  [+]

  No paths found.
  - PTPRA\_HUMAN was not found.
- reactome-egfr
  [+]

  No paths found.
  - PTPRA\_HUMAN was not found.
- cancer-cell-map-egfr1
  [+]

  No paths found.
  - PTPRA\_HUMAN was not found.
- biocarta-egf
  [+]

  No paths found.
  - PTPRA\_HUMAN was not found.
- layek
  [+]

  No paths found.
  - PTPRA\_HUMAN was not found.
- pid-erbb1
  [+]

  No paths found.
  - PTPRA\_HUMAN was not found.

#### 4. Resources that don't match any endpoint


### MK01\_HUMAN ⊣ DYR1B\_HUMAN

#### 1. Resources that have the edge


#### 2. Resources that contain endpoints (but not an edge)

- phosphositeplus-kinase-substrate
  [+]

  No paths found.

#### 3. Resources that match only one endpoint (fringe)

- kegg-mapk
  [+]

  No paths found.
  - DYR1B\_HUMAN was not found.
- kegg-erbb
  [+]

  No paths found.
  - DYR1B\_HUMAN was not found.
- science-signaling-egfr
  [+]

  No paths found.
  - DYR1B\_HUMAN was not found.
- pid-erbb1
  [+]

  No paths found.
  - DYR1B\_HUMAN was not found.
- layek
  [+]

  No paths found.
  - DYR1B\_HUMAN was not found.
- reactome-egfr
  [+]

  No paths found.
  - DYR1B\_HUMAN was not found.
- cancer-cell-map-egfr1
  [+]

  No paths found.
  - DYR1B\_HUMAN was not found.

#### 4. Resources that don't match any endpoint

- biocarta-egf
  [+]

  No paths found.
  - DYR1B\_HUMAN was not found.
  - MK01\_HUMAN was not found.

### MK01\_HUMAN ⊣ ICK\_HUMAN

#### 1. Resources that have the edge


#### 2. Resources that contain endpoints (but not an edge)

- phosphositeplus-kinase-substrate
  [+]

  No paths found.

#### 3. Resources that match only one endpoint (fringe)

- pid-erbb1
  [+]

  No paths found.
  - ICK\_HUMAN was not found.
- reactome-egfr
  [+]

  No paths found.
  - ICK\_HUMAN was not found.
- cancer-cell-map-egfr1
  [+]

  No paths found.
  - ICK\_HUMAN was not found.
- kegg-mapk
  [+]

  No paths found.
  - ICK\_HUMAN was not found.
- layek
  [+]

  No paths found.
  - ICK\_HUMAN was not found.
- kegg-erbb
  [+]

  No paths found.
  - ICK\_HUMAN was not found.
- science-signaling-egfr
  [+]

  No paths found.
  - ICK\_HUMAN was not found.

#### 4. Resources that don't match any endpoint

- biocarta-egf
  [+]

  No paths found.
  - MK01\_HUMAN was not found.
  - ICK\_HUMAN was not found.

### HIPK2\_HUMAN → DYR1B\_HUMAN

#### 1. Resources that have the edge


#### 2. Resources that contain endpoints (but not an edge)

- phosphositeplus-kinase-substrate
  [+]

  No paths found.

#### 3. Resources that match only one endpoint (fringe)


#### 4. Resources that don't match any endpoint

- pid-erbb1
  [+]

  No paths found.
  - DYR1B\_HUMAN was not found.
  - HIPK2\_HUMAN was not found.
- kegg-mapk
  [+]

  No paths found.
  - DYR1B\_HUMAN was not found.
  - HIPK2\_HUMAN was not found.
- science-signaling-egfr
  [+]

  No paths found.
  - DYR1B\_HUMAN was not found.
  - HIPK2\_HUMAN was not found.
- biocarta-egf
  [+]

  No paths found.
  - DYR1B\_HUMAN was not found.
  - HIPK2\_HUMAN was not found.
- reactome-egfr
  [+]

  No paths found.
  - DYR1B\_HUMAN was not found.
  - HIPK2\_HUMAN was not found.
- cancer-cell-map-egfr1
  [+]

  No paths found.
  - DYR1B\_HUMAN was not found.
  - HIPK2\_HUMAN was not found.
- kegg-erbb
  [+]

  No paths found.
  - DYR1B\_HUMAN was not found.
  - HIPK2\_HUMAN was not found.
- layek
  [+]

  No paths found.
  - DYR1B\_HUMAN was not found.
  - HIPK2\_HUMAN was not found.

### SMC3\_HUMAN → NEK6\_HUMAN / SMC3\_HUMAN ⊣ NEK6\_HUMAN

#### 1. Resources that have the edge


#### 2. Resources that contain endpoints (but not an edge)

- phosphositeplus-kinase-substrate
  [+]

  No paths found.

#### 3. Resources that match only one endpoint (fringe)


#### 4. Resources that don't match any endpoint

- reactome-egfr
  [+]

  No paths found.
  - NEK6\_HUMAN was not found.
  - SMC3\_HUMAN was not found.
- kegg-mapk
  [+]

  No paths found.
  - NEK6\_HUMAN was not found.
  - SMC3\_HUMAN was not found.
- pid-erbb1
  [+]

  No paths found.
  - NEK6\_HUMAN was not found.
  - SMC3\_HUMAN was not found.
- layek
  [+]

  No paths found.
  - NEK6\_HUMAN was not found.
  - SMC3\_HUMAN was not found.
- biocarta-egf
  [+]

  No paths found.
  - NEK6\_HUMAN was not found.
  - SMC3\_HUMAN was not found.
- cancer-cell-map-egfr1
  [+]

  No paths found.
  - NEK6\_HUMAN was not found.
  - SMC3\_HUMAN was not found.
- kegg-erbb
  [+]

  No paths found.
  - NEK6\_HUMAN was not found.
  - SMC3\_HUMAN was not found.
- science-signaling-egfr
  [+]

  No paths found.
  - NEK6\_HUMAN was not found.
  - SMC3\_HUMAN was not found.

### 1433G\_HUMAN → LARP1\_HUMAN / 1433G\_HUMAN ⊣ LARP1\_HUMAN

#### 1. Resources that have the edge


#### 2. Resources that contain endpoints (but not an edge)

- phosphositeplus-kinase-substrate
  [+]

  No paths found.

#### 3. Resources that match only one endpoint (fringe)


#### 4. Resources that don't match any endpoint

- science-signaling-egfr
  [+]

  No paths found.
  - 1433G\_HUMAN was not found.
  - LARP1\_HUMAN was not found.
- biocarta-egf
  [+]

  No paths found.
  - 1433G\_HUMAN was not found.
  - LARP1\_HUMAN was not found.
- layek
  [+]

  No paths found.
  - 1433G\_HUMAN was not found.
  - LARP1\_HUMAN was not found.
- cancer-cell-map-egfr1
  [+]

  No paths found.
  - 1433G\_HUMAN was not found.
  - LARP1\_HUMAN was not found.
- reactome-egfr
  [+]

  No paths found.
  - 1433G\_HUMAN was not found.
  - LARP1\_HUMAN was not found.
- kegg-erbb
  [+]

  No paths found.
  - 1433G\_HUMAN was not found.
  - LARP1\_HUMAN was not found.
- kegg-mapk
  [+]

  No paths found.
  - 1433G\_HUMAN was not found.
  - LARP1\_HUMAN was not found.
- pid-erbb1
  [+]

  No paths found.
  - 1433G\_HUMAN was not found.
  - LARP1\_HUMAN was not found.

### DDX3X\_HUMAN → IF4G1\_HUMAN

#### 1. Resources that have the edge


#### 2. Resources that contain endpoints (but not an edge)

- phosphositeplus-kinase-substrate
  [+]

  No paths found.

#### 3. Resources that match only one endpoint (fringe)


#### 4. Resources that don't match any endpoint

- kegg-mapk
  [+]

  No paths found.
  - DDX3X\_HUMAN was not found.
  - IF4G1\_HUMAN was not found.
- pid-erbb1
  [+]

  No paths found.
  - DDX3X\_HUMAN was not found.
  - IF4G1\_HUMAN was not found.
- reactome-egfr
  [+]

  No paths found.
  - DDX3X\_HUMAN was not found.
  - IF4G1\_HUMAN was not found.
- biocarta-egf
  [+]

  No paths found.
  - DDX3X\_HUMAN was not found.
  - IF4G1\_HUMAN was not found.
- science-signaling-egfr
  [+]

  No paths found.
  - DDX3X\_HUMAN was not found.
  - IF4G1\_HUMAN was not found.
- kegg-erbb
  [+]

  No paths found.
  - DDX3X\_HUMAN was not found.
  - IF4G1\_HUMAN was not found.
- cancer-cell-map-egfr1
  [+]

  No paths found.
  - DDX3X\_HUMAN was not found.
  - IF4G1\_HUMAN was not found.
- layek
  [+]

  No paths found.
  - DDX3X\_HUMAN was not found.
  - IF4G1\_HUMAN was not found.

### LMNA\_HUMAN → TOIP1\_HUMAN / LMNA\_HUMAN ⊣ TOIP1\_HUMAN

#### 1. Resources that have the edge


#### 2. Resources that contain endpoints (but not an edge)

- phosphositeplus-kinase-substrate
  [+]

  No paths found.

#### 3. Resources that match only one endpoint (fringe)


#### 4. Resources that don't match any endpoint

- pid-erbb1
  [+]

  No paths found.
  - TOIP1\_HUMAN was not found.
  - LMNA\_HUMAN was not found.
- biocarta-egf
  [+]

  No paths found.
  - TOIP1\_HUMAN was not found.
  - LMNA\_HUMAN was not found.
- cancer-cell-map-egfr1
  [+]

  No paths found.
  - TOIP1\_HUMAN was not found.
  - LMNA\_HUMAN was not found.
- reactome-egfr
  [+]

  No paths found.
  - TOIP1\_HUMAN was not found.
  - LMNA\_HUMAN was not found.
- science-signaling-egfr
  [+]

  No paths found.
  - TOIP1\_HUMAN was not found.
  - LMNA\_HUMAN was not found.
- layek
  [+]

  No paths found.
  - TOIP1\_HUMAN was not found.
  - LMNA\_HUMAN was not found.
- kegg-mapk
  [+]

  No paths found.
  - TOIP1\_HUMAN was not found.
  - LMNA\_HUMAN was not found.
- kegg-erbb
  [+]

  No paths found.
  - TOIP1\_HUMAN was not found.
  - LMNA\_HUMAN was not found.

### SMC3\_HUMAN → PDS5B\_HUMAN / SMC3\_HUMAN ⊣ PDS5B\_HUMAN

#### 1. Resources that have the edge


#### 2. Resources that contain endpoints (but not an edge)

- phosphositeplus-kinase-substrate
  [+]

  No paths found.

#### 3. Resources that match only one endpoint (fringe)


#### 4. Resources that don't match any endpoint

- biocarta-egf
  [+]

  No paths found.
  - SMC3\_HUMAN was not found.
  - PDS5B\_HUMAN was not found.
- pid-erbb1
  [+]

  No paths found.
  - SMC3\_HUMAN was not found.
  - PDS5B\_HUMAN was not found.
- layek
  [+]

  No paths found.
  - SMC3\_HUMAN was not found.
  - PDS5B\_HUMAN was not found.
- cancer-cell-map-egfr1
  [+]

  No paths found.
  - SMC3\_HUMAN was not found.
  - PDS5B\_HUMAN was not found.
- kegg-mapk
  [+]

  No paths found.
  - SMC3\_HUMAN was not found.
  - PDS5B\_HUMAN was not found.
- kegg-erbb
  [+]

  No paths found.
  - SMC3\_HUMAN was not found.
  - PDS5B\_HUMAN was not found.
- reactome-egfr
  [+]

  No paths found.
  - SMC3\_HUMAN was not found.
  - PDS5B\_HUMAN was not found.
- science-signaling-egfr
  [+]

  No paths found.
  - SMC3\_HUMAN was not found.
  - PDS5B\_HUMAN was not found.

### SRRM1\_HUMAN → SRRM2\_HUMAN

#### 1. Resources that have the edge


#### 2. Resources that contain endpoints (but not an edge)

- phosphositeplus-kinase-substrate
  [+]

  No paths found.

#### 3. Resources that match only one endpoint (fringe)


#### 4. Resources that don't match any endpoint

- biocarta-egf
  [+]

  No paths found.
  - SRRM2\_HUMAN was not found.
  - SRRM1\_HUMAN was not found.
- reactome-egfr
  [+]

  No paths found.
  - SRRM2\_HUMAN was not found.
  - SRRM1\_HUMAN was not found.
- science-signaling-egfr
  [+]

  No paths found.
  - SRRM2\_HUMAN was not found.
  - SRRM1\_HUMAN was not found.
- kegg-erbb
  [+]

  No paths found.
  - SRRM2\_HUMAN was not found.
  - SRRM1\_HUMAN was not found.
- pid-erbb1
  [+]

  No paths found.
  - SRRM2\_HUMAN was not found.
  - SRRM1\_HUMAN was not found.
- layek
  [+]

  No paths found.
  - SRRM2\_HUMAN was not found.
  - SRRM1\_HUMAN was not found.
- cancer-cell-map-egfr1
  [+]

  No paths found.
  - SRRM2\_HUMAN was not found.
  - SRRM1\_HUMAN was not found.
- kegg-mapk
  [+]

  No paths found.
  - SRRM2\_HUMAN was not found.
  - SRRM1\_HUMAN was not found.

### 1433G\_HUMAN → AFAD\_HUMAN / 1433G\_HUMAN ⊣ AFAD\_HUMAN

#### 1. Resources that have the edge


#### 2. Resources that contain endpoints (but not an edge)

- phosphositeplus-kinase-substrate
  [+]

  No paths found.

#### 3. Resources that match only one endpoint (fringe)


#### 4. Resources that don't match any endpoint

- science-signaling-egfr
  [+]

  No paths found.
  - 1433G\_HUMAN was not found.
  - AFAD\_HUMAN was not found.
- reactome-egfr
  [+]

  No paths found.
  - 1433G\_HUMAN was not found.
  - AFAD\_HUMAN was not found.
- kegg-mapk
  [+]

  No paths found.
  - 1433G\_HUMAN was not found.
  - AFAD\_HUMAN was not found.
- biocarta-egf
  [+]

  No paths found.
  - 1433G\_HUMAN was not found.
  - AFAD\_HUMAN was not found.
- layek
  [+]

  No paths found.
  - 1433G\_HUMAN was not found.
  - AFAD\_HUMAN was not found.
- cancer-cell-map-egfr1
  [+]

  No paths found.
  - 1433G\_HUMAN was not found.
  - AFAD\_HUMAN was not found.
- pid-erbb1
  [+]

  No paths found.
  - 1433G\_HUMAN was not found.
  - AFAD\_HUMAN was not found.
- kegg-erbb
  [+]

  No paths found.
  - 1433G\_HUMAN was not found.
  - AFAD\_HUMAN was not found.

### CSK21\_HUMAN → IF4B\_HUMAN / CSK21\_HUMAN ⊣ IF4B\_HUMAN

#### 1. Resources that have the edge


#### 2. Resources that contain endpoints (but not an edge)

- phosphositeplus-kinase-substrate
  [+]

  No paths found.

#### 3. Resources that match only one endpoint (fringe)


#### 4. Resources that don't match any endpoint

- biocarta-egf
  [+]

  No paths found.
  - CSK21\_HUMAN was not found.
  - IF4B\_HUMAN was not found.
- kegg-mapk
  [+]

  No paths found.
  - CSK21\_HUMAN was not found.
  - IF4B\_HUMAN was not found.
- cancer-cell-map-egfr1
  [+]

  No paths found.
  - CSK21\_HUMAN was not found.
  - IF4B\_HUMAN was not found.
- layek
  [+]

  No paths found.
  - CSK21\_HUMAN was not found.
  - IF4B\_HUMAN was not found.
- pid-erbb1
  [+]

  No paths found.
  - CSK21\_HUMAN was not found.
  - IF4B\_HUMAN was not found.
- science-signaling-egfr
  [+]

  No paths found.
  - CSK21\_HUMAN was not found.
  - IF4B\_HUMAN was not found.
- kegg-erbb
  [+]

  No paths found.
  - CSK21\_HUMAN was not found.
  - IF4B\_HUMAN was not found.
- reactome-egfr
  [+]

  No paths found.
  - CSK21\_HUMAN was not found.
  - IF4B\_HUMAN was not found.

### AT1A1\_HUMAN ⊣ ADDA\_HUMAN

#### 1. Resources that have the edge


#### 2. Resources that contain endpoints (but not an edge)

- phosphositeplus-kinase-substrate
  [+]

  No paths found.

#### 3. Resources that match only one endpoint (fringe)


#### 4. Resources that don't match any endpoint

- kegg-mapk
  [+]

  No paths found.
  - ADDA\_HUMAN was not found.
  - AT1A1\_HUMAN was not found.
- layek
  [+]

  No paths found.
  - ADDA\_HUMAN was not found.
  - AT1A1\_HUMAN was not found.
- science-signaling-egfr
  [+]

  No paths found.
  - ADDA\_HUMAN was not found.
  - AT1A1\_HUMAN was not found.
- cancer-cell-map-egfr1
  [+]

  No paths found.
  - ADDA\_HUMAN was not found.
  - AT1A1\_HUMAN was not found.
- reactome-egfr
  [+]

  No paths found.
  - ADDA\_HUMAN was not found.
  - AT1A1\_HUMAN was not found.
- kegg-erbb
  [+]

  No paths found.
  - ADDA\_HUMAN was not found.
  - AT1A1\_HUMAN was not found.
- pid-erbb1
  [+]

  No paths found.
  - ADDA\_HUMAN was not found.
  - AT1A1\_HUMAN was not found.
- biocarta-egf
  [+]

  No paths found.
  - ADDA\_HUMAN was not found.
  - AT1A1\_HUMAN was not found.

### PARD3\_HUMAN → AMOT\_HUMAN / PARD3\_HUMAN ⊣ AMOT\_HUMAN

#### 1. Resources that have the edge


#### 2. Resources that contain endpoints (but not an edge)

- phosphositeplus-kinase-substrate
  [+]

  No paths found.

#### 3. Resources that match only one endpoint (fringe)


#### 4. Resources that don't match any endpoint

- biocarta-egf
  [+]

  No paths found.
  - AMOT\_HUMAN was not found.
  - PARD3\_HUMAN was not found.
- kegg-mapk
  [+]

  No paths found.
  - AMOT\_HUMAN was not found.
  - PARD3\_HUMAN was not found.
- science-signaling-egfr
  [+]

  No paths found.
  - AMOT\_HUMAN was not found.
  - PARD3\_HUMAN was not found.
- layek
  [+]

  No paths found.
  - AMOT\_HUMAN was not found.
  - PARD3\_HUMAN was not found.
- kegg-erbb
  [+]

  No paths found.
  - AMOT\_HUMAN was not found.
  - PARD3\_HUMAN was not found.
- reactome-egfr
  [+]

  No paths found.
  - AMOT\_HUMAN was not found.
  - PARD3\_HUMAN was not found.
- cancer-cell-map-egfr1
  [+]

  No paths found.
  - AMOT\_HUMAN was not found.
  - PARD3\_HUMAN was not found.
- pid-erbb1
  [+]

  No paths found.
  - AMOT\_HUMAN was not found.
  - PARD3\_HUMAN was not found.

### 1433G\_HUMAN → ZCH18\_HUMAN / 1433G\_HUMAN ⊣ ZCH18\_HUMAN

#### 1. Resources that have the edge


#### 2. Resources that contain endpoints (but not an edge)

- phosphositeplus-kinase-substrate
  [+]

  No paths found.

#### 3. Resources that match only one endpoint (fringe)


#### 4. Resources that don't match any endpoint

- layek
  [+]

  No paths found.
  - 1433G\_HUMAN was not found.
  - ZCH18\_HUMAN was not found.
- reactome-egfr
  [+]

  No paths found.
  - 1433G\_HUMAN was not found.
  - ZCH18\_HUMAN was not found.
- cancer-cell-map-egfr1
  [+]

  No paths found.
  - 1433G\_HUMAN was not found.
  - ZCH18\_HUMAN was not found.
- kegg-mapk
  [+]

  No paths found.
  - 1433G\_HUMAN was not found.
  - ZCH18\_HUMAN was not found.
- biocarta-egf
  [+]

  No paths found.
  - 1433G\_HUMAN was not found.
  - ZCH18\_HUMAN was not found.
- science-signaling-egfr
  [+]

  No paths found.
  - 1433G\_HUMAN was not found.
  - ZCH18\_HUMAN was not found.
- kegg-erbb
  [+]

  No paths found.
  - 1433G\_HUMAN was not found.
  - ZCH18\_HUMAN was not found.
- pid-erbb1
  [+]

  No paths found.
  - 1433G\_HUMAN was not found.
  - ZCH18\_HUMAN was not found.

### CDK5\_HUMAN ⊣ ATX2L\_HUMAN

#### 1. Resources that have the edge


#### 2. Resources that contain endpoints (but not an edge)

- phosphositeplus-kinase-substrate
  [+]

  No paths found.

#### 3. Resources that match only one endpoint (fringe)


#### 4. Resources that don't match any endpoint

- layek
  [+]

  No paths found.
  - ATX2L\_HUMAN was not found.
  - CDK5\_HUMAN was not found.
- biocarta-egf
  [+]

  No paths found.
  - ATX2L\_HUMAN was not found.
  - CDK5\_HUMAN was not found.
- reactome-egfr
  [+]

  No paths found.
  - ATX2L\_HUMAN was not found.
  - CDK5\_HUMAN was not found.
- kegg-erbb
  [+]

  No paths found.
  - ATX2L\_HUMAN was not found.
  - CDK5\_HUMAN was not found.
- science-signaling-egfr
  [+]

  No paths found.
  - ATX2L\_HUMAN was not found.
  - CDK5\_HUMAN was not found.
- cancer-cell-map-egfr1
  [+]

  No paths found.
  - ATX2L\_HUMAN was not found.
  - CDK5\_HUMAN was not found.
- kegg-mapk
  [+]

  No paths found.
  - ATX2L\_HUMAN was not found.
  - CDK5\_HUMAN was not found.
- pid-erbb1
  [+]

  No paths found.
  - ATX2L\_HUMAN was not found.
  - CDK5\_HUMAN was not found.

### GRB2\_HUMAN → PTN18\_HUMAN

#### 1. Resources that have the edge


#### 2. Resources that contain endpoints (but not an edge)


#### 3. Resources that match only one endpoint (fringe)

- biocarta-egf
  [+]

  No paths found.
  - PTN18\_HUMAN was not found.
- cancer-cell-map-egfr1
  [+]

  No paths found.
  - PTN18\_HUMAN was not found.
- kegg-erbb
  [+]

  No paths found.
  - PTN18\_HUMAN was not found.
- reactome-egfr
  [+]

  No paths found.
  - PTN18\_HUMAN was not found.
- phosphositeplus-kinase-substrate
  [+]

  No paths found.
  - PTN18\_HUMAN was not found.
- kegg-mapk
  [+]

  No paths found.
  - PTN18\_HUMAN was not found.
- science-signaling-egfr
  [+]

  No paths found.
  - PTN18\_HUMAN was not found.
- layek
  [+]

  No paths found.
  - PTN18\_HUMAN was not found.
- pid-erbb1
  [+]

  No paths found.
  - PTN18\_HUMAN was not found.

#### 4. Resources that don't match any endpoint


### GRB2\_HUMAN → DOCK4\_HUMAN

#### 1. Resources that have the edge


#### 2. Resources that contain endpoints (but not an edge)


#### 3. Resources that match only one endpoint (fringe)

- reactome-egfr
  [+]

  No paths found.
  - DOCK4\_HUMAN was not found.
- science-signaling-egfr
  [+]

  No paths found.
  - DOCK4\_HUMAN was not found.
- pid-erbb1
  [+]

  No paths found.
  - DOCK4\_HUMAN was not found.
- kegg-mapk
  [+]

  No paths found.
  - DOCK4\_HUMAN was not found.
- layek
  [+]

  No paths found.
  - DOCK4\_HUMAN was not found.
- cancer-cell-map-egfr1
  [+]

  No paths found.
  - DOCK4\_HUMAN was not found.
- phosphositeplus-kinase-substrate
  [+]

  No paths found.
  - DOCK4\_HUMAN was not found.
- biocarta-egf
  [+]

  No paths found.
  - DOCK4\_HUMAN was not found.
- kegg-erbb
  [+]

  No paths found.
  - DOCK4\_HUMAN was not found.

#### 4. Resources that don't match any endpoint


### GRB2\_HUMAN → GAREM\_HUMAN

#### 1. Resources that have the edge


#### 2. Resources that contain endpoints (but not an edge)


#### 3. Resources that match only one endpoint (fringe)

- biocarta-egf
  [+]

  No paths found.
  - GAREM\_HUMAN was not found.
- reactome-egfr
  [+]

  No paths found.
  - GAREM\_HUMAN was not found.
- kegg-erbb
  [+]

  No paths found.
  - GAREM\_HUMAN was not found.
- pid-erbb1
  [+]

  No paths found.
  - GAREM\_HUMAN was not found.
- science-signaling-egfr
  [+]

  No paths found.
  - GAREM\_HUMAN was not found.
- phosphositeplus-kinase-substrate
  [+]

  No paths found.
  - GAREM\_HUMAN was not found.
- cancer-cell-map-egfr1
  [+]

  No paths found.
  - GAREM\_HUMAN was not found.
- layek
  [+]

  No paths found.
  - GAREM\_HUMAN was not found.
- kegg-mapk
  [+]

  No paths found.
  - GAREM\_HUMAN was not found.

#### 4. Resources that don't match any endpoint


### GRB2\_HUMAN → PHAG1\_HUMAN / GRB2\_HUMAN ⊣ PHAG1\_HUMAN

#### 1. Resources that have the edge


#### 2. Resources that contain endpoints (but not an edge)


#### 3. Resources that match only one endpoint (fringe)

- reactome-egfr
  [+]

  No paths found.
  - PHAG1\_HUMAN was not found.
- biocarta-egf
  [+]

  No paths found.
  - PHAG1\_HUMAN was not found.
- layek
  [+]

  No paths found.
  - PHAG1\_HUMAN was not found.
- science-signaling-egfr
  [+]

  No paths found.
  - PHAG1\_HUMAN was not found.
- kegg-erbb
  [+]

  No paths found.
  - PHAG1\_HUMAN was not found.
- pid-erbb1
  [+]

  No paths found.
  - PHAG1\_HUMAN was not found.
- phosphositeplus-kinase-substrate
  [+]

  No paths found.
  - PHAG1\_HUMAN was not found.
- kegg-mapk
  [+]

  No paths found.
  - PHAG1\_HUMAN was not found.
- cancer-cell-map-egfr1
  [+]

  No paths found.
  - PHAG1\_HUMAN was not found.

#### 4. Resources that don't match any endpoint


### GRB2\_HUMAN → WIPF2\_HUMAN

#### 1. Resources that have the edge


#### 2. Resources that contain endpoints (but not an edge)


#### 3. Resources that match only one endpoint (fringe)

- biocarta-egf
  [+]

  No paths found.
  - WIPF2\_HUMAN was not found.
- reactome-egfr
  [+]

  No paths found.
  - WIPF2\_HUMAN was not found.
- phosphositeplus-kinase-substrate
  [+]

  No paths found.
  - WIPF2\_HUMAN was not found.
- cancer-cell-map-egfr1
  [+]

  No paths found.
  - WIPF2\_HUMAN was not found.
- kegg-mapk
  [+]

  No paths found.
  - WIPF2\_HUMAN was not found.
- pid-erbb1
  [+]

  No paths found.
  - WIPF2\_HUMAN was not found.
- science-signaling-egfr
  [+]

  No paths found.
  - WIPF2\_HUMAN was not found.
- kegg-erbb
  [+]

  No paths found.
  - WIPF2\_HUMAN was not found.
- layek
  [+]

  No paths found.
  - WIPF2\_HUMAN was not found.

#### 4. Resources that don't match any endpoint


### MK01\_HUMAN ⊣ KPRA\_HUMAN

#### 1. Resources that have the edge


#### 2. Resources that contain endpoints (but not an edge)


#### 3. Resources that match only one endpoint (fringe)

- reactome-egfr
  [+]

  No paths found.
  - KPRA\_HUMAN was not found.
- kegg-erbb
  [+]

  No paths found.
  - KPRA\_HUMAN was not found.
- kegg-mapk
  [+]

  No paths found.
  - KPRA\_HUMAN was not found.
- phosphositeplus-kinase-substrate
  [+]

  No paths found.
  - KPRA\_HUMAN was not found.
- cancer-cell-map-egfr1
  [+]

  No paths found.
  - KPRA\_HUMAN was not found.
- science-signaling-egfr
  [+]

  No paths found.
  - KPRA\_HUMAN was not found.
- pid-erbb1
  [+]

  No paths found.
  - KPRA\_HUMAN was not found.
- layek
  [+]

  No paths found.
  - KPRA\_HUMAN was not found.

#### 4. Resources that don't match any endpoint

- biocarta-egf
  [+]

  No paths found.
  - MK01\_HUMAN was not found.
  - KPRA\_HUMAN was not found.

### PLCG1\_HUMAN → ARHGB\_HUMAN

#### 1. Resources that have the edge


#### 2. Resources that contain endpoints (but not an edge)


#### 3. Resources that match only one endpoint (fringe)

- pid-erbb1
  [+]

  No paths found.
  - ARHGB\_HUMAN was not found.
- phosphositeplus-kinase-substrate
  [+]

  No paths found.
  - ARHGB\_HUMAN was not found.
- biocarta-egf
  [+]

  No paths found.
  - ARHGB\_HUMAN was not found.
- kegg-erbb
  [+]

  No paths found.
  - ARHGB\_HUMAN was not found.
- cancer-cell-map-egfr1
  [+]

  No paths found.
  - ARHGB\_HUMAN was not found.
- reactome-egfr
  [+]

  No paths found.
  - ARHGB\_HUMAN was not found.

#### 4. Resources that don't match any endpoint

- kegg-mapk
  [+]

  No paths found.
  - ARHGB\_HUMAN was not found.
  - PLCG1\_HUMAN was not found.
- layek
  [+]

  No paths found.
  - ARHGB\_HUMAN was not found.
  - PLCG1\_HUMAN was not found.
- science-signaling-egfr
  [+]

  No paths found.
  - ARHGB\_HUMAN was not found.
  - PLCG1\_HUMAN was not found.

### CBL\_HUMAN → ACTB\_HUMAN / CBL\_HUMAN ⊣ ACTB\_HUMAN

#### 1. Resources that have the edge


#### 2. Resources that contain endpoints (but not an edge)


#### 3. Resources that match only one endpoint (fringe)

- phosphositeplus-kinase-substrate
  [+]

  No paths found.
  - ACTB\_HUMAN was not found.
- cancer-cell-map-egfr1
  [+]

  No paths found.
  - ACTB\_HUMAN was not found.
- reactome-egfr
  [+]

  No paths found.
  - ACTB\_HUMAN was not found.
- science-signaling-egfr
  [+]

  No paths found.
  - ACTB\_HUMAN was not found.

#### 4. Resources that don't match any endpoint

- biocarta-egf
  [+]

  No paths found.
  - ACTB\_HUMAN was not found.
  - CBL\_HUMAN was not found.
- pid-erbb1
  [+]

  No paths found.
  - ACTB\_HUMAN was not found.
  - CBL\_HUMAN was not found.
- layek
  [+]

  No paths found.
  - ACTB\_HUMAN was not found.
  - CBL\_HUMAN was not found.
- kegg-mapk
  [+]

  No paths found.
  - ACTB\_HUMAN was not found.
  - CBL\_HUMAN was not found.
- kegg-erbb
  [+]

  No paths found.
  - ACTB\_HUMAN was not found.
  - CBL\_HUMAN was not found.

### AFAD\_HUMAN → RRAS2\_HUMAN / AFAD\_HUMAN ⊣ RRAS2\_HUMAN

#### 1. Resources that have the edge


#### 2. Resources that contain endpoints (but not an edge)


#### 3. Resources that match only one endpoint (fringe)

- phosphositeplus-kinase-substrate
  [+]

  No paths found.
  - RRAS2\_HUMAN was not found.
- kegg-mapk
  [+]

  No paths found.
  - AFAD\_HUMAN was not found.

#### 4. Resources that don't match any endpoint

- layek
  [+]

  No paths found.
  - AFAD\_HUMAN was not found.
  - RRAS2\_HUMAN was not found.
- biocarta-egf
  [+]

  No paths found.
  - AFAD\_HUMAN was not found.
  - RRAS2\_HUMAN was not found.
- science-signaling-egfr
  [+]

  No paths found.
  - AFAD\_HUMAN was not found.
  - RRAS2\_HUMAN was not found.
- reactome-egfr
  [+]

  No paths found.
  - AFAD\_HUMAN was not found.
  - RRAS2\_HUMAN was not found.
- pid-erbb1
  [+]

  No paths found.
  - AFAD\_HUMAN was not found.
  - RRAS2\_HUMAN was not found.
- kegg-erbb
  [+]

  No paths found.
  - AFAD\_HUMAN was not found.
  - RRAS2\_HUMAN was not found.
- cancer-cell-map-egfr1
  [+]

  No paths found.
  - AFAD\_HUMAN was not found.
  - RRAS2\_HUMAN was not found.

### DREB\_HUMAN → MARCS\_HUMAN

#### 1. Resources that have the edge


#### 2. Resources that contain endpoints (but not an edge)


#### 3. Resources that match only one endpoint (fringe)

- phosphositeplus-kinase-substrate
  [+]

  No paths found.
  - DREB\_HUMAN was not found.

#### 4. Resources that don't match any endpoint

- kegg-mapk
  [+]

  No paths found.
  - MARCS\_HUMAN was not found.
  - DREB\_HUMAN was not found.
- cancer-cell-map-egfr1
  [+]

  No paths found.
  - MARCS\_HUMAN was not found.
  - DREB\_HUMAN was not found.
- pid-erbb1
  [+]

  No paths found.
  - MARCS\_HUMAN was not found.
  - DREB\_HUMAN was not found.
- kegg-erbb
  [+]

  No paths found.
  - MARCS\_HUMAN was not found.
  - DREB\_HUMAN was not found.
- biocarta-egf
  [+]

  No paths found.
  - MARCS\_HUMAN was not found.
  - DREB\_HUMAN was not found.
- science-signaling-egfr
  [+]

  No paths found.
  - MARCS\_HUMAN was not found.
  - DREB\_HUMAN was not found.
- layek
  [+]

  No paths found.
  - MARCS\_HUMAN was not found.
  - DREB\_HUMAN was not found.
- reactome-egfr
  [+]

  No paths found.
  - MARCS\_HUMAN was not found.
  - DREB\_HUMAN was not found.

### AFAD\_HUMAN → ZO1\_HUMAN / AFAD\_HUMAN ⊣ ZO1\_HUMAN

#### 1. Resources that have the edge


#### 2. Resources that contain endpoints (but not an edge)


#### 3. Resources that match only one endpoint (fringe)

- phosphositeplus-kinase-substrate
  [+]

  No paths found.
  - ZO1\_HUMAN was not found.

#### 4. Resources that don't match any endpoint

- biocarta-egf
  [+]

  No paths found.
  - ZO1\_HUMAN was not found.
  - AFAD\_HUMAN was not found.
- pid-erbb1
  [+]

  No paths found.
  - ZO1\_HUMAN was not found.
  - AFAD\_HUMAN was not found.
- science-signaling-egfr
  [+]

  No paths found.
  - ZO1\_HUMAN was not found.
  - AFAD\_HUMAN was not found.
- reactome-egfr
  [+]

  No paths found.
  - ZO1\_HUMAN was not found.
  - AFAD\_HUMAN was not found.
- kegg-mapk
  [+]

  No paths found.
  - ZO1\_HUMAN was not found.
  - AFAD\_HUMAN was not found.
- kegg-erbb
  [+]

  No paths found.
  - ZO1\_HUMAN was not found.
  - AFAD\_HUMAN was not found.
- layek
  [+]

  No paths found.
  - ZO1\_HUMAN was not found.
  - AFAD\_HUMAN was not found.
- cancer-cell-map-egfr1
  [+]

  No paths found.
  - ZO1\_HUMAN was not found.
  - AFAD\_HUMAN was not found.

### 1433G\_HUMAN → ANS1A\_HUMAN / 1433G\_HUMAN ⊣ ANS1A\_HUMAN

#### 1. Resources that have the edge


#### 2. Resources that contain endpoints (but not an edge)


#### 3. Resources that match only one endpoint (fringe)

- phosphositeplus-kinase-substrate
  [+]

  No paths found.
  - ANS1A\_HUMAN was not found.

#### 4. Resources that don't match any endpoint

- kegg-erbb
  [+]

  No paths found.
  - 1433G\_HUMAN was not found.
  - ANS1A\_HUMAN was not found.
- pid-erbb1
  [+]

  No paths found.
  - 1433G\_HUMAN was not found.
  - ANS1A\_HUMAN was not found.
- layek
  [+]

  No paths found.
  - 1433G\_HUMAN was not found.
  - ANS1A\_HUMAN was not found.
- kegg-mapk
  [+]

  No paths found.
  - 1433G\_HUMAN was not found.
  - ANS1A\_HUMAN was not found.
- biocarta-egf
  [+]

  No paths found.
  - 1433G\_HUMAN was not found.
  - ANS1A\_HUMAN was not found.
- reactome-egfr
  [+]

  No paths found.
  - 1433G\_HUMAN was not found.
  - ANS1A\_HUMAN was not found.
- cancer-cell-map-egfr1
  [+]

  No paths found.
  - 1433G\_HUMAN was not found.
  - ANS1A\_HUMAN was not found.
- science-signaling-egfr
  [+]

  No paths found.
  - 1433G\_HUMAN was not found.
  - ANS1A\_HUMAN was not found.

### SAT1\_HUMAN → CH10\_HUMAN / SAT1\_HUMAN ⊣ CH10\_HUMAN

#### 1. Resources that have the edge


#### 2. Resources that contain endpoints (but not an edge)


#### 3. Resources that match only one endpoint (fringe)

- phosphositeplus-kinase-substrate
  [+]

  No paths found.
  - CH10\_HUMAN was not found.

#### 4. Resources that don't match any endpoint

- cancer-cell-map-egfr1
  [+]

  No paths found.
  - CH10\_HUMAN was not found.
  - SAT1\_HUMAN was not found.
- biocarta-egf
  [+]

  No paths found.
  - CH10\_HUMAN was not found.
  - SAT1\_HUMAN was not found.
- kegg-erbb
  [+]

  No paths found.
  - CH10\_HUMAN was not found.
  - SAT1\_HUMAN was not found.
- layek
  [+]

  No paths found.
  - CH10\_HUMAN was not found.
  - SAT1\_HUMAN was not found.
- pid-erbb1
  [+]

  No paths found.
  - CH10\_HUMAN was not found.
  - SAT1\_HUMAN was not found.
- science-signaling-egfr
  [+]

  No paths found.
  - CH10\_HUMAN was not found.
  - SAT1\_HUMAN was not found.
- reactome-egfr
  [+]

  No paths found.
  - CH10\_HUMAN was not found.
  - SAT1\_HUMAN was not found.
- kegg-mapk
  [+]

  No paths found.
  - CH10\_HUMAN was not found.
  - SAT1\_HUMAN was not found.

### MECP2\_HUMAN → CDKL5\_HUMAN / MECP2\_HUMAN ⊣ CDKL5\_HUMAN

#### 1. Resources that have the edge


#### 2. Resources that contain endpoints (but not an edge)


#### 3. Resources that match only one endpoint (fringe)

- phosphositeplus-kinase-substrate
  [+]

  No paths found.
  - MECP2\_HUMAN was not found.

#### 4. Resources that don't match any endpoint

- layek
  [+]

  No paths found.
  - CDKL5\_HUMAN was not found.
  - MECP2\_HUMAN was not found.
- kegg-erbb
  [+]

  No paths found.
  - CDKL5\_HUMAN was not found.
  - MECP2\_HUMAN was not found.
- reactome-egfr
  [+]

  No paths found.
  - CDKL5\_HUMAN was not found.
  - MECP2\_HUMAN was not found.
- biocarta-egf
  [+]

  No paths found.
  - CDKL5\_HUMAN was not found.
  - MECP2\_HUMAN was not found.
- pid-erbb1
  [+]

  No paths found.
  - CDKL5\_HUMAN was not found.
  - MECP2\_HUMAN was not found.
- science-signaling-egfr
  [+]

  No paths found.
  - CDKL5\_HUMAN was not found.
  - MECP2\_HUMAN was not found.
- cancer-cell-map-egfr1
  [+]

  No paths found.
  - CDKL5\_HUMAN was not found.
  - MECP2\_HUMAN was not found.
- kegg-mapk
  [+]

  No paths found.
  - CDKL5\_HUMAN was not found.
  - MECP2\_HUMAN was not found.

### AFAD\_HUMAN → DREB\_HUMAN / AFAD\_HUMAN ⊣ DREB\_HUMAN

#### 1. Resources that have the edge


#### 2. Resources that contain endpoints (but not an edge)


#### 3. Resources that match only one endpoint (fringe)

- phosphositeplus-kinase-substrate
  [+]

  No paths found.
  - DREB\_HUMAN was not found.

#### 4. Resources that don't match any endpoint

- cancer-cell-map-egfr1
  [+]

  No paths found.
  - AFAD\_HUMAN was not found.
  - DREB\_HUMAN was not found.
- kegg-mapk
  [+]

  No paths found.
  - AFAD\_HUMAN was not found.
  - DREB\_HUMAN was not found.
- biocarta-egf
  [+]

  No paths found.
  - AFAD\_HUMAN was not found.
  - DREB\_HUMAN was not found.
- pid-erbb1
  [+]

  No paths found.
  - AFAD\_HUMAN was not found.
  - DREB\_HUMAN was not found.
- reactome-egfr
  [+]

  No paths found.
  - AFAD\_HUMAN was not found.
  - DREB\_HUMAN was not found.
- layek
  [+]

  No paths found.
  - AFAD\_HUMAN was not found.
  - DREB\_HUMAN was not found.
- kegg-erbb
  [+]

  No paths found.
  - AFAD\_HUMAN was not found.
  - DREB\_HUMAN was not found.
- science-signaling-egfr
  [+]

  No paths found.
  - AFAD\_HUMAN was not found.
  - DREB\_HUMAN was not found.

### NEK6\_HUMAN → EMAL4\_HUMAN / NEK6\_HUMAN ⊣ EMAL4\_HUMAN

#### 1. Resources that have the edge


#### 2. Resources that contain endpoints (but not an edge)


#### 3. Resources that match only one endpoint (fringe)

- phosphositeplus-kinase-substrate
  [+]

  No paths found.
  - EMAL4\_HUMAN was not found.

#### 4. Resources that don't match any endpoint

- biocarta-egf
  [+]

  No paths found.
  - EMAL4\_HUMAN was not found.
  - NEK6\_HUMAN was not found.
- pid-erbb1
  [+]

  No paths found.
  - EMAL4\_HUMAN was not found.
  - NEK6\_HUMAN was not found.
- layek
  [+]

  No paths found.
  - EMAL4\_HUMAN was not found.
  - NEK6\_HUMAN was not found.
- kegg-mapk
  [+]

  No paths found.
  - EMAL4\_HUMAN was not found.
  - NEK6\_HUMAN was not found.
- science-signaling-egfr
  [+]

  No paths found.
  - EMAL4\_HUMAN was not found.
  - NEK6\_HUMAN was not found.
- cancer-cell-map-egfr1
  [+]

  No paths found.
  - EMAL4\_HUMAN was not found.
  - NEK6\_HUMAN was not found.
- kegg-erbb
  [+]

  No paths found.
  - EMAL4\_HUMAN was not found.
  - NEK6\_HUMAN was not found.
- reactome-egfr
  [+]

  No paths found.
  - EMAL4\_HUMAN was not found.
  - NEK6\_HUMAN was not found.

### PARD3\_HUMAN ⊣ PSME3\_HUMAN

#### 1. Resources that have the edge


#### 2. Resources that contain endpoints (but not an edge)


#### 3. Resources that match only one endpoint (fringe)

- phosphositeplus-kinase-substrate
  [+]

  No paths found.
  - PSME3\_HUMAN was not found.

#### 4. Resources that don't match any endpoint

- cancer-cell-map-egfr1
  [+]

  No paths found.
  - PSME3\_HUMAN was not found.
  - PARD3\_HUMAN was not found.
- science-signaling-egfr
  [+]

  No paths found.
  - PSME3\_HUMAN was not found.
  - PARD3\_HUMAN was not found.
- reactome-egfr
  [+]

  No paths found.
  - PSME3\_HUMAN was not found.
  - PARD3\_HUMAN was not found.
- pid-erbb1
  [+]

  No paths found.
  - PSME3\_HUMAN was not found.
  - PARD3\_HUMAN was not found.
- kegg-erbb
  [+]

  No paths found.
  - PSME3\_HUMAN was not found.
  - PARD3\_HUMAN was not found.
- layek
  [+]

  No paths found.
  - PSME3\_HUMAN was not found.
  - PARD3\_HUMAN was not found.
- biocarta-egf
  [+]

  No paths found.
  - PSME3\_HUMAN was not found.
  - PARD3\_HUMAN was not found.
- kegg-mapk
  [+]

  No paths found.
  - PSME3\_HUMAN was not found.
  - PARD3\_HUMAN was not found.

### SAT1\_HUMAN → T22D4\_HUMAN / SAT1\_HUMAN ⊣ T22D4\_HUMAN

#### 1. Resources that have the edge


#### 2. Resources that contain endpoints (but not an edge)


#### 3. Resources that match only one endpoint (fringe)

- phosphositeplus-kinase-substrate
  [+]

  No paths found.
  - T22D4\_HUMAN was not found.

#### 4. Resources that don't match any endpoint

- science-signaling-egfr
  [+]

  No paths found.
  - SAT1\_HUMAN was not found.
  - T22D4\_HUMAN was not found.
- kegg-erbb
  [+]

  No paths found.
  - SAT1\_HUMAN was not found.
  - T22D4\_HUMAN was not found.
- pid-erbb1
  [+]

  No paths found.
  - SAT1\_HUMAN was not found.
  - T22D4\_HUMAN was not found.
- kegg-mapk
  [+]

  No paths found.
  - SAT1\_HUMAN was not found.
  - T22D4\_HUMAN was not found.
- biocarta-egf
  [+]

  No paths found.
  - SAT1\_HUMAN was not found.
  - T22D4\_HUMAN was not found.
- layek
  [+]

  No paths found.
  - SAT1\_HUMAN was not found.
  - T22D4\_HUMAN was not found.
- cancer-cell-map-egfr1
  [+]

  No paths found.
  - SAT1\_HUMAN was not found.
  - T22D4\_HUMAN was not found.
- reactome-egfr
  [+]

  No paths found.
  - SAT1\_HUMAN was not found.
  - T22D4\_HUMAN was not found.

### CSK21\_HUMAN → PSIP1\_HUMAN / CSK21\_HUMAN ⊣ PSIP1\_HUMAN

#### 1. Resources that have the edge


#### 2. Resources that contain endpoints (but not an edge)


#### 3. Resources that match only one endpoint (fringe)

- phosphositeplus-kinase-substrate
  [+]

  No paths found.
  - PSIP1\_HUMAN was not found.

#### 4. Resources that don't match any endpoint

- cancer-cell-map-egfr1
  [+]

  No paths found.
  - CSK21\_HUMAN was not found.
  - PSIP1\_HUMAN was not found.
- pid-erbb1
  [+]

  No paths found.
  - CSK21\_HUMAN was not found.
  - PSIP1\_HUMAN was not found.
- kegg-erbb
  [+]

  No paths found.
  - CSK21\_HUMAN was not found.
  - PSIP1\_HUMAN was not found.
- reactome-egfr
  [+]

  No paths found.
  - CSK21\_HUMAN was not found.
  - PSIP1\_HUMAN was not found.
- biocarta-egf
  [+]

  No paths found.
  - CSK21\_HUMAN was not found.
  - PSIP1\_HUMAN was not found.
- science-signaling-egfr
  [+]

  No paths found.
  - CSK21\_HUMAN was not found.
  - PSIP1\_HUMAN was not found.
- kegg-mapk
  [+]

  No paths found.
  - CSK21\_HUMAN was not found.
  - PSIP1\_HUMAN was not found.
- layek
  [+]

  No paths found.
  - CSK21\_HUMAN was not found.
  - PSIP1\_HUMAN was not found.

### SMC3\_HUMAN → REC8\_HUMAN / SMC3\_HUMAN ⊣ REC8\_HUMAN

#### 1. Resources that have the edge


#### 2. Resources that contain endpoints (but not an edge)


#### 3. Resources that match only one endpoint (fringe)

- phosphositeplus-kinase-substrate
  [+]

  No paths found.
  - REC8\_HUMAN was not found.

#### 4. Resources that don't match any endpoint

- cancer-cell-map-egfr1
  [+]

  No paths found.
  - REC8\_HUMAN was not found.
  - SMC3\_HUMAN was not found.
- reactome-egfr
  [+]

  No paths found.
  - REC8\_HUMAN was not found.
  - SMC3\_HUMAN was not found.
- science-signaling-egfr
  [+]

  No paths found.
  - REC8\_HUMAN was not found.
  - SMC3\_HUMAN was not found.
- kegg-erbb
  [+]

  No paths found.
  - REC8\_HUMAN was not found.
  - SMC3\_HUMAN was not found.
- kegg-mapk
  [+]

  No paths found.
  - REC8\_HUMAN was not found.
  - SMC3\_HUMAN was not found.
- layek
  [+]

  No paths found.
  - REC8\_HUMAN was not found.
  - SMC3\_HUMAN was not found.
- pid-erbb1
  [+]

  No paths found.
  - REC8\_HUMAN was not found.
  - SMC3\_HUMAN was not found.
- biocarta-egf
  [+]

  No paths found.
  - REC8\_HUMAN was not found.
  - SMC3\_HUMAN was not found.

### NUCL\_HUMAN ⊣ PP1B\_HUMAN

#### 1. Resources that have the edge


#### 2. Resources that contain endpoints (but not an edge)


#### 3. Resources that match only one endpoint (fringe)

- phosphositeplus-kinase-substrate
  [+]

  No paths found.
  - PP1B\_HUMAN was not found.

#### 4. Resources that don't match any endpoint

- reactome-egfr
  [+]

  No paths found.
  - PP1B\_HUMAN was not found.
  - NUCL\_HUMAN was not found.
- kegg-mapk
  [+]

  No paths found.
  - PP1B\_HUMAN was not found.
  - NUCL\_HUMAN was not found.
- biocarta-egf
  [+]

  No paths found.
  - PP1B\_HUMAN was not found.
  - NUCL\_HUMAN was not found.
- cancer-cell-map-egfr1
  [+]

  No paths found.
  - PP1B\_HUMAN was not found.
  - NUCL\_HUMAN was not found.
- pid-erbb1
  [+]

  No paths found.
  - PP1B\_HUMAN was not found.
  - NUCL\_HUMAN was not found.
- science-signaling-egfr
  [+]

  No paths found.
  - PP1B\_HUMAN was not found.
  - NUCL\_HUMAN was not found.
- kegg-erbb
  [+]

  No paths found.
  - PP1B\_HUMAN was not found.
  - NUCL\_HUMAN was not found.
- layek
  [+]

  No paths found.
  - PP1B\_HUMAN was not found.
  - NUCL\_HUMAN was not found.

### 1433G\_HUMAN → SMAG2\_HUMAN / 1433G\_HUMAN ⊣ SMAG2\_HUMAN

#### 1. Resources that have the edge


#### 2. Resources that contain endpoints (but not an edge)


#### 3. Resources that match only one endpoint (fringe)

- phosphositeplus-kinase-substrate
  [+]

  No paths found.
  - SMAG2\_HUMAN was not found.

#### 4. Resources that don't match any endpoint

- cancer-cell-map-egfr1
  [+]

  No paths found.
  - SMAG2\_HUMAN was not found.
  - 1433G\_HUMAN was not found.
- kegg-erbb
  [+]

  No paths found.
  - SMAG2\_HUMAN was not found.
  - 1433G\_HUMAN was not found.
- reactome-egfr
  [+]

  No paths found.
  - SMAG2\_HUMAN was not found.
  - 1433G\_HUMAN was not found.
- biocarta-egf
  [+]

  No paths found.
  - SMAG2\_HUMAN was not found.
  - 1433G\_HUMAN was not found.
- layek
  [+]

  No paths found.
  - SMAG2\_HUMAN was not found.
  - 1433G\_HUMAN was not found.
- science-signaling-egfr
  [+]

  No paths found.
  - SMAG2\_HUMAN was not found.
  - 1433G\_HUMAN was not found.
- kegg-mapk
  [+]

  No paths found.
  - SMAG2\_HUMAN was not found.
  - 1433G\_HUMAN was not found.
- pid-erbb1
  [+]

  No paths found.
  - SMAG2\_HUMAN was not found.
  - 1433G\_HUMAN was not found.

### CP3A4\_HUMAN → PGRC1\_HUMAN / CP3A4\_HUMAN ⊣ PGRC1\_HUMAN

#### 1. Resources that have the edge


#### 2. Resources that contain endpoints (but not an edge)


#### 3. Resources that match only one endpoint (fringe)

- phosphositeplus-kinase-substrate
  [+]

  No paths found.
  - PGRC1\_HUMAN was not found.

#### 4. Resources that don't match any endpoint

- reactome-egfr
  [+]

  No paths found.
  - CP3A4\_HUMAN was not found.
  - PGRC1\_HUMAN was not found.
- kegg-mapk
  [+]

  No paths found.
  - CP3A4\_HUMAN was not found.
  - PGRC1\_HUMAN was not found.
- science-signaling-egfr
  [+]

  No paths found.
  - CP3A4\_HUMAN was not found.
  - PGRC1\_HUMAN was not found.
- cancer-cell-map-egfr1
  [+]

  No paths found.
  - CP3A4\_HUMAN was not found.
  - PGRC1\_HUMAN was not found.
- biocarta-egf
  [+]

  No paths found.
  - CP3A4\_HUMAN was not found.
  - PGRC1\_HUMAN was not found.
- kegg-erbb
  [+]

  No paths found.
  - CP3A4\_HUMAN was not found.
  - PGRC1\_HUMAN was not found.
- layek
  [+]

  No paths found.
  - CP3A4\_HUMAN was not found.
  - PGRC1\_HUMAN was not found.
- pid-erbb1
  [+]

  No paths found.
  - CP3A4\_HUMAN was not found.
  - PGRC1\_HUMAN was not found.

### CSK21\_HUMAN → HIRP3\_HUMAN / CSK21\_HUMAN ⊣ HIRP3\_HUMAN

#### 1. Resources that have the edge


#### 2. Resources that contain endpoints (but not an edge)


#### 3. Resources that match only one endpoint (fringe)

- phosphositeplus-kinase-substrate
  [+]

  No paths found.
  - HIRP3\_HUMAN was not found.

#### 4. Resources that don't match any endpoint

- kegg-erbb
  [+]

  No paths found.
  - HIRP3\_HUMAN was not found.
  - CSK21\_HUMAN was not found.
- cancer-cell-map-egfr1
  [+]

  No paths found.
  - HIRP3\_HUMAN was not found.
  - CSK21\_HUMAN was not found.
- pid-erbb1
  [+]

  No paths found.
  - HIRP3\_HUMAN was not found.
  - CSK21\_HUMAN was not found.
- biocarta-egf
  [+]

  No paths found.
  - HIRP3\_HUMAN was not found.
  - CSK21\_HUMAN was not found.
- science-signaling-egfr
  [+]

  No paths found.
  - HIRP3\_HUMAN was not found.
  - CSK21\_HUMAN was not found.
- kegg-mapk
  [+]

  No paths found.
  - HIRP3\_HUMAN was not found.
  - CSK21\_HUMAN was not found.
- layek
  [+]

  No paths found.
  - HIRP3\_HUMAN was not found.
  - CSK21\_HUMAN was not found.
- reactome-egfr
  [+]

  No paths found.
  - HIRP3\_HUMAN was not found.
  - CSK21\_HUMAN was not found.

### SIN3A\_HUMAN → MKX\_HUMAN / SIN3A\_HUMAN ⊣ MKX\_HUMAN

#### 1. Resources that have the edge


#### 2. Resources that contain endpoints (but not an edge)


#### 3. Resources that match only one endpoint (fringe)

- cancer-cell-map-egfr1
  [+]

  No paths found.
  - MKX\_HUMAN was not found.

#### 4. Resources that don't match any endpoint

- pid-erbb1
  [+]

  No paths found.
  - SIN3A\_HUMAN was not found.
  - MKX\_HUMAN was not found.
- reactome-egfr
  [+]

  No paths found.
  - SIN3A\_HUMAN was not found.
  - MKX\_HUMAN was not found.
- phosphositeplus-kinase-substrate
  [+]

  No paths found.
  - SIN3A\_HUMAN was not found.
  - MKX\_HUMAN was not found.
- science-signaling-egfr
  [+]

  No paths found.
  - SIN3A\_HUMAN was not found.
  - MKX\_HUMAN was not found.
- biocarta-egf
  [+]

  No paths found.
  - SIN3A\_HUMAN was not found.
  - MKX\_HUMAN was not found.
- kegg-mapk
  [+]

  No paths found.
  - SIN3A\_HUMAN was not found.
  - MKX\_HUMAN was not found.
- layek
  [+]

  No paths found.
  - SIN3A\_HUMAN was not found.
  - MKX\_HUMAN was not found.
- kegg-erbb
  [+]

  No paths found.
  - SIN3A\_HUMAN was not found.
  - MKX\_HUMAN was not found.

### KI67\_HUMAN → KIF15\_HUMAN / KI67\_HUMAN ⊣ KIF15\_HUMAN

#### 1. Resources that have the edge


#### 2. Resources that contain endpoints (but not an edge)


#### 3. Resources that match only one endpoint (fringe)

- phosphositeplus-kinase-substrate
  [+]

  No paths found.
  - KIF15\_HUMAN was not found.

#### 4. Resources that don't match any endpoint

- biocarta-egf
  [+]

  No paths found.
  - KI67\_HUMAN was not found.
  - KIF15\_HUMAN was not found.
- kegg-mapk
  [+]

  No paths found.
  - KI67\_HUMAN was not found.
  - KIF15\_HUMAN was not found.
- kegg-erbb
  [+]

  No paths found.
  - KI67\_HUMAN was not found.
  - KIF15\_HUMAN was not found.
- cancer-cell-map-egfr1
  [+]

  No paths found.
  - KI67\_HUMAN was not found.
  - KIF15\_HUMAN was not found.
- science-signaling-egfr
  [+]

  No paths found.
  - KI67\_HUMAN was not found.
  - KIF15\_HUMAN was not found.
- layek
  [+]

  No paths found.
  - KI67\_HUMAN was not found.
  - KIF15\_HUMAN was not found.
- pid-erbb1
  [+]

  No paths found.
  - KI67\_HUMAN was not found.
  - KIF15\_HUMAN was not found.
- reactome-egfr
  [+]

  No paths found.
  - KI67\_HUMAN was not found.
  - KIF15\_HUMAN was not found.

### 1433G\_HUMAN → LSR\_HUMAN / 1433G\_HUMAN ⊣ LSR\_HUMAN

#### 1. Resources that have the edge


#### 2. Resources that contain endpoints (but not an edge)


#### 3. Resources that match only one endpoint (fringe)

- phosphositeplus-kinase-substrate
  [+]

  No paths found.
  - LSR\_HUMAN was not found.

#### 4. Resources that don't match any endpoint

- science-signaling-egfr
  [+]

  No paths found.
  - 1433G\_HUMAN was not found.
  - LSR\_HUMAN was not found.
- biocarta-egf
  [+]

  No paths found.
  - 1433G\_HUMAN was not found.
  - LSR\_HUMAN was not found.
- pid-erbb1
  [+]

  No paths found.
  - 1433G\_HUMAN was not found.
  - LSR\_HUMAN was not found.
- cancer-cell-map-egfr1
  [+]

  No paths found.
  - 1433G\_HUMAN was not found.
  - LSR\_HUMAN was not found.
- layek
  [+]

  No paths found.
  - 1433G\_HUMAN was not found.
  - LSR\_HUMAN was not found.
- reactome-egfr
  [+]

  No paths found.
  - 1433G\_HUMAN was not found.
  - LSR\_HUMAN was not found.
- kegg-erbb
  [+]

  No paths found.
  - 1433G\_HUMAN was not found.
  - LSR\_HUMAN was not found.
- kegg-mapk
  [+]

  No paths found.
  - 1433G\_HUMAN was not found.
  - LSR\_HUMAN was not found.

### KAP2\_HUMAN → AKAP1\_HUMAN / KAP2\_HUMAN ⊣ AKAP1\_HUMAN

#### 1. Resources that have the edge


#### 2. Resources that contain endpoints (but not an edge)


#### 3. Resources that match only one endpoint (fringe)

- reactome-egfr
  [+]

  No paths found.
  - AKAP1\_HUMAN was not found.

#### 4. Resources that don't match any endpoint

- phosphositeplus-kinase-substrate
  [+]

  No paths found.
  - KAP2\_HUMAN was not found.
  - AKAP1\_HUMAN was not found.
- pid-erbb1
  [+]

  No paths found.
  - KAP2\_HUMAN was not found.
  - AKAP1\_HUMAN was not found.
- kegg-erbb
  [+]

  No paths found.
  - KAP2\_HUMAN was not found.
  - AKAP1\_HUMAN was not found.
- biocarta-egf
  [+]

  No paths found.
  - KAP2\_HUMAN was not found.
  - AKAP1\_HUMAN was not found.
- cancer-cell-map-egfr1
  [+]

  No paths found.
  - KAP2\_HUMAN was not found.
  - AKAP1\_HUMAN was not found.
- layek
  [+]

  No paths found.
  - KAP2\_HUMAN was not found.
  - AKAP1\_HUMAN was not found.
- science-signaling-egfr
  [+]

  No paths found.
  - KAP2\_HUMAN was not found.
  - AKAP1\_HUMAN was not found.
- kegg-mapk
  [+]

  No paths found.
  - KAP2\_HUMAN was not found.
  - AKAP1\_HUMAN was not found.

### CSK21\_HUMAN → RBM33\_HUMAN / CSK21\_HUMAN ⊣ RBM33\_HUMAN

#### 1. Resources that have the edge


#### 2. Resources that contain endpoints (but not an edge)


#### 3. Resources that match only one endpoint (fringe)

- phosphositeplus-kinase-substrate
  [+]

  No paths found.
  - RBM33\_HUMAN was not found.

#### 4. Resources that don't match any endpoint

- cancer-cell-map-egfr1
  [+]

  No paths found.
  - CSK21\_HUMAN was not found.
  - RBM33\_HUMAN was not found.
- layek
  [+]

  No paths found.
  - CSK21\_HUMAN was not found.
  - RBM33\_HUMAN was not found.
- kegg-erbb
  [+]

  No paths found.
  - CSK21\_HUMAN was not found.
  - RBM33\_HUMAN was not found.
- pid-erbb1
  [+]

  No paths found.
  - CSK21\_HUMAN was not found.
  - RBM33\_HUMAN was not found.
- science-signaling-egfr
  [+]

  No paths found.
  - CSK21\_HUMAN was not found.
  - RBM33\_HUMAN was not found.
- kegg-mapk
  [+]

  No paths found.
  - CSK21\_HUMAN was not found.
  - RBM33\_HUMAN was not found.
- biocarta-egf
  [+]

  No paths found.
  - CSK21\_HUMAN was not found.
  - RBM33\_HUMAN was not found.
- reactome-egfr
  [+]

  No paths found.
  - CSK21\_HUMAN was not found.
  - RBM33\_HUMAN was not found.

### CSK21\_HUMAN → RL1D1\_HUMAN / CSK21\_HUMAN ⊣ RL1D1\_HUMAN

#### 1. Resources that have the edge


#### 2. Resources that contain endpoints (but not an edge)


#### 3. Resources that match only one endpoint (fringe)

- phosphositeplus-kinase-substrate
  [+]

  No paths found.
  - RL1D1\_HUMAN was not found.

#### 4. Resources that don't match any endpoint

- cancer-cell-map-egfr1
  [+]

  No paths found.
  - CSK21\_HUMAN was not found.
  - RL1D1\_HUMAN was not found.
- layek
  [+]

  No paths found.
  - CSK21\_HUMAN was not found.
  - RL1D1\_HUMAN was not found.
- pid-erbb1
  [+]

  No paths found.
  - CSK21\_HUMAN was not found.
  - RL1D1\_HUMAN was not found.
- kegg-mapk
  [+]

  No paths found.
  - CSK21\_HUMAN was not found.
  - RL1D1\_HUMAN was not found.
- kegg-erbb
  [+]

  No paths found.
  - CSK21\_HUMAN was not found.
  - RL1D1\_HUMAN was not found.
- biocarta-egf
  [+]

  No paths found.
  - CSK21\_HUMAN was not found.
  - RL1D1\_HUMAN was not found.
- reactome-egfr
  [+]

  No paths found.
  - CSK21\_HUMAN was not found.
  - RL1D1\_HUMAN was not found.
- science-signaling-egfr
  [+]

  No paths found.
  - CSK21\_HUMAN was not found.
  - RL1D1\_HUMAN was not found.

### KAP2\_HUMAN → AKA11\_HUMAN / KAP2\_HUMAN ⊣ AKA11\_HUMAN

#### 1. Resources that have the edge


#### 2. Resources that contain endpoints (but not an edge)


#### 3. Resources that match only one endpoint (fringe)

- reactome-egfr
  [+]

  No paths found.
  - AKA11\_HUMAN was not found.

#### 4. Resources that don't match any endpoint

- kegg-mapk
  [+]

  No paths found.
  - KAP2\_HUMAN was not found.
  - AKA11\_HUMAN was not found.
- layek
  [+]

  No paths found.
  - KAP2\_HUMAN was not found.
  - AKA11\_HUMAN was not found.
- science-signaling-egfr
  [+]

  No paths found.
  - KAP2\_HUMAN was not found.
  - AKA11\_HUMAN was not found.
- cancer-cell-map-egfr1
  [+]

  No paths found.
  - KAP2\_HUMAN was not found.
  - AKA11\_HUMAN was not found.
- kegg-erbb
  [+]

  No paths found.
  - KAP2\_HUMAN was not found.
  - AKA11\_HUMAN was not found.
- pid-erbb1
  [+]

  No paths found.
  - KAP2\_HUMAN was not found.
  - AKA11\_HUMAN was not found.
- biocarta-egf
  [+]

  No paths found.
  - KAP2\_HUMAN was not found.
  - AKA11\_HUMAN was not found.
- phosphositeplus-kinase-substrate
  [+]

  No paths found.
  - KAP2\_HUMAN was not found.
  - AKA11\_HUMAN was not found.

### KAP2\_HUMAN → AKAP2\_HUMAN / KAP2\_HUMAN ⊣ AKAP2\_HUMAN

#### 1. Resources that have the edge


#### 2. Resources that contain endpoints (but not an edge)


#### 3. Resources that match only one endpoint (fringe)

- reactome-egfr
  [+]

  No paths found.
  - AKAP2\_HUMAN was not found.

#### 4. Resources that don't match any endpoint

- kegg-erbb
  [+]

  No paths found.
  - KAP2\_HUMAN was not found.
  - AKAP2\_HUMAN was not found.
- layek
  [+]

  No paths found.
  - KAP2\_HUMAN was not found.
  - AKAP2\_HUMAN was not found.
- science-signaling-egfr
  [+]

  No paths found.
  - KAP2\_HUMAN was not found.
  - AKAP2\_HUMAN was not found.
- kegg-mapk
  [+]

  No paths found.
  - KAP2\_HUMAN was not found.
  - AKAP2\_HUMAN was not found.
- pid-erbb1
  [+]

  No paths found.
  - KAP2\_HUMAN was not found.
  - AKAP2\_HUMAN was not found.
- phosphositeplus-kinase-substrate
  [+]

  No paths found.
  - KAP2\_HUMAN was not found.
  - AKAP2\_HUMAN was not found.
- biocarta-egf
  [+]

  No paths found.
  - KAP2\_HUMAN was not found.
  - AKAP2\_HUMAN was not found.
- cancer-cell-map-egfr1
  [+]

  No paths found.
  - KAP2\_HUMAN was not found.
  - AKAP2\_HUMAN was not found.

### SAT1\_HUMAN → RLA1\_HUMAN / SAT1\_HUMAN ⊣ RLA1\_HUMAN

#### 1. Resources that have the edge


#### 2. Resources that contain endpoints (but not an edge)


#### 3. Resources that match only one endpoint (fringe)

- phosphositeplus-kinase-substrate
  [+]

  No paths found.
  - RLA1\_HUMAN was not found.

#### 4. Resources that don't match any endpoint

- biocarta-egf
  [+]

  No paths found.
  - RLA1\_HUMAN was not found.
  - SAT1\_HUMAN was not found.
- pid-erbb1
  [+]

  No paths found.
  - RLA1\_HUMAN was not found.
  - SAT1\_HUMAN was not found.
- kegg-erbb
  [+]

  No paths found.
  - RLA1\_HUMAN was not found.
  - SAT1\_HUMAN was not found.
- layek
  [+]

  No paths found.
  - RLA1\_HUMAN was not found.
  - SAT1\_HUMAN was not found.
- reactome-egfr
  [+]

  No paths found.
  - RLA1\_HUMAN was not found.
  - SAT1\_HUMAN was not found.
- kegg-mapk
  [+]

  No paths found.
  - RLA1\_HUMAN was not found.
  - SAT1\_HUMAN was not found.
- cancer-cell-map-egfr1
  [+]

  No paths found.
  - RLA1\_HUMAN was not found.
  - SAT1\_HUMAN was not found.
- science-signaling-egfr
  [+]

  No paths found.
  - RLA1\_HUMAN was not found.
  - SAT1\_HUMAN was not found.

### 1433G\_HUMAN → KLC4\_HUMAN / 1433G\_HUMAN ⊣ KLC4\_HUMAN

#### 1. Resources that have the edge


#### 2. Resources that contain endpoints (but not an edge)


#### 3. Resources that match only one endpoint (fringe)

- phosphositeplus-kinase-substrate
  [+]

  No paths found.
  - KLC4\_HUMAN was not found.

#### 4. Resources that don't match any endpoint

- kegg-erbb
  [+]

  No paths found.
  - 1433G\_HUMAN was not found.
  - KLC4\_HUMAN was not found.
- biocarta-egf
  [+]

  No paths found.
  - 1433G\_HUMAN was not found.
  - KLC4\_HUMAN was not found.
- cancer-cell-map-egfr1
  [+]

  No paths found.
  - 1433G\_HUMAN was not found.
  - KLC4\_HUMAN was not found.
- pid-erbb1
  [+]

  No paths found.
  - 1433G\_HUMAN was not found.
  - KLC4\_HUMAN was not found.
- kegg-mapk
  [+]

  No paths found.
  - 1433G\_HUMAN was not found.
  - KLC4\_HUMAN was not found.
- layek
  [+]

  No paths found.
  - 1433G\_HUMAN was not found.
  - KLC4\_HUMAN was not found.
- science-signaling-egfr
  [+]

  No paths found.
  - 1433G\_HUMAN was not found.
  - KLC4\_HUMAN was not found.
- reactome-egfr
  [+]

  No paths found.
  - 1433G\_HUMAN was not found.
  - KLC4\_HUMAN was not found.

### CSK21\_HUMAN → RPRD2\_HUMAN / CSK21\_HUMAN ⊣ RPRD2\_HUMAN

#### 1. Resources that have the edge


#### 2. Resources that contain endpoints (but not an edge)


#### 3. Resources that match only one endpoint (fringe)

- phosphositeplus-kinase-substrate
  [+]

  No paths found.
  - RPRD2\_HUMAN was not found.

#### 4. Resources that don't match any endpoint

- reactome-egfr
  [+]

  No paths found.
  - CSK21\_HUMAN was not found.
  - RPRD2\_HUMAN was not found.
- kegg-erbb
  [+]

  No paths found.
  - CSK21\_HUMAN was not found.
  - RPRD2\_HUMAN was not found.
- kegg-mapk
  [+]

  No paths found.
  - CSK21\_HUMAN was not found.
  - RPRD2\_HUMAN was not found.
- cancer-cell-map-egfr1
  [+]

  No paths found.
  - CSK21\_HUMAN was not found.
  - RPRD2\_HUMAN was not found.
- science-signaling-egfr
  [+]

  No paths found.
  - CSK21\_HUMAN was not found.
  - RPRD2\_HUMAN was not found.
- layek
  [+]

  No paths found.
  - CSK21\_HUMAN was not found.
  - RPRD2\_HUMAN was not found.
- biocarta-egf
  [+]

  No paths found.
  - CSK21\_HUMAN was not found.
  - RPRD2\_HUMAN was not found.
- pid-erbb1
  [+]

  No paths found.
  - CSK21\_HUMAN was not found.
  - RPRD2\_HUMAN was not found.

### MPRI\_HUMAN → GGA3\_HUMAN / MPRI\_HUMAN ⊣ GGA3\_HUMAN

#### 1. Resources that have the edge


#### 2. Resources that contain endpoints (but not an edge)


#### 3. Resources that match only one endpoint (fringe)

- phosphositeplus-kinase-substrate
  [+]

  No paths found.
  - GGA3\_HUMAN was not found.

#### 4. Resources that don't match any endpoint

- biocarta-egf
  [+]

  No paths found.
  - GGA3\_HUMAN was not found.
  - MPRI\_HUMAN was not found.
- kegg-erbb
  [+]

  No paths found.
  - GGA3\_HUMAN was not found.
  - MPRI\_HUMAN was not found.
- science-signaling-egfr
  [+]

  No paths found.
  - GGA3\_HUMAN was not found.
  - MPRI\_HUMAN was not found.
- layek
  [+]

  No paths found.
  - GGA3\_HUMAN was not found.
  - MPRI\_HUMAN was not found.
- reactome-egfr
  [+]

  No paths found.
  - GGA3\_HUMAN was not found.
  - MPRI\_HUMAN was not found.
- cancer-cell-map-egfr1
  [+]

  No paths found.
  - GGA3\_HUMAN was not found.
  - MPRI\_HUMAN was not found.
- kegg-mapk
  [+]

  No paths found.
  - GGA3\_HUMAN was not found.
  - MPRI\_HUMAN was not found.
- pid-erbb1
  [+]

  No paths found.
  - GGA3\_HUMAN was not found.
  - MPRI\_HUMAN was not found.

### CHD4\_HUMAN → SMCA4\_HUMAN

#### 1. Resources that have the edge


#### 2. Resources that contain endpoints (but not an edge)


#### 3. Resources that match only one endpoint (fringe)

- phosphositeplus-kinase-substrate
  [+]

  No paths found.
  - SMCA4\_HUMAN was not found.

#### 4. Resources that don't match any endpoint

- kegg-erbb
  [+]

  No paths found.
  - CHD4\_HUMAN was not found.
  - SMCA4\_HUMAN was not found.
- layek
  [+]

  No paths found.
  - CHD4\_HUMAN was not found.
  - SMCA4\_HUMAN was not found.
- reactome-egfr
  [+]

  No paths found.
  - CHD4\_HUMAN was not found.
  - SMCA4\_HUMAN was not found.
- cancer-cell-map-egfr1
  [+]

  No paths found.
  - CHD4\_HUMAN was not found.
  - SMCA4\_HUMAN was not found.
- kegg-mapk
  [+]

  No paths found.
  - CHD4\_HUMAN was not found.
  - SMCA4\_HUMAN was not found.
- biocarta-egf
  [+]

  No paths found.
  - CHD4\_HUMAN was not found.
  - SMCA4\_HUMAN was not found.
- science-signaling-egfr
  [+]

  No paths found.
  - CHD4\_HUMAN was not found.
  - SMCA4\_HUMAN was not found.
- pid-erbb1
  [+]

  No paths found.
  - CHD4\_HUMAN was not found.
  - SMCA4\_HUMAN was not found.

### CSK21\_HUMAN → ICLN\_HUMAN / CSK21\_HUMAN ⊣ ICLN\_HUMAN

#### 1. Resources that have the edge


#### 2. Resources that contain endpoints (but not an edge)


#### 3. Resources that match only one endpoint (fringe)

- phosphositeplus-kinase-substrate
  [+]

  No paths found.
  - ICLN\_HUMAN was not found.

#### 4. Resources that don't match any endpoint

- reactome-egfr
  [+]

  No paths found.
  - CSK21\_HUMAN was not found.
  - ICLN\_HUMAN was not found.
- science-signaling-egfr
  [+]

  No paths found.
  - CSK21\_HUMAN was not found.
  - ICLN\_HUMAN was not found.
- layek
  [+]

  No paths found.
  - CSK21\_HUMAN was not found.
  - ICLN\_HUMAN was not found.
- kegg-mapk
  [+]

  No paths found.
  - CSK21\_HUMAN was not found.
  - ICLN\_HUMAN was not found.
- biocarta-egf
  [+]

  No paths found.
  - CSK21\_HUMAN was not found.
  - ICLN\_HUMAN was not found.
- cancer-cell-map-egfr1
  [+]

  No paths found.
  - CSK21\_HUMAN was not found.
  - ICLN\_HUMAN was not found.
- kegg-erbb
  [+]

  No paths found.
  - CSK21\_HUMAN was not found.
  - ICLN\_HUMAN was not found.
- pid-erbb1
  [+]

  No paths found.
  - CSK21\_HUMAN was not found.
  - ICLN\_HUMAN was not found.

### SAT1\_HUMAN → FUND2\_HUMAN / SAT1\_HUMAN ⊣ FUND2\_HUMAN

#### 1. Resources that have the edge


#### 2. Resources that contain endpoints (but not an edge)


#### 3. Resources that match only one endpoint (fringe)

- phosphositeplus-kinase-substrate
  [+]

  No paths found.
  - FUND2\_HUMAN was not found.

#### 4. Resources that don't match any endpoint

- biocarta-egf
  [+]

  No paths found.
  - SAT1\_HUMAN was not found.
  - FUND2\_HUMAN was not found.
- reactome-egfr
  [+]

  No paths found.
  - SAT1\_HUMAN was not found.
  - FUND2\_HUMAN was not found.
- kegg-mapk
  [+]

  No paths found.
  - SAT1\_HUMAN was not found.
  - FUND2\_HUMAN was not found.
- science-signaling-egfr
  [+]

  No paths found.
  - SAT1\_HUMAN was not found.
  - FUND2\_HUMAN was not found.
- layek
  [+]

  No paths found.
  - SAT1\_HUMAN was not found.
  - FUND2\_HUMAN was not found.
- pid-erbb1
  [+]

  No paths found.
  - SAT1\_HUMAN was not found.
  - FUND2\_HUMAN was not found.
- kegg-erbb
  [+]

  No paths found.
  - SAT1\_HUMAN was not found.
  - FUND2\_HUMAN was not found.
- cancer-cell-map-egfr1
  [+]

  No paths found.
  - SAT1\_HUMAN was not found.
  - FUND2\_HUMAN was not found.

### 1433G\_HUMAN → PANK2\_HUMAN / 1433G\_HUMAN ⊣ PANK2\_HUMAN

#### 1. Resources that have the edge


#### 2. Resources that contain endpoints (but not an edge)


#### 3. Resources that match only one endpoint (fringe)

- phosphositeplus-kinase-substrate
  [+]

  No paths found.
  - PANK2\_HUMAN was not found.

#### 4. Resources that don't match any endpoint

- science-signaling-egfr
  [+]

  No paths found.
  - 1433G\_HUMAN was not found.
  - PANK2\_HUMAN was not found.
- kegg-erbb
  [+]

  No paths found.
  - 1433G\_HUMAN was not found.
  - PANK2\_HUMAN was not found.
- cancer-cell-map-egfr1
  [+]

  No paths found.
  - 1433G\_HUMAN was not found.
  - PANK2\_HUMAN was not found.
- biocarta-egf
  [+]

  No paths found.
  - 1433G\_HUMAN was not found.
  - PANK2\_HUMAN was not found.
- pid-erbb1
  [+]

  No paths found.
  - 1433G\_HUMAN was not found.
  - PANK2\_HUMAN was not found.
- layek
  [+]

  No paths found.
  - 1433G\_HUMAN was not found.
  - PANK2\_HUMAN was not found.
- reactome-egfr
  [+]

  No paths found.
  - 1433G\_HUMAN was not found.
  - PANK2\_HUMAN was not found.
- kegg-mapk
  [+]

  No paths found.
  - 1433G\_HUMAN was not found.
  - PANK2\_HUMAN was not found.

### 1433G\_HUMAN → ZO2\_HUMAN / 1433G\_HUMAN ⊣ ZO2\_HUMAN

#### 1. Resources that have the edge


#### 2. Resources that contain endpoints (but not an edge)


#### 3. Resources that match only one endpoint (fringe)

- phosphositeplus-kinase-substrate
  [+]

  No paths found.
  - ZO2\_HUMAN was not found.

#### 4. Resources that don't match any endpoint

- pid-erbb1
  [+]

  No paths found.
  - 1433G\_HUMAN was not found.
  - ZO2\_HUMAN was not found.
- cancer-cell-map-egfr1
  [+]

  No paths found.
  - 1433G\_HUMAN was not found.
  - ZO2\_HUMAN was not found.
- kegg-erbb
  [+]

  No paths found.
  - 1433G\_HUMAN was not found.
  - ZO2\_HUMAN was not found.
- reactome-egfr
  [+]

  No paths found.
  - 1433G\_HUMAN was not found.
  - ZO2\_HUMAN was not found.
- layek
  [+]

  No paths found.
  - 1433G\_HUMAN was not found.
  - ZO2\_HUMAN was not found.
- kegg-mapk
  [+]

  No paths found.
  - 1433G\_HUMAN was not found.
  - ZO2\_HUMAN was not found.
- science-signaling-egfr
  [+]

  No paths found.
  - 1433G\_HUMAN was not found.
  - ZO2\_HUMAN was not found.
- biocarta-egf
  [+]

  No paths found.
  - 1433G\_HUMAN was not found.
  - ZO2\_HUMAN was not found.

### ZYX\_HUMAN → HIPK3\_HUMAN / ZYX\_HUMAN ⊣ HIPK3\_HUMAN

#### 1. Resources that have the edge


#### 2. Resources that contain endpoints (but not an edge)


#### 3. Resources that match only one endpoint (fringe)

- phosphositeplus-kinase-substrate
  [+]

  No paths found.
  - HIPK3\_HUMAN was not found.

#### 4. Resources that don't match any endpoint

- science-signaling-egfr
  [+]

  No paths found.
  - ZYX\_HUMAN was not found.
  - HIPK3\_HUMAN was not found.
- pid-erbb1
  [+]

  No paths found.
  - ZYX\_HUMAN was not found.
  - HIPK3\_HUMAN was not found.
- kegg-erbb
  [+]

  No paths found.
  - ZYX\_HUMAN was not found.
  - HIPK3\_HUMAN was not found.
- biocarta-egf
  [+]

  No paths found.
  - ZYX\_HUMAN was not found.
  - HIPK3\_HUMAN was not found.
- cancer-cell-map-egfr1
  [+]

  No paths found.
  - ZYX\_HUMAN was not found.
  - HIPK3\_HUMAN was not found.
- reactome-egfr
  [+]

  No paths found.
  - ZYX\_HUMAN was not found.
  - HIPK3\_HUMAN was not found.
- kegg-mapk
  [+]

  No paths found.
  - ZYX\_HUMAN was not found.
  - HIPK3\_HUMAN was not found.
- layek
  [+]

  No paths found.
  - ZYX\_HUMAN was not found.
  - HIPK3\_HUMAN was not found.

### 1433G\_HUMAN → PPR3D\_HUMAN / 1433G\_HUMAN ⊣ PPR3D\_HUMAN

#### 1. Resources that have the edge


#### 2. Resources that contain endpoints (but not an edge)


#### 3. Resources that match only one endpoint (fringe)

- phosphositeplus-kinase-substrate
  [+]

  No paths found.
  - PPR3D\_HUMAN was not found.

#### 4. Resources that don't match any endpoint

- layek
  [+]

  No paths found.
  - 1433G\_HUMAN was not found.
  - PPR3D\_HUMAN was not found.
- cancer-cell-map-egfr1
  [+]

  No paths found.
  - 1433G\_HUMAN was not found.
  - PPR3D\_HUMAN was not found.
- science-signaling-egfr
  [+]

  No paths found.
  - 1433G\_HUMAN was not found.
  - PPR3D\_HUMAN was not found.
- kegg-mapk
  [+]

  No paths found.
  - 1433G\_HUMAN was not found.
  - PPR3D\_HUMAN was not found.
- biocarta-egf
  [+]

  No paths found.
  - 1433G\_HUMAN was not found.
  - PPR3D\_HUMAN was not found.
- kegg-erbb
  [+]

  No paths found.
  - 1433G\_HUMAN was not found.
  - PPR3D\_HUMAN was not found.
- pid-erbb1
  [+]

  No paths found.
  - 1433G\_HUMAN was not found.
  - PPR3D\_HUMAN was not found.
- reactome-egfr
  [+]

  No paths found.
  - 1433G\_HUMAN was not found.
  - PPR3D\_HUMAN was not found.

### 41\_HUMAN → PSME3\_HUMAN / 41\_HUMAN ⊣ PSME3\_HUMAN

#### 1. Resources that have the edge


#### 2. Resources that contain endpoints (but not an edge)


#### 3. Resources that match only one endpoint (fringe)

- phosphositeplus-kinase-substrate
  [+]

  No paths found.
  - PSME3\_HUMAN was not found.

#### 4. Resources that don't match any endpoint

- biocarta-egf
  [+]

  No paths found.
  - 41\_HUMAN was not found.
  - PSME3\_HUMAN was not found.
- science-signaling-egfr
  [+]

  No paths found.
  - 41\_HUMAN was not found.
  - PSME3\_HUMAN was not found.
- pid-erbb1
  [+]

  No paths found.
  - 41\_HUMAN was not found.
  - PSME3\_HUMAN was not found.
- kegg-erbb
  [+]

  No paths found.
  - 41\_HUMAN was not found.
  - PSME3\_HUMAN was not found.
- layek
  [+]

  No paths found.
  - 41\_HUMAN was not found.
  - PSME3\_HUMAN was not found.
- kegg-mapk
  [+]

  No paths found.
  - 41\_HUMAN was not found.
  - PSME3\_HUMAN was not found.
- reactome-egfr
  [+]

  No paths found.
  - 41\_HUMAN was not found.
  - PSME3\_HUMAN was not found.
- cancer-cell-map-egfr1
  [+]

  No paths found.
  - 41\_HUMAN was not found.
  - PSME3\_HUMAN was not found.

### 1433G\_HUMAN → CLAP1\_HUMAN / 1433G\_HUMAN ⊣ CLAP1\_HUMAN

#### 1. Resources that have the edge


#### 2. Resources that contain endpoints (but not an edge)


#### 3. Resources that match only one endpoint (fringe)

- phosphositeplus-kinase-substrate
  [+]

  No paths found.
  - CLAP1\_HUMAN was not found.

#### 4. Resources that don't match any endpoint

- biocarta-egf
  [+]

  No paths found.
  - 1433G\_HUMAN was not found.
  - CLAP1\_HUMAN was not found.
- reactome-egfr
  [+]

  No paths found.
  - 1433G\_HUMAN was not found.
  - CLAP1\_HUMAN was not found.
- kegg-mapk
  [+]

  No paths found.
  - 1433G\_HUMAN was not found.
  - CLAP1\_HUMAN was not found.
- layek
  [+]

  No paths found.
  - 1433G\_HUMAN was not found.
  - CLAP1\_HUMAN was not found.
- kegg-erbb
  [+]

  No paths found.
  - 1433G\_HUMAN was not found.
  - CLAP1\_HUMAN was not found.
- cancer-cell-map-egfr1
  [+]

  No paths found.
  - 1433G\_HUMAN was not found.
  - CLAP1\_HUMAN was not found.
- science-signaling-egfr
  [+]

  No paths found.
  - 1433G\_HUMAN was not found.
  - CLAP1\_HUMAN was not found.
- pid-erbb1
  [+]

  No paths found.
  - 1433G\_HUMAN was not found.
  - CLAP1\_HUMAN was not found.

### CSK21\_HUMAN → LYRIC\_HUMAN / CSK21\_HUMAN ⊣ LYRIC\_HUMAN

#### 1. Resources that have the edge


#### 2. Resources that contain endpoints (but not an edge)


#### 3. Resources that match only one endpoint (fringe)

- phosphositeplus-kinase-substrate
  [+]

  No paths found.
  - LYRIC\_HUMAN was not found.

#### 4. Resources that don't match any endpoint

- cancer-cell-map-egfr1
  [+]

  No paths found.
  - CSK21\_HUMAN was not found.
  - LYRIC\_HUMAN was not found.
- biocarta-egf
  [+]

  No paths found.
  - CSK21\_HUMAN was not found.
  - LYRIC\_HUMAN was not found.
- kegg-erbb
  [+]

  No paths found.
  - CSK21\_HUMAN was not found.
  - LYRIC\_HUMAN was not found.
- kegg-mapk
  [+]

  No paths found.
  - CSK21\_HUMAN was not found.
  - LYRIC\_HUMAN was not found.
- science-signaling-egfr
  [+]

  No paths found.
  - CSK21\_HUMAN was not found.
  - LYRIC\_HUMAN was not found.
- reactome-egfr
  [+]

  No paths found.
  - CSK21\_HUMAN was not found.
  - LYRIC\_HUMAN was not found.
- layek
  [+]

  No paths found.
  - CSK21\_HUMAN was not found.
  - LYRIC\_HUMAN was not found.
- pid-erbb1
  [+]

  No paths found.
  - CSK21\_HUMAN was not found.
  - LYRIC\_HUMAN was not found.

### 1433G\_HUMAN → PPIG\_HUMAN / 1433G\_HUMAN ⊣ PPIG\_HUMAN

#### 1. Resources that have the edge


#### 2. Resources that contain endpoints (but not an edge)


#### 3. Resources that match only one endpoint (fringe)

- phosphositeplus-kinase-substrate
  [+]

  No paths found.
  - PPIG\_HUMAN was not found.

#### 4. Resources that don't match any endpoint

- biocarta-egf
  [+]

  No paths found.
  - 1433G\_HUMAN was not found.
  - PPIG\_HUMAN was not found.
- science-signaling-egfr
  [+]

  No paths found.
  - 1433G\_HUMAN was not found.
  - PPIG\_HUMAN was not found.
- cancer-cell-map-egfr1
  [+]

  No paths found.
  - 1433G\_HUMAN was not found.
  - PPIG\_HUMAN was not found.
- kegg-erbb
  [+]

  No paths found.
  - 1433G\_HUMAN was not found.
  - PPIG\_HUMAN was not found.
- layek
  [+]

  No paths found.
  - 1433G\_HUMAN was not found.
  - PPIG\_HUMAN was not found.
- pid-erbb1
  [+]

  No paths found.
  - 1433G\_HUMAN was not found.
  - PPIG\_HUMAN was not found.
- reactome-egfr
  [+]

  No paths found.
  - 1433G\_HUMAN was not found.
  - PPIG\_HUMAN was not found.
- kegg-mapk
  [+]

  No paths found.
  - 1433G\_HUMAN was not found.
  - PPIG\_HUMAN was not found.

### SUMO2\_HUMAN → RBP2\_HUMAN / SUMO2\_HUMAN ⊣ RBP2\_HUMAN

#### 1. Resources that have the edge


#### 2. Resources that contain endpoints (but not an edge)


#### 3. Resources that match only one endpoint (fringe)

- phosphositeplus-kinase-substrate
  [+]

  No paths found.
  - SUMO2\_HUMAN was not found.

#### 4. Resources that don't match any endpoint

- pid-erbb1
  [+]

  No paths found.
  - RBP2\_HUMAN was not found.
  - SUMO2\_HUMAN was not found.
- kegg-mapk
  [+]

  No paths found.
  - RBP2\_HUMAN was not found.
  - SUMO2\_HUMAN was not found.
- kegg-erbb
  [+]

  No paths found.
  - RBP2\_HUMAN was not found.
  - SUMO2\_HUMAN was not found.
- reactome-egfr
  [+]

  No paths found.
  - RBP2\_HUMAN was not found.
  - SUMO2\_HUMAN was not found.
- cancer-cell-map-egfr1
  [+]

  No paths found.
  - RBP2\_HUMAN was not found.
  - SUMO2\_HUMAN was not found.
- biocarta-egf
  [+]

  No paths found.
  - RBP2\_HUMAN was not found.
  - SUMO2\_HUMAN was not found.
- layek
  [+]

  No paths found.
  - RBP2\_HUMAN was not found.
  - SUMO2\_HUMAN was not found.
- science-signaling-egfr
  [+]

  No paths found.
  - RBP2\_HUMAN was not found.
  - SUMO2\_HUMAN was not found.

### ZYX\_HUMAN → UBL7\_HUMAN / ZYX\_HUMAN ⊣ UBL7\_HUMAN

#### 1. Resources that have the edge


#### 2. Resources that contain endpoints (but not an edge)


#### 3. Resources that match only one endpoint (fringe)

- phosphositeplus-kinase-substrate
  [+]

  No paths found.
  - UBL7\_HUMAN was not found.

#### 4. Resources that don't match any endpoint

- reactome-egfr
  [+]

  No paths found.
  - ZYX\_HUMAN was not found.
  - UBL7\_HUMAN was not found.
- science-signaling-egfr
  [+]

  No paths found.
  - ZYX\_HUMAN was not found.
  - UBL7\_HUMAN was not found.
- layek
  [+]

  No paths found.
  - ZYX\_HUMAN was not found.
  - UBL7\_HUMAN was not found.
- cancer-cell-map-egfr1
  [+]

  No paths found.
  - ZYX\_HUMAN was not found.
  - UBL7\_HUMAN was not found.
- kegg-erbb
  [+]

  No paths found.
  - ZYX\_HUMAN was not found.
  - UBL7\_HUMAN was not found.
- biocarta-egf
  [+]

  No paths found.
  - ZYX\_HUMAN was not found.
  - UBL7\_HUMAN was not found.
- kegg-mapk
  [+]

  No paths found.
  - ZYX\_HUMAN was not found.
  - UBL7\_HUMAN was not found.
- pid-erbb1
  [+]

  No paths found.
  - ZYX\_HUMAN was not found.
  - UBL7\_HUMAN was not found.

### SUMO2\_HUMAN → CLSPN\_HUMAN / SUMO2\_HUMAN ⊣ CLSPN\_HUMAN

#### 1. Resources that have the edge


#### 2. Resources that contain endpoints (but not an edge)


#### 3. Resources that match only one endpoint (fringe)

- phosphositeplus-kinase-substrate
  [+]

  No paths found.
  - SUMO2\_HUMAN was not found.

#### 4. Resources that don't match any endpoint

- pid-erbb1
  [+]

  No paths found.
  - CLSPN\_HUMAN was not found.
  - SUMO2\_HUMAN was not found.
- kegg-erbb
  [+]

  No paths found.
  - CLSPN\_HUMAN was not found.
  - SUMO2\_HUMAN was not found.
- science-signaling-egfr
  [+]

  No paths found.
  - CLSPN\_HUMAN was not found.
  - SUMO2\_HUMAN was not found.
- biocarta-egf
  [+]

  No paths found.
  - CLSPN\_HUMAN was not found.
  - SUMO2\_HUMAN was not found.
- cancer-cell-map-egfr1
  [+]

  No paths found.
  - CLSPN\_HUMAN was not found.
  - SUMO2\_HUMAN was not found.
- kegg-mapk
  [+]

  No paths found.
  - CLSPN\_HUMAN was not found.
  - SUMO2\_HUMAN was not found.
- layek
  [+]

  No paths found.
  - CLSPN\_HUMAN was not found.
  - SUMO2\_HUMAN was not found.
- reactome-egfr
  [+]

  No paths found.
  - CLSPN\_HUMAN was not found.
  - SUMO2\_HUMAN was not found.

### 1433G\_HUMAN → ZN638\_HUMAN / 1433G\_HUMAN ⊣ ZN638\_HUMAN

#### 1. Resources that have the edge


#### 2. Resources that contain endpoints (but not an edge)


#### 3. Resources that match only one endpoint (fringe)

- phosphositeplus-kinase-substrate
  [+]

  No paths found.
  - ZN638\_HUMAN was not found.

#### 4. Resources that don't match any endpoint

- science-signaling-egfr
  [+]

  No paths found.
  - 1433G\_HUMAN was not found.
  - ZN638\_HUMAN was not found.
- pid-erbb1
  [+]

  No paths found.
  - 1433G\_HUMAN was not found.
  - ZN638\_HUMAN was not found.
- reactome-egfr
  [+]

  No paths found.
  - 1433G\_HUMAN was not found.
  - ZN638\_HUMAN was not found.
- biocarta-egf
  [+]

  No paths found.
  - 1433G\_HUMAN was not found.
  - ZN638\_HUMAN was not found.
- kegg-mapk
  [+]

  No paths found.
  - 1433G\_HUMAN was not found.
  - ZN638\_HUMAN was not found.
- layek
  [+]

  No paths found.
  - 1433G\_HUMAN was not found.
  - ZN638\_HUMAN was not found.
- cancer-cell-map-egfr1
  [+]

  No paths found.
  - 1433G\_HUMAN was not found.
  - ZN638\_HUMAN was not found.
- kegg-erbb
  [+]

  No paths found.
  - 1433G\_HUMAN was not found.
  - ZN638\_HUMAN was not found.

### CSK21\_HUMAN → AT2B1\_HUMAN / CSK21\_HUMAN ⊣ AT2B1\_HUMAN

#### 1. Resources that have the edge


#### 2. Resources that contain endpoints (but not an edge)


#### 3. Resources that match only one endpoint (fringe)

- phosphositeplus-kinase-substrate
  [+]

  No paths found.
  - AT2B1\_HUMAN was not found.

#### 4. Resources that don't match any endpoint

- layek
  [+]

  No paths found.
  - CSK21\_HUMAN was not found.
  - AT2B1\_HUMAN was not found.
- reactome-egfr
  [+]

  No paths found.
  - CSK21\_HUMAN was not found.
  - AT2B1\_HUMAN was not found.
- kegg-mapk
  [+]

  No paths found.
  - CSK21\_HUMAN was not found.
  - AT2B1\_HUMAN was not found.
- cancer-cell-map-egfr1
  [+]

  No paths found.
  - CSK21\_HUMAN was not found.
  - AT2B1\_HUMAN was not found.
- kegg-erbb
  [+]

  No paths found.
  - CSK21\_HUMAN was not found.
  - AT2B1\_HUMAN was not found.
- science-signaling-egfr
  [+]

  No paths found.
  - CSK21\_HUMAN was not found.
  - AT2B1\_HUMAN was not found.
- pid-erbb1
  [+]

  No paths found.
  - CSK21\_HUMAN was not found.
  - AT2B1\_HUMAN was not found.
- biocarta-egf
  [+]

  No paths found.
  - CSK21\_HUMAN was not found.
  - AT2B1\_HUMAN was not found.

### CSK21\_HUMAN → VAMP4\_HUMAN / CSK21\_HUMAN ⊣ VAMP4\_HUMAN

#### 1. Resources that have the edge


#### 2. Resources that contain endpoints (but not an edge)


#### 3. Resources that match only one endpoint (fringe)

- phosphositeplus-kinase-substrate
  [+]

  No paths found.
  - VAMP4\_HUMAN was not found.

#### 4. Resources that don't match any endpoint

- reactome-egfr
  [+]

  No paths found.
  - CSK21\_HUMAN was not found.
  - VAMP4\_HUMAN was not found.
- science-signaling-egfr
  [+]

  No paths found.
  - CSK21\_HUMAN was not found.
  - VAMP4\_HUMAN was not found.
- biocarta-egf
  [+]

  No paths found.
  - CSK21\_HUMAN was not found.
  - VAMP4\_HUMAN was not found.
- cancer-cell-map-egfr1
  [+]

  No paths found.
  - CSK21\_HUMAN was not found.
  - VAMP4\_HUMAN was not found.
- kegg-mapk
  [+]

  No paths found.
  - CSK21\_HUMAN was not found.
  - VAMP4\_HUMAN was not found.
- pid-erbb1
  [+]

  No paths found.
  - CSK21\_HUMAN was not found.
  - VAMP4\_HUMAN was not found.
- layek
  [+]

  No paths found.
  - CSK21\_HUMAN was not found.
  - VAMP4\_HUMAN was not found.
- kegg-erbb
  [+]

  No paths found.
  - CSK21\_HUMAN was not found.
  - VAMP4\_HUMAN was not found.

### 1433G\_HUMAN → E41L2\_HUMAN / 1433G\_HUMAN ⊣ E41L2\_HUMAN

#### 1. Resources that have the edge


#### 2. Resources that contain endpoints (but not an edge)


#### 3. Resources that match only one endpoint (fringe)

- phosphositeplus-kinase-substrate
  [+]

  No paths found.
  - E41L2\_HUMAN was not found.

#### 4. Resources that don't match any endpoint

- science-signaling-egfr
  [+]

  No paths found.
  - 1433G\_HUMAN was not found.
  - E41L2\_HUMAN was not found.
- cancer-cell-map-egfr1
  [+]

  No paths found.
  - 1433G\_HUMAN was not found.
  - E41L2\_HUMAN was not found.
- kegg-mapk
  [+]

  No paths found.
  - 1433G\_HUMAN was not found.
  - E41L2\_HUMAN was not found.
- biocarta-egf
  [+]

  No paths found.
  - 1433G\_HUMAN was not found.
  - E41L2\_HUMAN was not found.
- kegg-erbb
  [+]

  No paths found.
  - 1433G\_HUMAN was not found.
  - E41L2\_HUMAN was not found.
- layek
  [+]

  No paths found.
  - 1433G\_HUMAN was not found.
  - E41L2\_HUMAN was not found.
- pid-erbb1
  [+]

  No paths found.
  - 1433G\_HUMAN was not found.
  - E41L2\_HUMAN was not found.
- reactome-egfr
  [+]

  No paths found.
  - 1433G\_HUMAN was not found.
  - E41L2\_HUMAN was not found.

### 1433G\_HUMAN → TRA2A\_HUMAN / 1433G\_HUMAN ⊣ TRA2A\_HUMAN

#### 1. Resources that have the edge


#### 2. Resources that contain endpoints (but not an edge)


#### 3. Resources that match only one endpoint (fringe)

- phosphositeplus-kinase-substrate
  [+]

  No paths found.
  - TRA2A\_HUMAN was not found.

#### 4. Resources that don't match any endpoint

- pid-erbb1
  [+]

  No paths found.
  - TRA2A\_HUMAN was not found.
  - 1433G\_HUMAN was not found.
- layek
  [+]

  No paths found.
  - TRA2A\_HUMAN was not found.
  - 1433G\_HUMAN was not found.
- cancer-cell-map-egfr1
  [+]

  No paths found.
  - TRA2A\_HUMAN was not found.
  - 1433G\_HUMAN was not found.
- kegg-erbb
  [+]

  No paths found.
  - TRA2A\_HUMAN was not found.
  - 1433G\_HUMAN was not found.
- science-signaling-egfr
  [+]

  No paths found.
  - TRA2A\_HUMAN was not found.
  - 1433G\_HUMAN was not found.
- biocarta-egf
  [+]

  No paths found.
  - TRA2A\_HUMAN was not found.
  - 1433G\_HUMAN was not found.
- kegg-mapk
  [+]

  No paths found.
  - TRA2A\_HUMAN was not found.
  - 1433G\_HUMAN was not found.
- reactome-egfr
  [+]

  No paths found.
  - TRA2A\_HUMAN was not found.
  - 1433G\_HUMAN was not found.

### EPHA2\_HUMAN → EFNA5\_HUMAN / EPHA2\_HUMAN ⊣ EFNA5\_HUMAN

#### 1. Resources that have the edge


#### 2. Resources that contain endpoints (but not an edge)


#### 3. Resources that match only one endpoint (fringe)

- phosphositeplus-kinase-substrate
  [+]

  No paths found.
  - EFNA5\_HUMAN was not found.

#### 4. Resources that don't match any endpoint

- kegg-erbb
  [+]

  No paths found.
  - EPHA2\_HUMAN was not found.
  - EFNA5\_HUMAN was not found.
- pid-erbb1
  [+]

  No paths found.
  - EPHA2\_HUMAN was not found.
  - EFNA5\_HUMAN was not found.
- kegg-mapk
  [+]

  No paths found.
  - EPHA2\_HUMAN was not found.
  - EFNA5\_HUMAN was not found.
- cancer-cell-map-egfr1
  [+]

  No paths found.
  - EPHA2\_HUMAN was not found.
  - EFNA5\_HUMAN was not found.
- biocarta-egf
  [+]

  No paths found.
  - EPHA2\_HUMAN was not found.
  - EFNA5\_HUMAN was not found.
- layek
  [+]

  No paths found.
  - EPHA2\_HUMAN was not found.
  - EFNA5\_HUMAN was not found.
- reactome-egfr
  [+]

  No paths found.
  - EPHA2\_HUMAN was not found.
  - EFNA5\_HUMAN was not found.
- science-signaling-egfr
  [+]

  No paths found.
  - EPHA2\_HUMAN was not found.
  - EFNA5\_HUMAN was not found.

### CSK21\_HUMAN → CFDP1\_HUMAN / CSK21\_HUMAN ⊣ CFDP1\_HUMAN

#### 1. Resources that have the edge


#### 2. Resources that contain endpoints (but not an edge)


#### 3. Resources that match only one endpoint (fringe)

- phosphositeplus-kinase-substrate
  [+]

  No paths found.
  - CFDP1\_HUMAN was not found.

#### 4. Resources that don't match any endpoint

- kegg-mapk
  [+]

  No paths found.
  - CSK21\_HUMAN was not found.
  - CFDP1\_HUMAN was not found.
- kegg-erbb
  [+]

  No paths found.
  - CSK21\_HUMAN was not found.
  - CFDP1\_HUMAN was not found.
- pid-erbb1
  [+]

  No paths found.
  - CSK21\_HUMAN was not found.
  - CFDP1\_HUMAN was not found.
- cancer-cell-map-egfr1
  [+]

  No paths found.
  - CSK21\_HUMAN was not found.
  - CFDP1\_HUMAN was not found.
- reactome-egfr
  [+]

  No paths found.
  - CSK21\_HUMAN was not found.
  - CFDP1\_HUMAN was not found.
- layek
  [+]

  No paths found.
  - CSK21\_HUMAN was not found.
  - CFDP1\_HUMAN was not found.
- biocarta-egf
  [+]

  No paths found.
  - CSK21\_HUMAN was not found.
  - CFDP1\_HUMAN was not found.
- science-signaling-egfr
  [+]

  No paths found.
  - CSK21\_HUMAN was not found.
  - CFDP1\_HUMAN was not found.

### SCAM3\_HUMAN → HGS\_HUMAN

#### 1. Resources that have the edge


#### 2. Resources that contain endpoints (but not an edge)


#### 3. Resources that match only one endpoint (fringe)

- reactome-egfr
  [+]

  No paths found.
  - SCAM3\_HUMAN was not found.

#### 4. Resources that don't match any endpoint

- science-signaling-egfr
  [+]

  No paths found.
  - SCAM3\_HUMAN was not found.
  - HGS\_HUMAN was not found.
- layek
  [+]

  No paths found.
  - SCAM3\_HUMAN was not found.
  - HGS\_HUMAN was not found.
- phosphositeplus-kinase-substrate
  [+]

  No paths found.
  - SCAM3\_HUMAN was not found.
  - HGS\_HUMAN was not found.
- kegg-mapk
  [+]

  No paths found.
  - SCAM3\_HUMAN was not found.
  - HGS\_HUMAN was not found.
- pid-erbb1
  [+]

  No paths found.
  - SCAM3\_HUMAN was not found.
  - HGS\_HUMAN was not found.
- kegg-erbb
  [+]

  No paths found.
  - SCAM3\_HUMAN was not found.
  - HGS\_HUMAN was not found.
- cancer-cell-map-egfr1
  [+]

  No paths found.
  - SCAM3\_HUMAN was not found.
  - HGS\_HUMAN was not found.
- biocarta-egf
  [+]

  No paths found.
  - SCAM3\_HUMAN was not found.
  - HGS\_HUMAN was not found.

### NEK6\_HUMAN → HACD3\_HUMAN / NEK6\_HUMAN ⊣ HACD3\_HUMAN

#### 1. Resources that have the edge


#### 2. Resources that contain endpoints (but not an edge)


#### 3. Resources that match only one endpoint (fringe)

- phosphositeplus-kinase-substrate
  [+]

  No paths found.
  - HACD3\_HUMAN was not found.

#### 4. Resources that don't match any endpoint

- biocarta-egf
  [+]

  No paths found.
  - NEK6\_HUMAN was not found.
  - HACD3\_HUMAN was not found.
- pid-erbb1
  [+]

  No paths found.
  - NEK6\_HUMAN was not found.
  - HACD3\_HUMAN was not found.
- kegg-mapk
  [+]

  No paths found.
  - NEK6\_HUMAN was not found.
  - HACD3\_HUMAN was not found.
- reactome-egfr
  [+]

  No paths found.
  - NEK6\_HUMAN was not found.
  - HACD3\_HUMAN was not found.
- cancer-cell-map-egfr1
  [+]

  No paths found.
  - NEK6\_HUMAN was not found.
  - HACD3\_HUMAN was not found.
- layek
  [+]

  No paths found.
  - NEK6\_HUMAN was not found.
  - HACD3\_HUMAN was not found.
- kegg-erbb
  [+]

  No paths found.
  - NEK6\_HUMAN was not found.
  - HACD3\_HUMAN was not found.
- science-signaling-egfr
  [+]

  No paths found.
  - NEK6\_HUMAN was not found.
  - HACD3\_HUMAN was not found.

### 1433G\_HUMAN → FA53C\_HUMAN / 1433G\_HUMAN ⊣ FA53C\_HUMAN

#### 1. Resources that have the edge


#### 2. Resources that contain endpoints (but not an edge)


#### 3. Resources that match only one endpoint (fringe)

- phosphositeplus-kinase-substrate
  [+]

  No paths found.
  - FA53C\_HUMAN was not found.

#### 4. Resources that don't match any endpoint

- pid-erbb1
  [+]

  No paths found.
  - FA53C\_HUMAN was not found.
  - 1433G\_HUMAN was not found.
- kegg-erbb
  [+]

  No paths found.
  - FA53C\_HUMAN was not found.
  - 1433G\_HUMAN was not found.
- kegg-mapk
  [+]

  No paths found.
  - FA53C\_HUMAN was not found.
  - 1433G\_HUMAN was not found.
- layek
  [+]

  No paths found.
  - FA53C\_HUMAN was not found.
  - 1433G\_HUMAN was not found.
- biocarta-egf
  [+]

  No paths found.
  - FA53C\_HUMAN was not found.
  - 1433G\_HUMAN was not found.
- cancer-cell-map-egfr1
  [+]

  No paths found.
  - FA53C\_HUMAN was not found.
  - 1433G\_HUMAN was not found.
- science-signaling-egfr
  [+]

  No paths found.
  - FA53C\_HUMAN was not found.
  - 1433G\_HUMAN was not found.
- reactome-egfr
  [+]

  No paths found.
  - FA53C\_HUMAN was not found.
  - 1433G\_HUMAN was not found.

### CSK21\_HUMAN → AR6P4\_HUMAN / CSK21\_HUMAN ⊣ AR6P4\_HUMAN

#### 1. Resources that have the edge


#### 2. Resources that contain endpoints (but not an edge)


#### 3. Resources that match only one endpoint (fringe)

- phosphositeplus-kinase-substrate
  [+]

  No paths found.
  - AR6P4\_HUMAN was not found.

#### 4. Resources that don't match any endpoint

- kegg-erbb
  [+]

  No paths found.
  - CSK21\_HUMAN was not found.
  - AR6P4\_HUMAN was not found.
- kegg-mapk
  [+]

  No paths found.
  - CSK21\_HUMAN was not found.
  - AR6P4\_HUMAN was not found.
- science-signaling-egfr
  [+]

  No paths found.
  - CSK21\_HUMAN was not found.
  - AR6P4\_HUMAN was not found.
- biocarta-egf
  [+]

  No paths found.
  - CSK21\_HUMAN was not found.
  - AR6P4\_HUMAN was not found.
- reactome-egfr
  [+]

  No paths found.
  - CSK21\_HUMAN was not found.
  - AR6P4\_HUMAN was not found.
- pid-erbb1
  [+]

  No paths found.
  - CSK21\_HUMAN was not found.
  - AR6P4\_HUMAN was not found.
- layek
  [+]

  No paths found.
  - CSK21\_HUMAN was not found.
  - AR6P4\_HUMAN was not found.
- cancer-cell-map-egfr1
  [+]

  No paths found.
  - CSK21\_HUMAN was not found.
  - AR6P4\_HUMAN was not found.

### CSK21\_HUMAN → IF2P\_HUMAN / CSK21\_HUMAN ⊣ IF2P\_HUMAN

#### 1. Resources that have the edge


#### 2. Resources that contain endpoints (but not an edge)


#### 3. Resources that match only one endpoint (fringe)

- phosphositeplus-kinase-substrate
  [+]

  No paths found.
  - IF2P\_HUMAN was not found.

#### 4. Resources that don't match any endpoint

- kegg-mapk
  [+]

  No paths found.
  - IF2P\_HUMAN was not found.
  - CSK21\_HUMAN was not found.
- kegg-erbb
  [+]

  No paths found.
  - IF2P\_HUMAN was not found.
  - CSK21\_HUMAN was not found.
- reactome-egfr
  [+]

  No paths found.
  - IF2P\_HUMAN was not found.
  - CSK21\_HUMAN was not found.
- biocarta-egf
  [+]

  No paths found.
  - IF2P\_HUMAN was not found.
  - CSK21\_HUMAN was not found.
- cancer-cell-map-egfr1
  [+]

  No paths found.
  - IF2P\_HUMAN was not found.
  - CSK21\_HUMAN was not found.
- pid-erbb1
  [+]

  No paths found.
  - IF2P\_HUMAN was not found.
  - CSK21\_HUMAN was not found.
- science-signaling-egfr
  [+]

  No paths found.
  - IF2P\_HUMAN was not found.
  - CSK21\_HUMAN was not found.
- layek
  [+]

  No paths found.
  - IF2P\_HUMAN was not found.
  - CSK21\_HUMAN was not found.

### SMC3\_HUMAN → WAPL\_HUMAN / SMC3\_HUMAN ⊣ WAPL\_HUMAN

#### 1. Resources that have the edge


#### 2. Resources that contain endpoints (but not an edge)


#### 3. Resources that match only one endpoint (fringe)

- phosphositeplus-kinase-substrate
  [+]

  No paths found.
  - WAPL\_HUMAN was not found.

#### 4. Resources that don't match any endpoint

- kegg-mapk
  [+]

  No paths found.
  - WAPL\_HUMAN was not found.
  - SMC3\_HUMAN was not found.
- biocarta-egf
  [+]

  No paths found.
  - WAPL\_HUMAN was not found.
  - SMC3\_HUMAN was not found.
- pid-erbb1
  [+]

  No paths found.
  - WAPL\_HUMAN was not found.
  - SMC3\_HUMAN was not found.
- cancer-cell-map-egfr1
  [+]

  No paths found.
  - WAPL\_HUMAN was not found.
  - SMC3\_HUMAN was not found.
- science-signaling-egfr
  [+]

  No paths found.
  - WAPL\_HUMAN was not found.
  - SMC3\_HUMAN was not found.
- kegg-erbb
  [+]

  No paths found.
  - WAPL\_HUMAN was not found.
  - SMC3\_HUMAN was not found.
- reactome-egfr
  [+]

  No paths found.
  - WAPL\_HUMAN was not found.
  - SMC3\_HUMAN was not found.
- layek
  [+]

  No paths found.
  - WAPL\_HUMAN was not found.
  - SMC3\_HUMAN was not found.

### AKA12\_HUMAN → KAP2\_HUMAN / AKA12\_HUMAN ⊣ KAP2\_HUMAN

#### 1. Resources that have the edge


#### 2. Resources that contain endpoints (but not an edge)


#### 3. Resources that match only one endpoint (fringe)

- reactome-egfr
  [+]

  No paths found.
  - AKA12\_HUMAN was not found.

#### 4. Resources that don't match any endpoint

- science-signaling-egfr
  [+]

  No paths found.
  - AKA12\_HUMAN was not found.
  - KAP2\_HUMAN was not found.
- kegg-erbb
  [+]

  No paths found.
  - AKA12\_HUMAN was not found.
  - KAP2\_HUMAN was not found.
- layek
  [+]

  No paths found.
  - AKA12\_HUMAN was not found.
  - KAP2\_HUMAN was not found.
- phosphositeplus-kinase-substrate
  [+]

  No paths found.
  - AKA12\_HUMAN was not found.
  - KAP2\_HUMAN was not found.
- kegg-mapk
  [+]

  No paths found.
  - AKA12\_HUMAN was not found.
  - KAP2\_HUMAN was not found.
- pid-erbb1
  [+]

  No paths found.
  - AKA12\_HUMAN was not found.
  - KAP2\_HUMAN was not found.
- biocarta-egf
  [+]

  No paths found.
  - AKA12\_HUMAN was not found.
  - KAP2\_HUMAN was not found.
- cancer-cell-map-egfr1
  [+]

  No paths found.
  - AKA12\_HUMAN was not found.
  - KAP2\_HUMAN was not found.

### NUCL\_HUMAN ⊣ NUP62\_HUMAN

#### 1. Resources that have the edge


#### 2. Resources that contain endpoints (but not an edge)


#### 3. Resources that match only one endpoint (fringe)

- phosphositeplus-kinase-substrate
  [+]

  No paths found.
  - NUP62\_HUMAN was not found.

#### 4. Resources that don't match any endpoint

- science-signaling-egfr
  [+]

  No paths found.
  - NUP62\_HUMAN was not found.
  - NUCL\_HUMAN was not found.
- kegg-erbb
  [+]

  No paths found.
  - NUP62\_HUMAN was not found.
  - NUCL\_HUMAN was not found.
- cancer-cell-map-egfr1
  [+]

  No paths found.
  - NUP62\_HUMAN was not found.
  - NUCL\_HUMAN was not found.
- pid-erbb1
  [+]

  No paths found.
  - NUP62\_HUMAN was not found.
  - NUCL\_HUMAN was not found.
- biocarta-egf
  [+]

  No paths found.
  - NUP62\_HUMAN was not found.
  - NUCL\_HUMAN was not found.
- layek
  [+]

  No paths found.
  - NUP62\_HUMAN was not found.
  - NUCL\_HUMAN was not found.
- reactome-egfr
  [+]

  No paths found.
  - NUP62\_HUMAN was not found.
  - NUCL\_HUMAN was not found.
- kegg-mapk
  [+]

  No paths found.
  - NUP62\_HUMAN was not found.
  - NUCL\_HUMAN was not found.

### SMC3\_HUMAN → PDS5A\_HUMAN / SMC3\_HUMAN ⊣ PDS5A\_HUMAN

#### 1. Resources that have the edge


#### 2. Resources that contain endpoints (but not an edge)


#### 3. Resources that match only one endpoint (fringe)

- phosphositeplus-kinase-substrate
  [+]

  No paths found.
  - PDS5A\_HUMAN was not found.

#### 4. Resources that don't match any endpoint

- pid-erbb1
  [+]

  No paths found.
  - SMC3\_HUMAN was not found.
  - PDS5A\_HUMAN was not found.
- layek
  [+]

  No paths found.
  - SMC3\_HUMAN was not found.
  - PDS5A\_HUMAN was not found.
- biocarta-egf
  [+]

  No paths found.
  - SMC3\_HUMAN was not found.
  - PDS5A\_HUMAN was not found.
- kegg-erbb
  [+]

  No paths found.
  - SMC3\_HUMAN was not found.
  - PDS5A\_HUMAN was not found.
- kegg-mapk
  [+]

  No paths found.
  - SMC3\_HUMAN was not found.
  - PDS5A\_HUMAN was not found.
- science-signaling-egfr
  [+]

  No paths found.
  - SMC3\_HUMAN was not found.
  - PDS5A\_HUMAN was not found.
- reactome-egfr
  [+]

  No paths found.
  - SMC3\_HUMAN was not found.
  - PDS5A\_HUMAN was not found.
- cancer-cell-map-egfr1
  [+]

  No paths found.
  - SMC3\_HUMAN was not found.
  - PDS5A\_HUMAN was not found.

### T22D4\_HUMAN → ABLM1\_HUMAN / T22D4\_HUMAN ⊣ ABLM1\_HUMAN

#### 1. Resources that have the edge


#### 2. Resources that contain endpoints (but not an edge)


#### 3. Resources that match only one endpoint (fringe)


#### 4. Resources that don't match any endpoint

- phosphositeplus-kinase-substrate
  [+]

  No paths found.
  - ABLM1\_HUMAN was not found.
  - T22D4\_HUMAN was not found.
- science-signaling-egfr
  [+]

  No paths found.
  - ABLM1\_HUMAN was not found.
  - T22D4\_HUMAN was not found.
- biocarta-egf
  [+]

  No paths found.
  - ABLM1\_HUMAN was not found.
  - T22D4\_HUMAN was not found.
- kegg-mapk
  [+]

  No paths found.
  - ABLM1\_HUMAN was not found.
  - T22D4\_HUMAN was not found.
- kegg-erbb
  [+]

  No paths found.
  - ABLM1\_HUMAN was not found.
  - T22D4\_HUMAN was not found.
- layek
  [+]

  No paths found.
  - ABLM1\_HUMAN was not found.
  - T22D4\_HUMAN was not found.
- cancer-cell-map-egfr1
  [+]

  No paths found.
  - ABLM1\_HUMAN was not found.
  - T22D4\_HUMAN was not found.
- pid-erbb1
  [+]

  No paths found.
  - ABLM1\_HUMAN was not found.
  - T22D4\_HUMAN was not found.
- reactome-egfr
  [+]

  No paths found.
  - ABLM1\_HUMAN was not found.
  - T22D4\_HUMAN was not found.

### SUMO2\_HUMAN → POGZ\_HUMAN / SUMO2\_HUMAN ⊣ POGZ\_HUMAN

#### 1. Resources that have the edge


#### 2. Resources that contain endpoints (but not an edge)


#### 3. Resources that match only one endpoint (fringe)


#### 4. Resources that don't match any endpoint

- layek
  [+]

  No paths found.
  - POGZ\_HUMAN was not found.
  - SUMO2\_HUMAN was not found.
- science-signaling-egfr
  [+]

  No paths found.
  - POGZ\_HUMAN was not found.
  - SUMO2\_HUMAN was not found.
- biocarta-egf
  [+]

  No paths found.
  - POGZ\_HUMAN was not found.
  - SUMO2\_HUMAN was not found.
- reactome-egfr
  [+]

  No paths found.
  - POGZ\_HUMAN was not found.
  - SUMO2\_HUMAN was not found.
- kegg-erbb
  [+]

  No paths found.
  - POGZ\_HUMAN was not found.
  - SUMO2\_HUMAN was not found.
- cancer-cell-map-egfr1
  [+]

  No paths found.
  - POGZ\_HUMAN was not found.
  - SUMO2\_HUMAN was not found.
- phosphositeplus-kinase-substrate
  [+]

  No paths found.
  - POGZ\_HUMAN was not found.
  - SUMO2\_HUMAN was not found.
- kegg-mapk
  [+]

  No paths found.
  - POGZ\_HUMAN was not found.
  - SUMO2\_HUMAN was not found.
- pid-erbb1
  [+]

  No paths found.
  - POGZ\_HUMAN was not found.
  - SUMO2\_HUMAN was not found.

### DREB\_HUMAN → NIPBL\_HUMAN / DREB\_HUMAN ⊣ NIPBL\_HUMAN

#### 1. Resources that have the edge


#### 2. Resources that contain endpoints (but not an edge)


#### 3. Resources that match only one endpoint (fringe)


#### 4. Resources that don't match any endpoint

- kegg-mapk
  [+]

  No paths found.
  - NIPBL\_HUMAN was not found.
  - DREB\_HUMAN was not found.
- pid-erbb1
  [+]

  No paths found.
  - NIPBL\_HUMAN was not found.
  - DREB\_HUMAN was not found.
- layek
  [+]

  No paths found.
  - NIPBL\_HUMAN was not found.
  - DREB\_HUMAN was not found.
- kegg-erbb
  [+]

  No paths found.
  - NIPBL\_HUMAN was not found.
  - DREB\_HUMAN was not found.
- science-signaling-egfr
  [+]

  No paths found.
  - NIPBL\_HUMAN was not found.
  - DREB\_HUMAN was not found.
- reactome-egfr
  [+]

  No paths found.
  - NIPBL\_HUMAN was not found.
  - DREB\_HUMAN was not found.
- biocarta-egf
  [+]

  No paths found.
  - NIPBL\_HUMAN was not found.
  - DREB\_HUMAN was not found.
- phosphositeplus-kinase-substrate
  [+]

  No paths found.
  - NIPBL\_HUMAN was not found.
  - DREB\_HUMAN was not found.
- cancer-cell-map-egfr1
  [+]

  No paths found.
  - NIPBL\_HUMAN was not found.
  - DREB\_HUMAN was not found.

### TM1L1\_HUMAN → F208A\_HUMAN / TM1L1\_HUMAN ⊣ F208A\_HUMAN

#### 1. Resources that have the edge


#### 2. Resources that contain endpoints (but not an edge)


#### 3. Resources that match only one endpoint (fringe)


#### 4. Resources that don't match any endpoint

- science-signaling-egfr
  [+]

  No paths found.
  - TM1L1\_HUMAN was not found.
  - F208A\_HUMAN was not found.
- phosphositeplus-kinase-substrate
  [+]

  No paths found.
  - TM1L1\_HUMAN was not found.
  - F208A\_HUMAN was not found.
- kegg-erbb
  [+]

  No paths found.
  - TM1L1\_HUMAN was not found.
  - F208A\_HUMAN was not found.
- pid-erbb1
  [+]

  No paths found.
  - TM1L1\_HUMAN was not found.
  - F208A\_HUMAN was not found.
- biocarta-egf
  [+]

  No paths found.
  - TM1L1\_HUMAN was not found.
  - F208A\_HUMAN was not found.
- reactome-egfr
  [+]

  No paths found.
  - TM1L1\_HUMAN was not found.
  - F208A\_HUMAN was not found.
- layek
  [+]

  No paths found.
  - TM1L1\_HUMAN was not found.
  - F208A\_HUMAN was not found.
- cancer-cell-map-egfr1
  [+]

  No paths found.
  - TM1L1\_HUMAN was not found.
  - F208A\_HUMAN was not found.
- kegg-mapk
  [+]

  No paths found.
  - TM1L1\_HUMAN was not found.
  - F208A\_HUMAN was not found.

### PNKP\_HUMAN → LAS1L\_HUMAN / PNKP\_HUMAN ⊣ LAS1L\_HUMAN

#### 1. Resources that have the edge


#### 2. Resources that contain endpoints (but not an edge)


#### 3. Resources that match only one endpoint (fringe)


#### 4. Resources that don't match any endpoint

- pid-erbb1
  [+]

  No paths found.
  - LAS1L\_HUMAN was not found.
  - PNKP\_HUMAN was not found.
- kegg-mapk
  [+]

  No paths found.
  - LAS1L\_HUMAN was not found.
  - PNKP\_HUMAN was not found.
- biocarta-egf
  [+]

  No paths found.
  - LAS1L\_HUMAN was not found.
  - PNKP\_HUMAN was not found.
- cancer-cell-map-egfr1
  [+]

  No paths found.
  - LAS1L\_HUMAN was not found.
  - PNKP\_HUMAN was not found.
- science-signaling-egfr
  [+]

  No paths found.
  - LAS1L\_HUMAN was not found.
  - PNKP\_HUMAN was not found.
- phosphositeplus-kinase-substrate
  [+]

  No paths found.
  - LAS1L\_HUMAN was not found.
  - PNKP\_HUMAN was not found.
- reactome-egfr
  [+]

  No paths found.
  - LAS1L\_HUMAN was not found.
  - PNKP\_HUMAN was not found.
- layek
  [+]

  No paths found.
  - LAS1L\_HUMAN was not found.
  - PNKP\_HUMAN was not found.
- kegg-erbb
  [+]

  No paths found.
  - LAS1L\_HUMAN was not found.
  - PNKP\_HUMAN was not found.

### SUMO2\_HUMAN → ZKSC1\_HUMAN / SUMO2\_HUMAN ⊣ ZKSC1\_HUMAN

#### 1. Resources that have the edge


#### 2. Resources that contain endpoints (but not an edge)


#### 3. Resources that match only one endpoint (fringe)


#### 4. Resources that don't match any endpoint

- biocarta-egf
  [+]

  No paths found.
  - ZKSC1\_HUMAN was not found.
  - SUMO2\_HUMAN was not found.
- cancer-cell-map-egfr1
  [+]

  No paths found.
  - ZKSC1\_HUMAN was not found.
  - SUMO2\_HUMAN was not found.
- phosphositeplus-kinase-substrate
  [+]

  No paths found.
  - ZKSC1\_HUMAN was not found.
  - SUMO2\_HUMAN was not found.
- kegg-erbb
  [+]

  No paths found.
  - ZKSC1\_HUMAN was not found.
  - SUMO2\_HUMAN was not found.
- layek
  [+]

  No paths found.
  - ZKSC1\_HUMAN was not found.
  - SUMO2\_HUMAN was not found.
- pid-erbb1
  [+]

  No paths found.
  - ZKSC1\_HUMAN was not found.
  - SUMO2\_HUMAN was not found.
- science-signaling-egfr
  [+]

  No paths found.
  - ZKSC1\_HUMAN was not found.
  - SUMO2\_HUMAN was not found.
- reactome-egfr
  [+]

  No paths found.
  - ZKSC1\_HUMAN was not found.
  - SUMO2\_HUMAN was not found.
- kegg-mapk
  [+]

  No paths found.
  - ZKSC1\_HUMAN was not found.
  - SUMO2\_HUMAN was not found.

### PP1B\_HUMAN ⊣ DENR\_HUMAN

#### 1. Resources that have the edge


#### 2. Resources that contain endpoints (but not an edge)


#### 3. Resources that match only one endpoint (fringe)


#### 4. Resources that don't match any endpoint

- science-signaling-egfr
  [+]

  No paths found.
  - DENR\_HUMAN was not found.
  - PP1B\_HUMAN was not found.
- phosphositeplus-kinase-substrate
  [+]

  No paths found.
  - DENR\_HUMAN was not found.
  - PP1B\_HUMAN was not found.
- layek
  [+]

  No paths found.
  - DENR\_HUMAN was not found.
  - PP1B\_HUMAN was not found.
- pid-erbb1
  [+]

  No paths found.
  - DENR\_HUMAN was not found.
  - PP1B\_HUMAN was not found.
- reactome-egfr
  [+]

  No paths found.
  - DENR\_HUMAN was not found.
  - PP1B\_HUMAN was not found.
- cancer-cell-map-egfr1
  [+]

  No paths found.
  - DENR\_HUMAN was not found.
  - PP1B\_HUMAN was not found.
- biocarta-egf
  [+]

  No paths found.
  - DENR\_HUMAN was not found.
  - PP1B\_HUMAN was not found.
- kegg-mapk
  [+]

  No paths found.
  - DENR\_HUMAN was not found.
  - PP1B\_HUMAN was not found.
- kegg-erbb
  [+]

  No paths found.
  - DENR\_HUMAN was not found.
  - PP1B\_HUMAN was not found.

### MCRS1\_HUMAN → ITSN2\_HUMAN / MCRS1\_HUMAN ⊣ ITSN2\_HUMAN

#### 1. Resources that have the edge


#### 2. Resources that contain endpoints (but not an edge)


#### 3. Resources that match only one endpoint (fringe)


#### 4. Resources that don't match any endpoint

- science-signaling-egfr
  [+]

  No paths found.
  - ITSN2\_HUMAN was not found.
  - MCRS1\_HUMAN was not found.
- phosphositeplus-kinase-substrate
  [+]

  No paths found.
  - ITSN2\_HUMAN was not found.
  - MCRS1\_HUMAN was not found.
- pid-erbb1
  [+]

  No paths found.
  - ITSN2\_HUMAN was not found.
  - MCRS1\_HUMAN was not found.
- reactome-egfr
  [+]

  No paths found.
  - ITSN2\_HUMAN was not found.
  - MCRS1\_HUMAN was not found.
- kegg-erbb
  [+]

  No paths found.
  - ITSN2\_HUMAN was not found.
  - MCRS1\_HUMAN was not found.
- layek
  [+]

  No paths found.
  - ITSN2\_HUMAN was not found.
  - MCRS1\_HUMAN was not found.
- kegg-mapk
  [+]

  No paths found.
  - ITSN2\_HUMAN was not found.
  - MCRS1\_HUMAN was not found.
- cancer-cell-map-egfr1
  [+]

  No paths found.
  - ITSN2\_HUMAN was not found.
  - MCRS1\_HUMAN was not found.
- biocarta-egf
  [+]

  No paths found.
  - ITSN2\_HUMAN was not found.
  - MCRS1\_HUMAN was not found.

### SUMO2\_HUMAN → DPP3\_HUMAN / SUMO2\_HUMAN ⊣ DPP3\_HUMAN

#### 1. Resources that have the edge


#### 2. Resources that contain endpoints (but not an edge)


#### 3. Resources that match only one endpoint (fringe)


#### 4. Resources that don't match any endpoint

- pid-erbb1
  [+]

  No paths found.
  - SUMO2\_HUMAN was not found.
  - DPP3\_HUMAN was not found.
- kegg-mapk
  [+]

  No paths found.
  - SUMO2\_HUMAN was not found.
  - DPP3\_HUMAN was not found.
- phosphositeplus-kinase-substrate
  [+]

  No paths found.
  - SUMO2\_HUMAN was not found.
  - DPP3\_HUMAN was not found.
- layek
  [+]

  No paths found.
  - SUMO2\_HUMAN was not found.
  - DPP3\_HUMAN was not found.
- reactome-egfr
  [+]

  No paths found.
  - SUMO2\_HUMAN was not found.
  - DPP3\_HUMAN was not found.
- kegg-erbb
  [+]

  No paths found.
  - SUMO2\_HUMAN was not found.
  - DPP3\_HUMAN was not found.
- biocarta-egf
  [+]

  No paths found.
  - SUMO2\_HUMAN was not found.
  - DPP3\_HUMAN was not found.
- cancer-cell-map-egfr1
  [+]

  No paths found.
  - SUMO2\_HUMAN was not found.
  - DPP3\_HUMAN was not found.
- science-signaling-egfr
  [+]

  No paths found.
  - SUMO2\_HUMAN was not found.
  - DPP3\_HUMAN was not found.

### SCG1\_HUMAN → SG223\_HUMAN / SCG1\_HUMAN ⊣ SG223\_HUMAN

#### 1. Resources that have the edge


#### 2. Resources that contain endpoints (but not an edge)


#### 3. Resources that match only one endpoint (fringe)


#### 4. Resources that don't match any endpoint

- cancer-cell-map-egfr1
  [+]

  No paths found.
  - SG223\_HUMAN was not found.
  - SCG1\_HUMAN was not found.
- biocarta-egf
  [+]

  No paths found.
  - SG223\_HUMAN was not found.
  - SCG1\_HUMAN was not found.
- pid-erbb1
  [+]

  No paths found.
  - SG223\_HUMAN was not found.
  - SCG1\_HUMAN was not found.
- science-signaling-egfr
  [+]

  No paths found.
  - SG223\_HUMAN was not found.
  - SCG1\_HUMAN was not found.
- phosphositeplus-kinase-substrate
  [+]

  No paths found.
  - SG223\_HUMAN was not found.
  - SCG1\_HUMAN was not found.
- reactome-egfr
  [+]

  No paths found.
  - SG223\_HUMAN was not found.
  - SCG1\_HUMAN was not found.
- kegg-mapk
  [+]

  No paths found.
  - SG223\_HUMAN was not found.
  - SCG1\_HUMAN was not found.
- kegg-erbb
  [+]

  No paths found.
  - SG223\_HUMAN was not found.
  - SCG1\_HUMAN was not found.
- layek
  [+]

  No paths found.
  - SG223\_HUMAN was not found.
  - SCG1\_HUMAN was not found.

### EFNA5\_HUMAN → EPHA7\_HUMAN / EFNA5\_HUMAN ⊣ EPHA7\_HUMAN

#### 1. Resources that have the edge


#### 2. Resources that contain endpoints (but not an edge)


#### 3. Resources that match only one endpoint (fringe)


#### 4. Resources that don't match any endpoint

- kegg-erbb
  [+]

  No paths found.
  - EPHA7\_HUMAN was not found.
  - EFNA5\_HUMAN was not found.
- cancer-cell-map-egfr1
  [+]

  No paths found.
  - EPHA7\_HUMAN was not found.
  - EFNA5\_HUMAN was not found.
- layek
  [+]

  No paths found.
  - EPHA7\_HUMAN was not found.
  - EFNA5\_HUMAN was not found.
- pid-erbb1
  [+]

  No paths found.
  - EPHA7\_HUMAN was not found.
  - EFNA5\_HUMAN was not found.
- kegg-mapk
  [+]

  No paths found.
  - EPHA7\_HUMAN was not found.
  - EFNA5\_HUMAN was not found.
- phosphositeplus-kinase-substrate
  [+]

  No paths found.
  - EPHA7\_HUMAN was not found.
  - EFNA5\_HUMAN was not found.
- science-signaling-egfr
  [+]

  No paths found.
  - EPHA7\_HUMAN was not found.
  - EFNA5\_HUMAN was not found.
- biocarta-egf
  [+]

  No paths found.
  - EPHA7\_HUMAN was not found.
  - EFNA5\_HUMAN was not found.
- reactome-egfr
  [+]

  No paths found.
  - EPHA7\_HUMAN was not found.
  - EFNA5\_HUMAN was not found.

### SUMO2\_HUMAN → RBM15\_HUMAN / SUMO2\_HUMAN ⊣ RBM15\_HUMAN

#### 1. Resources that have the edge


#### 2. Resources that contain endpoints (but not an edge)


#### 3. Resources that match only one endpoint (fringe)


#### 4. Resources that don't match any endpoint

- reactome-egfr
  [+]

  No paths found.
  - RBM15\_HUMAN was not found.
  - SUMO2\_HUMAN was not found.
- kegg-erbb
  [+]

  No paths found.
  - RBM15\_HUMAN was not found.
  - SUMO2\_HUMAN was not found.
- biocarta-egf
  [+]

  No paths found.
  - RBM15\_HUMAN was not found.
  - SUMO2\_HUMAN was not found.
- kegg-mapk
  [+]

  No paths found.
  - RBM15\_HUMAN was not found.
  - SUMO2\_HUMAN was not found.
- science-signaling-egfr
  [+]

  No paths found.
  - RBM15\_HUMAN was not found.
  - SUMO2\_HUMAN was not found.
- pid-erbb1
  [+]

  No paths found.
  - RBM15\_HUMAN was not found.
  - SUMO2\_HUMAN was not found.
- phosphositeplus-kinase-substrate
  [+]

  No paths found.
  - RBM15\_HUMAN was not found.
  - SUMO2\_HUMAN was not found.
- cancer-cell-map-egfr1
  [+]

  No paths found.
  - RBM15\_HUMAN was not found.
  - SUMO2\_HUMAN was not found.
- layek
  [+]

  No paths found.
  - RBM15\_HUMAN was not found.
  - SUMO2\_HUMAN was not found.

### T22D4\_HUMAN → CCNK\_HUMAN / T22D4\_HUMAN ⊣ CCNK\_HUMAN

#### 1. Resources that have the edge


#### 2. Resources that contain endpoints (but not an edge)


#### 3. Resources that match only one endpoint (fringe)


#### 4. Resources that don't match any endpoint

- layek
  [+]

  No paths found.
  - CCNK\_HUMAN was not found.
  - T22D4\_HUMAN was not found.
- pid-erbb1
  [+]

  No paths found.
  - CCNK\_HUMAN was not found.
  - T22D4\_HUMAN was not found.
- reactome-egfr
  [+]

  No paths found.
  - CCNK\_HUMAN was not found.
  - T22D4\_HUMAN was not found.
- phosphositeplus-kinase-substrate
  [+]

  No paths found.
  - CCNK\_HUMAN was not found.
  - T22D4\_HUMAN was not found.
- science-signaling-egfr
  [+]

  No paths found.
  - CCNK\_HUMAN was not found.
  - T22D4\_HUMAN was not found.
- kegg-erbb
  [+]

  No paths found.
  - CCNK\_HUMAN was not found.
  - T22D4\_HUMAN was not found.
- kegg-mapk
  [+]

  No paths found.
  - CCNK\_HUMAN was not found.
  - T22D4\_HUMAN was not found.
- cancer-cell-map-egfr1
  [+]

  No paths found.
  - CCNK\_HUMAN was not found.
  - T22D4\_HUMAN was not found.
- biocarta-egf
  [+]

  No paths found.
  - CCNK\_HUMAN was not found.
  - T22D4\_HUMAN was not found.

### WDR48\_HUMAN ⊣ PSME3\_HUMAN

#### 1. Resources that have the edge


#### 2. Resources that contain endpoints (but not an edge)


#### 3. Resources that match only one endpoint (fringe)


#### 4. Resources that don't match any endpoint

- kegg-mapk
  [+]

  No paths found.
  - PSME3\_HUMAN was not found.
  - WDR48\_HUMAN was not found.
- cancer-cell-map-egfr1
  [+]

  No paths found.
  - PSME3\_HUMAN was not found.
  - WDR48\_HUMAN was not found.
- biocarta-egf
  [+]

  No paths found.
  - PSME3\_HUMAN was not found.
  - WDR48\_HUMAN was not found.
- phosphositeplus-kinase-substrate
  [+]

  No paths found.
  - PSME3\_HUMAN was not found.
  - WDR48\_HUMAN was not found.
- reactome-egfr
  [+]

  No paths found.
  - PSME3\_HUMAN was not found.
  - WDR48\_HUMAN was not found.
- pid-erbb1
  [+]

  No paths found.
  - PSME3\_HUMAN was not found.
  - WDR48\_HUMAN was not found.
- kegg-erbb
  [+]

  No paths found.
  - PSME3\_HUMAN was not found.
  - WDR48\_HUMAN was not found.
- science-signaling-egfr
  [+]

  No paths found.
  - PSME3\_HUMAN was not found.
  - WDR48\_HUMAN was not found.
- layek
  [+]

  No paths found.
  - PSME3\_HUMAN was not found.
  - WDR48\_HUMAN was not found.

### T22D4\_HUMAN → NRBP\_HUMAN / T22D4\_HUMAN ⊣ NRBP\_HUMAN

#### 1. Resources that have the edge


#### 2. Resources that contain endpoints (but not an edge)


#### 3. Resources that match only one endpoint (fringe)


#### 4. Resources that don't match any endpoint

- science-signaling-egfr
  [+]

  No paths found.
  - NRBP\_HUMAN was not found.
  - T22D4\_HUMAN was not found.
- reactome-egfr
  [+]

  No paths found.
  - NRBP\_HUMAN was not found.
  - T22D4\_HUMAN was not found.
- kegg-mapk
  [+]

  No paths found.
  - NRBP\_HUMAN was not found.
  - T22D4\_HUMAN was not found.
- phosphositeplus-kinase-substrate
  [+]

  No paths found.
  - NRBP\_HUMAN was not found.
  - T22D4\_HUMAN was not found.
- cancer-cell-map-egfr1
  [+]

  No paths found.
  - NRBP\_HUMAN was not found.
  - T22D4\_HUMAN was not found.
- biocarta-egf
  [+]

  No paths found.
  - NRBP\_HUMAN was not found.
  - T22D4\_HUMAN was not found.
- pid-erbb1
  [+]

  No paths found.
  - NRBP\_HUMAN was not found.
  - T22D4\_HUMAN was not found.
- kegg-erbb
  [+]

  No paths found.
  - NRBP\_HUMAN was not found.
  - T22D4\_HUMAN was not found.
- layek
  [+]

  No paths found.
  - NRBP\_HUMAN was not found.
  - T22D4\_HUMAN was not found.

### DPP3\_HUMAN → NHLC2\_HUMAN / DPP3\_HUMAN ⊣ NHLC2\_HUMAN

#### 1. Resources that have the edge


#### 2. Resources that contain endpoints (but not an edge)


#### 3. Resources that match only one endpoint (fringe)


#### 4. Resources that don't match any endpoint

- phosphositeplus-kinase-substrate
  [+]

  No paths found.
  - NHLC2\_HUMAN was not found.
  - DPP3\_HUMAN was not found.
- kegg-erbb
  [+]

  No paths found.
  - NHLC2\_HUMAN was not found.
  - DPP3\_HUMAN was not found.
- science-signaling-egfr
  [+]

  No paths found.
  - NHLC2\_HUMAN was not found.
  - DPP3\_HUMAN was not found.
- biocarta-egf
  [+]

  No paths found.
  - NHLC2\_HUMAN was not found.
  - DPP3\_HUMAN was not found.
- layek
  [+]

  No paths found.
  - NHLC2\_HUMAN was not found.
  - DPP3\_HUMAN was not found.
- reactome-egfr
  [+]

  No paths found.
  - NHLC2\_HUMAN was not found.
  - DPP3\_HUMAN was not found.
- kegg-mapk
  [+]

  No paths found.
  - NHLC2\_HUMAN was not found.
  - DPP3\_HUMAN was not found.
- cancer-cell-map-egfr1
  [+]

  No paths found.
  - NHLC2\_HUMAN was not found.
  - DPP3\_HUMAN was not found.
- pid-erbb1
  [+]

  No paths found.
  - NHLC2\_HUMAN was not found.
  - DPP3\_HUMAN was not found.

### SUMO2\_HUMAN → ZMYM4\_HUMAN / SUMO2\_HUMAN ⊣ ZMYM4\_HUMAN

#### 1. Resources that have the edge


#### 2. Resources that contain endpoints (but not an edge)


#### 3. Resources that match only one endpoint (fringe)


#### 4. Resources that don't match any endpoint

- kegg-erbb
  [+]

  No paths found.
  - ZMYM4\_HUMAN was not found.
  - SUMO2\_HUMAN was not found.
- biocarta-egf
  [+]

  No paths found.
  - ZMYM4\_HUMAN was not found.
  - SUMO2\_HUMAN was not found.
- science-signaling-egfr
  [+]

  No paths found.
  - ZMYM4\_HUMAN was not found.
  - SUMO2\_HUMAN was not found.
- layek
  [+]

  No paths found.
  - ZMYM4\_HUMAN was not found.
  - SUMO2\_HUMAN was not found.
- reactome-egfr
  [+]

  No paths found.
  - ZMYM4\_HUMAN was not found.
  - SUMO2\_HUMAN was not found.
- cancer-cell-map-egfr1
  [+]

  No paths found.
  - ZMYM4\_HUMAN was not found.
  - SUMO2\_HUMAN was not found.
- kegg-mapk
  [+]

  No paths found.
  - ZMYM4\_HUMAN was not found.
  - SUMO2\_HUMAN was not found.
- pid-erbb1
  [+]

  No paths found.
  - ZMYM4\_HUMAN was not found.
  - SUMO2\_HUMAN was not found.
- phosphositeplus-kinase-substrate
  [+]

  No paths found.
  - ZMYM4\_HUMAN was not found.
  - SUMO2\_HUMAN was not found.

### SPB1\_HUMAN ⊣ WDHD1\_HUMAN

#### 1. Resources that have the edge


#### 2. Resources that contain endpoints (but not an edge)


#### 3. Resources that match only one endpoint (fringe)


#### 4. Resources that don't match any endpoint

- layek
  [+]

  No paths found.
  - WDHD1\_HUMAN was not found.
  - SPB1\_HUMAN was not found.
- kegg-erbb
  [+]

  No paths found.
  - WDHD1\_HUMAN was not found.
  - SPB1\_HUMAN was not found.
- biocarta-egf
  [+]

  No paths found.
  - WDHD1\_HUMAN was not found.
  - SPB1\_HUMAN was not found.
- reactome-egfr
  [+]

  No paths found.
  - WDHD1\_HUMAN was not found.
  - SPB1\_HUMAN was not found.
- kegg-mapk
  [+]

  No paths found.
  - WDHD1\_HUMAN was not found.
  - SPB1\_HUMAN was not found.
- cancer-cell-map-egfr1
  [+]

  No paths found.
  - WDHD1\_HUMAN was not found.
  - SPB1\_HUMAN was not found.
- pid-erbb1
  [+]

  No paths found.
  - WDHD1\_HUMAN was not found.
  - SPB1\_HUMAN was not found.
- science-signaling-egfr
  [+]

  No paths found.
  - WDHD1\_HUMAN was not found.
  - SPB1\_HUMAN was not found.
- phosphositeplus-kinase-substrate
  [+]

  No paths found.
  - WDHD1\_HUMAN was not found.
  - SPB1\_HUMAN was not found.

### RNF31\_HUMAN → OTUL\_HUMAN / RNF31\_HUMAN ⊣ OTUL\_HUMAN

#### 1. Resources that have the edge


#### 2. Resources that contain endpoints (but not an edge)


#### 3. Resources that match only one endpoint (fringe)


#### 4. Resources that don't match any endpoint

- kegg-erbb
  [+]

  No paths found.
  - RNF31\_HUMAN was not found.
  - OTUL\_HUMAN was not found.
- reactome-egfr
  [+]

  No paths found.
  - RNF31\_HUMAN was not found.
  - OTUL\_HUMAN was not found.
- pid-erbb1
  [+]

  No paths found.
  - RNF31\_HUMAN was not found.
  - OTUL\_HUMAN was not found.
- biocarta-egf
  [+]

  No paths found.
  - RNF31\_HUMAN was not found.
  - OTUL\_HUMAN was not found.
- science-signaling-egfr
  [+]

  No paths found.
  - RNF31\_HUMAN was not found.
  - OTUL\_HUMAN was not found.
- layek
  [+]

  No paths found.
  - RNF31\_HUMAN was not found.
  - OTUL\_HUMAN was not found.
- kegg-mapk
  [+]

  No paths found.
  - RNF31\_HUMAN was not found.
  - OTUL\_HUMAN was not found.
- cancer-cell-map-egfr1
  [+]

  No paths found.
  - RNF31\_HUMAN was not found.
  - OTUL\_HUMAN was not found.
- phosphositeplus-kinase-substrate
  [+]

  No paths found.
  - RNF31\_HUMAN was not found.
  - OTUL\_HUMAN was not found.

### EPHA3\_HUMAN → EFNB2\_HUMAN

#### 1. Resources that have the edge


#### 2. Resources that contain endpoints (but not an edge)


#### 3. Resources that match only one endpoint (fringe)


#### 4. Resources that don't match any endpoint

- pid-erbb1
  [+]

  No paths found.
  - EFNB2\_HUMAN was not found.
  - EPHA3\_HUMAN was not found.
- science-signaling-egfr
  [+]

  No paths found.
  - EFNB2\_HUMAN was not found.
  - EPHA3\_HUMAN was not found.
- phosphositeplus-kinase-substrate
  [+]

  No paths found.
  - EFNB2\_HUMAN was not found.
  - EPHA3\_HUMAN was not found.
- kegg-erbb
  [+]

  No paths found.
  - EFNB2\_HUMAN was not found.
  - EPHA3\_HUMAN was not found.
- kegg-mapk
  [+]

  No paths found.
  - EFNB2\_HUMAN was not found.
  - EPHA3\_HUMAN was not found.
- biocarta-egf
  [+]

  No paths found.
  - EFNB2\_HUMAN was not found.
  - EPHA3\_HUMAN was not found.
- reactome-egfr
  [+]

  No paths found.
  - EFNB2\_HUMAN was not found.
  - EPHA3\_HUMAN was not found.
- layek
  [+]

  No paths found.
  - EFNB2\_HUMAN was not found.
  - EPHA3\_HUMAN was not found.
- cancer-cell-map-egfr1
  [+]

  No paths found.
  - EFNB2\_HUMAN was not found.
  - EPHA3\_HUMAN was not found.

### SUMO2\_HUMAN → CHAP1\_HUMAN / SUMO2\_HUMAN ⊣ CHAP1\_HUMAN

#### 1. Resources that have the edge


#### 2. Resources that contain endpoints (but not an edge)


#### 3. Resources that match only one endpoint (fringe)


#### 4. Resources that don't match any endpoint

- pid-erbb1
  [+]

  No paths found.
  - CHAP1\_HUMAN was not found.
  - SUMO2\_HUMAN was not found.
- kegg-erbb
  [+]

  No paths found.
  - CHAP1\_HUMAN was not found.
  - SUMO2\_HUMAN was not found.
- phosphositeplus-kinase-substrate
  [+]

  No paths found.
  - CHAP1\_HUMAN was not found.
  - SUMO2\_HUMAN was not found.
- reactome-egfr
  [+]

  No paths found.
  - CHAP1\_HUMAN was not found.
  - SUMO2\_HUMAN was not found.
- kegg-mapk
  [+]

  No paths found.
  - CHAP1\_HUMAN was not found.
  - SUMO2\_HUMAN was not found.
- cancer-cell-map-egfr1
  [+]

  No paths found.
  - CHAP1\_HUMAN was not found.
  - SUMO2\_HUMAN was not found.
- layek
  [+]

  No paths found.
  - CHAP1\_HUMAN was not found.
  - SUMO2\_HUMAN was not found.
- biocarta-egf
  [+]

  No paths found.
  - CHAP1\_HUMAN was not found.
  - SUMO2\_HUMAN was not found.
- science-signaling-egfr
  [+]

  No paths found.
  - CHAP1\_HUMAN was not found.
  - SUMO2\_HUMAN was not found.

### SUMO2\_HUMAN → SENP3\_HUMAN / SUMO2\_HUMAN ⊣ SENP3\_HUMAN

#### 1. Resources that have the edge


#### 2. Resources that contain endpoints (but not an edge)


#### 3. Resources that match only one endpoint (fringe)


#### 4. Resources that don't match any endpoint

- science-signaling-egfr
  [+]

  No paths found.
  - SENP3\_HUMAN was not found.
  - SUMO2\_HUMAN was not found.
- kegg-erbb
  [+]

  No paths found.
  - SENP3\_HUMAN was not found.
  - SUMO2\_HUMAN was not found.
- biocarta-egf
  [+]

  No paths found.
  - SENP3\_HUMAN was not found.
  - SUMO2\_HUMAN was not found.
- pid-erbb1
  [+]

  No paths found.
  - SENP3\_HUMAN was not found.
  - SUMO2\_HUMAN was not found.
- phosphositeplus-kinase-substrate
  [+]

  No paths found.
  - SENP3\_HUMAN was not found.
  - SUMO2\_HUMAN was not found.
- kegg-mapk
  [+]

  No paths found.
  - SENP3\_HUMAN was not found.
  - SUMO2\_HUMAN was not found.
- cancer-cell-map-egfr1
  [+]

  No paths found.
  - SENP3\_HUMAN was not found.
  - SUMO2\_HUMAN was not found.
- reactome-egfr
  [+]

  No paths found.
  - SENP3\_HUMAN was not found.
  - SUMO2\_HUMAN was not found.
- layek
  [+]

  No paths found.
  - SENP3\_HUMAN was not found.
  - SUMO2\_HUMAN was not found.

### PPIG\_HUMAN → NKAP\_HUMAN / PPIG\_HUMAN ⊣ NKAP\_HUMAN

#### 1. Resources that have the edge


#### 2. Resources that contain endpoints (but not an edge)


#### 3. Resources that match only one endpoint (fringe)


#### 4. Resources that don't match any endpoint

- layek
  [+]

  No paths found.
  - NKAP\_HUMAN was not found.
  - PPIG\_HUMAN was not found.
- biocarta-egf
  [+]

  No paths found.
  - NKAP\_HUMAN was not found.
  - PPIG\_HUMAN was not found.
- pid-erbb1
  [+]

  No paths found.
  - NKAP\_HUMAN was not found.
  - PPIG\_HUMAN was not found.
- kegg-erbb
  [+]

  No paths found.
  - NKAP\_HUMAN was not found.
  - PPIG\_HUMAN was not found.
- cancer-cell-map-egfr1
  [+]

  No paths found.
  - NKAP\_HUMAN was not found.
  - PPIG\_HUMAN was not found.
- phosphositeplus-kinase-substrate
  [+]

  No paths found.
  - NKAP\_HUMAN was not found.
  - PPIG\_HUMAN was not found.
- kegg-mapk
  [+]

  No paths found.
  - NKAP\_HUMAN was not found.
  - PPIG\_HUMAN was not found.
- reactome-egfr
  [+]

  No paths found.
  - NKAP\_HUMAN was not found.
  - PPIG\_HUMAN was not found.
- science-signaling-egfr
  [+]

  No paths found.
  - NKAP\_HUMAN was not found.
  - PPIG\_HUMAN was not found.

### ABI2\_HUMAN → MIPT3\_HUMAN / ABI2\_HUMAN ⊣ MIPT3\_HUMAN

#### 1. Resources that have the edge


#### 2. Resources that contain endpoints (but not an edge)


#### 3. Resources that match only one endpoint (fringe)


#### 4. Resources that don't match any endpoint

- reactome-egfr
  [+]

  No paths found.
  - ABI2\_HUMAN was not found.
  - MIPT3\_HUMAN was not found.
- layek
  [+]

  No paths found.
  - ABI2\_HUMAN was not found.
  - MIPT3\_HUMAN was not found.
- phosphositeplus-kinase-substrate
  [+]

  No paths found.
  - ABI2\_HUMAN was not found.
  - MIPT3\_HUMAN was not found.
- kegg-mapk
  [+]

  No paths found.
  - ABI2\_HUMAN was not found.
  - MIPT3\_HUMAN was not found.
- cancer-cell-map-egfr1
  [+]

  No paths found.
  - ABI2\_HUMAN was not found.
  - MIPT3\_HUMAN was not found.
- pid-erbb1
  [+]

  No paths found.
  - ABI2\_HUMAN was not found.
  - MIPT3\_HUMAN was not found.
- science-signaling-egfr
  [+]

  No paths found.
  - ABI2\_HUMAN was not found.
  - MIPT3\_HUMAN was not found.
- biocarta-egf
  [+]

  No paths found.
  - ABI2\_HUMAN was not found.
  - MIPT3\_HUMAN was not found.
- kegg-erbb
  [+]

  No paths found.
  - ABI2\_HUMAN was not found.
  - MIPT3\_HUMAN was not found.

### RNF31\_HUMAN → HOIL1\_HUMAN / RNF31\_HUMAN ⊣ HOIL1\_HUMAN

#### 1. Resources that have the edge


#### 2. Resources that contain endpoints (but not an edge)


#### 3. Resources that match only one endpoint (fringe)


#### 4. Resources that don't match any endpoint

- reactome-egfr
  [+]

  No paths found.
  - RNF31\_HUMAN was not found.
  - HOIL1\_HUMAN was not found.
- kegg-erbb
  [+]

  No paths found.
  - RNF31\_HUMAN was not found.
  - HOIL1\_HUMAN was not found.
- biocarta-egf
  [+]

  No paths found.
  - RNF31\_HUMAN was not found.
  - HOIL1\_HUMAN was not found.
- kegg-mapk
  [+]

  No paths found.
  - RNF31\_HUMAN was not found.
  - HOIL1\_HUMAN was not found.
- cancer-cell-map-egfr1
  [+]

  No paths found.
  - RNF31\_HUMAN was not found.
  - HOIL1\_HUMAN was not found.
- layek
  [+]

  No paths found.
  - RNF31\_HUMAN was not found.
  - HOIL1\_HUMAN was not found.
- pid-erbb1
  [+]

  No paths found.
  - RNF31\_HUMAN was not found.
  - HOIL1\_HUMAN was not found.
- phosphositeplus-kinase-substrate
  [+]

  No paths found.
  - RNF31\_HUMAN was not found.
  - HOIL1\_HUMAN was not found.
- science-signaling-egfr
  [+]

  No paths found.
  - RNF31\_HUMAN was not found.
  - HOIL1\_HUMAN was not found.

### ARL6\_HUMAN → AR6P6\_HUMAN / ARL6\_HUMAN ⊣ AR6P6\_HUMAN

#### 1. Resources that have the edge


#### 2. Resources that contain endpoints (but not an edge)


#### 3. Resources that match only one endpoint (fringe)


#### 4. Resources that don't match any endpoint

- science-signaling-egfr
  [+]

  No paths found.
  - ARL6\_HUMAN was not found.
  - AR6P6\_HUMAN was not found.
- reactome-egfr
  [+]

  No paths found.
  - ARL6\_HUMAN was not found.
  - AR6P6\_HUMAN was not found.
- pid-erbb1
  [+]

  No paths found.
  - ARL6\_HUMAN was not found.
  - AR6P6\_HUMAN was not found.
- layek
  [+]

  No paths found.
  - ARL6\_HUMAN was not found.
  - AR6P6\_HUMAN was not found.
- biocarta-egf
  [+]

  No paths found.
  - ARL6\_HUMAN was not found.
  - AR6P6\_HUMAN was not found.
- phosphositeplus-kinase-substrate
  [+]

  No paths found.
  - ARL6\_HUMAN was not found.
  - AR6P6\_HUMAN was not found.
- kegg-mapk
  [+]

  No paths found.
  - ARL6\_HUMAN was not found.
  - AR6P6\_HUMAN was not found.
- cancer-cell-map-egfr1
  [+]

  No paths found.
  - ARL6\_HUMAN was not found.
  - AR6P6\_HUMAN was not found.
- kegg-erbb
  [+]

  No paths found.
  - ARL6\_HUMAN was not found.
  - AR6P6\_HUMAN was not found.

### ICLN\_HUMAN → PDLI1\_HUMAN / ICLN\_HUMAN ⊣ PDLI1\_HUMAN

#### 1. Resources that have the edge


#### 2. Resources that contain endpoints (but not an edge)


#### 3. Resources that match only one endpoint (fringe)


#### 4. Resources that don't match any endpoint

- kegg-erbb
  [+]

  No paths found.
  - PDLI1\_HUMAN was not found.
  - ICLN\_HUMAN was not found.
- kegg-mapk
  [+]

  No paths found.
  - PDLI1\_HUMAN was not found.
  - ICLN\_HUMAN was not found.
- pid-erbb1
  [+]

  No paths found.
  - PDLI1\_HUMAN was not found.
  - ICLN\_HUMAN was not found.
- phosphositeplus-kinase-substrate
  [+]

  No paths found.
  - PDLI1\_HUMAN was not found.
  - ICLN\_HUMAN was not found.
- biocarta-egf
  [+]

  No paths found.
  - PDLI1\_HUMAN was not found.
  - ICLN\_HUMAN was not found.
- cancer-cell-map-egfr1
  [+]

  No paths found.
  - PDLI1\_HUMAN was not found.
  - ICLN\_HUMAN was not found.
- science-signaling-egfr
  [+]

  No paths found.
  - PDLI1\_HUMAN was not found.
  - ICLN\_HUMAN was not found.
- layek
  [+]

  No paths found.
  - PDLI1\_HUMAN was not found.
  - ICLN\_HUMAN was not found.
- reactome-egfr
  [+]

  No paths found.
  - PDLI1\_HUMAN was not found.
  - ICLN\_HUMAN was not found.

### SUMO2\_HUMAN → SCAM3\_HUMAN / SUMO2\_HUMAN ⊣ SCAM3\_HUMAN

#### 1. Resources that have the edge


#### 2. Resources that contain endpoints (but not an edge)


#### 3. Resources that match only one endpoint (fringe)


#### 4. Resources that don't match any endpoint

- kegg-erbb
  [+]

  No paths found.
  - SCAM3\_HUMAN was not found.
  - SUMO2\_HUMAN was not found.
- phosphositeplus-kinase-substrate
  [+]

  No paths found.
  - SCAM3\_HUMAN was not found.
  - SUMO2\_HUMAN was not found.
- biocarta-egf
  [+]

  No paths found.
  - SCAM3\_HUMAN was not found.
  - SUMO2\_HUMAN was not found.
- pid-erbb1
  [+]

  No paths found.
  - SCAM3\_HUMAN was not found.
  - SUMO2\_HUMAN was not found.
- kegg-mapk
  [+]

  No paths found.
  - SCAM3\_HUMAN was not found.
  - SUMO2\_HUMAN was not found.
- reactome-egfr
  [+]

  No paths found.
  - SCAM3\_HUMAN was not found.
  - SUMO2\_HUMAN was not found.
- cancer-cell-map-egfr1
  [+]

  No paths found.
  - SCAM3\_HUMAN was not found.
  - SUMO2\_HUMAN was not found.
- layek
  [+]

  No paths found.
  - SCAM3\_HUMAN was not found.
  - SUMO2\_HUMAN was not found.
- science-signaling-egfr
  [+]

  No paths found.
  - SCAM3\_HUMAN was not found.
  - SUMO2\_HUMAN was not found.

### WDR48\_HUMAN → WDR70\_HUMAN

#### 1. Resources that have the edge


#### 2. Resources that contain endpoints (but not an edge)


#### 3. Resources that match only one endpoint (fringe)


#### 4. Resources that don't match any endpoint

- pid-erbb1
  [+]

  No paths found.
  - WDR70\_HUMAN was not found.
  - WDR48\_HUMAN was not found.
- kegg-erbb
  [+]

  No paths found.
  - WDR70\_HUMAN was not found.
  - WDR48\_HUMAN was not found.
- kegg-mapk
  [+]

  No paths found.
  - WDR70\_HUMAN was not found.
  - WDR48\_HUMAN was not found.
- biocarta-egf
  [+]

  No paths found.
  - WDR70\_HUMAN was not found.
  - WDR48\_HUMAN was not found.
- phosphositeplus-kinase-substrate
  [+]

  No paths found.
  - WDR70\_HUMAN was not found.
  - WDR48\_HUMAN was not found.
- cancer-cell-map-egfr1
  [+]

  No paths found.
  - WDR70\_HUMAN was not found.
  - WDR48\_HUMAN was not found.
- science-signaling-egfr
  [+]

  No paths found.
  - WDR70\_HUMAN was not found.
  - WDR48\_HUMAN was not found.
- reactome-egfr
  [+]

  No paths found.
  - WDR70\_HUMAN was not found.
  - WDR48\_HUMAN was not found.
- layek
  [+]

  No paths found.
  - WDR70\_HUMAN was not found.
  - WDR48\_HUMAN was not found.

### SVIL\_HUMAN ⊣ KIF4A\_HUMAN

#### 1. Resources that have the edge


#### 2. Resources that contain endpoints (but not an edge)


#### 3. Resources that match only one endpoint (fringe)


#### 4. Resources that don't match any endpoint

- kegg-mapk
  [+]

  No paths found.
  - SVIL\_HUMAN was not found.
  - KIF4A\_HUMAN was not found.
- biocarta-egf
  [+]

  No paths found.
  - SVIL\_HUMAN was not found.
  - KIF4A\_HUMAN was not found.
- pid-erbb1
  [+]

  No paths found.
  - SVIL\_HUMAN was not found.
  - KIF4A\_HUMAN was not found.
- reactome-egfr
  [+]

  No paths found.
  - SVIL\_HUMAN was not found.
  - KIF4A\_HUMAN was not found.
- kegg-erbb
  [+]

  No paths found.
  - SVIL\_HUMAN was not found.
  - KIF4A\_HUMAN was not found.
- science-signaling-egfr
  [+]

  No paths found.
  - SVIL\_HUMAN was not found.
  - KIF4A\_HUMAN was not found.
- cancer-cell-map-egfr1
  [+]

  No paths found.
  - SVIL\_HUMAN was not found.
  - KIF4A\_HUMAN was not found.
- phosphositeplus-kinase-substrate
  [+]

  No paths found.
  - SVIL\_HUMAN was not found.
  - KIF4A\_HUMAN was not found.
- layek
  [+]

  No paths found.
  - SVIL\_HUMAN was not found.
  - KIF4A\_HUMAN was not found.

### CCD53\_HUMAN → ABI2\_HUMAN / CCD53\_HUMAN ⊣ ABI2\_HUMAN

#### 1. Resources that have the edge


#### 2. Resources that contain endpoints (but not an edge)


#### 3. Resources that match only one endpoint (fringe)


#### 4. Resources that don't match any endpoint

- layek
  [+]

  No paths found.
  - ABI2\_HUMAN was not found.
  - CCD53\_HUMAN was not found.
- pid-erbb1
  [+]

  No paths found.
  - ABI2\_HUMAN was not found.
  - CCD53\_HUMAN was not found.
- phosphositeplus-kinase-substrate
  [+]

  No paths found.
  - ABI2\_HUMAN was not found.
  - CCD53\_HUMAN was not found.
- reactome-egfr
  [+]

  No paths found.
  - ABI2\_HUMAN was not found.
  - CCD53\_HUMAN was not found.
- biocarta-egf
  [+]

  No paths found.
  - ABI2\_HUMAN was not found.
  - CCD53\_HUMAN was not found.
- cancer-cell-map-egfr1
  [+]

  No paths found.
  - ABI2\_HUMAN was not found.
  - CCD53\_HUMAN was not found.
- science-signaling-egfr
  [+]

  No paths found.
  - ABI2\_HUMAN was not found.
  - CCD53\_HUMAN was not found.
- kegg-mapk
  [+]

  No paths found.
  - ABI2\_HUMAN was not found.
  - CCD53\_HUMAN was not found.
- kegg-erbb
  [+]

  No paths found.
  - ABI2\_HUMAN was not found.
  - CCD53\_HUMAN was not found.

### DDX55\_HUMAN ⊣ SPB1\_HUMAN

#### 1. Resources that have the edge


#### 2. Resources that contain endpoints (but not an edge)


#### 3. Resources that match only one endpoint (fringe)


#### 4. Resources that don't match any endpoint

- kegg-erbb
  [+]

  No paths found.
  - DDX55\_HUMAN was not found.
  - SPB1\_HUMAN was not found.
- science-signaling-egfr
  [+]

  No paths found.
  - DDX55\_HUMAN was not found.
  - SPB1\_HUMAN was not found.
- pid-erbb1
  [+]

  No paths found.
  - DDX55\_HUMAN was not found.
  - SPB1\_HUMAN was not found.
- cancer-cell-map-egfr1
  [+]

  No paths found.
  - DDX55\_HUMAN was not found.
  - SPB1\_HUMAN was not found.
- phosphositeplus-kinase-substrate
  [+]

  No paths found.
  - DDX55\_HUMAN was not found.
  - SPB1\_HUMAN was not found.
- layek
  [+]

  No paths found.
  - DDX55\_HUMAN was not found.
  - SPB1\_HUMAN was not found.
- biocarta-egf
  [+]

  No paths found.
  - DDX55\_HUMAN was not found.
  - SPB1\_HUMAN was not found.
- kegg-mapk
  [+]

  No paths found.
  - DDX55\_HUMAN was not found.
  - SPB1\_HUMAN was not found.
- reactome-egfr
  [+]

  No paths found.
  - DDX55\_HUMAN was not found.
  - SPB1\_HUMAN was not found.

### SVIL\_HUMAN → ACTB\_HUMAN / SVIL\_HUMAN ⊣ ACTB\_HUMAN

#### 1. Resources that have the edge


#### 2. Resources that contain endpoints (but not an edge)


#### 3. Resources that match only one endpoint (fringe)


#### 4. Resources that don't match any endpoint

- phosphositeplus-kinase-substrate
  [+]

  No paths found.
  - SVIL\_HUMAN was not found.
  - ACTB\_HUMAN was not found.
- cancer-cell-map-egfr1
  [+]

  No paths found.
  - SVIL\_HUMAN was not found.
  - ACTB\_HUMAN was not found.
- biocarta-egf
  [+]

  No paths found.
  - SVIL\_HUMAN was not found.
  - ACTB\_HUMAN was not found.
- pid-erbb1
  [+]

  No paths found.
  - SVIL\_HUMAN was not found.
  - ACTB\_HUMAN was not found.
- science-signaling-egfr
  [+]

  No paths found.
  - SVIL\_HUMAN was not found.
  - ACTB\_HUMAN was not found.
- layek
  [+]

  No paths found.
  - SVIL\_HUMAN was not found.
  - ACTB\_HUMAN was not found.
- reactome-egfr
  [+]

  No paths found.
  - SVIL\_HUMAN was not found.
  - ACTB\_HUMAN was not found.
- kegg-mapk
  [+]

  No paths found.
  - SVIL\_HUMAN was not found.
  - ACTB\_HUMAN was not found.
- kegg-erbb
  [+]

  No paths found.
  - SVIL\_HUMAN was not found.
  - ACTB\_HUMAN was not found.

### SUMO2\_HUMAN → E9PAU2\_HUMAN / SUMO2\_HUMAN ⊣ E9PAU2\_HUMAN

#### 1. Resources that have the edge


#### 2. Resources that contain endpoints (but not an edge)


#### 3. Resources that match only one endpoint (fringe)


#### 4. Resources that don't match any endpoint

- phosphositeplus-kinase-substrate
  [+]

  No paths found.
  - SUMO2\_HUMAN was not found.
  - E9PAU2\_HUMAN was not found.
- kegg-erbb
  [+]

  No paths found.
  - SUMO2\_HUMAN was not found.
  - E9PAU2\_HUMAN was not found.
- reactome-egfr
  [+]

  No paths found.
  - SUMO2\_HUMAN was not found.
  - E9PAU2\_HUMAN was not found.
- biocarta-egf
  [+]

  No paths found.
  - SUMO2\_HUMAN was not found.
  - E9PAU2\_HUMAN was not found.
- layek
  [+]

  No paths found.
  - SUMO2\_HUMAN was not found.
  - E9PAU2\_HUMAN was not found.
- kegg-mapk
  [+]

  No paths found.
  - SUMO2\_HUMAN was not found.
  - E9PAU2\_HUMAN was not found.
- pid-erbb1
  [+]

  No paths found.
  - SUMO2\_HUMAN was not found.
  - E9PAU2\_HUMAN was not found.
- cancer-cell-map-egfr1
  [+]

  No paths found.
  - SUMO2\_HUMAN was not found.
  - E9PAU2\_HUMAN was not found.
- science-signaling-egfr
  [+]

  No paths found.
  - SUMO2\_HUMAN was not found.
  - E9PAU2\_HUMAN was not found.

### EFNA5\_HUMAN → EPHA3\_HUMAN / EFNA5\_HUMAN ⊣ EPHA3\_HUMAN

#### 1. Resources that have the edge


#### 2. Resources that contain endpoints (but not an edge)


#### 3. Resources that match only one endpoint (fringe)


#### 4. Resources that don't match any endpoint

- biocarta-egf
  [+]

  No paths found.
  - EPHA3\_HUMAN was not found.
  - EFNA5\_HUMAN was not found.
- pid-erbb1
  [+]

  No paths found.
  - EPHA3\_HUMAN was not found.
  - EFNA5\_HUMAN was not found.
- cancer-cell-map-egfr1
  [+]

  No paths found.
  - EPHA3\_HUMAN was not found.
  - EFNA5\_HUMAN was not found.
- layek
  [+]

  No paths found.
  - EPHA3\_HUMAN was not found.
  - EFNA5\_HUMAN was not found.
- phosphositeplus-kinase-substrate
  [+]

  No paths found.
  - EPHA3\_HUMAN was not found.
  - EFNA5\_HUMAN was not found.
- kegg-mapk
  [+]

  No paths found.
  - EPHA3\_HUMAN was not found.
  - EFNA5\_HUMAN was not found.
- kegg-erbb
  [+]

  No paths found.
  - EPHA3\_HUMAN was not found.
  - EFNA5\_HUMAN was not found.
- reactome-egfr
  [+]

  No paths found.
  - EPHA3\_HUMAN was not found.
  - EFNA5\_HUMAN was not found.
- science-signaling-egfr
  [+]

  No paths found.
  - EPHA3\_HUMAN was not found.
  - EFNA5\_HUMAN was not found.

### AR6P4\_HUMAN → ARL6\_HUMAN / AR6P4\_HUMAN ⊣ ARL6\_HUMAN

#### 1. Resources that have the edge


#### 2. Resources that contain endpoints (but not an edge)


#### 3. Resources that match only one endpoint (fringe)


#### 4. Resources that don't match any endpoint

- pid-erbb1
  [+]

  No paths found.
  - ARL6\_HUMAN was not found.
  - AR6P4\_HUMAN was not found.
- biocarta-egf
  [+]

  No paths found.
  - ARL6\_HUMAN was not found.
  - AR6P4\_HUMAN was not found.
- phosphositeplus-kinase-substrate
  [+]

  No paths found.
  - ARL6\_HUMAN was not found.
  - AR6P4\_HUMAN was not found.
- reactome-egfr
  [+]

  No paths found.
  - ARL6\_HUMAN was not found.
  - AR6P4\_HUMAN was not found.
- cancer-cell-map-egfr1
  [+]

  No paths found.
  - ARL6\_HUMAN was not found.
  - AR6P4\_HUMAN was not found.
- layek
  [+]

  No paths found.
  - ARL6\_HUMAN was not found.
  - AR6P4\_HUMAN was not found.
- kegg-erbb
  [+]

  No paths found.
  - ARL6\_HUMAN was not found.
  - AR6P4\_HUMAN was not found.
- kegg-mapk
  [+]

  No paths found.
  - ARL6\_HUMAN was not found.
  - AR6P4\_HUMAN was not found.
- science-signaling-egfr
  [+]

  No paths found.
  - ARL6\_HUMAN was not found.
  - AR6P4\_HUMAN was not found.

### WAPL\_HUMAN → WAP53\_HUMAN / WAPL\_HUMAN ⊣ WAP53\_HUMAN

#### 1. Resources that have the edge


#### 2. Resources that contain endpoints (but not an edge)


#### 3. Resources that match only one endpoint (fringe)


#### 4. Resources that don't match any endpoint

- cancer-cell-map-egfr1
  [+]

  No paths found.
  - WAPL\_HUMAN was not found.
  - WAP53\_HUMAN was not found.
- science-signaling-egfr
  [+]

  No paths found.
  - WAPL\_HUMAN was not found.
  - WAP53\_HUMAN was not found.
- kegg-erbb
  [+]

  No paths found.
  - WAPL\_HUMAN was not found.
  - WAP53\_HUMAN was not found.
- phosphositeplus-kinase-substrate
  [+]

  No paths found.
  - WAPL\_HUMAN was not found.
  - WAP53\_HUMAN was not found.
- reactome-egfr
  [+]

  No paths found.
  - WAPL\_HUMAN was not found.
  - WAP53\_HUMAN was not found.
- biocarta-egf
  [+]

  No paths found.
  - WAPL\_HUMAN was not found.
  - WAP53\_HUMAN was not found.
- kegg-mapk
  [+]

  No paths found.
  - WAPL\_HUMAN was not found.
  - WAP53\_HUMAN was not found.
- layek
  [+]

  No paths found.
  - WAPL\_HUMAN was not found.
  - WAP53\_HUMAN was not found.
- pid-erbb1
  [+]

  No paths found.
  - WAPL\_HUMAN was not found.
  - WAP53\_HUMAN was not found.

### SUMO2\_HUMAN → PHIP\_HUMAN / SUMO2\_HUMAN ⊣ PHIP\_HUMAN

#### 1. Resources that have the edge


#### 2. Resources that contain endpoints (but not an edge)


#### 3. Resources that match only one endpoint (fringe)


#### 4. Resources that don't match any endpoint

- pid-erbb1
  [+]

  No paths found.
  - PHIP\_HUMAN was not found.
  - SUMO2\_HUMAN was not found.
- reactome-egfr
  [+]

  No paths found.
  - PHIP\_HUMAN was not found.
  - SUMO2\_HUMAN was not found.
- kegg-mapk
  [+]

  No paths found.
  - PHIP\_HUMAN was not found.
  - SUMO2\_HUMAN was not found.
- cancer-cell-map-egfr1
  [+]

  No paths found.
  - PHIP\_HUMAN was not found.
  - SUMO2\_HUMAN was not found.
- kegg-erbb
  [+]

  No paths found.
  - PHIP\_HUMAN was not found.
  - SUMO2\_HUMAN was not found.
- biocarta-egf
  [+]

  No paths found.
  - PHIP\_HUMAN was not found.
  - SUMO2\_HUMAN was not found.
- layek
  [+]

  No paths found.
  - PHIP\_HUMAN was not found.
  - SUMO2\_HUMAN was not found.
- phosphositeplus-kinase-substrate
  [+]

  No paths found.
  - PHIP\_HUMAN was not found.
  - SUMO2\_HUMAN was not found.
- science-signaling-egfr
  [+]

  No paths found.
  - PHIP\_HUMAN was not found.
  - SUMO2\_HUMAN was not found.

##
