# Supplementary material for: Synthesizing Signaling Pathways from Temporal Phosphoproteomic Data
Source: Cell Rep. Author manuscript; Available in PMC 2018 Dec 16. (PMC6295338; doi:10.1016/j.celrep.2018.08.085)
Supplement: 5 [file NIHMS1508952-supplement-5.zip › signed_directed_evaluation.html]

Evaluate TPS pathway signed, directed edges


# Analysis of TPS signed, directed predictions

## Statistics

Number of predictions with unambiguous sign and direction:
38
(9.2%)
  

## Color legend

- Exact prediction match
- Approximate prediction match
- Imprecise prediction
- Partial conflict
- Total conflict

## Edges

### GRB2\_HUMAN → PTN11\_HUMAN

#### 1. Resources that have the edge

- cancer-cell-map-egfr1
  [+]

  Resource has common complex(es):
  - physicalInteraction3002 {SHP2/SHPTP2/PTP-1D/SHPTP3/Growth factor receptor bound protein 2/Protein tyrosine phosphatase, non-receptor type 11/abundant SRC homology/ASH/GRB2/PTPN11} (Complex)
  - PTN11\_HUMAN and SHP2 are synonyms.
  - GRB2\_HUMAN and ASH are synonyms.
- pid-erbb1
  [+]

  Resource paths:
  - pid\_40369 {EGFR/EGFR/EGF/EGF/GRB2/GAB1} (Complex) → pid\_47514 {SHP2} (Protein) [Catalysis (activation)]
  - PTN11\_HUMAN and SHP2 are synonyms.
  - GRB2\_HUMAN and Grb2 are synonyms.
- reactome-egfr
  [+]

  Resource has common complex(es):
  - Complex3670 {SHP2-GRB2:Phospho GAB1(dephos)-EGF-Phospho-EGFR dimer, EGF:p-6Y-EGFR:GRB2:p-Y627,659-GAB1:SHP2} (Complex)
  - Complex3672 {SHP2:GRB2:Phospho GAB1-EGF-Phospho-EGFR (-Y992) dimer, EGF:p-5Y-EGFR:GRB2:p-5Y-GAB1:SHP2} (Complex)
  - Complex3669 {SHP2-GRB2:Phospho GAB1-EGF-Phospho-EGFR dimer, EGF:p-6Y-EGFR:GRB2:p-5Y-GAB1:SHP2} (Complex)
  - PTN11\_HUMAN and UniProt:Q06124 PTPN11 are synonyms.
  - GRB2\_HUMAN and ASH are synonyms.

#### 2. Resources that contain endpoints (but not an edge)

- phosphositeplus-kinase-substrate
  [+]

  No paths found.

#### 3. Resources that match only one endpoint (fringe)

- layek
  [+]

  No paths found.
  - PTN11\_HUMAN was not found.
- science-signaling-egfr
  [+]

  No paths found.
  - PTN11\_HUMAN was not found.
- kegg-erbb
  [+]

  No paths found.
  - PTN11\_HUMAN was not found.
- biocarta-egf
  [+]

  No paths found.
  - PTN11\_HUMAN was not found.
- kegg-mapk
  [+]

  No paths found.
  - PTN11\_HUMAN was not found.

#### 4. Resources that don't match any endpoint


### GRB2\_HUMAN ⊣ WASL\_HUMAN

#### 1. Resources that have the edge

- pid-erbb1
  [+]

  Resource has common complex(es):
  - pid\_47942 {GRB2/N-WASP} (Complex)
  - pid\_47699 {EGFR/EGFR/EGF/EGF/GRB2/N-WASP} (Complex)
  - GRB2\_HUMAN and Grb2 are synonyms.
  - WASL\_HUMAN and N-WASP are synonyms.

#### 2. Resources that contain endpoints (but not an edge)

- science-signaling-egfr
  [+]

  No paths found.

#### 3. Resources that match only one endpoint (fringe)

- cancer-cell-map-egfr1
  [+]

  No paths found.
  - WASL\_HUMAN was not found.
- kegg-erbb
  [+]

  No paths found.
  - WASL\_HUMAN was not found.
- biocarta-egf
  [+]

  No paths found.
  - WASL\_HUMAN was not found.
- reactome-egfr
  [+]

  No paths found.
  - WASL\_HUMAN was not found.
- phosphositeplus-kinase-substrate
  [+]

  No paths found.
  - WASL\_HUMAN was not found.
- kegg-mapk
  [+]

  No paths found.
  - WASL\_HUMAN was not found.
- layek
  [+]

  No paths found.
  - WASL\_HUMAN was not found.

#### 4. Resources that don't match any endpoint


### MK01\_HUMAN ⊣ ABI1\_HUMAN

#### 1. Resources that have the edge

- phosphositeplus-kinase-substrate
  [+]

  Resource paths:
  - P28482 {MK01\_HUMAN} (Protein) → Q8IZP0 {ABI1\_HUMAN} (Protein) [ProteinProteinInteraction (unknown sign)]

#### 2. Resources that contain endpoints (but not an edge)

- cancer-cell-map-egfr1
  [+]

  No paths found.

#### 3. Resources that match only one endpoint (fringe)

- layek
  [+]

  No paths found.
  - ABI1\_HUMAN was not found.
- kegg-mapk
  [+]

  No paths found.
  - ABI1\_HUMAN was not found.
- kegg-erbb
  [+]

  No paths found.
  - ABI1\_HUMAN was not found.
- reactome-egfr
  [+]

  No paths found.
  - ABI1\_HUMAN was not found.
- pid-erbb1
  [+]

  No paths found.
  - ABI1\_HUMAN was not found.
- science-signaling-egfr
  [+]

  No paths found.
  - ABI1\_HUMAN was not found.

#### 4. Resources that don't match any endpoint

- biocarta-egf
  [+]

  No paths found.
  - MK01\_HUMAN was not found.
  - ABI1\_HUMAN was not found.

### EGFR\_HUMAN → CCD50\_HUMAN

#### 1. Resources that have the edge

- phosphositeplus-kinase-substrate
  [+]

  Resource paths:
  - P00533 {EGFR\_HUMAN} (Protein) → Q8IVM0 {CCD50\_HUMAN} (Protein) [ProteinProteinInteraction (unknown sign)]

#### 2. Resources that contain endpoints (but not an edge)


#### 3. Resources that match only one endpoint (fringe)

- science-signaling-egfr
  [+]

  No paths found.
  - CCD50\_HUMAN was not found.
- biocarta-egf
  [+]

  No paths found.
  - CCD50\_HUMAN was not found.
- kegg-erbb
  [+]

  No paths found.
  - CCD50\_HUMAN was not found.
- reactome-egfr
  [+]

  No paths found.
  - CCD50\_HUMAN was not found.
- cancer-cell-map-egfr1
  [+]

  No paths found.
  - CCD50\_HUMAN was not found.
- pid-erbb1
  [+]

  No paths found.
  - CCD50\_HUMAN was not found.
- layek
  [+]

  No paths found.
  - CCD50\_HUMAN was not found.
- kegg-mapk
  [+]

  No paths found.
  - CCD50\_HUMAN was not found.

#### 4. Resources that don't match any endpoint


### MK01\_HUMAN → MKL1\_HUMAN

#### 1. Resources that have the edge

- phosphositeplus-kinase-substrate
  [+]

  Resource paths:
  - P28482 {MK01\_HUMAN} (Protein) → Q969V6 {MKL1\_HUMAN} (Protein) [ProteinProteinInteraction (unknown sign)]

#### 2. Resources that contain endpoints (but not an edge)


#### 3. Resources that match only one endpoint (fringe)

- science-signaling-egfr
  [+]

  No paths found.
  - MKL1\_HUMAN was not found.
- pid-erbb1
  [+]

  No paths found.
  - MKL1\_HUMAN was not found.
- kegg-erbb
  [+]

  No paths found.
  - MKL1\_HUMAN was not found.
- reactome-egfr
  [+]

  No paths found.
  - MKL1\_HUMAN was not found.
- layek
  [+]

  No paths found.
  - MKL1\_HUMAN was not found.
- kegg-mapk
  [+]

  No paths found.
  - MKL1\_HUMAN was not found.
- cancer-cell-map-egfr1
  [+]

  No paths found.
  - MKL1\_HUMAN was not found.

#### 4. Resources that don't match any endpoint

- biocarta-egf
  [+]

  No paths found.
  - MK01\_HUMAN was not found.
  - MKL1\_HUMAN was not found.

### MK03\_HUMAN → MKL1\_HUMAN

#### 1. Resources that have the edge

- phosphositeplus-kinase-substrate
  [+]

  Resource paths:
  - P27361 {MK03\_HUMAN} (Protein) → Q969V6 {MKL1\_HUMAN} (Protein) [ProteinProteinInteraction (unknown sign)]

#### 2. Resources that contain endpoints (but not an edge)


#### 3. Resources that match only one endpoint (fringe)

- cancer-cell-map-egfr1
  [+]

  No paths found.
  - MKL1\_HUMAN was not found.
- layek
  [+]

  No paths found.
  - MKL1\_HUMAN was not found.
- reactome-egfr
  [+]

  No paths found.
  - MKL1\_HUMAN was not found.
- science-signaling-egfr
  [+]

  No paths found.
  - MKL1\_HUMAN was not found.

#### 4. Resources that don't match any endpoint

- kegg-mapk
  [+]

  No paths found.
  - MK03\_HUMAN was not found.
  - MKL1\_HUMAN was not found.
- kegg-erbb
  [+]

  No paths found.
  - MK03\_HUMAN was not found.
  - MKL1\_HUMAN was not found.
- pid-erbb1
  [+]

  No paths found.
  - MK03\_HUMAN was not found.
  - MKL1\_HUMAN was not found.
- biocarta-egf
  [+]

  No paths found.
  - MK03\_HUMAN was not found.
  - MKL1\_HUMAN was not found.

### CDK1\_HUMAN ⊣ DDX3X\_HUMAN

#### 1. Resources that have the edge

- phosphositeplus-kinase-substrate
  [+]

  Resource paths:
  - P06493 {CDK1\_HUMAN} (Protein) → O00571 {DDX3X\_HUMAN} (Protein) [ProteinProteinInteraction (unknown sign)]

#### 2. Resources that contain endpoints (but not an edge)


#### 3. Resources that match only one endpoint (fringe)


#### 4. Resources that don't match any endpoint

- reactome-egfr
  [+]

  No paths found.
  - CDK1\_HUMAN was not found.
  - DDX3X\_HUMAN was not found.
- kegg-erbb
  [+]

  No paths found.
  - CDK1\_HUMAN was not found.
  - DDX3X\_HUMAN was not found.
- biocarta-egf
  [+]

  No paths found.
  - CDK1\_HUMAN was not found.
  - DDX3X\_HUMAN was not found.
- layek
  [+]

  No paths found.
  - CDK1\_HUMAN was not found.
  - DDX3X\_HUMAN was not found.
- pid-erbb1
  [+]

  No paths found.
  - CDK1\_HUMAN was not found.
  - DDX3X\_HUMAN was not found.
- kegg-mapk
  [+]

  No paths found.
  - CDK1\_HUMAN was not found.
  - DDX3X\_HUMAN was not found.
- science-signaling-egfr
  [+]

  No paths found.
  - CDK1\_HUMAN was not found.
  - DDX3X\_HUMAN was not found.
- cancer-cell-map-egfr1
  [+]

  No paths found.
  - CDK1\_HUMAN was not found.
  - DDX3X\_HUMAN was not found.

### CDK1\_HUMAN ⊣ STMN1\_HUMAN

#### 1. Resources that have the edge

- phosphositeplus-kinase-substrate
  [+]

  Resource paths:
  - P06493 {CDK1\_HUMAN} (Protein) → P16949 {STMN1\_HUMAN} (Protein) [ProteinProteinInteraction (unknown sign)]

#### 2. Resources that contain endpoints (but not an edge)


#### 3. Resources that match only one endpoint (fringe)


#### 4. Resources that don't match any endpoint

- biocarta-egf
  [+]

  No paths found.
  - CDK1\_HUMAN was not found.
  - STMN1\_HUMAN was not found.
- reactome-egfr
  [+]

  No paths found.
  - CDK1\_HUMAN was not found.
  - STMN1\_HUMAN was not found.
- kegg-erbb
  [+]

  No paths found.
  - CDK1\_HUMAN was not found.
  - STMN1\_HUMAN was not found.
- kegg-mapk
  [+]

  No paths found.
  - CDK1\_HUMAN was not found.
  - STMN1\_HUMAN was not found.
- layek
  [+]

  No paths found.
  - CDK1\_HUMAN was not found.
  - STMN1\_HUMAN was not found.
- cancer-cell-map-egfr1
  [+]

  No paths found.
  - CDK1\_HUMAN was not found.
  - STMN1\_HUMAN was not found.
- science-signaling-egfr
  [+]

  No paths found.
  - CDK1\_HUMAN was not found.
  - STMN1\_HUMAN was not found.
- pid-erbb1
  [+]

  No paths found.
  - CDK1\_HUMAN was not found.
  - STMN1\_HUMAN was not found.

### CDK1\_HUMAN → DNM1L\_HUMAN

#### 1. Resources that have the edge

- phosphositeplus-kinase-substrate
  [+]

  Resource paths:
  - P06493 {CDK1\_HUMAN} (Protein) → O00429 {DNM1L\_HUMAN} (Protein) [ProteinProteinInteraction (unknown sign)]

#### 2. Resources that contain endpoints (but not an edge)


#### 3. Resources that match only one endpoint (fringe)


#### 4. Resources that don't match any endpoint

- reactome-egfr
  [+]

  No paths found.
  - CDK1\_HUMAN was not found.
  - DNM1L\_HUMAN was not found.
- layek
  [+]

  No paths found.
  - CDK1\_HUMAN was not found.
  - DNM1L\_HUMAN was not found.
- cancer-cell-map-egfr1
  [+]

  No paths found.
  - CDK1\_HUMAN was not found.
  - DNM1L\_HUMAN was not found.
- kegg-mapk
  [+]

  No paths found.
  - CDK1\_HUMAN was not found.
  - DNM1L\_HUMAN was not found.
- biocarta-egf
  [+]

  No paths found.
  - CDK1\_HUMAN was not found.
  - DNM1L\_HUMAN was not found.
- kegg-erbb
  [+]

  No paths found.
  - CDK1\_HUMAN was not found.
  - DNM1L\_HUMAN was not found.
- pid-erbb1
  [+]

  No paths found.
  - CDK1\_HUMAN was not found.
  - DNM1L\_HUMAN was not found.
- science-signaling-egfr
  [+]

  No paths found.
  - CDK1\_HUMAN was not found.
  - DNM1L\_HUMAN was not found.

### CDK1\_HUMAN → PUR6\_HUMAN

#### 1. Resources that have the edge

- phosphositeplus-kinase-substrate
  [+]

  Resource paths:
  - P06493 {CDK1\_HUMAN} (Protein) → P22234 {PUR6\_HUMAN} (Protein) [ProteinProteinInteraction (unknown sign)]

#### 2. Resources that contain endpoints (but not an edge)


#### 3. Resources that match only one endpoint (fringe)


#### 4. Resources that don't match any endpoint

- science-signaling-egfr
  [+]

  No paths found.
  - CDK1\_HUMAN was not found.
  - PUR6\_HUMAN was not found.
- pid-erbb1
  [+]

  No paths found.
  - CDK1\_HUMAN was not found.
  - PUR6\_HUMAN was not found.
- layek
  [+]

  No paths found.
  - CDK1\_HUMAN was not found.
  - PUR6\_HUMAN was not found.
- biocarta-egf
  [+]

  No paths found.
  - CDK1\_HUMAN was not found.
  - PUR6\_HUMAN was not found.
- kegg-erbb
  [+]

  No paths found.
  - CDK1\_HUMAN was not found.
  - PUR6\_HUMAN was not found.
- cancer-cell-map-egfr1
  [+]

  No paths found.
  - CDK1\_HUMAN was not found.
  - PUR6\_HUMAN was not found.
- reactome-egfr
  [+]

  No paths found.
  - CDK1\_HUMAN was not found.
  - PUR6\_HUMAN was not found.
- kegg-mapk
  [+]

  No paths found.
  - CDK1\_HUMAN was not found.
  - PUR6\_HUMAN was not found.

### CDK1\_HUMAN ⊣ TOP2B\_HUMAN

#### 1. Resources that have the edge

- phosphositeplus-kinase-substrate
  [+]

  Resource paths:
  - P06493 {CDK1\_HUMAN} (Protein) → Q02880 {TOP2B\_HUMAN} (Protein) [ProteinProteinInteraction (unknown sign)]

#### 2. Resources that contain endpoints (but not an edge)


#### 3. Resources that match only one endpoint (fringe)


#### 4. Resources that don't match any endpoint

- kegg-mapk
  [+]

  No paths found.
  - CDK1\_HUMAN was not found.
  - TOP2B\_HUMAN was not found.
- science-signaling-egfr
  [+]

  No paths found.
  - CDK1\_HUMAN was not found.
  - TOP2B\_HUMAN was not found.
- pid-erbb1
  [+]

  No paths found.
  - CDK1\_HUMAN was not found.
  - TOP2B\_HUMAN was not found.
- layek
  [+]

  No paths found.
  - CDK1\_HUMAN was not found.
  - TOP2B\_HUMAN was not found.
- biocarta-egf
  [+]

  No paths found.
  - CDK1\_HUMAN was not found.
  - TOP2B\_HUMAN was not found.
- cancer-cell-map-egfr1
  [+]

  No paths found.
  - CDK1\_HUMAN was not found.
  - TOP2B\_HUMAN was not found.
- reactome-egfr
  [+]

  No paths found.
  - CDK1\_HUMAN was not found.
  - TOP2B\_HUMAN was not found.
- kegg-erbb
  [+]

  No paths found.
  - CDK1\_HUMAN was not found.
  - TOP2B\_HUMAN was not found.

### CDK1\_HUMAN ⊣ NSF1C\_HUMAN

#### 1. Resources that have the edge

- phosphositeplus-kinase-substrate
  [+]

  Resource paths:
  - P06493 {CDK1\_HUMAN} (Protein) → Q9UNZ2 {NSF1C\_HUMAN} (Protein) [ProteinProteinInteraction (unknown sign)]

#### 2. Resources that contain endpoints (but not an edge)


#### 3. Resources that match only one endpoint (fringe)


#### 4. Resources that don't match any endpoint

- pid-erbb1
  [+]

  No paths found.
  - CDK1\_HUMAN was not found.
  - NSF1C\_HUMAN was not found.
- cancer-cell-map-egfr1
  [+]

  No paths found.
  - CDK1\_HUMAN was not found.
  - NSF1C\_HUMAN was not found.
- layek
  [+]

  No paths found.
  - CDK1\_HUMAN was not found.
  - NSF1C\_HUMAN was not found.
- reactome-egfr
  [+]

  No paths found.
  - CDK1\_HUMAN was not found.
  - NSF1C\_HUMAN was not found.
- kegg-erbb
  [+]

  No paths found.
  - CDK1\_HUMAN was not found.
  - NSF1C\_HUMAN was not found.
- science-signaling-egfr
  [+]

  No paths found.
  - CDK1\_HUMAN was not found.
  - NSF1C\_HUMAN was not found.
- biocarta-egf
  [+]

  No paths found.
  - CDK1\_HUMAN was not found.
  - NSF1C\_HUMAN was not found.
- kegg-mapk
  [+]

  No paths found.
  - CDK1\_HUMAN was not found.
  - NSF1C\_HUMAN was not found.

### MK01\_HUMAN ⊣ DYR1B\_HUMAN

#### 1. Resources that have the edge


#### 2. Resources that contain endpoints (but not an edge)

- phosphositeplus-kinase-substrate
  [+]

  No paths found.

#### 3. Resources that match only one endpoint (fringe)

- pid-erbb1
  [+]

  No paths found.
  - DYR1B\_HUMAN was not found.
- kegg-erbb
  [+]

  No paths found.
  - DYR1B\_HUMAN was not found.
- cancer-cell-map-egfr1
  [+]

  No paths found.
  - DYR1B\_HUMAN was not found.
- reactome-egfr
  [+]

  No paths found.
  - DYR1B\_HUMAN was not found.
- science-signaling-egfr
  [+]

  No paths found.
  - DYR1B\_HUMAN was not found.
- layek
  [+]

  No paths found.
  - DYR1B\_HUMAN was not found.
- kegg-mapk
  [+]

  No paths found.
  - DYR1B\_HUMAN was not found.

#### 4. Resources that don't match any endpoint

- biocarta-egf
  [+]

  No paths found.
  - DYR1B\_HUMAN was not found.
  - MK01\_HUMAN was not found.

### MK01\_HUMAN ⊣ ICK\_HUMAN

#### 1. Resources that have the edge


#### 2. Resources that contain endpoints (but not an edge)

- phosphositeplus-kinase-substrate
  [+]

  No paths found.

#### 3. Resources that match only one endpoint (fringe)

- kegg-erbb
  [+]

  No paths found.
  - ICK\_HUMAN was not found.
- science-signaling-egfr
  [+]

  No paths found.
  - ICK\_HUMAN was not found.
- layek
  [+]

  No paths found.
  - ICK\_HUMAN was not found.
- reactome-egfr
  [+]

  No paths found.
  - ICK\_HUMAN was not found.
- pid-erbb1
  [+]

  No paths found.
  - ICK\_HUMAN was not found.
- kegg-mapk
  [+]

  No paths found.
  - ICK\_HUMAN was not found.
- cancer-cell-map-egfr1
  [+]

  No paths found.
  - ICK\_HUMAN was not found.

#### 4. Resources that don't match any endpoint

- biocarta-egf
  [+]

  No paths found.
  - MK01\_HUMAN was not found.
  - ICK\_HUMAN was not found.

### HIPK2\_HUMAN → DYR1B\_HUMAN

#### 1. Resources that have the edge


#### 2. Resources that contain endpoints (but not an edge)

- phosphositeplus-kinase-substrate
  [+]

  No paths found.

#### 3. Resources that match only one endpoint (fringe)


#### 4. Resources that don't match any endpoint

- biocarta-egf
  [+]

  No paths found.
  - DYR1B\_HUMAN was not found.
  - HIPK2\_HUMAN was not found.
- kegg-erbb
  [+]

  No paths found.
  - DYR1B\_HUMAN was not found.
  - HIPK2\_HUMAN was not found.
- pid-erbb1
  [+]

  No paths found.
  - DYR1B\_HUMAN was not found.
  - HIPK2\_HUMAN was not found.
- kegg-mapk
  [+]

  No paths found.
  - DYR1B\_HUMAN was not found.
  - HIPK2\_HUMAN was not found.
- cancer-cell-map-egfr1
  [+]

  No paths found.
  - DYR1B\_HUMAN was not found.
  - HIPK2\_HUMAN was not found.
- layek
  [+]

  No paths found.
  - DYR1B\_HUMAN was not found.
  - HIPK2\_HUMAN was not found.
- science-signaling-egfr
  [+]

  No paths found.
  - DYR1B\_HUMAN was not found.
  - HIPK2\_HUMAN was not found.
- reactome-egfr
  [+]

  No paths found.
  - DYR1B\_HUMAN was not found.
  - HIPK2\_HUMAN was not found.

### DDX3X\_HUMAN → IF4G1\_HUMAN

#### 1. Resources that have the edge


#### 2. Resources that contain endpoints (but not an edge)

- phosphositeplus-kinase-substrate
  [+]

  No paths found.

#### 3. Resources that match only one endpoint (fringe)


#### 4. Resources that don't match any endpoint

- biocarta-egf
  [+]

  No paths found.
  - DDX3X\_HUMAN was not found.
  - IF4G1\_HUMAN was not found.
- reactome-egfr
  [+]

  No paths found.
  - DDX3X\_HUMAN was not found.
  - IF4G1\_HUMAN was not found.
- layek
  [+]

  No paths found.
  - DDX3X\_HUMAN was not found.
  - IF4G1\_HUMAN was not found.
- kegg-mapk
  [+]

  No paths found.
  - DDX3X\_HUMAN was not found.
  - IF4G1\_HUMAN was not found.
- pid-erbb1
  [+]

  No paths found.
  - DDX3X\_HUMAN was not found.
  - IF4G1\_HUMAN was not found.
- kegg-erbb
  [+]

  No paths found.
  - DDX3X\_HUMAN was not found.
  - IF4G1\_HUMAN was not found.
- science-signaling-egfr
  [+]

  No paths found.
  - DDX3X\_HUMAN was not found.
  - IF4G1\_HUMAN was not found.
- cancer-cell-map-egfr1
  [+]

  No paths found.
  - DDX3X\_HUMAN was not found.
  - IF4G1\_HUMAN was not found.

### SRRM1\_HUMAN → SRRM2\_HUMAN

#### 1. Resources that have the edge


#### 2. Resources that contain endpoints (but not an edge)

- phosphositeplus-kinase-substrate
  [+]

  No paths found.

#### 3. Resources that match only one endpoint (fringe)


#### 4. Resources that don't match any endpoint

- science-signaling-egfr
  [+]

  No paths found.
  - SRRM2\_HUMAN was not found.
  - SRRM1\_HUMAN was not found.
- reactome-egfr
  [+]

  No paths found.
  - SRRM2\_HUMAN was not found.
  - SRRM1\_HUMAN was not found.
- pid-erbb1
  [+]

  No paths found.
  - SRRM2\_HUMAN was not found.
  - SRRM1\_HUMAN was not found.
- cancer-cell-map-egfr1
  [+]

  No paths found.
  - SRRM2\_HUMAN was not found.
  - SRRM1\_HUMAN was not found.
- biocarta-egf
  [+]

  No paths found.
  - SRRM2\_HUMAN was not found.
  - SRRM1\_HUMAN was not found.
- layek
  [+]

  No paths found.
  - SRRM2\_HUMAN was not found.
  - SRRM1\_HUMAN was not found.
- kegg-erbb
  [+]

  No paths found.
  - SRRM2\_HUMAN was not found.
  - SRRM1\_HUMAN was not found.
- kegg-mapk
  [+]

  No paths found.
  - SRRM2\_HUMAN was not found.
  - SRRM1\_HUMAN was not found.

### AT1A1\_HUMAN ⊣ ADDA\_HUMAN

#### 1. Resources that have the edge


#### 2. Resources that contain endpoints (but not an edge)

- phosphositeplus-kinase-substrate
  [+]

  No paths found.

#### 3. Resources that match only one endpoint (fringe)


#### 4. Resources that don't match any endpoint

- layek
  [+]

  No paths found.
  - ADDA\_HUMAN was not found.
  - AT1A1\_HUMAN was not found.
- biocarta-egf
  [+]

  No paths found.
  - ADDA\_HUMAN was not found.
  - AT1A1\_HUMAN was not found.
- reactome-egfr
  [+]

  No paths found.
  - ADDA\_HUMAN was not found.
  - AT1A1\_HUMAN was not found.
- cancer-cell-map-egfr1
  [+]

  No paths found.
  - ADDA\_HUMAN was not found.
  - AT1A1\_HUMAN was not found.
- pid-erbb1
  [+]

  No paths found.
  - ADDA\_HUMAN was not found.
  - AT1A1\_HUMAN was not found.
- kegg-mapk
  [+]

  No paths found.
  - ADDA\_HUMAN was not found.
  - AT1A1\_HUMAN was not found.
- kegg-erbb
  [+]

  No paths found.
  - ADDA\_HUMAN was not found.
  - AT1A1\_HUMAN was not found.
- science-signaling-egfr
  [+]

  No paths found.
  - ADDA\_HUMAN was not found.
  - AT1A1\_HUMAN was not found.

### CDK5\_HUMAN ⊣ ATX2L\_HUMAN

#### 1. Resources that have the edge


#### 2. Resources that contain endpoints (but not an edge)

- phosphositeplus-kinase-substrate
  [+]

  No paths found.

#### 3. Resources that match only one endpoint (fringe)


#### 4. Resources that don't match any endpoint

- biocarta-egf
  [+]

  No paths found.
  - ATX2L\_HUMAN was not found.
  - CDK5\_HUMAN was not found.
- science-signaling-egfr
  [+]

  No paths found.
  - ATX2L\_HUMAN was not found.
  - CDK5\_HUMAN was not found.
- pid-erbb1
  [+]

  No paths found.
  - ATX2L\_HUMAN was not found.
  - CDK5\_HUMAN was not found.
- cancer-cell-map-egfr1
  [+]

  No paths found.
  - ATX2L\_HUMAN was not found.
  - CDK5\_HUMAN was not found.
- layek
  [+]

  No paths found.
  - ATX2L\_HUMAN was not found.
  - CDK5\_HUMAN was not found.
- reactome-egfr
  [+]

  No paths found.
  - ATX2L\_HUMAN was not found.
  - CDK5\_HUMAN was not found.
- kegg-mapk
  [+]

  No paths found.
  - ATX2L\_HUMAN was not found.
  - CDK5\_HUMAN was not found.
- kegg-erbb
  [+]

  No paths found.
  - ATX2L\_HUMAN was not found.
  - CDK5\_HUMAN was not found.

### GRB2\_HUMAN → PTN18\_HUMAN

#### 1. Resources that have the edge


#### 2. Resources that contain endpoints (but not an edge)


#### 3. Resources that match only one endpoint (fringe)

- science-signaling-egfr
  [+]

  No paths found.
  - PTN18\_HUMAN was not found.
- phosphositeplus-kinase-substrate
  [+]

  No paths found.
  - PTN18\_HUMAN was not found.
- kegg-erbb
  [+]

  No paths found.
  - PTN18\_HUMAN was not found.
- pid-erbb1
  [+]

  No paths found.
  - PTN18\_HUMAN was not found.
- reactome-egfr
  [+]

  No paths found.
  - PTN18\_HUMAN was not found.
- kegg-mapk
  [+]

  No paths found.
  - PTN18\_HUMAN was not found.
- layek
  [+]

  No paths found.
  - PTN18\_HUMAN was not found.
- cancer-cell-map-egfr1
  [+]

  No paths found.
  - PTN18\_HUMAN was not found.
- biocarta-egf
  [+]

  No paths found.
  - PTN18\_HUMAN was not found.

#### 4. Resources that don't match any endpoint


### GRB2\_HUMAN → DOCK4\_HUMAN

#### 1. Resources that have the edge


#### 2. Resources that contain endpoints (but not an edge)


#### 3. Resources that match only one endpoint (fringe)

- reactome-egfr
  [+]

  No paths found.
  - DOCK4\_HUMAN was not found.
- phosphositeplus-kinase-substrate
  [+]

  No paths found.
  - DOCK4\_HUMAN was not found.
- kegg-erbb
  [+]

  No paths found.
  - DOCK4\_HUMAN was not found.
- science-signaling-egfr
  [+]

  No paths found.
  - DOCK4\_HUMAN was not found.
- pid-erbb1
  [+]

  No paths found.
  - DOCK4\_HUMAN was not found.
- kegg-mapk
  [+]

  No paths found.
  - DOCK4\_HUMAN was not found.
- cancer-cell-map-egfr1
  [+]

  No paths found.
  - DOCK4\_HUMAN was not found.
- layek
  [+]

  No paths found.
  - DOCK4\_HUMAN was not found.
- biocarta-egf
  [+]

  No paths found.
  - DOCK4\_HUMAN was not found.

#### 4. Resources that don't match any endpoint


### GRB2\_HUMAN → GAREM\_HUMAN

#### 1. Resources that have the edge


#### 2. Resources that contain endpoints (but not an edge)


#### 3. Resources that match only one endpoint (fringe)

- science-signaling-egfr
  [+]

  No paths found.
  - GAREM\_HUMAN was not found.
- layek
  [+]

  No paths found.
  - GAREM\_HUMAN was not found.
- kegg-erbb
  [+]

  No paths found.
  - GAREM\_HUMAN was not found.
- pid-erbb1
  [+]

  No paths found.
  - GAREM\_HUMAN was not found.
- kegg-mapk
  [+]

  No paths found.
  - GAREM\_HUMAN was not found.
- reactome-egfr
  [+]

  No paths found.
  - GAREM\_HUMAN was not found.
- cancer-cell-map-egfr1
  [+]

  No paths found.
  - GAREM\_HUMAN was not found.
- phosphositeplus-kinase-substrate
  [+]

  No paths found.
  - GAREM\_HUMAN was not found.
- biocarta-egf
  [+]

  No paths found.
  - GAREM\_HUMAN was not found.

#### 4. Resources that don't match any endpoint


### GRB2\_HUMAN → WIPF2\_HUMAN

#### 1. Resources that have the edge


#### 2. Resources that contain endpoints (but not an edge)


#### 3. Resources that match only one endpoint (fringe)

- pid-erbb1
  [+]

  No paths found.
  - WIPF2\_HUMAN was not found.
- cancer-cell-map-egfr1
  [+]

  No paths found.
  - WIPF2\_HUMAN was not found.
- science-signaling-egfr
  [+]

  No paths found.
  - WIPF2\_HUMAN was not found.
- kegg-erbb
  [+]

  No paths found.
  - WIPF2\_HUMAN was not found.
- biocarta-egf
  [+]

  No paths found.
  - WIPF2\_HUMAN was not found.
- kegg-mapk
  [+]

  No paths found.
  - WIPF2\_HUMAN was not found.
- phosphositeplus-kinase-substrate
  [+]

  No paths found.
  - WIPF2\_HUMAN was not found.
- layek
  [+]

  No paths found.
  - WIPF2\_HUMAN was not found.
- reactome-egfr
  [+]

  No paths found.
  - WIPF2\_HUMAN was not found.

#### 4. Resources that don't match any endpoint


### MK01\_HUMAN ⊣ KPRA\_HUMAN

#### 1. Resources that have the edge


#### 2. Resources that contain endpoints (but not an edge)


#### 3. Resources that match only one endpoint (fringe)

- reactome-egfr
  [+]

  No paths found.
  - KPRA\_HUMAN was not found.
- layek
  [+]

  No paths found.
  - KPRA\_HUMAN was not found.
- phosphositeplus-kinase-substrate
  [+]

  No paths found.
  - KPRA\_HUMAN was not found.
- pid-erbb1
  [+]

  No paths found.
  - KPRA\_HUMAN was not found.
- cancer-cell-map-egfr1
  [+]

  No paths found.
  - KPRA\_HUMAN was not found.
- kegg-mapk
  [+]

  No paths found.
  - KPRA\_HUMAN was not found.
- kegg-erbb
  [+]

  No paths found.
  - KPRA\_HUMAN was not found.
- science-signaling-egfr
  [+]

  No paths found.
  - KPRA\_HUMAN was not found.

#### 4. Resources that don't match any endpoint

- biocarta-egf
  [+]

  No paths found.
  - MK01\_HUMAN was not found.
  - KPRA\_HUMAN was not found.

### PLCG1\_HUMAN → ARHGB\_HUMAN

#### 1. Resources that have the edge


#### 2. Resources that contain endpoints (but not an edge)


#### 3. Resources that match only one endpoint (fringe)

- biocarta-egf
  [+]

  No paths found.
  - ARHGB\_HUMAN was not found.
- cancer-cell-map-egfr1
  [+]

  No paths found.
  - ARHGB\_HUMAN was not found.
- phosphositeplus-kinase-substrate
  [+]

  No paths found.
  - ARHGB\_HUMAN was not found.
- pid-erbb1
  [+]

  No paths found.
  - ARHGB\_HUMAN was not found.
- reactome-egfr
  [+]

  No paths found.
  - ARHGB\_HUMAN was not found.
- kegg-erbb
  [+]

  No paths found.
  - ARHGB\_HUMAN was not found.

#### 4. Resources that don't match any endpoint

- kegg-mapk
  [+]

  No paths found.
  - ARHGB\_HUMAN was not found.
  - PLCG1\_HUMAN was not found.
- layek
  [+]

  No paths found.
  - ARHGB\_HUMAN was not found.
  - PLCG1\_HUMAN was not found.
- science-signaling-egfr
  [+]

  No paths found.
  - ARHGB\_HUMAN was not found.
  - PLCG1\_HUMAN was not found.

### DREB\_HUMAN → MARCS\_HUMAN

#### 1. Resources that have the edge


#### 2. Resources that contain endpoints (but not an edge)


#### 3. Resources that match only one endpoint (fringe)

- phosphositeplus-kinase-substrate
  [+]

  No paths found.
  - DREB\_HUMAN was not found.

#### 4. Resources that don't match any endpoint

- biocarta-egf
  [+]

  No paths found.
  - MARCS\_HUMAN was not found.
  - DREB\_HUMAN was not found.
- pid-erbb1
  [+]

  No paths found.
  - MARCS\_HUMAN was not found.
  - DREB\_HUMAN was not found.
- kegg-erbb
  [+]

  No paths found.
  - MARCS\_HUMAN was not found.
  - DREB\_HUMAN was not found.
- cancer-cell-map-egfr1
  [+]

  No paths found.
  - MARCS\_HUMAN was not found.
  - DREB\_HUMAN was not found.
- layek
  [+]

  No paths found.
  - MARCS\_HUMAN was not found.
  - DREB\_HUMAN was not found.
- reactome-egfr
  [+]

  No paths found.
  - MARCS\_HUMAN was not found.
  - DREB\_HUMAN was not found.
- kegg-mapk
  [+]

  No paths found.
  - MARCS\_HUMAN was not found.
  - DREB\_HUMAN was not found.
- science-signaling-egfr
  [+]

  No paths found.
  - MARCS\_HUMAN was not found.
  - DREB\_HUMAN was not found.

### PARD3\_HUMAN ⊣ PSME3\_HUMAN

#### 1. Resources that have the edge


#### 2. Resources that contain endpoints (but not an edge)


#### 3. Resources that match only one endpoint (fringe)

- phosphositeplus-kinase-substrate
  [+]

  No paths found.
  - PSME3\_HUMAN was not found.

#### 4. Resources that don't match any endpoint

- layek
  [+]

  No paths found.
  - PSME3\_HUMAN was not found.
  - PARD3\_HUMAN was not found.
- kegg-mapk
  [+]

  No paths found.
  - PSME3\_HUMAN was not found.
  - PARD3\_HUMAN was not found.
- kegg-erbb
  [+]

  No paths found.
  - PSME3\_HUMAN was not found.
  - PARD3\_HUMAN was not found.
- cancer-cell-map-egfr1
  [+]

  No paths found.
  - PSME3\_HUMAN was not found.
  - PARD3\_HUMAN was not found.
- pid-erbb1
  [+]

  No paths found.
  - PSME3\_HUMAN was not found.
  - PARD3\_HUMAN was not found.
- reactome-egfr
  [+]

  No paths found.
  - PSME3\_HUMAN was not found.
  - PARD3\_HUMAN was not found.
- science-signaling-egfr
  [+]

  No paths found.
  - PSME3\_HUMAN was not found.
  - PARD3\_HUMAN was not found.
- biocarta-egf
  [+]

  No paths found.
  - PSME3\_HUMAN was not found.
  - PARD3\_HUMAN was not found.

### NUCL\_HUMAN ⊣ PP1B\_HUMAN

#### 1. Resources that have the edge


#### 2. Resources that contain endpoints (but not an edge)


#### 3. Resources that match only one endpoint (fringe)

- phosphositeplus-kinase-substrate
  [+]

  No paths found.
  - PP1B\_HUMAN was not found.

#### 4. Resources that don't match any endpoint

- layek
  [+]

  No paths found.
  - PP1B\_HUMAN was not found.
  - NUCL\_HUMAN was not found.
- reactome-egfr
  [+]

  No paths found.
  - PP1B\_HUMAN was not found.
  - NUCL\_HUMAN was not found.
- pid-erbb1
  [+]

  No paths found.
  - PP1B\_HUMAN was not found.
  - NUCL\_HUMAN was not found.
- biocarta-egf
  [+]

  No paths found.
  - PP1B\_HUMAN was not found.
  - NUCL\_HUMAN was not found.
- kegg-mapk
  [+]

  No paths found.
  - PP1B\_HUMAN was not found.
  - NUCL\_HUMAN was not found.
- kegg-erbb
  [+]

  No paths found.
  - PP1B\_HUMAN was not found.
  - NUCL\_HUMAN was not found.
- cancer-cell-map-egfr1
  [+]

  No paths found.
  - PP1B\_HUMAN was not found.
  - NUCL\_HUMAN was not found.
- science-signaling-egfr
  [+]

  No paths found.
  - PP1B\_HUMAN was not found.
  - NUCL\_HUMAN was not found.

### CHD4\_HUMAN → SMCA4\_HUMAN

#### 1. Resources that have the edge


#### 2. Resources that contain endpoints (but not an edge)


#### 3. Resources that match only one endpoint (fringe)

- phosphositeplus-kinase-substrate
  [+]

  No paths found.
  - SMCA4\_HUMAN was not found.

#### 4. Resources that don't match any endpoint

- pid-erbb1
  [+]

  No paths found.
  - CHD4\_HUMAN was not found.
  - SMCA4\_HUMAN was not found.
- cancer-cell-map-egfr1
  [+]

  No paths found.
  - CHD4\_HUMAN was not found.
  - SMCA4\_HUMAN was not found.
- kegg-erbb
  [+]

  No paths found.
  - CHD4\_HUMAN was not found.
  - SMCA4\_HUMAN was not found.
- kegg-mapk
  [+]

  No paths found.
  - CHD4\_HUMAN was not found.
  - SMCA4\_HUMAN was not found.
- reactome-egfr
  [+]

  No paths found.
  - CHD4\_HUMAN was not found.
  - SMCA4\_HUMAN was not found.
- science-signaling-egfr
  [+]

  No paths found.
  - CHD4\_HUMAN was not found.
  - SMCA4\_HUMAN was not found.
- layek
  [+]

  No paths found.
  - CHD4\_HUMAN was not found.
  - SMCA4\_HUMAN was not found.
- biocarta-egf
  [+]

  No paths found.
  - CHD4\_HUMAN was not found.
  - SMCA4\_HUMAN was not found.

### SCAM3\_HUMAN → HGS\_HUMAN

#### 1. Resources that have the edge


#### 2. Resources that contain endpoints (but not an edge)


#### 3. Resources that match only one endpoint (fringe)

- reactome-egfr
  [+]

  No paths found.
  - SCAM3\_HUMAN was not found.

#### 4. Resources that don't match any endpoint

- science-signaling-egfr
  [+]

  No paths found.
  - SCAM3\_HUMAN was not found.
  - HGS\_HUMAN was not found.
- biocarta-egf
  [+]

  No paths found.
  - SCAM3\_HUMAN was not found.
  - HGS\_HUMAN was not found.
- phosphositeplus-kinase-substrate
  [+]

  No paths found.
  - SCAM3\_HUMAN was not found.
  - HGS\_HUMAN was not found.
- layek
  [+]

  No paths found.
  - SCAM3\_HUMAN was not found.
  - HGS\_HUMAN was not found.
- cancer-cell-map-egfr1
  [+]

  No paths found.
  - SCAM3\_HUMAN was not found.
  - HGS\_HUMAN was not found.
- kegg-mapk
  [+]

  No paths found.
  - SCAM3\_HUMAN was not found.
  - HGS\_HUMAN was not found.
- pid-erbb1
  [+]

  No paths found.
  - SCAM3\_HUMAN was not found.
  - HGS\_HUMAN was not found.
- kegg-erbb
  [+]

  No paths found.
  - SCAM3\_HUMAN was not found.
  - HGS\_HUMAN was not found.

### NUCL\_HUMAN ⊣ NUP62\_HUMAN

#### 1. Resources that have the edge


#### 2. Resources that contain endpoints (but not an edge)


#### 3. Resources that match only one endpoint (fringe)

- phosphositeplus-kinase-substrate
  [+]

  No paths found.
  - NUP62\_HUMAN was not found.

#### 4. Resources that don't match any endpoint

- layek
  [+]

  No paths found.
  - NUP62\_HUMAN was not found.
  - NUCL\_HUMAN was not found.
- kegg-mapk
  [+]

  No paths found.
  - NUP62\_HUMAN was not found.
  - NUCL\_HUMAN was not found.
- biocarta-egf
  [+]

  No paths found.
  - NUP62\_HUMAN was not found.
  - NUCL\_HUMAN was not found.
- science-signaling-egfr
  [+]

  No paths found.
  - NUP62\_HUMAN was not found.
  - NUCL\_HUMAN was not found.
- cancer-cell-map-egfr1
  [+]

  No paths found.
  - NUP62\_HUMAN was not found.
  - NUCL\_HUMAN was not found.
- reactome-egfr
  [+]

  No paths found.
  - NUP62\_HUMAN was not found.
  - NUCL\_HUMAN was not found.
- pid-erbb1
  [+]

  No paths found.
  - NUP62\_HUMAN was not found.
  - NUCL\_HUMAN was not found.
- kegg-erbb
  [+]

  No paths found.
  - NUP62\_HUMAN was not found.
  - NUCL\_HUMAN was not found.

### PP1B\_HUMAN ⊣ DENR\_HUMAN

#### 1. Resources that have the edge


#### 2. Resources that contain endpoints (but not an edge)


#### 3. Resources that match only one endpoint (fringe)


#### 4. Resources that don't match any endpoint

- kegg-erbb
  [+]

  No paths found.
  - DENR\_HUMAN was not found.
  - PP1B\_HUMAN was not found.
- cancer-cell-map-egfr1
  [+]

  No paths found.
  - DENR\_HUMAN was not found.
  - PP1B\_HUMAN was not found.
- phosphositeplus-kinase-substrate
  [+]

  No paths found.
  - DENR\_HUMAN was not found.
  - PP1B\_HUMAN was not found.
- kegg-mapk
  [+]

  No paths found.
  - DENR\_HUMAN was not found.
  - PP1B\_HUMAN was not found.
- reactome-egfr
  [+]

  No paths found.
  - DENR\_HUMAN was not found.
  - PP1B\_HUMAN was not found.
- biocarta-egf
  [+]

  No paths found.
  - DENR\_HUMAN was not found.
  - PP1B\_HUMAN was not found.
- science-signaling-egfr
  [+]

  No paths found.
  - DENR\_HUMAN was not found.
  - PP1B\_HUMAN was not found.
- layek
  [+]

  No paths found.
  - DENR\_HUMAN was not found.
  - PP1B\_HUMAN was not found.
- pid-erbb1
  [+]

  No paths found.
  - DENR\_HUMAN was not found.
  - PP1B\_HUMAN was not found.

### WDR48\_HUMAN ⊣ PSME3\_HUMAN

#### 1. Resources that have the edge


#### 2. Resources that contain endpoints (but not an edge)


#### 3. Resources that match only one endpoint (fringe)


#### 4. Resources that don't match any endpoint

- phosphositeplus-kinase-substrate
  [+]

  No paths found.
  - PSME3\_HUMAN was not found.
  - WDR48\_HUMAN was not found.
- pid-erbb1
  [+]

  No paths found.
  - PSME3\_HUMAN was not found.
  - WDR48\_HUMAN was not found.
- layek
  [+]

  No paths found.
  - PSME3\_HUMAN was not found.
  - WDR48\_HUMAN was not found.
- science-signaling-egfr
  [+]

  No paths found.
  - PSME3\_HUMAN was not found.
  - WDR48\_HUMAN was not found.
- cancer-cell-map-egfr1
  [+]

  No paths found.
  - PSME3\_HUMAN was not found.
  - WDR48\_HUMAN was not found.
- kegg-erbb
  [+]

  No paths found.
  - PSME3\_HUMAN was not found.
  - WDR48\_HUMAN was not found.
- biocarta-egf
  [+]

  No paths found.
  - PSME3\_HUMAN was not found.
  - WDR48\_HUMAN was not found.
- kegg-mapk
  [+]

  No paths found.
  - PSME3\_HUMAN was not found.
  - WDR48\_HUMAN was not found.
- reactome-egfr
  [+]

  No paths found.
  - PSME3\_HUMAN was not found.
  - WDR48\_HUMAN was not found.

### SPB1\_HUMAN ⊣ WDHD1\_HUMAN

#### 1. Resources that have the edge


#### 2. Resources that contain endpoints (but not an edge)


#### 3. Resources that match only one endpoint (fringe)


#### 4. Resources that don't match any endpoint

- cancer-cell-map-egfr1
  [+]

  No paths found.
  - WDHD1\_HUMAN was not found.
  - SPB1\_HUMAN was not found.
- science-signaling-egfr
  [+]

  No paths found.
  - WDHD1\_HUMAN was not found.
  - SPB1\_HUMAN was not found.
- layek
  [+]

  No paths found.
  - WDHD1\_HUMAN was not found.
  - SPB1\_HUMAN was not found.
- phosphositeplus-kinase-substrate
  [+]

  No paths found.
  - WDHD1\_HUMAN was not found.
  - SPB1\_HUMAN was not found.
- kegg-mapk
  [+]

  No paths found.
  - WDHD1\_HUMAN was not found.
  - SPB1\_HUMAN was not found.
- reactome-egfr
  [+]

  No paths found.
  - WDHD1\_HUMAN was not found.
  - SPB1\_HUMAN was not found.
- pid-erbb1
  [+]

  No paths found.
  - WDHD1\_HUMAN was not found.
  - SPB1\_HUMAN was not found.
- biocarta-egf
  [+]

  No paths found.
  - WDHD1\_HUMAN was not found.
  - SPB1\_HUMAN was not found.
- kegg-erbb
  [+]

  No paths found.
  - WDHD1\_HUMAN was not found.
  - SPB1\_HUMAN was not found.

### EPHA3\_HUMAN → EFNB2\_HUMAN

#### 1. Resources that have the edge


#### 2. Resources that contain endpoints (but not an edge)


#### 3. Resources that match only one endpoint (fringe)


#### 4. Resources that don't match any endpoint

- layek
  [+]

  No paths found.
  - EFNB2\_HUMAN was not found.
  - EPHA3\_HUMAN was not found.
- cancer-cell-map-egfr1
  [+]

  No paths found.
  - EFNB2\_HUMAN was not found.
  - EPHA3\_HUMAN was not found.
- biocarta-egf
  [+]

  No paths found.
  - EFNB2\_HUMAN was not found.
  - EPHA3\_HUMAN was not found.
- kegg-erbb
  [+]

  No paths found.
  - EFNB2\_HUMAN was not found.
  - EPHA3\_HUMAN was not found.
- pid-erbb1
  [+]

  No paths found.
  - EFNB2\_HUMAN was not found.
  - EPHA3\_HUMAN was not found.
- reactome-egfr
  [+]

  No paths found.
  - EFNB2\_HUMAN was not found.
  - EPHA3\_HUMAN was not found.
- phosphositeplus-kinase-substrate
  [+]

  No paths found.
  - EFNB2\_HUMAN was not found.
  - EPHA3\_HUMAN was not found.
- kegg-mapk
  [+]

  No paths found.
  - EFNB2\_HUMAN was not found.
  - EPHA3\_HUMAN was not found.
- science-signaling-egfr
  [+]

  No paths found.
  - EFNB2\_HUMAN was not found.
  - EPHA3\_HUMAN was not found.

### WDR48\_HUMAN → WDR70\_HUMAN

#### 1. Resources that have the edge


#### 2. Resources that contain endpoints (but not an edge)


#### 3. Resources that match only one endpoint (fringe)


#### 4. Resources that don't match any endpoint

- layek
  [+]

  No paths found.
  - WDR70\_HUMAN was not found.
  - WDR48\_HUMAN was not found.
- science-signaling-egfr
  [+]

  No paths found.
  - WDR70\_HUMAN was not found.
  - WDR48\_HUMAN was not found.
- pid-erbb1
  [+]

  No paths found.
  - WDR70\_HUMAN was not found.
  - WDR48\_HUMAN was not found.
- reactome-egfr
  [+]

  No paths found.
  - WDR70\_HUMAN was not found.
  - WDR48\_HUMAN was not found.
- kegg-mapk
  [+]

  No paths found.
  - WDR70\_HUMAN was not found.
  - WDR48\_HUMAN was not found.
- cancer-cell-map-egfr1
  [+]

  No paths found.
  - WDR70\_HUMAN was not found.
  - WDR48\_HUMAN was not found.
- kegg-erbb
  [+]

  No paths found.
  - WDR70\_HUMAN was not found.
  - WDR48\_HUMAN was not found.
- biocarta-egf
  [+]

  No paths found.
  - WDR70\_HUMAN was not found.
  - WDR48\_HUMAN was not found.
- phosphositeplus-kinase-substrate
  [+]

  No paths found.
  - WDR70\_HUMAN was not found.
  - WDR48\_HUMAN was not found.

### SVIL\_HUMAN ⊣ KIF4A\_HUMAN

#### 1. Resources that have the edge


#### 2. Resources that contain endpoints (but not an edge)


#### 3. Resources that match only one endpoint (fringe)


#### 4. Resources that don't match any endpoint

- cancer-cell-map-egfr1
  [+]

  No paths found.
  - SVIL\_HUMAN was not found.
  - KIF4A\_HUMAN was not found.
- layek
  [+]

  No paths found.
  - SVIL\_HUMAN was not found.
  - KIF4A\_HUMAN was not found.
- phosphositeplus-kinase-substrate
  [+]

  No paths found.
  - SVIL\_HUMAN was not found.
  - KIF4A\_HUMAN was not found.
- science-signaling-egfr
  [+]

  No paths found.
  - SVIL\_HUMAN was not found.
  - KIF4A\_HUMAN was not found.
- kegg-erbb
  [+]

  No paths found.
  - SVIL\_HUMAN was not found.
  - KIF4A\_HUMAN was not found.
- reactome-egfr
  [+]

  No paths found.
  - SVIL\_HUMAN was not found.
  - KIF4A\_HUMAN was not found.
- pid-erbb1
  [+]

  No paths found.
  - SVIL\_HUMAN was not found.
  - KIF4A\_HUMAN was not found.
- biocarta-egf
  [+]

  No paths found.
  - SVIL\_HUMAN was not found.
  - KIF4A\_HUMAN was not found.
- kegg-mapk
  [+]

  No paths found.
  - SVIL\_HUMAN was not found.
  - KIF4A\_HUMAN was not found.

### DDX55\_HUMAN ⊣ SPB1\_HUMAN

#### 1. Resources that have the edge


#### 2. Resources that contain endpoints (but not an edge)


#### 3. Resources that match only one endpoint (fringe)


#### 4. Resources that don't match any endpoint

- biocarta-egf
  [+]

  No paths found.
  - DDX55\_HUMAN was not found.
  - SPB1\_HUMAN was not found.
- layek
  [+]

  No paths found.
  - DDX55\_HUMAN was not found.
  - SPB1\_HUMAN was not found.
- kegg-mapk
  [+]

  No paths found.
  - DDX55\_HUMAN was not found.
  - SPB1\_HUMAN was not found.
- kegg-erbb
  [+]

  No paths found.
  - DDX55\_HUMAN was not found.
  - SPB1\_HUMAN was not found.
- reactome-egfr
  [+]

  No paths found.
  - DDX55\_HUMAN was not found.
  - SPB1\_HUMAN was not found.
- cancer-cell-map-egfr1
  [+]

  No paths found.
  - DDX55\_HUMAN was not found.
  - SPB1\_HUMAN was not found.
- phosphositeplus-kinase-substrate
  [+]

  No paths found.
  - DDX55\_HUMAN was not found.
  - SPB1\_HUMAN was not found.
- pid-erbb1
  [+]

  No paths found.
  - DDX55\_HUMAN was not found.
  - SPB1\_HUMAN was not found.
- science-signaling-egfr
  [+]

  No paths found.
  - DDX55\_HUMAN was not found.
  - SPB1\_HUMAN was not found.

##
